# An Integrated Plasmonic Sensing Array for Chemical Fingerprinting and Flavor Profiling in Beverages and Other Liquids

**DOI:** 10.1021/acssensors.5c02485

**Published:** 2025-10-03

**Authors:** Justin R. Sperling, Daniel D. Osborne, Badri Aekbote, Anthony E. Perri, Rebecca A. Setford, Hanyu Gao, Liam T. Wilson, Chad M. Sipperley, Rudolf J. Schick, Caroline Gauchotte-Lindsay, William J. Peveler, Alasdair W. Clark

**Affiliations:** † James Watt School of Engineering, Advanced Research Centre, 3526 University of Glasgow, Glasgow G11 6EW, United Kingdom; ‡ School of Chemistry, Joseph Black Building, University of Glasgow, Glasgow G12 8QQ, United Kingdom; § Spray Analysis and Research Services, 682575 Spraying Systems Co., Wheaton, Illinois 60139, United States

**Keywords:** plasmonics, sensor array, artificial olfaction, droplet microarray, hyperspectral imaging

## Abstract

We report a reusable cross-reactive plasmonic sensing system that generates unique optical fingerprints for any liquid mixture. The platform leverages a multiplexed plasmonic chip and droplet microarray methodology for creating 24 orthogonally modified sensors coupled with hyperspectral imaging. We demonstrate the versatility and sensitivity of our platform by distinguishing not only between broad categories of chemically diverse beverages but also between individual products within those categories, capturing subtle chemical variations even within highly dilute samples, such as mineral waters. Capable of rapidly fingerprinting liquid samples and measuring the kinetics of reversible supramolecular interactions underlying those fingerprints, this technology represents a significant advance in cross-reactive liquid-phase sensor arrays. Our portable tool provides a practical solution for QA/QC in beverage production, a platform to extend liquid fingerprint analysis beyond food and drink, and, with array expansion, the potential to profile the complex molecular attributes that shape taste and flavor.

## Graphical abstract

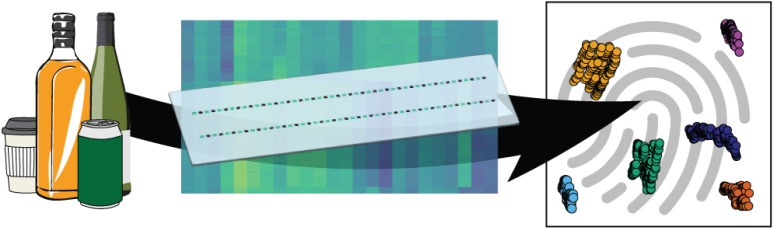

Smell and taste are the senses most closely linked to emotion, while also offering immediate feedback on the nutrition and safety of the food and drink we consume.[Fn fn1] ^1–3^ The tongue allows us to perceive core flavor profiles (salt, sweet, sour, bitter, and umami), but in mammals, volatilized portions of what we eat are passed up the back of the throat to the olfactory bulb, enabling us to perceive many more flavors (retronasal olfaction). However, mammalian taste and smell do not work like typical analytical chemical instruments, for example, a gas chromatograph (GC) or mass spectrometer (MS).^4^ Instead, it relies on the adsorption of different flavor molecules into a variety of cross-reactive cellular receptors (that do not target just one molecule), triggering a pattern of nervous impulses the brain decodes to generate the overall flavors perceived. In this scheme, not every flavor molecule will make an impact (depending on both binding to the sensor and weight in the analysis), and rather than individual molecules being identified, the overall pattern is matched to a perception of flavor.^5,6^

For this reason, smell and taste can be challenging to predict from molecular (GC or LC-MS) analysis, leading many manufacturers to rely on human tasters or taste panels to assess the product quality and consistency between batches. However, the use of human tasters presents challenges such as taste blindness to very strong flavors and the need for rigorous ingredient checking, which can be unpalatable. Moreover, in cases in which products pose health risks or are toxic, human testing is impractical and unsafe. The expense of maintaining highly trained tasters can also limit the testing frequency. Therefore, there is growing interest in developing point-of-need or in-line sensors that can offer reliable assessments in scenarios in which human testing is impractical or hazardous.

Researchers and manufacturers have therefore turned to artificial taste and olfaction systems (often referred to as e-noses) that employ the cross-reactive adsorption of flavor compounds over many sensor regions, as in the mammalian olfaction system, but with an electronic or optoelectronic readout that is processed in pattern recognition algorithms.^7–10^ This has been long exploited for gas sensing, with arrays of resistive gas sensors employed in headspace and odor analysis,^11–13^ but has been far less developed for tasting liquids and the exploration of what less volatile chemistries might be detected retronasally or on the taste buds.^14,15^ The majority of optical liquid sensing arrays in the literature rely on multisample, multisensor homogeneous assays in well plates that are not suited to continuous in-line or online analysis at a manufacturing site due to their limited multiplexing potential.^8,16,17^ While spatially multiplexed paper-based assays have also had success, these are inherently one-time devices, also limiting applications in manufacturing.^18–20^

We describe here a massively multiplexed plasmonic metasurface sensing array for the rapid, repeatable fingerprinting of liquid foodstuffs. The premise of this sensing scheme is based on cross-reactivity, so no one sensing element within the array is specific to one thing and one thing only (so-called “lock-and-key”); rather, the patterns that arise from the interactions of lots of different molecules with the different array elements are used to identify liquid mixtures. We bias the interactions of each sensing element to different molecules by altering their hydrophobicity and charge, including macrocyclic preorganization and other strategies. Thus, the binding of each modification is not specific, but the fingerprint arising from all of the modifications interacting with a liquid will be. Similar liquids should develop similar fingerprints as a function of the relative concentration of different components within them; liquids with very different concentrations of similar components or very different components altogether generate quite different fingerprints.

We have previously demonstrated that plasmonic sensors coated with such varied self-assembled monolayers can be used in this way, where local supramolecular interactions between the monolayer and the sample result in the local concentration of particular species around particular array elements, shifting their plasmonic resonance.^21,22^ Individually prepared sensors with six surface chemistries could identify and differentiate whiskeys based on the liquid chemical content.^23^ Subsequently, 8-element sensor arrays integrated with microfluidics enabled continuous water monitoring, using an automated microscope stage to measure each sensing element in turn.^24^ While effective for steady-state measurements, this setup lacked the flexibility to achieve kinetic resolution and limited the number of measurable elements due to the need to individually probe each sensor in the array.

By incorporating many more sensor elements, novel chemical modifications, simultaneous readout, and reusability, the technological advancement we describe herein significantly increases the functionality of cross-reactive liquid-phase sensor arrays. While 96-well plate plasmonic arrays have been previously described,^25^ our high degree of spatial multiplexing allows us to monitor each sensing element of our hyperspectral chip simultaneously, and our optical patterns are driven by cross-reactive mechanisms that are inherently reversible. This better mimics the taste/olfaction system, and while patterns can be generated instantly, the chip is also fully reusable for in/online liquid sensing applications. To demonstrate the potential of this sensory system for the food and beverage industry, we use a single chip to taste a wide range of beverages, showing highly sensitive differential responses both between and within six different classes of liquid and identification of subtle distinctions within those classes. We go on to show that our new sensing modality is uniquely placed to make real-time kinetic measurements of reversible supramolecular interactions occurring across the array. Given that the kinetic regime of supramolecular adsorption is likely a key part of flavor perception,^26,27^ the ability to track these changes under flow conditions positions the technology as a truly exciting development that will not only serve as a valuable liquid QA/QC tool but also has the potential to bridge the gap between analytics and flavor perception.

## Results

### Design and Optimization of Optics and Surface Chemistry for Nanoplasmonic Sensing Arrays

The plasmonic chip consists of a Borofloat glass (Schott Nexterion) substrate of microscope slide dimensions (75 mm × 25 mm × 1 mm), with 24 sensing regions, each containing gold nanostructures comprising 146 ± 4 nm-sided, 56 ± 4 nm-tall squares, in an array with a period of ∼390 nm in X and Y (Figures 1a–c and S1). To allow hyperspectral transmission imaging, the top surface of the slide is rendered opaque by the deposition of a 130 nm-thick multimetal (Au and Ti) film, optimized for light exclusion. Within this layer, 55 slit-shaped apertures (25 × 500 μm, spaced 750 μm apart) are defined, where the outer 4 on each side are “blanks” and the inner 47 alternate between the Au nanostructures (24×) and additional “blanks” (23×), which act as local light references for measurement (Figure 1b).

**Figure 1:**
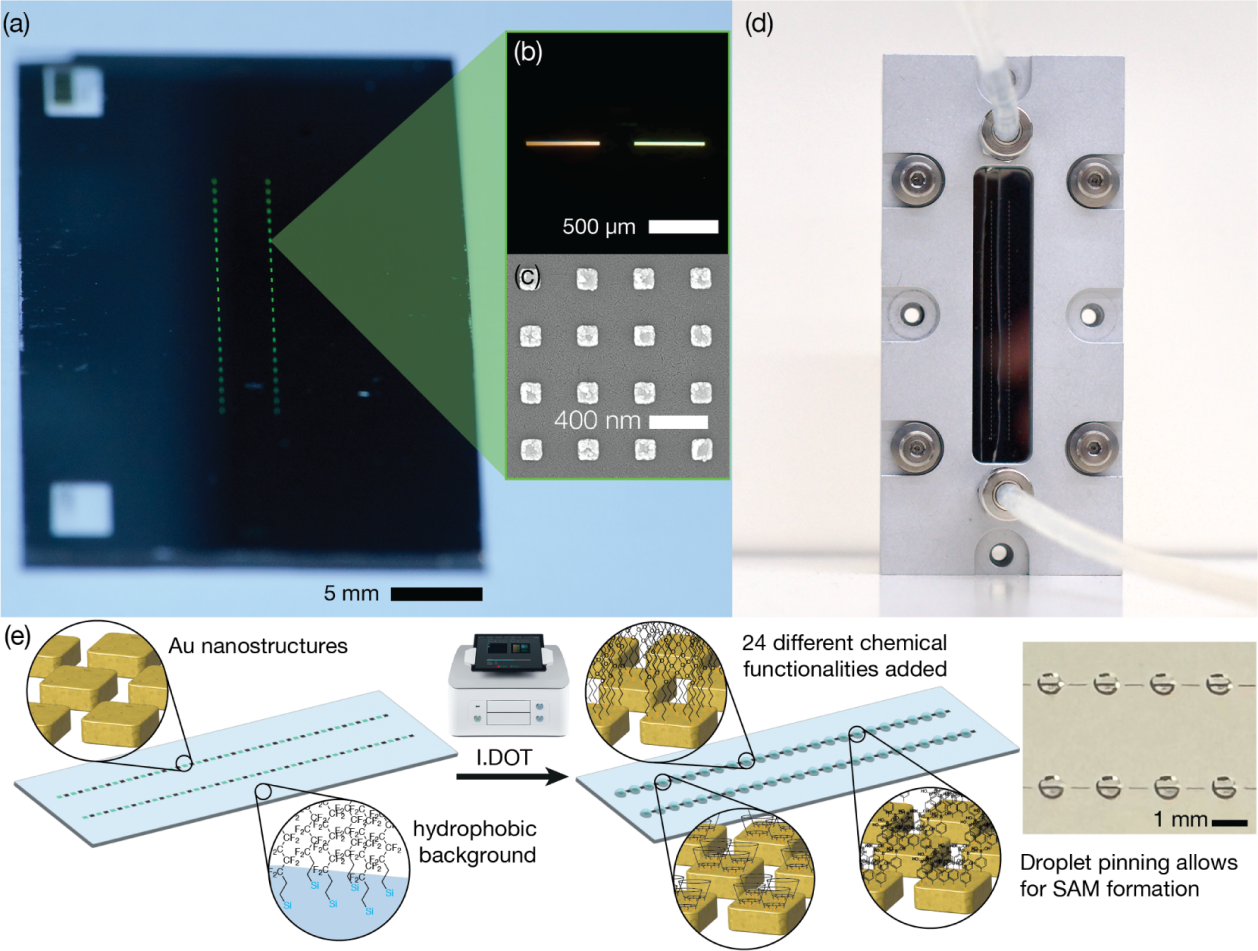
(a) Oblique image of a sensor chip featuring two rows of sensors (scattering green light) separated by empty light references. (b) Close-up image of a sensor element in the aperture (right) next to its light reference (left). (c) Representative SEM of the gold nanostructures on the sensor surface. (d) Sensor chip mounted in a holder with a liquid interface and PDMS channel shown. The sensor is measured in transmission with light filling the back side of the sensor and measurement taking place on the front side. (e) Schematic of the orthogonal creation of self-assembled monolayers at each sensing region with an I.DOT noncontact liquid dispensing tool, using compressed air to create 100 nL droplets at each sensor region in the array. Inset shows an image of the droplets incubating on a sensor.

The unmodified sensor regions have a plasmonic resonance at 709 ± 4 nm in water (Figure S2). These resonances are particularly sensitive to changes in local RI, meaning that when in contact with different liquids, the sensors shift in color by a measurable amount (>0.1 nm). The principle of our sensing system relies on each sensing element producing an observable resonance shift that is linked to the presence of a particular subset of chemicals present in the sample. By doing so, we can produce a 24-color pattern or “fingerprint” that is linked to the unique chemical content and concentration of the sample. It should be noted that while we have used 24 sensor regions here for the purposes of practicality with our surface functionalization method and use of microscope slide substrates (*vide infra*), varying the length of the channel and the size and spacing of the sensor regions would enable hundreds of sensor regions to be measured simultaneously with the same optical system.

Immediately postfabrication, all 24 nanostructured regions are the same and would respond uniformly to any liquid, producing no unique pattern; all experiencing the same resonance shift linked to the bulk RI of the liquid and its adsorption to bare gold surfaces. To produce a cross-reactive response, we engineer each region to interact only with molecules with certain chemical properties, producing an RI-induced resonance shift driven by those molecules alone and not from the bulk liquid. Since the nanostructures are only sensitive to their *immediate* surroundings—a sensing volume that extends only a few nm from their surface—we can influence the molecular content of this volume, and thus which molecules are probed by each sensor element, by modifying the nanostructures with chemical groups that “gate” access to the volume via *repulsion/attraction/-philicity/-phobicity* of certain molecular properties.

The titanium top layer of the chip was modified with a monolayer of heptadecafluoro-1,1,2,2-tetrahydrodecyl trimethoxysilane, patterned to leave a circular hydrophilic region over each sensor element. Modification of the sensor elements was performed using our drop-on-demand printing process.^28^ An I.DOT contactless liquid deposition tool was used to dispense a 100 nL droplet of a different functional thiol, at 1–20 mM in a suitable low-volatility solvent (water, mixtures of ethanol and ethylene glycol, or DMSO), onto each distinct plasmonic region (Figure 1e and Table S1). After incubation in a humidified atmosphere at 4 °C overnight, a different chemical SAM was produced for each of the 24 sensing regions. The presence of the thiols was verified previously by surface-enhanced Raman spectroscopy,^24^ and here by X-ray photoelectron spectroscopy (Figures S3 and S5).

The 24 chemicals chosen for the modification process in this work are shown in Scheme 1, and additional chemistries have also been previously tested (Table S1). Molecules were selected with a range of hydrophilicities/phobicities, both fixed or pH-dependent positive and negative charges, π-electron content, as well as supramolecular multidentate/cavitand binding opportunities. Many of the molecules used here were developed specifically for the project, and their synthesis was invented or optimized (details in Supporting Information).

**Scheme 1:**
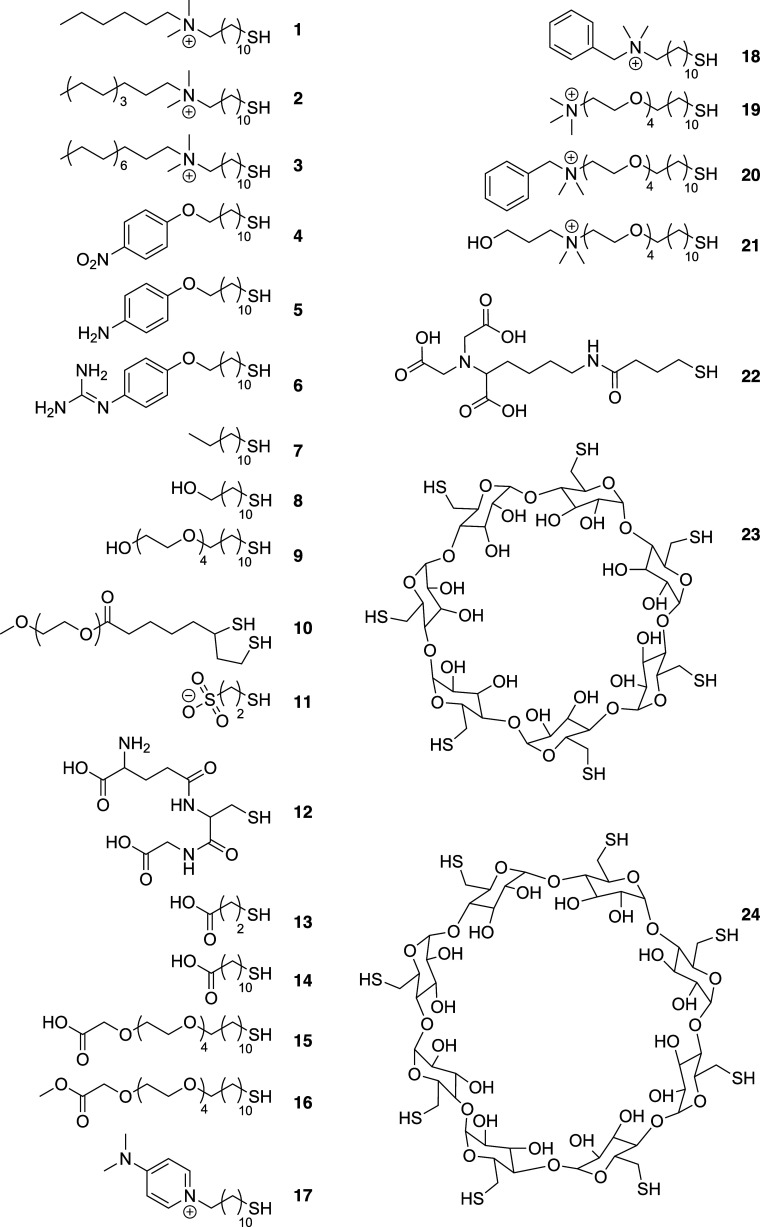
The Chemistries Applied to Each Sensor Element (Bold Numbers) as Self-Assembled Monolayers

To create a diverse library of functionalities in our SAMs, a standard structure was used consisting of a linear carbon chain (typically 11 carbons) to ensure good monolayer packing, and then an optional tetraethylene glycol spacer to further improve packing and also add hydrophilicity, followed by the headgroup.^29,30^ A range of similar chemical headgroups that differed in chain length/construction were also incorporated for comparison, and these were supplemented with a molecule featuring a dithiol lipoic acid anchor (**10**), zwitterionic peptides such as glutathione (**12**), a strong cation chelator (**22**), and two macrocyclic supramolecular hosts of differing cavity sizes, beta- (**23**) and gamma- (**24**) cyclodextrin. While we have focused on single chemistries per element here, it would be trivial to create mixed monolayers or postfunctionalized monolayers with the same approach and will be the topic of future exploration.

Once the chip is fabricated, liquid samples are delivered over the surface using a single PDMS microfluidic channel (62 mm × 2 mm × 1.3 mm) that runs the length of the chip (Figure 1d). The microfluidic chamber is clamped onto the plasmonic chip using a machined aluminum holder, with the clamping pressure being sufficient to contain the liquid without permanent bonding of the fluidic channel to the chip. Samples are delivered to the chamber using platinum-cured silicone tubing (MasterFlex L/S 13) connected to inlet/outlet ports in the aluminum holder via a threaded connector with an O-ring. The whole system volume (tubing and channel) is approximately 600 μL.

### Hyperspectral Measurement of the Plasmonic Sensing Array

Measurement of the sensing chip is made via hyperspectral imaging using white light illumination (150 W tungsten halogen bulb), a diffraction grating, and a monochrome CMOS camera sensor (Figures 2a and S4). The instrumentation has been codeveloped and manufactured with Spraying Systems Co. into a small-footprint (∼0.2 m^2^), rail-mounted functional prototype tool. The chip is mounted vertically, with all the sensory regions illuminated simultaneously from the “backside” of the sensor. The known position of the apertures enables hyperspectral imaging of the full chip; each aperture produces a spatially separated column of transmitted light on the camera, where the horizontal position of the column is the physical location of the aperture on the sensor, and the vertical position on that column represents transmission at a particular wavelength. The resonance peak of each sensing region appears as a darkened, lower-transmission band inside the column (Figure 2b). This image is converted into a set of transmission spectra (Figure 2c), from which the transmission minimum for each sensor is determined. Each sample measurement is compared to a “standard” fluid of ultrapure deionized (DI) water, so that shifts away from that standard can be recorded for all 24 sensors. The magnitude of these 24 shifts from the standard fluid comprises the sample’s plasmonic fingerprint (Figure 2d).

**Figure 2:**
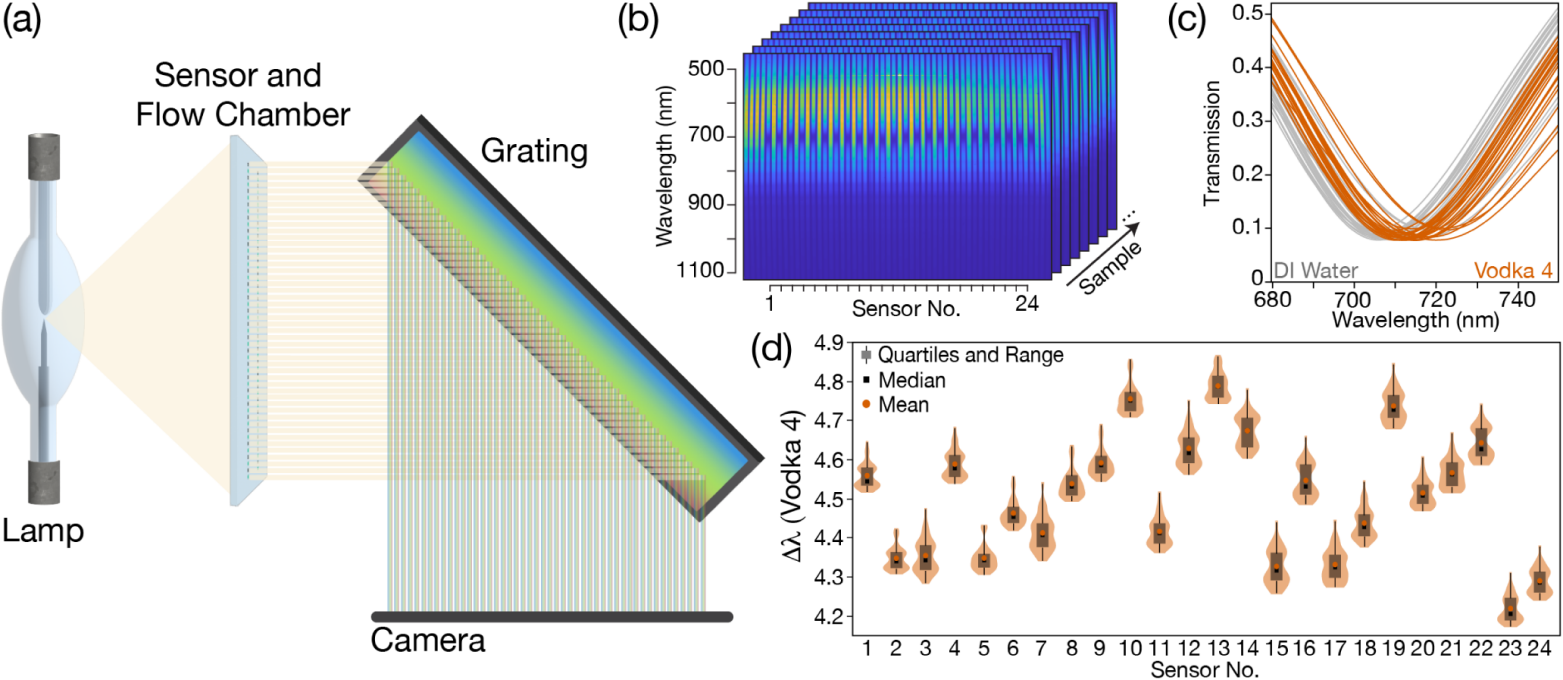
(a) Schematic of the hyperspectral system (an image of the system is shown in Figure S6). (b) The hyperspectral images are collected for each sample and feature the light reference and transmission spectrum for each element, and (c) are converted into transmission curves. (d) The difference between each of the 24 sensor elements for the sample in question and a standard ultrapure DI water sample is used to create the sample fingerprint. Here the repeat measurements for one sample (Vodka 4) across multiple runs are shown.

### Sample Measurement

Multiple commercially available examples from six liquid “classes” were tested over the course of 3 weeks using the same sensor chip. The classes comprised mineral water (6 examples), beer (6 examples), white wine (8 examples), whiskey (5 examples), vodka (4 examples), and gin (6 examples); 35 in total. A full table of individual sample identities is provided in Table S3. Individual samples were introduced to the chip using a syringe to fill the microfluidic chamber (using an excess of sample to ensure complete exchange of liquid in the tubing and chamber; Figure 1d). Each sample was introduced to the chip on 3 occasions, with the measurement order randomized among all samples in that liquid class. For every individual sample introduced, 10 readings of the 24-part plasmonic fingerprint were taken, one every 30 s, to capture any kinetic development of the fingerprint. Beer was the exception, where 20 readings of samples were taken, one every 30 s, to account for the greater observed time-dependent interaction of beer with the surface of the chip (*vide infra* Figure 4 and surrounding discussion).

In other experiments, the samples explored included fruit juices, red wines, and other spirits, such as rum, tequila, and coffee (Figures S5 and S6), but these are considered separately. A further study was undertaken on a subset of the vodka samples across two sensors, 3 weeks, and two separate instruments to demonstrate the coherence of the measurements (Figure S7).

To ensure that operation could be maintained without disassembling the sensor unit, a rinsing protocol was developed offline for each liquid class (further details in Methods section and Supporting Information). Complete rinsing was measured by observing the sensor’s response revert to its baseline value in DI water. Regardless of the liquid class, the system was first rinsed with ultrapure DI water. For the mineral water, vodka, and gin samples, this single rinse was enough to clean the sensor. Whiskey samples required additional rinses of ethanol followed by water. For wine and beer, a mild surfactant cleaning was necessary, so a small volume of 0.1 M sodium dodecyl sulfate (SDS) in DI water was introduced to the chamber with a “dwell” of 5 min before being rinsed out with DI water. When not in use (e.g., overnight), the chip was stored in ultrapure DI water and then flushed with fresh water before use.

While this is an inherently reusable technology, and the same chip was used over the course of this experiment, we have developed a method to recondition the sensor element’s chemical modification if any performance loss is identified. To do this, we strip the modification from the nanostructures using sodium borohydride,^31^ leaving the plasmonic features and the hydrophobic surface layer intact, enabling remodification with a new SAM via the droplet deposition method (Figure S8).

### “Universal Sensor” Demonstration

The plasmonic fingerprints for each of the 35 beverage samples across the six classes were collated and used to distinguish and classify the samples. Each sample produced a unique plasmonic fingerprint versus the DI water background, and samples from within the same class tended to produce similar but different fingerprints. These are shown in their averaged form (Figure 3a), and individual fingerprints are shown in Figure S9, with the underlying data provided as a supplementary data file. Immediately obvious is the influence of alcohol by volume (ABV) on the overall shift from water, as might be expected by the change in RI caused by the ethanol content of the sample. Measurements of RI on samples with the same ABV also revealed additional changes beyond alcohol content (attributed to dissolved sugars and other matter, *vide infra*). These dissolved compounds are significant drivers of the discrimination that we observe.

**Figure 3:**
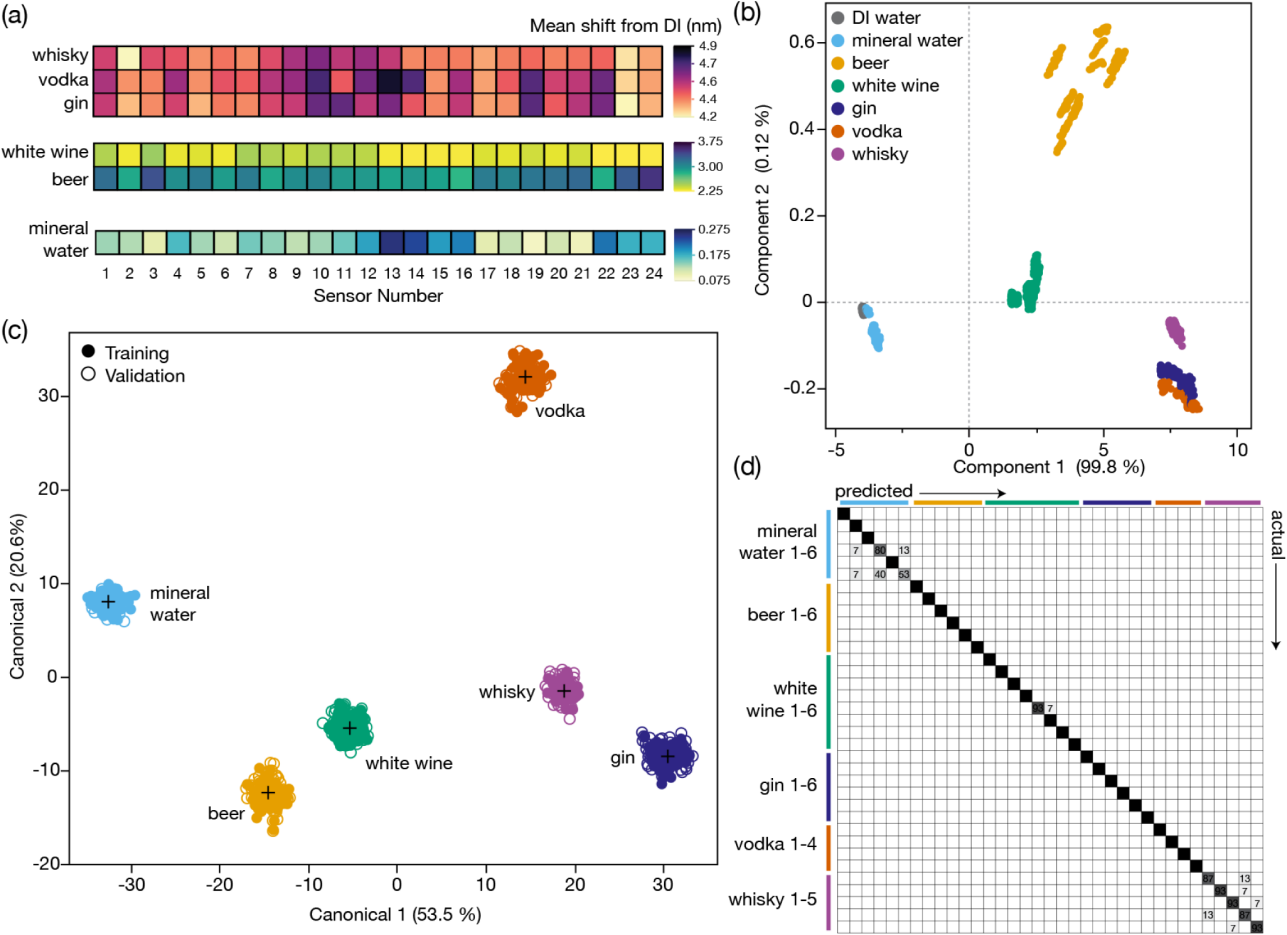
(a) Averaged fingerprints for each of the six liquid classes containing multiple examples of each. Clear distinctions can be seen. (b) PCA of the individual samples, measured in triplicate, and colored by class membership. The DI water zero value is also shown. The first component (PC1) is driven by ABV but also sample content beyond alcohol percentage, and Component 2 (PC2) contains additional information for separating the sample classes. (c) Supervised LDA to distinguish classes based on using half the data to train the model and half to test. The training model and test models for class distinction were 100% accurate. (d) When the LDA model is trained by individual sample ID instead, it still achieves 96.5% accuracy, with all confusions being intraclass (e.g., water for water or whiskey for whiskey). Numbers shown are the percentages of samples in the confusion matrix position.

An unsupervised principal component analysis (PCA) to explore the natural multivariate clustering of all the data shows clear differences between the classes of samples. The majority of separation is on the first component (PC1), likely driven by the local absorbance of materials and changes in the RI (Figure 3b) as confirmed by the equal contribution of all 24 elements to the first component (Figure S10). The second and third components provide significant separation between samples that are in similar positions on the PC1 (Figure S10). Crucially, for most of the liquid classes, the individual samples visibly separate within their overall class grouping, even when they have the same RI and ABV, indicating that the multivariate sensor interactions with dissolved molecules in the samples contribute to the separations observed in the major components.

To demonstrate the ability of the chip to classify both different classes of liquids and different examples of the same class, a supervised discriminant analysis model was applied to both the data classes (Figure 3c) and individual samples (Figure 3d). The total data set (30 measures comprising 10 measurements on each liquid over 3 replicates) was split into equally sized training and validation sets using a stratified but random split to ensure every individual liquid was represented equally in both the testing and validation sets. When using the discriminant model to categorize by sample class, 100% classification accuracy was achieved with the validation set. When individual samples were interrogated, 18 out of the 520 validation samples were misclassified (Figure 3d), giving an accuracy of 96.5%. The highest confusion was seen within the mineral water and whiskey classes, and all misclassified samples were intraclass (i.e., whiskey was confused with another whiskey, not a gin or vodka).

### Sample-Sensor Interactions

The untargeted analysis was explored in more detail to elucidate which sensor elements are driving the different sample separations. The second component of the PCA features a heavier weighting of sensors **3** (a hydrophobic chain hiding a fixed positive charge), **13** (a short-chain carboxylate anion), **16** (a long-chain polarizable but neutral ester), **22** (a strong cation receptor), **23**, and **24** (the two supramolecular cyclodextrin molecular cavitands). This component largely separates the beer class from other samples but also draws a distinction between the three groups of spirit drinks and also between DI water and several of the mineral water samples.

It was notable that, despite all the water samples having the same measured RI to the third decimal place, mineral water (MW) 3 sits far closer to the DI water control than the others and, on examination of the published analytical data for this product (Table S4), has notably low total dissolved solids compared to many other mineral water brands. MW1 and MW5 also separate toward the DI water control, again explainable by a lower mineral content. Based on control experiments with monovalent and divalent cations, and known chemical interactions between carboxylate chemistries 13, 14, 15, and 22, as well as macrocycles 23 and 24 with cations, we see a particularly strong response from divalent cations, in the presence of monovalent cations, on several elements of our sensor that explain this sensitivity (Figure S11).

For some liquid classes, particularly beer, sensor response is time-dependent. Figure 4b shows clear elongation of the first and second PCs of the beer samples over the course of a 10-min measurement process versus other samples that remained more compact. In separate experiments, similar trends were observed for coffee (Figure S6), suggesting that these interactions are perhaps driven by larger macromolecules/proteins that diffuse to the sensor surface more slowly or more weakly binding molecules. In our analysis, the models can differentiate these samples based both on an “end-point” measure at time 10 min, or using the parameters from a kinetic “on-curve” fitted to the plasmonic response for each sensor. The parameters of this curve can also be used as an extra point of discrimination for particular samples (Figure S12). We believe this kinetic data will be useful in the future for further discriminating between samples on the basis of complex chemical content interacting with the sensor, better matching how we perceive flavor, and this highlights the importance of our multiplexed array measurement system, capable of capturing kinetic data with sub-2 s resolution.

**Figure 4:**
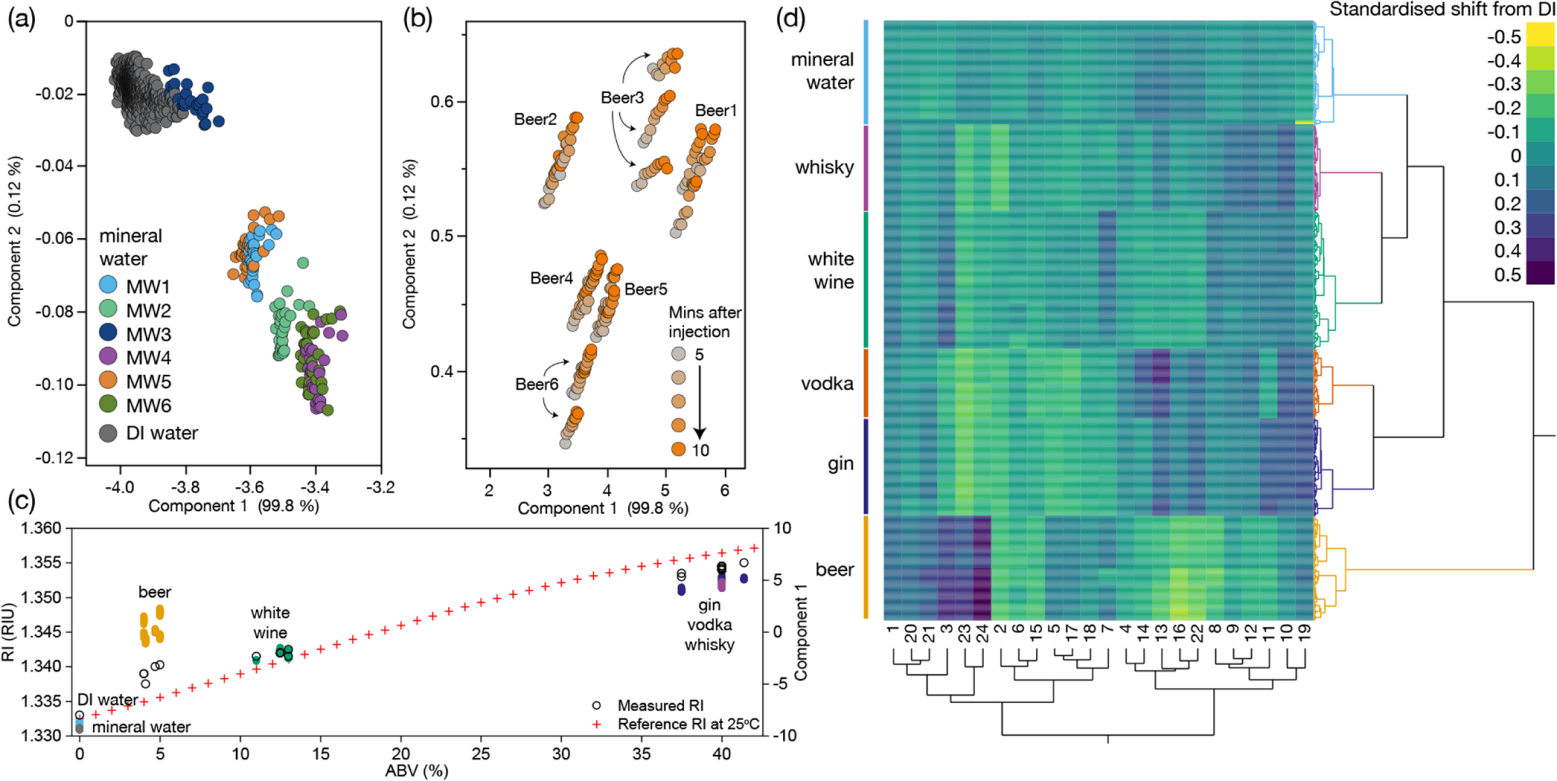
(a) Zoom-in of Figure 3b to show mineral water (MW) grouping compared to the DI water “zero”. (b) Zoom-in of Figure 3b to show the Beer grouping, with coloration by measurement time from 5 to 10 min after introduction (every 30 s, 10 measurements), showing the increase in Component 2 over time that stabilizes after around 8 min. (c) Plot of sample ABV (stated by the manufacturer) against measured RI (open circles) and position on Component 1 (colored circles). A reference measurement for ethanol in water is shown in red crosses with data from Scott Jr. measured at 25 °C.^32^ (d) When the mean shift for each sample measurement is removed to account for the bulk shift in RI, unsupervised hierarchical clustering analysis can still accurately group the classes of sample (colored tree on the right) and distinguish individual samples with easily visualized fingerprints. 2D analysis enables subgroups of the surface chemistries that contribute to the separation to all be visualized (tree underneath).

The impact of RI was explored further by comparing the measured RI, manufacturer-reported ABV, and first principal component (PC1) (Figure 4c). We can see that for several liquids, RI differed from that predicted by ABV alone: beer had a larger RI, likely due to the higher sugar and protein content compared to other samples, while spirits tended to have a slightly lower RI than might be expected by the ABV alone. There was a strong correlation of these parameters, as expected, to sensor response, but it was notable that for many samples (and for beer in particular) that PC1 did not match what would be predicted if it was being driven by ABV (or RI) alone. Many samples with matched RIs or ABVs were separated in the PCA (Figure S5). This indicates that while ABV is the main contributor to PC1, this component’s value is being driven in part by differential chemical response, with yet further discrimination coming from PC2 and PC3 (as stated above).

To explore this impact of ABV/RI on the data and demonstrate that the chemistries on the chip were reporting on the content of the samples, not just bulk RI, the data for each of the 24 measurements for a sample were standardized by subtracting the overall mean for those measurements. This effectively removes the bulk RI shift caused by dissolved alcohol while leaving the relative variations across the 24-sensor fingerprint unaltered. Further untargeted PCA (Figure S13) and a Hierarchical Clustering Analysis were carried out on this centered data, and not only is the clear separation of liquid classes maintained, but the chemical fingerprints for individual samples within a particular class become clear enough to easily visualize (Figure 4d). For example, sensors 3, 23, and 24 contributed to the discrimination of beer and mineral waters, whereas sensors 13, 14, and 22 contributed to mineral water ID, while also separating the spirit drinks. Overall, all 24 chemistries made useful contributions, highlighting the universality of our multisensor array.

## Conclusion

We demonstrated a reusable cross-reactive metasurface sensing array capable of generating plasmonic fingerprints for liquid mixtures. Testing with different beverage types, we show that distinct fingerprints are produced for each class of beverage and for individual products within those classes; fingerprints that are tied to subtle differences in the chemical content of the sample and not solely due to their color, RI, or ABV. A key feature of this technology is its versatility. Our array of 24 cross-reactive sensors is performant across a wide variety of products with variable chemical content, while also demonstrating high sensitivity (a few mg/L or lower) across many very similar samples (e.g., mineral waters). Furthermore, the chip is reusable, a simple washing step preparing the chip for the next sample or product class, and it is possible to recondition the chip’s chemical modifications after prolonged use. Our combination of lithographically defined sensor arrays with a hyperspectral imaging system allows us to strictly control the optical performance, location, and chemical modification of each sensor and to probe the sensor array simultaneously, rapidly producing the fingerprint for each sample. The system lends itself to scalability far beyond 24 sensor elements, with hundreds of sensors possible—a move that would bring even greater granularity of sample analysis (more detailed, more distinct fingerprints), which could be used to analyze extremely similar liquids where detecting low levels of difference/contamination is required and to quickly gather the depth and breadth of data that are required to link sensory output to perception.^33^ In its current state, this system could provide beverage manufacturers with a valuable QA/QC tool at the point of need or in-line, and in the future, with further development, this system may allow one-to-one mapping of fingerprints to a sample’s molecular content and its perceived flavor for product development. Limits of detection would be best determined by the end user for their given sample and scenario. Beyond food and beverage applications, this advance in liquid-phase sensor arrays provides the first true equivalent to the array-based e-nose technology used for gases, now enabling the same cross-reactive, multielement, reusable sensing capabilities for liquid samples. As such, the technology now has the potential to be extended to other critical fields such as environmental monitoring, security and defense, and healthcare (e.g., diagnostics through biological fluid fingerprinting).

### Methods

A detailed chemical synthetic methodology and details of standard instrumentation used are given in the Supporting Information. Throughout this work, ultrapure deionized (DI) water was obtained from an ELGA PURELAB Chorus 1 Complete, Recirculating Type 1 Ultrapure Water system with 18.2 MΩ·cm resistivity.

### Sensor Fabrication

The nanoplasmonic sensing regions and photolithography alignment markers were fabricated onto a borofloat glass sample (75 mm × 25 mm × 1 mm, Schott Minifab). Electron-beam lithography (Raith EBPG 5200) was used to pattern a bilayer of poly­(methyl methacrylate) resists (AR-P 642.04/200k/4% in anisole and AR-P 679.02/950k/2% in ethyl lactate) followed by metal evaporation (Plassys MEB 550s) of a 2 nm titanium adhesion layer and 50 nm of gold. Acetone liftoff was then performed to remove unwanted metal. The sensor was then annealed (Jiplec RTA) at 500 °C for 10 min. In the second stage, the opacity layer was added by patterning S1818 (DuPont) resist via UV photolithography (Suss MA-BA8 Optical Mask Aligner), followed by metal evaporation of a 130 nm thick multimetal gold and titanium film. Acetone liftoff was used to remove unwanted metal. In the final patterning step, photolithography of S1818 resist and subsequent silanization of the surface with heptadecafluoro-1,1,2,2-tetrahydrodecyl trimethoxysilane (Gelest) defined hydrophobic regions around the sensor apertures.

### Sensor Modification

To print a ligand of interest, an “ink” was formulated. The inks were formulated with a range of solvents (80:20 EtOH:ethylene glycol, DMSO, or water) and concentrations (1–20 mM) based on the compounds and their solubilities (Table S1). For most compounds, an 80:20 (v/v) EtOH:ethylene glycol mixture was used, as it was found to have a good balance of viscosity and volatility while remaining a good solvent. Where compounds were water-soluble, then this was used as a green solvent. The two per-thiolated cyclodextrins (**23** and **24**) were formulated as 1 mM inks in DMSO due to their poorer solubility in water or EtOH:ethylene glycol mixtures. Molecules HO-PEG_750_-Lipoic Acid (**10**), **23**, and **24** were each subjected to 10, 70, and 80 mol equiv of TCEP, respectively, prior to printing to help cleave disulfides and maximize the number of free thiols present.

Once the inks were formulated, 40 μL of each ink was dispensed into Dispendix I.DOT “100” series source wells (80 μL capacity, 1 μL dead volume) and loaded into the noncontact liquid dispenser (I.DOT Liquid Handler, Dispendix). After priming each source well, 100 nL volume droplets were printed at each sensor region, with the droplets being pinned by the local perfluorinated surface. The sensor was then placed in a clean plastic Petri dish with a damp folded towel for humidification and sealed with parafilm tape before being placed in the fridge at 4 °C overnight to allow for SAM formation on each sensor to occur. After 16–24 h, the droplets remained, and the slide was rinsed at a very high dilution (rapid submersion in over 100 mL of ethanol, followed by an ethanol and DI water rinse) to ensure no intermixing of the surface chemistries occurred. The sensor was then dried with a dry nitrogen stream before being installed into the device.

### Measurement

To introduce the sample to the plasmonic chip, a single-channel PDMS (Sylgard 184, DOW, mixed 10:1) layer was sandwiched between the chip and a blank borofloat glass slide aligned with the 24 sensing regions. Socket shoulder screws with conical spring washers were used to clamp and hold the sensor-PDMS-glass sandwich within a milled aluminum holder to give a liquid-tight seal. Chemically inert platinum-cured silicone tubing (MasterFlex 96410–13) was attached at the inlet and outlet. The system was first rinsed with 50 mL of ultrapure DI water. For the mineral water, vodka, and gin samples, this single rinse was shown to clean the sensor. Whiskey samples required two additional postsample rinses: 50 mL of pure ethanol followed by 50 mL of DI water. Due to the high protein and sugar content of wine and beer, a surfactant-based cleaning was necessary to completely clean the sensor. Five mL of 0.1 M sodium dodecyl sulfate (SDS) in DI water was shown to be effective at removing sample residues when allowed to “dwell” in the system for 5 min before being rinsed out with 50 mL of DI water. Wine required only one cycle of surfactant cleaning, whereas beer required two. When not in use, the chip was stored in DI water, and before the next round of measurements, the chip was flushed following the rinsing protocol specific to the liquid class last tested (at least 50 mL of DI water).

Multiple images of the light transmitted through the sensor array were collected for each liquid tested (white light from a 150 W tungsten halogen lamp was transmitted through the array onto a diffraction grating and then collected by a monochrome camera). These images were combined, integrated, and sectioned using an in-house MATLAB script to collect spectral information for each sensor in the array as a measure of transmission per wavelength (with a resolution of 0.1 nm). The measures were made over 5 min or longer for each liquid, with data collection every 30 s. Each liquid was independently repeated three times. The transmission minimum in the plasmonic region was extracted by spline fitting; the difference between the minimum for each liquid sample at a given time point was calculated as a shift in wavelength compared to the average of the preceding ten DI water measurements (Δλ). The different liquids were sampled over the course of multiple days, as described in the main text and Supporting Information.

### Statistical Analysis

These “difference” Δλ data were tabulated as a matrix of 1041 × 24 data points across the six categories of samples for each sensor region for each sample, at a given time *t* and repeat *n*. This table was used for all of the further statistical comparisons. All analyses and plotting were performed in JMP 18.0. PCA and HCA were carried out using standard package methods. PCA and HCA were additionally performed on the data after standardizing each row against the mean of the 24 Δλ values in that row. This was intended to remove the gross effect of RI change over all 24 sensor elements without changing the differential shifts between elements within each measurement. LDA was trained and tested on a 50:50 stratified split of the data table with stratification and sample identification against either the sample category (e.g., whiskey) or the individual sample name (e.g., whiskey 1). Random Forest classification was carried out in R (randomForest) on a separate detailed data set from three vodka samples collected across two independent instruments on two independent sensors over 3 weeks.

## Supplementary Material



## Data Availability

The raw data underlying the analysis is available at https://doi.org/10.5525/gla.researchdata.1949 and on request from the authors.

## Footnotes

1 A preprint of this article was posted on ChemRxiv: Sperling J, Osborne D, Aekbote B, Perri A, Setford R, Gao H, et al. An Integrated Plasmonic Sensing Array for Chemical Fingerprinting and Flavor Profiling in Beverages and Other Liquids. ChemRxiv. 2025; doi:10.26434/chemrxiv-2025-dd7tv (accessed 25th September 2025).

## Notes

Synthetic protocols and characterization for all molecules applied to the sensing array, full list of analytical samples, additional methods for array fabrication, additional data, experiments and analysis (PDF)
