# Supplementary material for: An Integrated Plasmonic Sensing Array for Chemical Fingerprinting and Flavor Profiling in Beverages and Other Liquids
Source: ACS Sens. 2025 Oct 3;10(10):7936–46. doi: 10.1021/acssensors.5c02485 (PMC12560130; doi:10.1021/acssensors.5c02485)
Supplement: Supplementary file 1 [file se5c02485_si_001.pdf]

# An Integrated Plasmonic Sensing Array for Chemical Fingerprinting and Flavour Profiling in Beverages and Other Liquids

Justin R Sperling,[1] Daniel D Osborne,[2] Badri Aekbote,[1] Anthony E Perri,[3] Rebecca A. Setford,[1] Hanyu Gao,[1] Liam T Wilson,[2] Chad Sipperley,[3] Rudolf J Schick,[3] Caroline Gauchotte-Lindsay,[1] William J Peveler,[2]* Alasdair W Clark[1]*

1. James Watt School of Engineering, Advanced Research Centre, University of Glasgow, Glasgow, G11 6EW, United Kingdom. E-mail: alasdair.clark@glasgow.ac.uk
2. School of Chemistry, Joseph Black Building, University of Glasgow, Glasgow, G12 8QQ, United Kingdom. E-mail: william.peveler@glasgow.ac.uk
3. Spray Analysis and Research Services, Spraying Systems Co., Wheaton, IL, 60139, USA

## Supplementary Information

## Table of Contents

Figure S1: Scanning electron microscopy of the Au nanostructure regions ............ 2
Figure S2: Transmission spectra of unmodified Au nanostructures ............ 3
Figure S3: Characterisation of surface assembled monolayers on Au nanostructures ............ 4
Figure S4: Image of the hyperspectral imaging system ............ 5
Figure S5: Additional performance testing against beverage samples ............ 6
Figure S6: Additional performance testing against coffee samples ............ 7
Figure S7: Additional performance data for multiple sensors and measurement devices on a common sample set ............ 9
Figure S8: Method for rejuvenating or replacing surface chemistries ............ 10
Figure S9: Individual plasmonic fingerprints for samples ............ 11
Figure S10: Additional Structure of Principal Components (Figure 3) ............ 12
Figure S11: Classification of water samples based on $Ca^{2+}$ content ............ 13
Figure S12: Analysis of kinetic changes of fingerprints for beer ............ 14
Figure S13: Principal Component Analysis of shift-standardised data ............ 16
Table S1: Table of surface modifications trialled ............ 18
Table S2: Table of sample identities and properties ............ 20
Table S3: Details of additional samples tested ............ 21
Table S4: Table of mineral water stated contents ............ 22
Supplementary Methods: Chemical synthesis and characterisation ............ 23
Supplementary References ............ 96

**Figure S1: Scanning electron microscopy of the Au nanostructure regions**

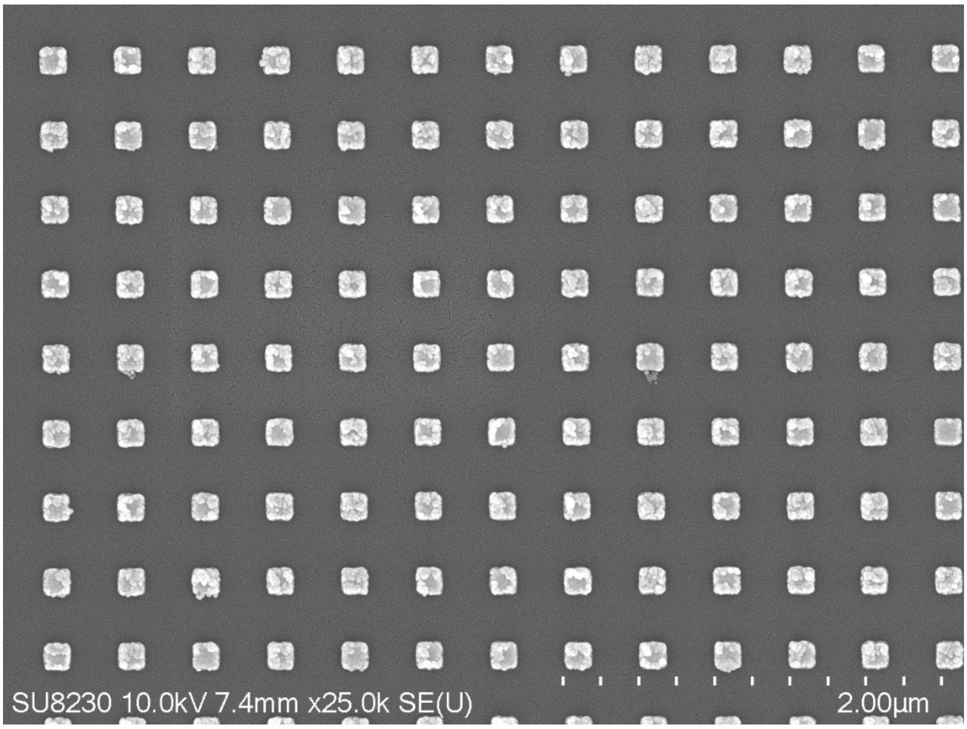


Representative SEM of the plasmonic nanostructures on one of the chip's sensors. Measured to be 146 ± 4 nm sided, 56 ± 4 nm tall squares, in an array with a period of *c.* 390 nm (N = 96).

The gold nanostructures that comprise each sensor on our chip comprising were characterised by SEM. **Figure S1** shows the micrograph used to calculate the size of the structures that is quoted in the main paper text. As all the 24 active sensors on the chip were fabricated using the same design, this SEM is representative of all the chip's nanoscale features.

**Figure S2: Transmission spectra of unmodified Au nanostructures**

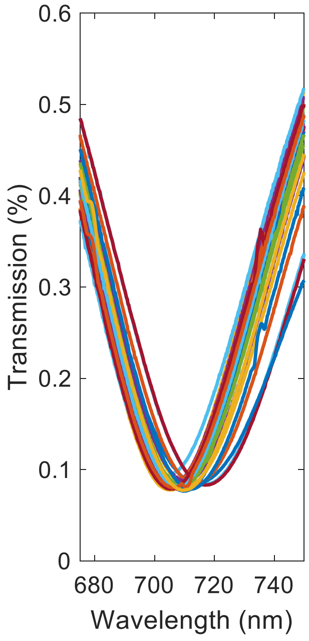


Although each sensor in the array has the same design parameters, small variations in the fabrication process mean that each sensor on the chip has a slightly different resonance property. This variance does not influence the performance of the chip, since all measurements are made as a shift from each sensor's resonance value in DI water. **Figure S2** shows the resonance values of all 24 sensors on the chip used for the majority of this work, measured as transmission minima in deionised water (RI = 1.333). Transmission measurements used to calculate the average resonance value quoted in the main text (709 ± 4 nm).

## **Figure S3: Characterisation of surface assembled monolayers on Au nanostructures**

Representative samples containing different chemical functionalities were prepared as for sensing and analysed by X-ray Photoelectron Spectroscopy (XPS), to demonstrate the presence of the self-assembled monolayer at the gold surface. The fill-factor of the Au nanostructures is less than 15 %, and thus the amount of SAM present is very small, signals were inherently weak and sat under the much larger Si environment from the borosilicate glass, so a full fitting and deconvolution was challenging. However, once the data is referenced to the Au 4f signals, S 2p and 2s signals were weakly visible for all samples. In addition, high-resolution C 2s data supported the presence of the target chemistries at the surface, showing carbonyl (C=O) environments where present for example. We have previously also demonstrated SAM formation using surface enhanced Raman spectroscopy (SERS, vide infra).[1]

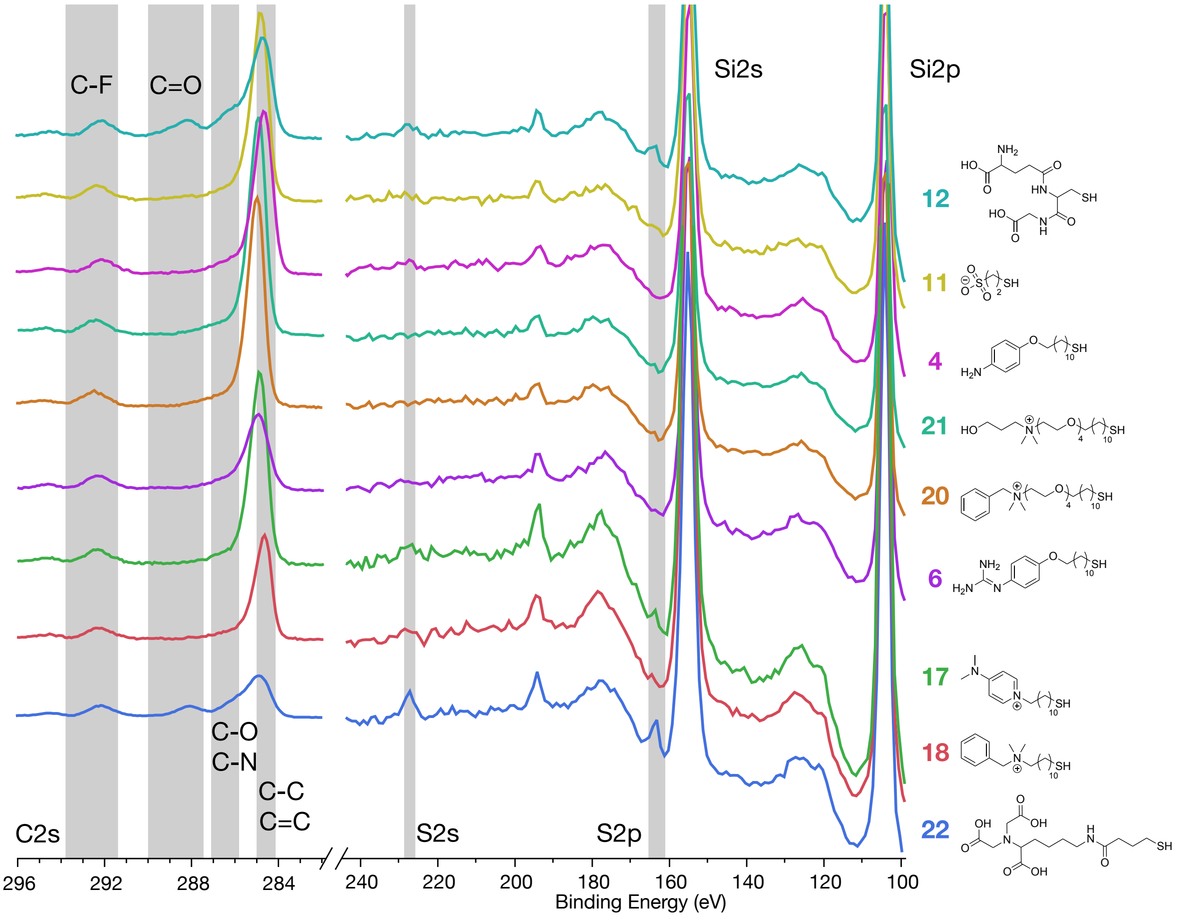

## Figure S4: Image of the hyperspectral imaging system

The hyperspectral imaging system is shown here, with the chip/holder installed, but without the tubing attached (seen in **Figure 1**). The system is laid out as per **Figure 2** and is around 40 cm long. Light power source and control computer not pictured but overall, the system is transportable and can be field deployed in a rigid case. The system is robust and has been deployed at industrial sites via car, public transport and air travel, maintaining its alignment and performance on arrival.

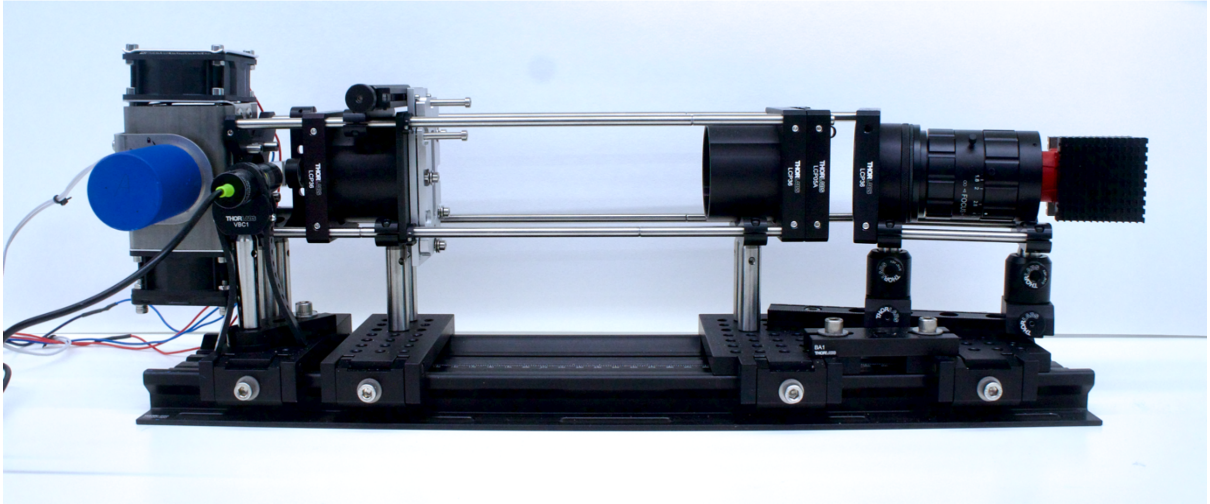

**Figure S5: Additional performance testing against beverage samples**

During the development of the final sensors shown used for the main experiments, earlier iterations of the sensor were tested with other beverage samples. This sensor comprised of 20 surface chemistries (in sequence **1**, **2**, **3**, **5**, **6**, **9**, **8**, **43**, **17**, **18**, **21**, **44**, **20**, **24**, **13**, **15**, **23**, **25**, **16** & **10**) and was tested against spirits, wines, beers and fruit juices. The complete sample list is given in **Table S3**. The outcomes of these tests are shown here as principal component analyses and discriminant analysis based on board sample class. Whilst alcohol and dissolved sugar content drove most discrimination on the first principal component (PC), with separations on PC 2 largely driven by the sensor coated with **24**.

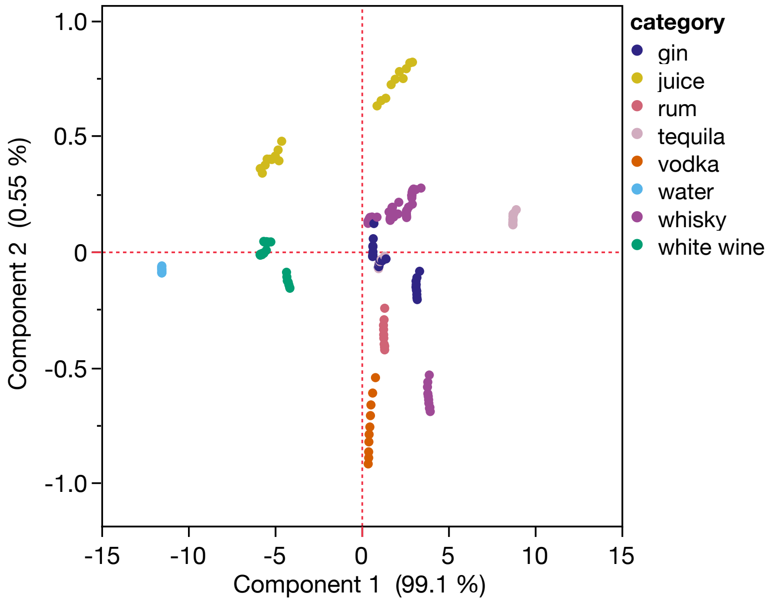


In the LDA, separation between the clusters (across the first two canonical axes) was largely driven by chemistries **18**, **20** and **6** amongst others. In all cases these chemistries were retained for future experiments but some others that showed high covariance or had small contributions were swapped for alternatives.

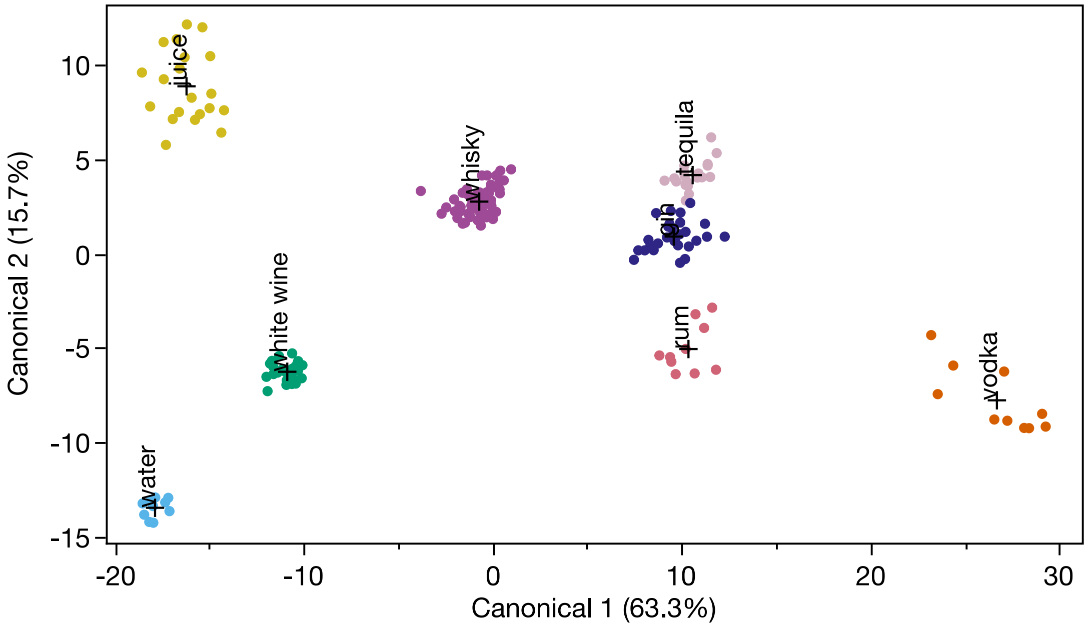

## Figure S6: Additional performance testing against coffee samples

The ability of the sensor to 'taste' coffees was explored as an even more challenging and complex chemical mixture. A series of instant 'espresso' style coffees and their longer versions were prepared according to manufacturer instructions on a commercially available Nespresso machine. The use of a machine and pod-style coffee like this enabled repeatable and comparable extraction across repeats and different available samples.

The espresso samples were prepared, allowed to cool to room temperature and then centrifuged at 10 °C for 5 minutes (RCF = 12,000 x *g*) to remove the majority of sediment, before a final finishing through a 0.45 µm PES filter before injecting over the sensor. Here the sample dwell time was 5 min or longer, and the washing step post sample used 1 M SDS. The sensor chemistries used for this analysis were (in sequence) **14**, **7**, **33**, **13**, **26**, **27**, **35**, **32**, **31**, **29**, **28**, **25**, **30**, **34**, **12**, **11**, **36**, **22**, **41**, **39**, **42**, **38** & **37**.

The coffees showed a distinct 'on-curve' over the initial 2 minutes, but plateaued by 5 minutes, with examples for one coffee shown below, so 5 minutes (measured every 30 seconds) was used for further analysis but the complete data across the 5 minutes was used in the final analysis.

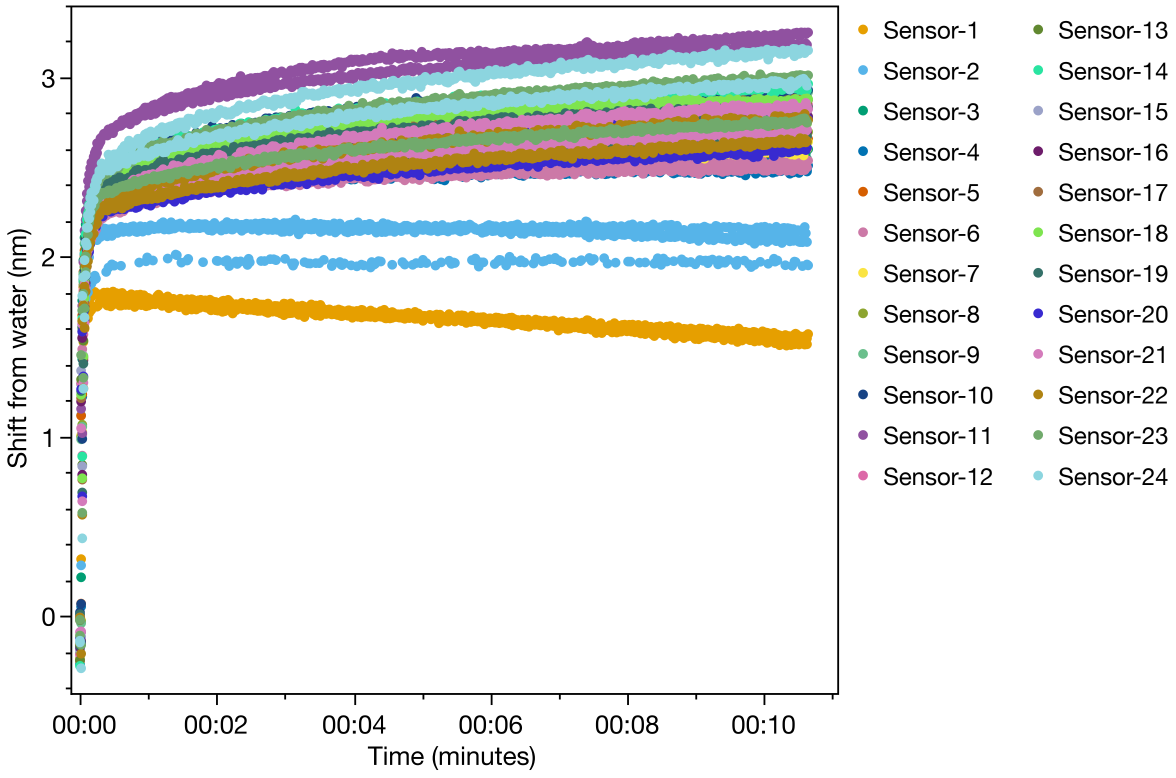


Nine coffees were analysed, comprising a range of origins, intensity scores (assigned by the manufacturer) and extraction styles (long and short). When analysed with multivariate methods, the major difference on the first PC, and the subgroupings observed by LDA were linked to the volume of extraction (short, concentrated coffees in open circles and more dilute, long coffees in solid colour circles), likely linked to dilution.

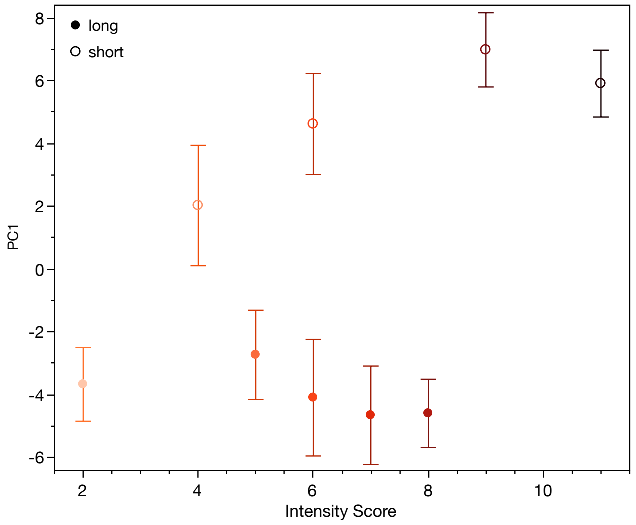


However, it was noted that the there was also a trend with the intensity score from the manufacturer (coloured from pale to dark and exemplified by plotting the average position of each coffee on the first PC). A similar trend was observed in LDA with the second canonical separating the different intensities.

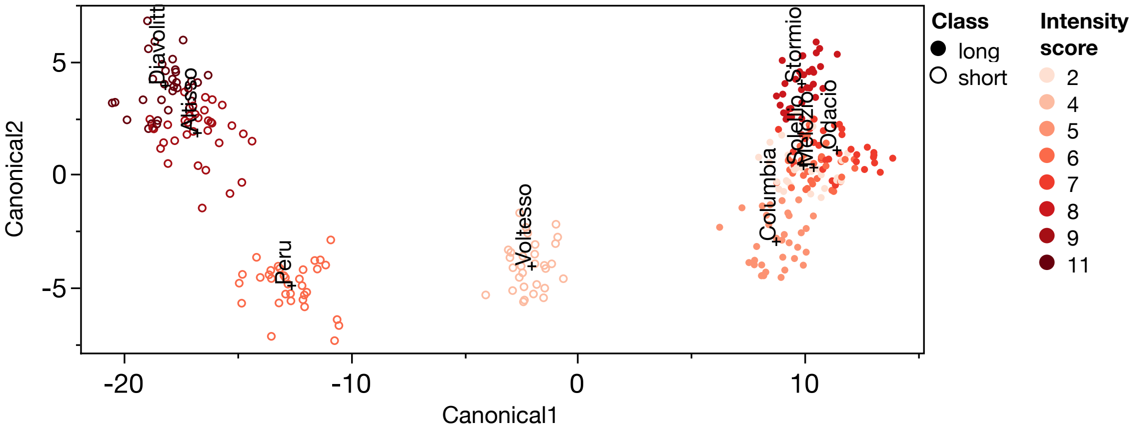


The chemical contribution to this separation on the sensing array is perhaps correlated with roast, with darker roasts developing more intense flavours as products of the Maillard reaction including furfuryl compounds. Notably roasted coffee contains 2-furanmethane thiol which might adsorb to the sensor surface, but this was not observed (there was a good return to baseline) when using short measurement time and a thorough washing regimen with surfactant to remove any build up.

## Figure S7: Additional performance data for multiple sensors and measurement devices on a common sample set

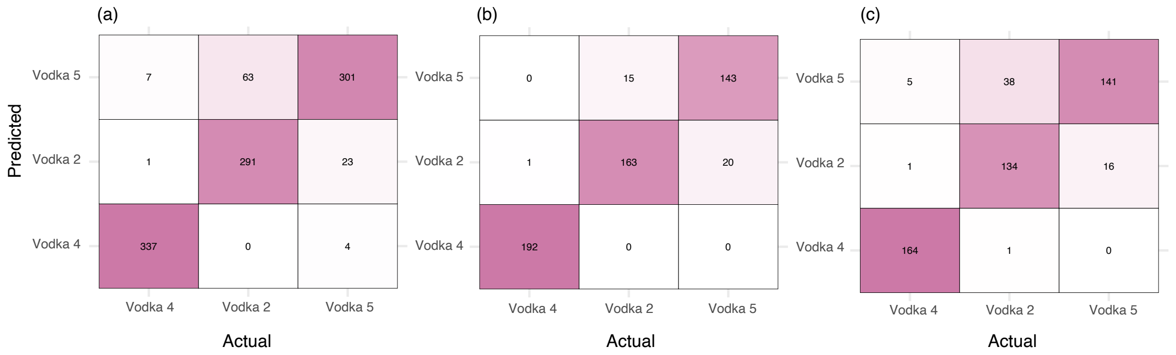


A sample of three vodkas was run on two independent prototype instruments, with two independent sensor arrays over the course of three weeks, with data collection on all samples on all days, by multiple instrument operators. Due to the scale of the data a Random Forest discriminator tool was trained on 50% of the data and tested on 50% of the data for (a) all the instruments and sensors, or (b) Prototype 1 and (c) Prototype 2. As shown in the confusion matrices above, Vodka 4 was well discriminated in all cases, with Vodka 2 and 5 appearing more similar to the independent sensors in both prototypes. The ability of the data to be successfully classified across multiple instruments, operators and sensor chips demonstrates the robustness of the system.

## Figure S8: Method for rejuvenating or replacing surface chemistries

The sensors shown in the main paper, and those used to generate the results shown in **Figure S5 and S6**, were simply washed between sample runs. However, it is inevitable that the sensors will not last indefinitely and at some point, would need to be replaced. With that in mind, we have developed a regeneration process for the chemical modifications on the sensor that allows the user to retain the original nanoplasmonic chip. To remove the chemical modifications we agitated the sensor in a 0.5M $NaBH_4$ (50:50 ethanol/water) solution for 10 minutes,[2] followed by a water and ethanol rinse and finally dried with a stream of $N_2$ gas. Sensors were then re-modified using the standard droplet printing technique (**Figure 1**). To demonstrate this process, we measured surface-enhanced Raman spectroscopy (SERS) signals from a sensor region modified with 4-nitrothiophenol (**34**, a well-known SERS probe). This sensor was "stripped" of its chemical modification and then re-modified, going through that cycle 4 times without any evident deterioration of the physical sensor itself (the nanostructures and hydrophobic droplet-pinning layer remain intact and performant). As can be seen from **Figure S7**, the SERS signals from the sensor are largely stable on each re-modification, albeit of slightly lower intensity compared the first modification. This provides a cost-efficient way to quickly rejuvenate sensors without having to dispose of the photonic chip itself.

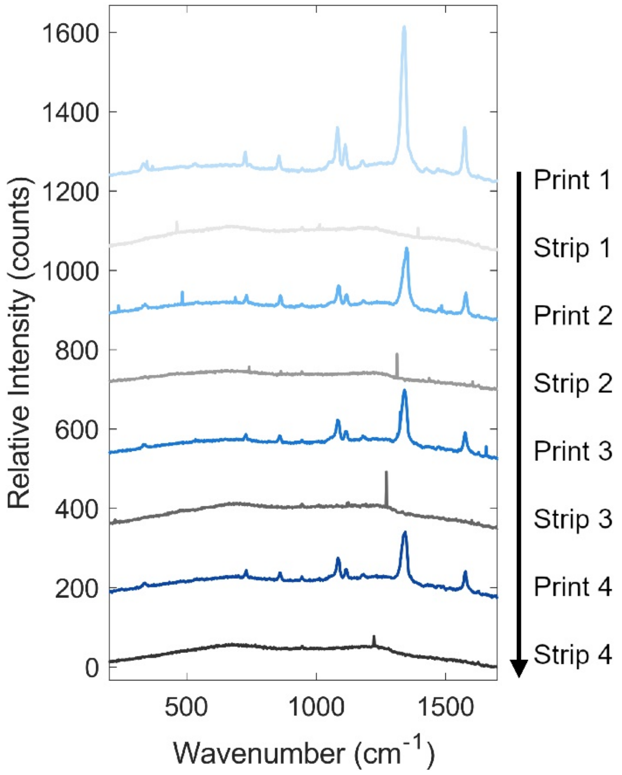

## Figure S9: Individual plasmonic fingerprints for samples

The unique 24-dimensional patterns (fingerprints) generated by the plasmonic chip for each of the liquids tested in the main paper are visualised here. Each value is calculated as a shift from the sensor's response in DI water and represented on a colour map. To emphasise the differences beyond the changes caused by differing ABV, three scales are used.

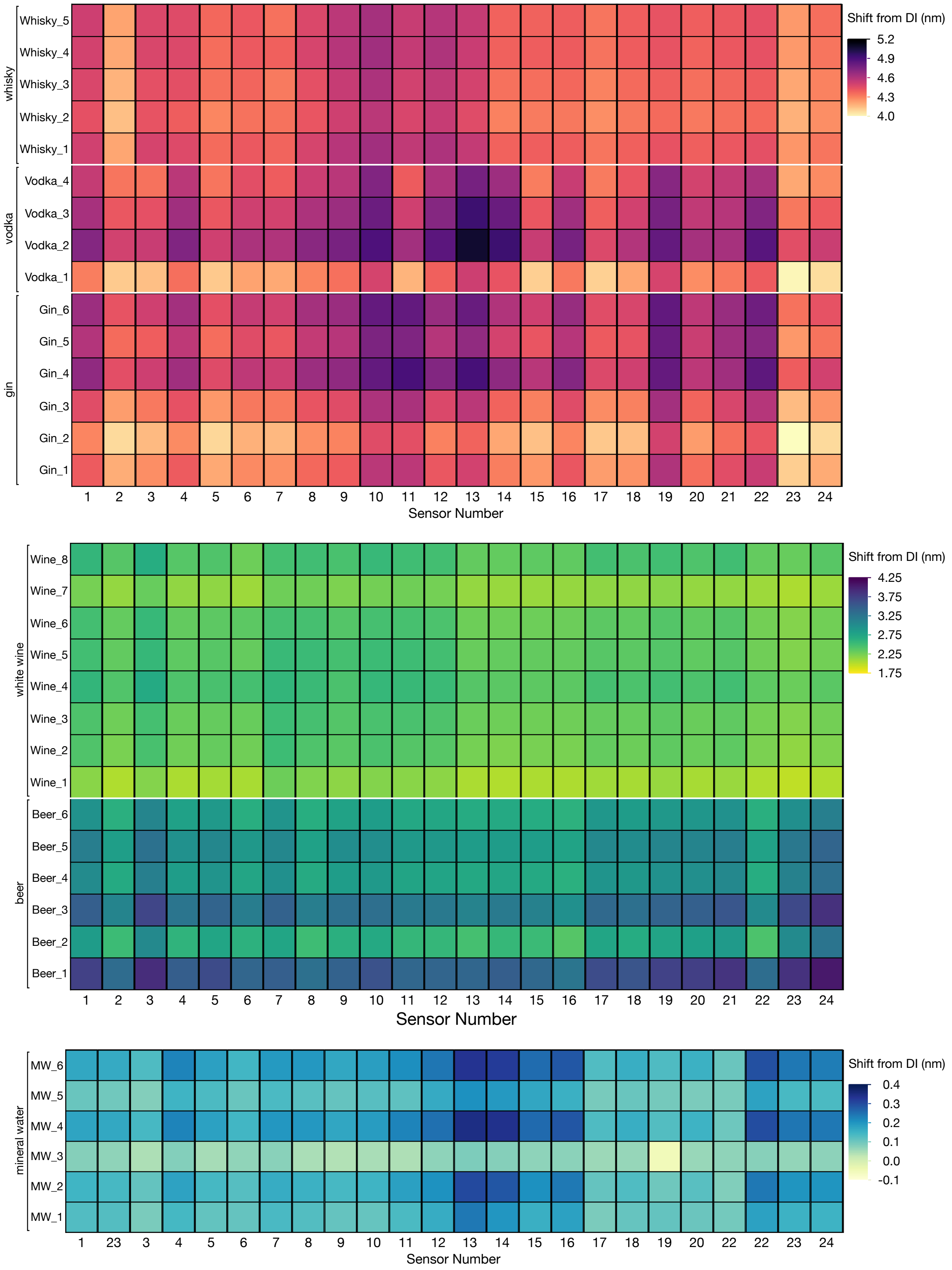

## Figure S10: Additional Structure of Principal Components (Figure 3)

A fuller PC structure of the data in **Figure 3b** is shown here. Although the first PC dominates (driven by overall refractive index change from alcohol content), the second PC shows discrimination of beer, and the third and beyond discriminate the spirits that are not separated on the first PC.

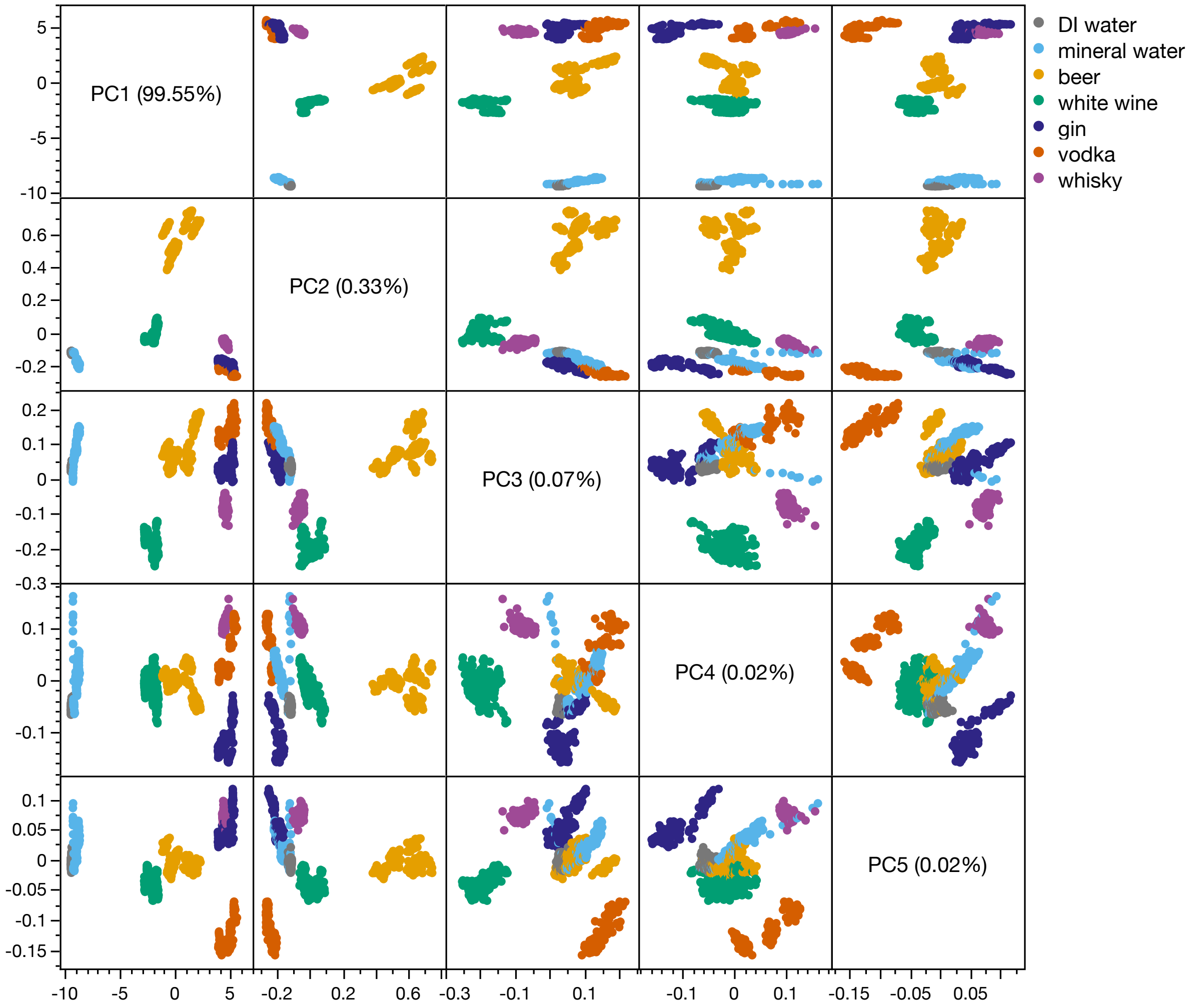


The impact of the RI shift is evidenced by the equal contribution of all sensors to the first PC, but there are significant impacts of individual or groups of sensors in later PCs, as shown below in the plot of partial contributions per sensor to the first 5 PCs.

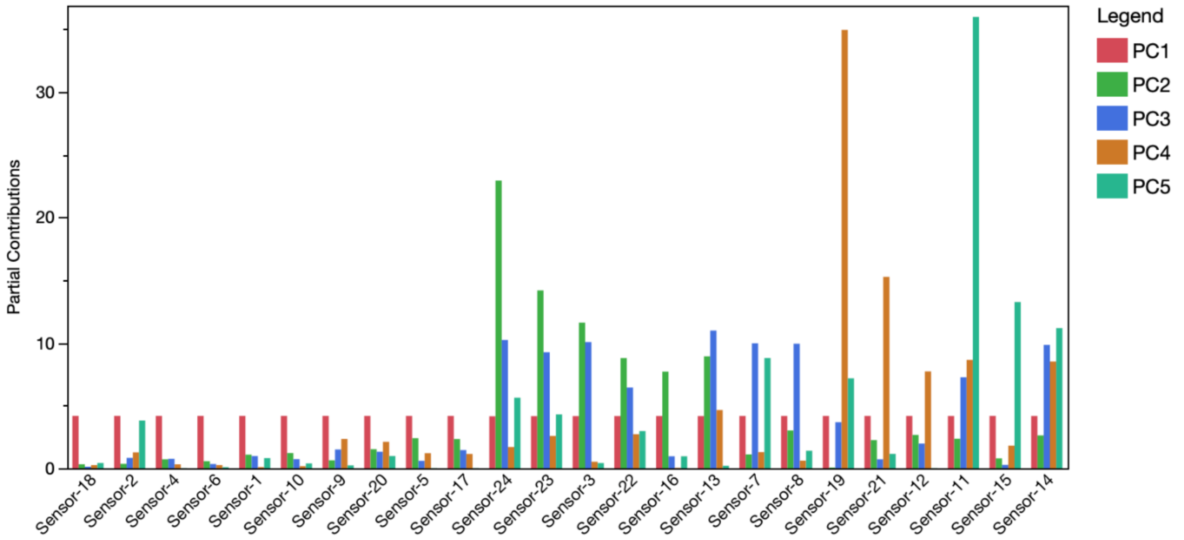

## Figure S11: Classification of water samples based on $Ca^{2+}$ content

As part of a screening and optimisation experiment to explore the sensor response to salts in water and water hardness, a set of fingerprints was collected from samples of DI water spiked with $CaCl_2$ ($Ca^{2+}$) at 0, 17 or 170 mg/L (0, 1 or 10 GPG or grains per gallon, a standard measure of water hardness) in the presence of varying levels of NaCl (0, 20 or 200 mg/L). The 24-part sensor comprised chemistries **14**, **7**, **33**, **13**, **40**, **27**, **35**, **32**, **31**, **29**, **28**, **25**, **30**, **34**, **12**, **24**, **11**, **36**, **22**, **23**, **39**, **42**, **38** & **37**. Discriminant analysis was undertaken. From this analysis, several significant chemistries were identified, as measured by their contribution to discrimination. These included glutathione (**12**) and the cyclodextrins (**23** and **24**), resulting in their inclusion in the final 24 for the main sensing chip.

These data demonstrate that mixtures and dilutions of samples, at low concentration, are well discriminated by the tool and technique. With further data collection and analysis such mixtures might be quantitatively analysed.[3]

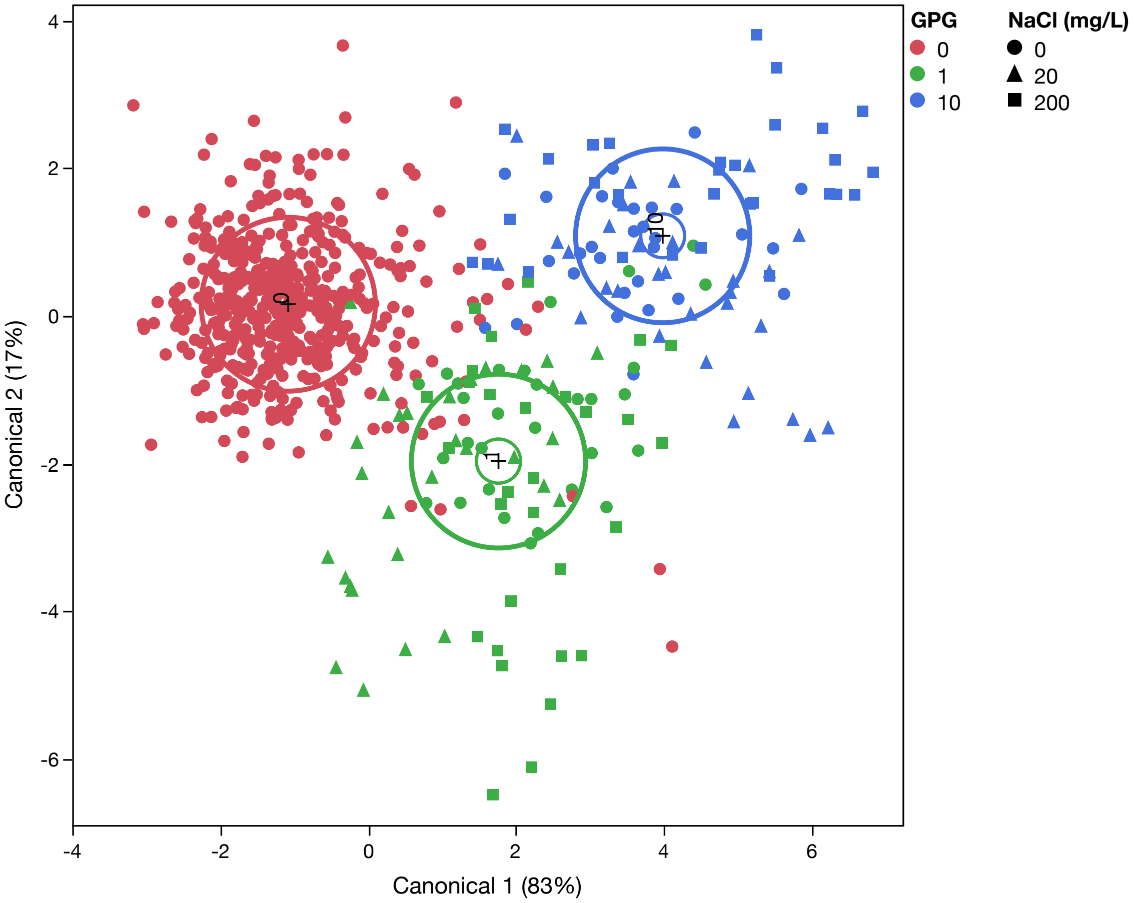

## Figure S12: Analysis of kinetic changes of fingerprints for beer

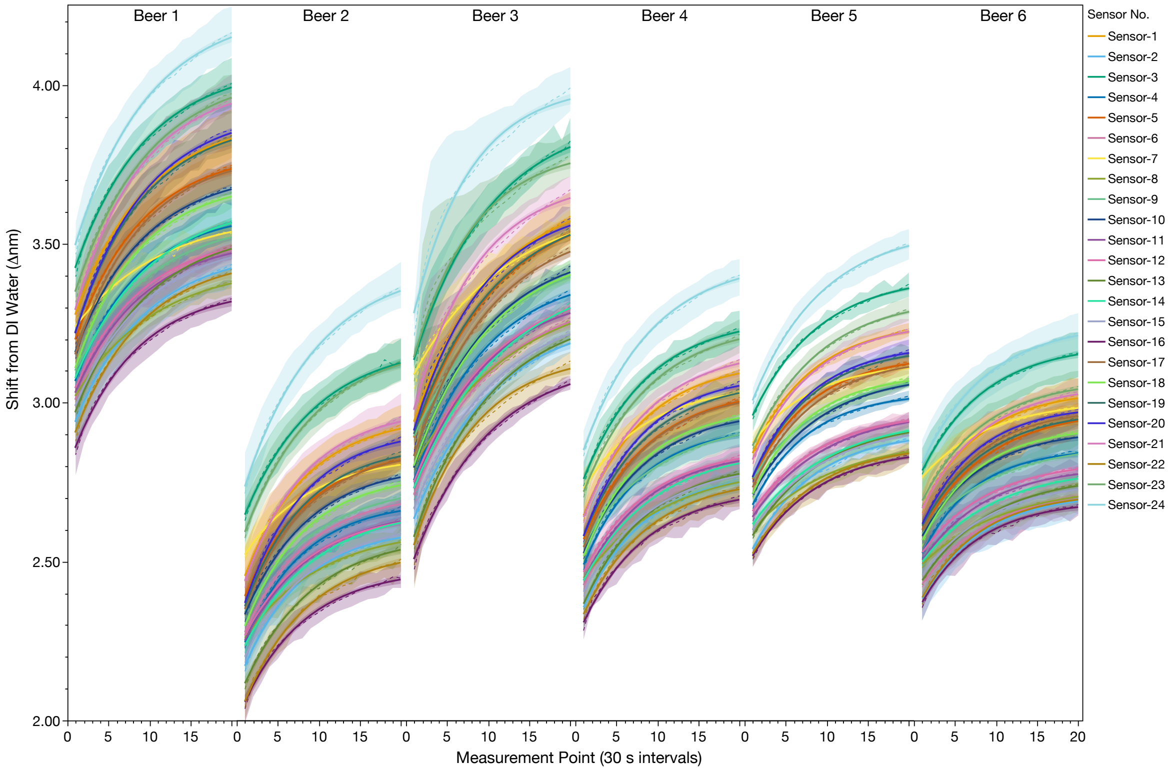


To demonstrate the importance of kinetics, beer samples were measured every 30 s over 10 minutes. Because a completely accurate 0 time could not be obtained (due to slight variance in the loading speed etc) the data are plotted against the measurement point (1-20) as a form of normalisation.

The mean of triplicate measures of shift versus DI water for each beer sample (listed in Table S2 and end point data used in **Figure 3** and **4**) are shown as dashed lines, and the range of the triplicate data is shown as a shaded area. These data are fitted well with a single exponential decay with an offset (overplotted as solid lines for the averaged data).

$$\text{shift} = \text{asymptote} + \text{scale} \cdot \exp(\text{growth rate} \cdot \text{time})$$

For this function, the scale, asymptote (final value reached) and growth rate (exponential factor) can be extracted for each fitted curve, per sensor, and used as additional inputs into a multidimensional model. The asymptote is closely related to the input used in the regular 'end-point' model, but the growth rate adds additional information that is gained form the kinetic analysis.

In these data it's notable that some beers that have higher asymptotes (end points) typically have more negative scale values and less negative growth rates averaged over all sensors and replicates. For example, Beers 1 and 3 have the highest asymptote and lowest scale value. However, despite Beers 4-6 having similar asymptote values, they show significantly differing scale values.

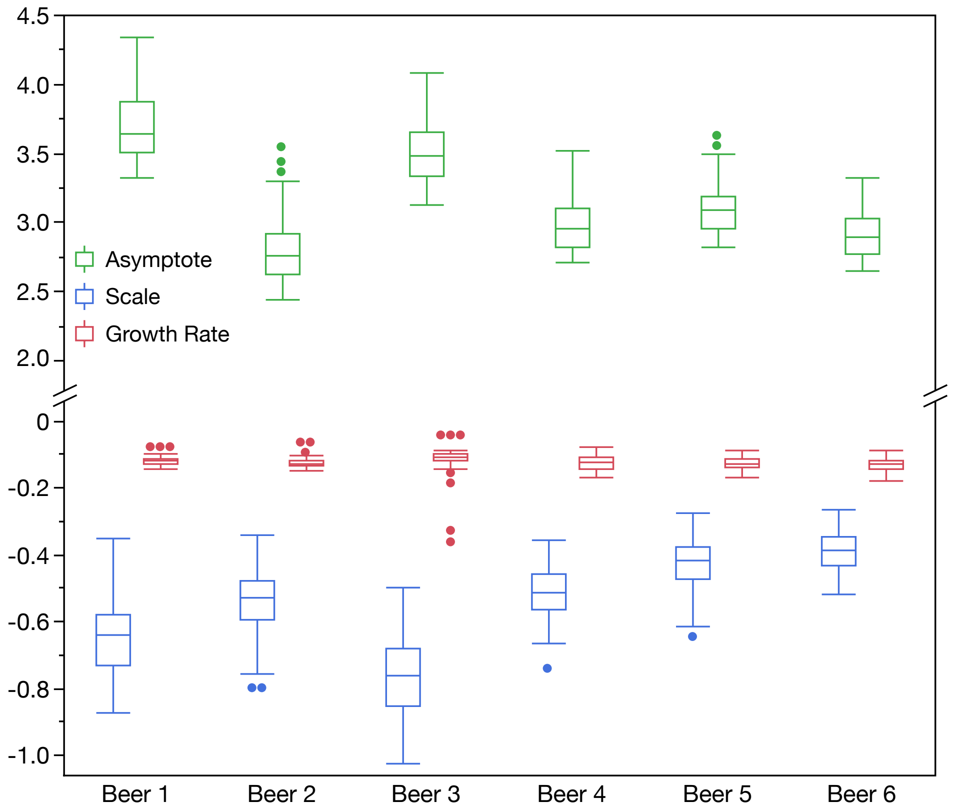


The differences become even more apparent when a per-sensor analysis is undertaken, where kinetic rates on certain sensors in the array are very different, all adding to the discrimination possible.

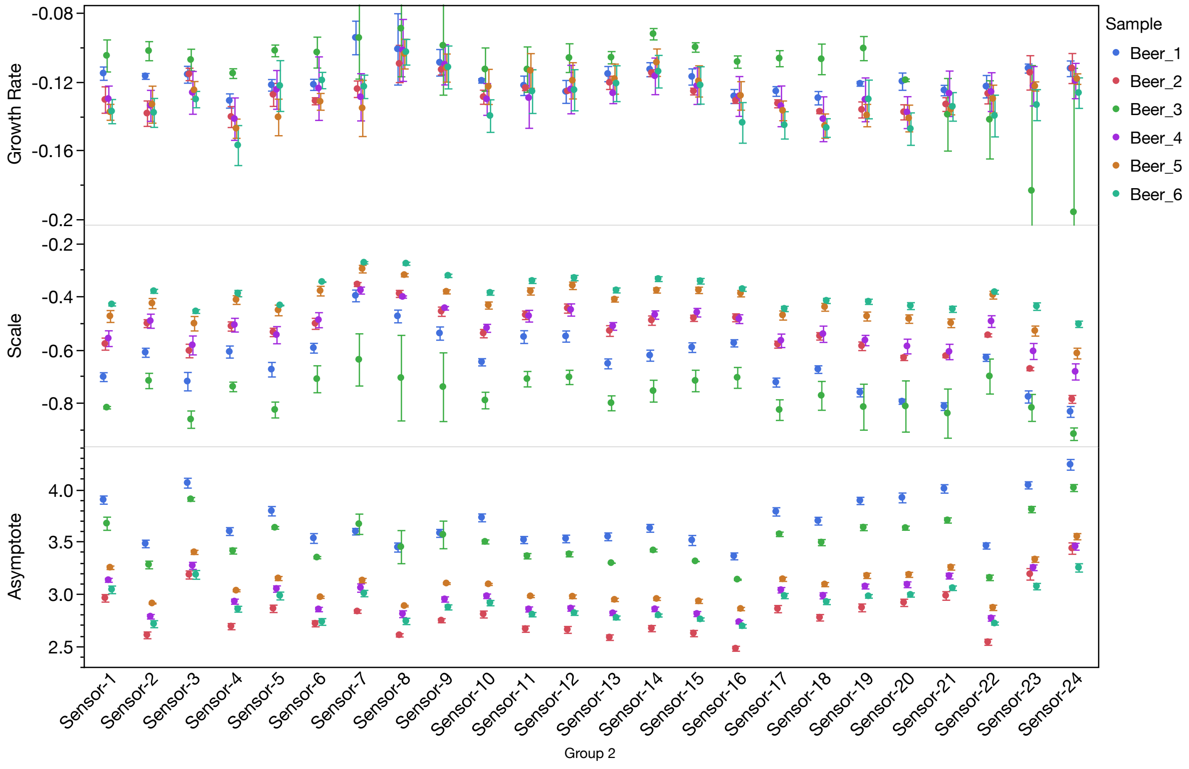

### Figure S13: Principal Component Analysis of shift-standardised data

After the mean of each measurement was removed from the fingerprint to account for the differing bulk refractive index induced shifts (see main text for details) PCA was re-run on data from sensors 1-23 (23 and 24 were highly colinear so 24 was removed to maintain the integrity of the PCA). The first 5 PCs shown below account for 94% of the variance and show natural clustering of the differing sample types. It is notable that PC1 now only accounts for 54.6% of the variance (rather than over 99% when using the raw data), and whilst vodka and gin overlap on the first 2 PCs, they are well separated on the 3[rd] and 4[th].

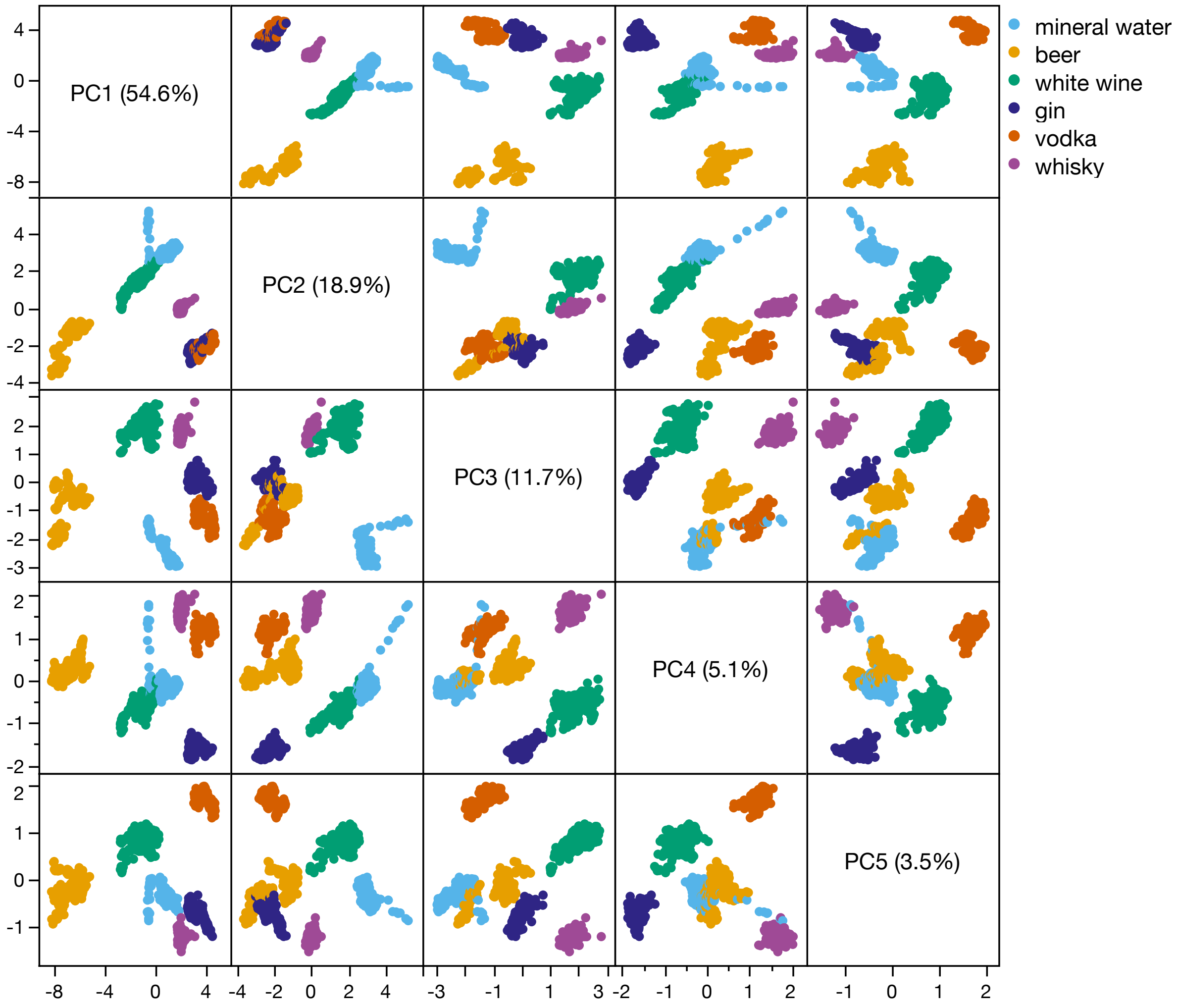


Plots of the relative contributions of the sensors to the first few PCs are also shown here. The loading plot for the first two PCs shows that all sensors contribute to the first two PCs, with some featured across both (diagonals e.g. 1, 6, 20 and 21) and others a primarily contributing to the first PC (left-right e.g. 3, 13, 22, 23 separating out beer from wine from spirits) or second PC (up-down e.g. 2, 15 and 19 separating out water from wines from beers etc.).

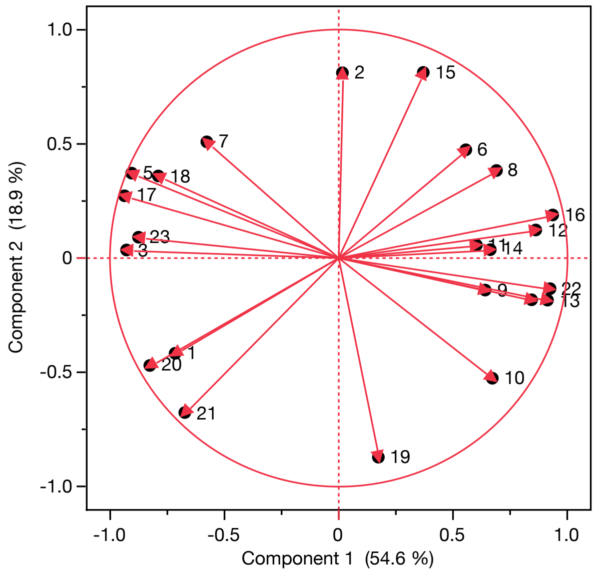


A plot of sensor partial contributions extends a more general analysis of contribution of each sensor to the first five PCs. This demonstrates the importance of all the sensors in the analysis as well as their cross-reactivity, with some sensors contributing across the first few PCs, and others contributing quite specifically to one particular PC.

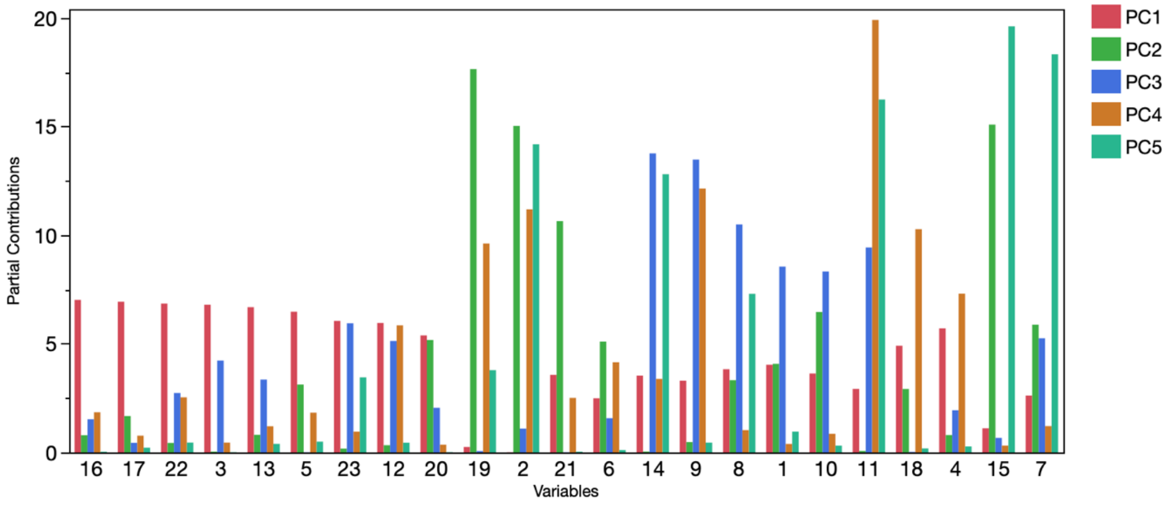

## Table S1: Table of surface modifications trialled

The 24 chemical modifications used on the chip are listed (numbers 1-24) in **Table S1**, along with their carrier solvent and concentration. These mixtures were drop printed onto the chip as described in the main text and shown in **Figure 1(e)**. Also shown are 20 additional surface modifications that have been trialled with the chip in the course of this work, including variants of the chip that generated the results shown in **Figures S5-7**. For each SAM the printing solvent and concentration used are given.

| No. | Abbr. | Thiol Name | Solvent | Conc. (mM) |
|---|---|---|---|---|
| 1 | hexyl-diMe(N+)-alk$_{11}$-thiol | *N*-hexyl-11-mercapto-*N*,*N*-dimethylundecan-1-aminium chloride | 80:20 EtOH:EG | 20 |
| 2 | decyl-diMe(N+)-alk$_{11}$-thiol | *N*-decyl-11-mercapto-*N*,*N*-dimethylundecan-1-aminium chloride | 80:20 EtOH:EG | 20 |
| 3 | hexadecyl-diMe(N+)-alk$_{11}$-thiol | *N*-(11-mercaptoundecyl)-*N*,*N*-dimethylhexadecan-1-aminium chloride | 80:20 EtOH:EG | 20 |
| 4 | para-nitro-phenoxy-alk$_{11}$-thiol | 11-(4-Nitrophenoxy)undecane-1-thiol | 80:20 EtOH:EG | 20 |
| 5 | para-amino-phenoxy-alk$_{11}$-thiol | 4-((11 Mercaptoundecyl)oxy)benzenaminium chloride | 80:20 EtOH:EG | 20 |
| 6 | guanidine-phenoxy-alk$_{11}$-thiol | N-(diaminomethylene)-4-((11-mercaptoundecyl)oxy)benzenaminium chloride | 80:20 EtOH:EG | 10 |
| 7 | DDT | Dodecanethiol | 80:20 EtOH:EG | 20 |
| 8 | HO-alk$_{11}$-thiol | 11-Mercaptoundecan-1-ol | 80:20 EtOH:EG | 20 |
| 9 | HO-PEG4-alk$_{11}$-thiol | 23-Mercapto-3,6,9,12-tetraoxatricosan-1-ol | 80:20 EtOH:EG | 20 |
| 10 | HO-PEG$_{MW750}$-Lipoic Acid | *O*-(5-(1,2-Dithiolan-3-yl)pentanoate)polyethylene glycol | DMSO | 10 |
| 11 | MES | 2-Mercaptoethanesulfonate | water | 20 |
| 12 | GLU | *L*-glutathione | water | 20 |
| 13 | MPA | 3-Mercaptopropionic acid | 80:20 EtOH:EG | 20 |
| 14 | MUA | 1-Mercaptoundecanoic acid | 80:20 EtOH:EG | 20 |
| 15 | COOH-PEG$_4$-alk$_{11}$-thiol | 26-Mercapto-3,6,9,12,15-pentaoxahexacosanoic acid | 80:20 EtOH:EG | 20 |
| 16 | COOMe-PEG$_4$-alk$_{11}$-thiol | Methyl 26-mercapto-3,6,9,12,15-pentaoxahexacosanoate | 80:20 EtOH:EG | 20 |
| 17 | DMAP-alk$_{11}$-thiol | 4-(Dimethylamino)-1-(11-mercaptoundecyl)pyridin-1-ium chloride | 80:20 EtOH:EG | 20 |
| 18 | diMeBenzyl(N+)-alk$_{11}$-thiol | *N*-benzyl-11-mercapto-*N*,*N*-dimethylundecan-1-aminium chloride | 80:20 EtOH:EG | 20 |
| 19 | triMe(N+)-PEG$_4$-alk$_{11}$-thiol | 23-Mercapto-*N*,*N*,*N*-trimethyl-3,6,9,12-tetraoxatricosan-1-aminium chloride | 80:20 EtOH:EG | 20 |
| 20 | diMeBenzyl(N+)-PEG$_4$-alk$_{11}$-thiol | *N*-benzyl-23-mercapto-*N*,*N*-dimethyl-3,6,9,12-tetraoxatricosan-1-aminium methanesulfonate | 80:20 EtOH:EG | 20 |
| 21 | propanol-DiMe(N+)-PEG$_4$-alk$_{11}$-thiol | *N*-(3-hydroxypropyl)-23-mercapto-*N*,*N*-dimethyl-3,6,9,12-tetraoxatricosan-1-aminium chloride | 80:20 EtOH:EG | 20 |
| 22 | NTA-thiol | 2,2'-((1-Carboxy-5-(4-mercaptobutanamido)pentyl)azanediyl)diacetic acid | water | 20 |
| 23 | β-CD | Perthiolated *beta*-cyclodextrin | DMSO | 1 |
| 24 | γ-CD | Perthiolated *gamma*-cyclodextrin | DMSO | 1 |
| 25 | OT | 1-Octanethiol | 80:20 EtOH:EG | 20 |
| 26 | PET | 2-Phenyletanethiol | 80:20 EtOH:EG | 20 |

| | | | | |
|---|---|---|---|---|
| 27 | MBA | 4-Mercaptobenzoic acid | 80:20 EtOH:EG | 20 |
| 28 | MHOH | 6-Mercapto-1-hexanol | 80:20 EtOH:EG | 20 |
| 29 | FMT | 2-furanmethanethiol | 80:20 EtOH:EG | 20 |
| 30 | AMT | 3-amino-5-mercapto-1,2,4-triazole | 80:20 EtOH:EG | 5 |
| 31 | MPBA | 4-mercaptophenylboronic acid | 80:20 EtOH:EG | 20 |
| 32 | PFDT | *1H,1H,2H,2H*-perfluorodecanethiol | 80:20 EtOH:EG | 20 |
| 33 | NBA | 5,5'-dithiobis(2-nitrobenzoic acid) | 80:20 EtOH:EG | 20 |
| 34 | NTP | 4-nitrothiophenol | 80:20 EtOH:EG | 20 |
| 35 | DTP | 3,4-dichlorothiophenol | 80:20 EtOH:EG | 20 |
| 36 | CA | cysteamine (2-aminoethanethiol) | 80:20 EtOH:EG | 20 |
| 37 | AATP | 4-acetamidothiophenol | 80:20 EtOH:EG | 20 |
| 38 | NTT | 2-naphthalenethiol | 80:20 EtOH:EG | 5 |
| 39 | ATP | 4-aminothiophenol | 80:20 EtOH:EG | 20 |
| 40 | FTP | 4-fluorothiophenol | 80:20 EtOH:EG | 20 |
| 41 | ODT | 1-octadecanethiol | 80:20 EtOH:EG | 20 |
| 42 | LC | *L*-cysteine | 80:20 EtOH:EG | 20 |
| 43 | PFBM | perfluorobenzyl mercaptan | 80:20 EtOH:EG | 20 |
| 44 | triMe(N+)-alk$_{11}$-thiol | 11-mercapto-N,N,N-trimethylundecan-1-aminium chloride | 80:20 EtOH:EG | 20 |

**Table S2: Table of sample identities and properties**

A full list of the individual samples used in **Figures 2-4**. All samples were store-bought, and samples such as beer and wine that might rapidly degrade once opened, were used as soon as possible after purchase and opening.

| ID | Name | ABV | RI | Notes |
|---|---|---|---|---|
| MW 1 | Filtered Tap Water | 0 | 1.333 | |
| MW 2 | M&S Still Scottish Mountain Water | 0 | 1.333 | |
| MW 3 | VOSS Mineral Water | 0 | 1.333 | |
| MW 4 | Highland Spring Still Spring Water | 0 | 1.333 | |
| MW 5 | Smart Water | 0 | 1.333 | |
| MW 6 | Evian Natural Mineral Water | 0 | 1.333 | |
| Vodka 1 | Smirnoff | 37.5 | 1.353 | |
| Vodka 2 | Black Cow | 40 | 1.3545 | |
| Vodka 3 | Finlandia | 40 | 1.35425 | |
| Vodka 4 | Absolut | 40 | 1.35425 | |
| Gin 1 | Isle of Islay Nesabus Gorse | 40 | 1.354 | |
| Gin 2 | Gordons | 37.5 | 1.3535 | |
| Gin 3 | Nordes | 40 | 1.354 | |
| Gin 4 | Opihr | 40 | 1.3545 | |
| Gin 5 | Beefeater | 40 | 1.3545 | |
| Gin 6 | Hendrick's | 41.4 | 1.355 | |
| Whisky 1 | Bells Blended Whisky | 40 | 1.3545 | |
| Whisky 2 | Famous Grouse Blended Whisky | 40 | 1.3545 | |
| Whisky 3 | Glenmorangie 10y | 40 | 1.354 | |
| Whisky 4 | High Commissioner Blended Whisky | 40 | 1.3545 | |
| Whisky 5 | Glenfiddich 12y | 40 | 1.3545 | |
| Beer 1 | Heineken | 5 | 1.34025 | |
| Beer 2 | Amstel | 4.1 | 1.3375 | |
| Beer 3 | Tennent's | 4 | 1.339 | |
| Beer 4 | Fosters | 4 | 1.339 | |
| Beer 5 | Red Stripe | 4.7 | 1.340 | |
| Beer 6 | Peroni | 5 | 1.34025 | |
| Wine 1 | Acacia Road 2023 Sauvignon Blanc | 11 | 1.3415 | Sauvignon Blanc, South Africa |
| Wine 2 | Pringle Bay 2021 Sauvignon Blanc | 12.5 | 1.342 | Sauvignon Blanc, South Africa |
| Wine 3 | Mud House 2022 Sauvignon Blanc | 12.5 | 1.342 | Sauvignon Blanc, New Zealand |
| Wine 4 | Villa Maria 2023 Sauvignon Blanc | 12.5 | 1.342 | Sauvignon Blanc, New Zealand |
| Wine 5 | Bourgogne 2022 Chardonnay | 13 | 1.3425 | Chardonnay, France |
| Wine 6 | Louis Jadot Les Roches Blanches 2022 Macon-Villages | 12.5 | 1.342 | Chardonnay, France |
| Wine 7 | Adelaide Hills Kangarilla Road 2021 Chardonnay | 13 | 1.3415 | Chardonnay, Australia |
| Wine 8 | Margaret River Vasse Felix 2023 Chardonnay | 12.5 | 1.342 | Chardonnay, Australia |

## Table S3: Details of additional samples tested

**Table S3** details the beverage samples used in **Figures S5, S6 and S7**. All samples were store-bought and samples such as beer and wine that are known to rapidly degrade once opened were used as soon as possible after purchase and opening. Coffee samples were brewed freshly according to manufacturer instructions (on an appropriate automatic machine) and were centrifuged and filtered as described above before measurement to remove sediments.

| ID | Name | ABV | RI |
|---|---|---|---|
| gin_1 | Isle of Islay Nesabus Gorse Gin | 40 | 1.353 |
| gin_2 | Gordons | 37.5 | 1.353 |
| gin_3 | Harris Gin | 45 | 1.357 |
| juice_1 | Sainsbury's Cloudy Apple Juice | 0 | 1.349 |
| juice_2 | Sainsbury's Cranberry Juice | 0 | 1.335 |
| rum_1 | Bacardi Carta Blanca | 37.5 | 1.353 |
| tapWater_1 | Filtered Tap Water | 0 | 1.333 |
| tequila_1 | Olmeca Altos Blanco | 38 | 1.353 |
| tequila_2 | 1800 Coconut | 35 | 1.365 |
| vodka_1 | Smirnoff | 37.5 | 1.353 |
| Vodka 5 | Titos | 40 | 1.3545 |
| whisky_1 | Tamnavulin Red Wine Cask | 40 | 1.354 |
| whisky_2 | Glen Garioch Founders | 48 | 1.358 |
| whisky_3 | Glenmorangie 10y | 40 | 1.354 |
| whisky_4 | Glendronach 12y | 43 | 1.355 |
| whisky_5 | Glenfiddich 18y | 40 | 1.354 |
| whisky_6 | Glenfiddich 12y | 40 | 1.354 |
| wWine_1 | Cape Kyala Chenin Blanc | 11 | 1.341 |
| wWine_2 | Terre di Chiete IGT | 11 | 1.341 |
| wWine_3 | Casillero del Diablo Sauvignon Blanc | 13 | 1.342 |

| ID | Name | Type | Intensity |
|---|---|---|---|
| C01 | Melozio | long pour | 6 |
| C02 | Altissio | espresso | 9 |
| C03 | Solellio | long pour | 2 |
| C04 | Diavolitto | espresso | 11 |
| C05 | Odacio | long pour | 7 |
| C06 | Peru | espresso | 6 |
| C07 | Stormio | long pour | 8 |
| C08 | Voltesso | espresso | 4 |
| C09 | Columbia | long pour | 5 |

### Table S4: Table of mineral water stated contents

The reported ion and total dissolved solids (TDS) for each sample was collated where available from the manufacturer or online analyses. It is notable that MW3 had the lowest reported mineral content and sits closest to DI water in our analysis (**Figure 4**). MW1 and MW5 (reportedly DI water with minerals added back) are next closest, and then the three natural mineral/spring waters with higher mineral content are furthest from DI. To discriminate these waters the key chemistries in the analysis have COOH groups or will otherwise bind positive ions such as $Ca^{2+}$: **12**, **15**, **22**, **23**, **13** & **14**.

| Sample | Name | $Ca^{2+}$ | $Mg^{2+}$ | $Na^{+}$ | $K^{+}$ | $HCO_3^{-}$ | $SO_4^{2-}$ | $Cl^{-}$ | $NO_3^{-}$ | TDS | pH |
|---|---|---|---|---|---|---|---|---|---|---|---|
| MW1 | Filtered tap* | | | | | | | | | | |
| MW2 | M&S Still Scottish Mountain Water | 42 | 9 | 12 | 1 | 135 | 13 | 10 | 8 | * | 7.4 |
| MW3 | VOSS Mineral Water | 3 | <1 | 4 | - | - | - | 5 | - | 40 | 5.9 |
| MW4 | Highland Spring Still Spring Water | 40.5 | 10.1 | 5.6 | 0.7 | 150 | 5.3 | 6.1 | 3.1 | 170 | 7.8 |
| MW5 | Smart Water* | 10-17* | > 0* | - | > 0* | > 0* | - | > 0* | - | ~20 | 6.5 |
| MW6 | Evian Natural Mineral Water | 80 | 26 | 6.5 | 1 | 360 | 14 | 10 | 3.8 | 345 | 7.2 |

Values are given in mg/L and an * indicates unknown or not officially reported value, but some indicative numbers or suggestion of presence can be found *via* online searches.

## Supplementary Methods: Chemical synthesis and characterisation

### Standard Procedures

Unless otherwise stated, reagents were purchased from commercial suppliers (Merck Life Sciences, Fisher Scientific Tokyo Chemical Industry or Santa Cruz Biotechnology) and used without additional purification. The water used was deionised (>15 MΩ, unless stated otherwise) and the non-aqueous solvents used were of analytical grade, purchased from the suppliers stated above. Anhydrous solvents (THF, $Et_2O$, Toluene and DCM) were obtained by passage through Innovative Technologies Pure Solv solvent filtration systems and solvents transferred by syringe or cannula to maintain the anhydrous environment. Reactions were conducted within round bottom flasks or sealed microwave vials using Radleys Pro hotplates with DrySyn adaptors and controlled using a Radleys temperature probe. All glassware was dried with a heat gun or stored in the oven prior to use. Reactions performed at room temperature (rt) were run at approximately 20 °C under standard atmospheric conditions unless otherwise stated. Reactions requiring an inert atmosphere were performed using nitrogen and the use of Schlenk techniques.

Thin Layer Chromatography (TLC) was performed as a reaction monitoring tool and as a determination of solvent systems for column chromatography using self-cut Supelco silica gel coated aluminium plates (0.2 mm particle size, 60 Å pore-size) impregnated with a fluorescent indicator (254 nm). Plates were visualised using ultraviolet (UV) light ($\lambda_{Max}$ = 254 nm or 365 nm) or through staining (Iodine, Vanillin or $KMnO_4$). Solvents and other volatile compounds were removed using a Buchi Rotary Evaporator or a Christ Alpha 2-4 LO plus freeze-dryer. Flash chromatography was performed manually using self-packed columns (d = 40 mm or 70 mm) filled with silica gel (60 Å, 40-63 u). Solvents used were of reagent grade and were used as received from suppliers unless otherwise stated. Preparative high-performance liquid chromatography (HPLC) was performed using a Dionex HPLC system equipped with Dionex P680 pumps and a Dionex UVD170U UV-Vis detector (monitoring at 214 nm and 280 nm), using a Phenomenex, Gemini C18- column (5 $\mu$m particle size, 250 x 21.2 mm). Gradients were performed using a solvent system consisting of A ($H_2O$ + 0.1% TFA) and B (MeCN + 0.1% TFA). Desired fractions were lyophilised using a Christ Alpha 2-4 LO plus freeze dryer. Centrifugation was performed with a Hareus Megafuge 8R in 50 mL falcon tubes at 9500 rpm or less (12,108 × g or less) or in 1.5 mL Eppendorf tubes at 14000 rpm or less (21694 × g or less). Analytical HP-LC was performed on a Dionex Ultimate 3000 LC fitted with a Reprosil Gold C18- column (3 µm particle size, 150 x 4mm) using a solvent system consisting of A ($H_2O$ + 0.1% TFA) and B (MeCN + 0.1% TFA).

Nuclear Magnetic Resonance (NMR) Spectrometry – Proton ($^1H$) and Carbon ($^{13}C$) were recorded in deuterated solvents, unless otherwise stated, using standard pulse methods on an AVANCE III 400 Bruker ($^1H$ = 400 MHz, $^{13}C$ = 101 MHz). Chemical shifts are expressed in parts per million (ppm, δ scale) and are referenced to tetramethylsilane (TMS) or to residual protium in the deuterated solvent ($CDCl_3$ ($^1H$ = 7.26 ppm, $^{13}C$ = 77.0 ppm), MeOD ($^1H$ = 3.31 ppm, $^{13}C$ = 49.0 ppm), $D_2O$ ($^1H$ = 4.790 ppm) and DMSO ($^1H$ = 2.50 ppm, $^{13}C$ = 39.5 ppm)). Coupling constants, *J*, are quoted to the nearest 0.1 Hz and multiplicities are described as singlet (s), doublet (d), triplet (t), quartet (q), pentet (p), sextet (sxt), septet (sept), broad (br) and multiplet (m). Mass spectrometry data was collected by School of Chemistry Technicians on an Agilent 6546 LC/Q-TOF instrument under positive or negative electrospray ionisation.

**Synthesis of Quaternary ammonium thiol derivatives 1–3**

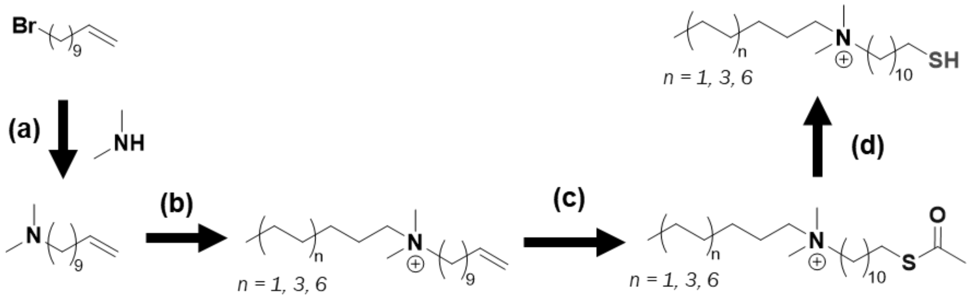


(a) EtOH, rt, 24 h, 85% (b) Br-R (R = *n*-hexyl 85%, *n*-decyl, 83%, *n*-hexadecyl, 81%), 80 °C, 48 h (> 90%) (c) Thioacetic acid, AIBN, anhydrous MeOH, $N_2$, 70 °C, (81%, 84%, 76%) (d) HCl /MeOH (1.25 M, > 10 eq.), 3h, 55 °C, quant.

N,N-dimethylundec-10-en-1-amine **S1**

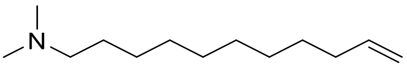


To a solution of 11-bromo-undecene (2.0 g, 8.58 mmol) in THF (10 mL), dimethyl amine (12.9 mL, 25.73 mmol, 2M in THF) was added and stirred for 24 hrs at RT. 1M NaOH (10 mL) was subsequently added to the reaction. The product was extracted with diethyl ether (3 × 40 mL), the combined organic layers were dried ($MgSO_4$), filtered through cotton wool and concentrated *in vacuo*. The product **S1** was obtained without further purification as a yellow oil (1.43 g, 85%). Observed analytical data matched that of the original work.[4] **$^1$H NMR** (400 MHz, $CDCl_3$) δ 5.88–5.74 (m, 1H), 5.04–4.88 (m, 2H), 2.27–2.22 (m, 2H), 2.21 (s, 6H), 2.09–1.98 (m, 2H), 1.50–1.21 (m, 14H). **$^{13}$C NMR** (101 MHz, $CDCl_3$) δ 139.0, 114.0, 59.9, 45.5, 33.7, 29.6, 29.5, 29.4, 29.1, 28.9, 27.8, 27.4.

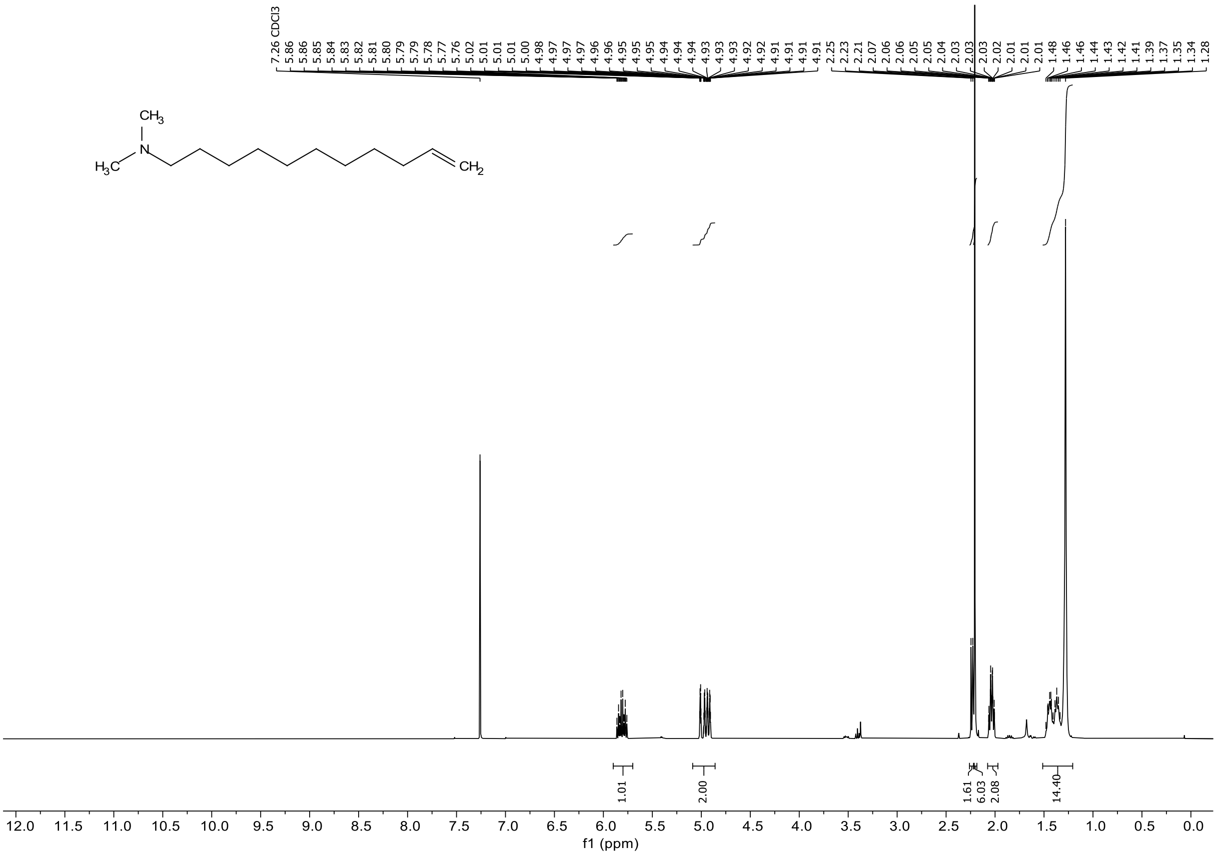

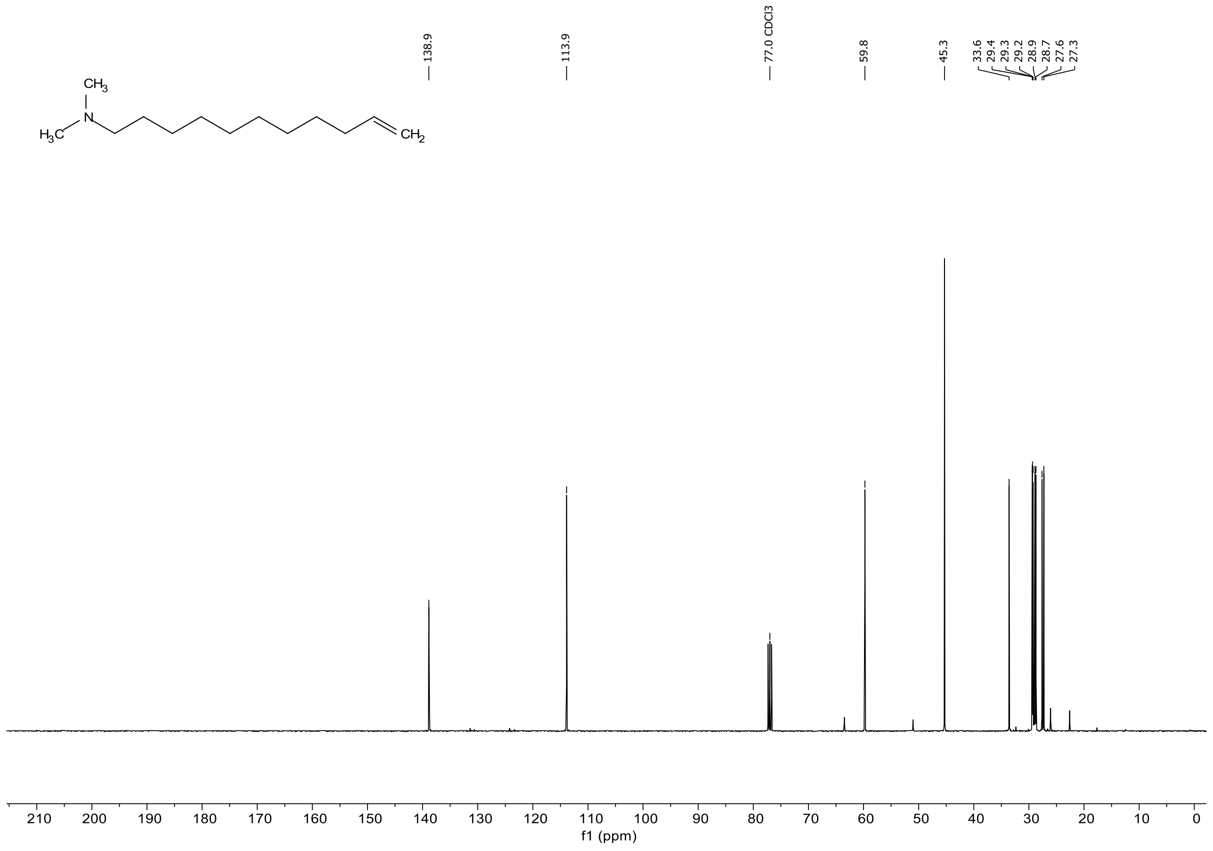

CH3
N
H3C
CH2
138.9
113.9
77.0 CDCl3
59.8
45.3
33.6
29.4
29.3
29.2
28.9
28.7
27.6
27.3
210
200
190
180
170
160
150
140
130
120
110
100
90
80
70
60
50
40
30
20
10
0
f1 (ppm)

**General procedure for the synthesis of quaternary ammonium salts S2–S4**

Adapted from the method by Thebault *et al.*[5] In a dry MW vial, amine **S1** (0.200 g, 1.01 mmol, 1.0 eq.), was added. The respective amount of 1-bromo-hexane (0.17 mL, 1.22 mmol), 1-bromo-decane (0.25 mL, 1.22 mmol) or 1-bromo-hexdecane (0.37 mL, 1.22 mmol) was added to the respective vials. The reaction was sparged with $N_2$ for 15 mins and stirred at 80 °C without solvent for 48 hrs. Cold hexane was used to triturate the products, giving white solids that were dried *in vacuo*. The products were carried forward without any further purification. The observed analytical data matched that of the original work for the series of molecules synthesised.[5]

N-hexyl-N,N-dimethylundec-10-en-1-aminium bromide **S2**

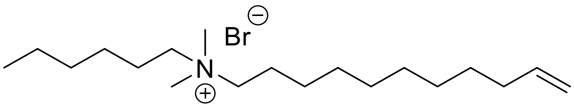


Yield: white solid (0.312 g, 0.86 mmol, 85 %). **$^1$H NMR** (400 MHz, $CDCl_3$) δ 5.88–5.73 (m, 1H), 5.04–4.90 (m, 2H), 3.54–3.48 (m, 4H), 3.40 (s, 6H), 2.09–1.98 (m, 2H), 1.76–1.65 (m, 4H), 1.50–1.24 (m, 18H), 0.90 (d, $J$ = 6.9 Hz, 3H). **ESI-MS** *m/z calcd* for $C_{19}H_{40}N^+$ [$M^+$] 282.3156 *found* 282.3155.

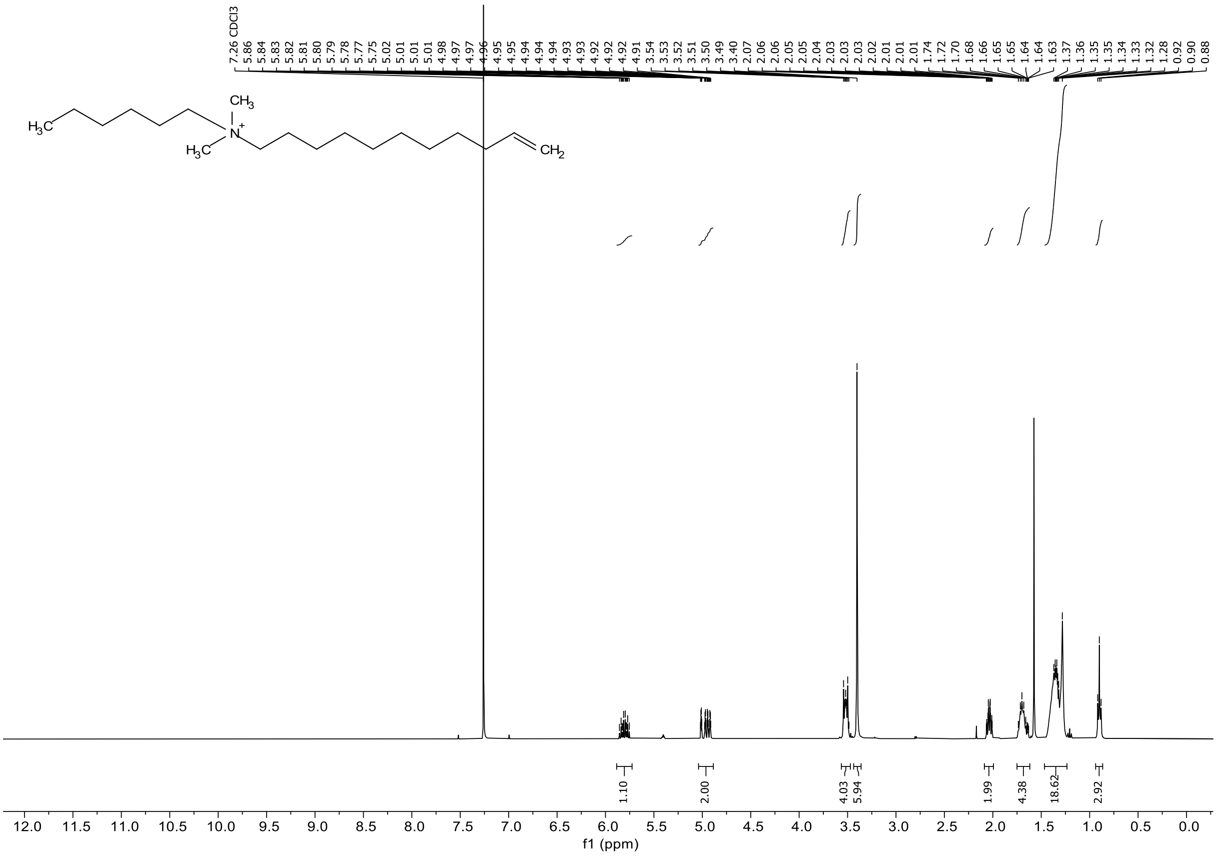

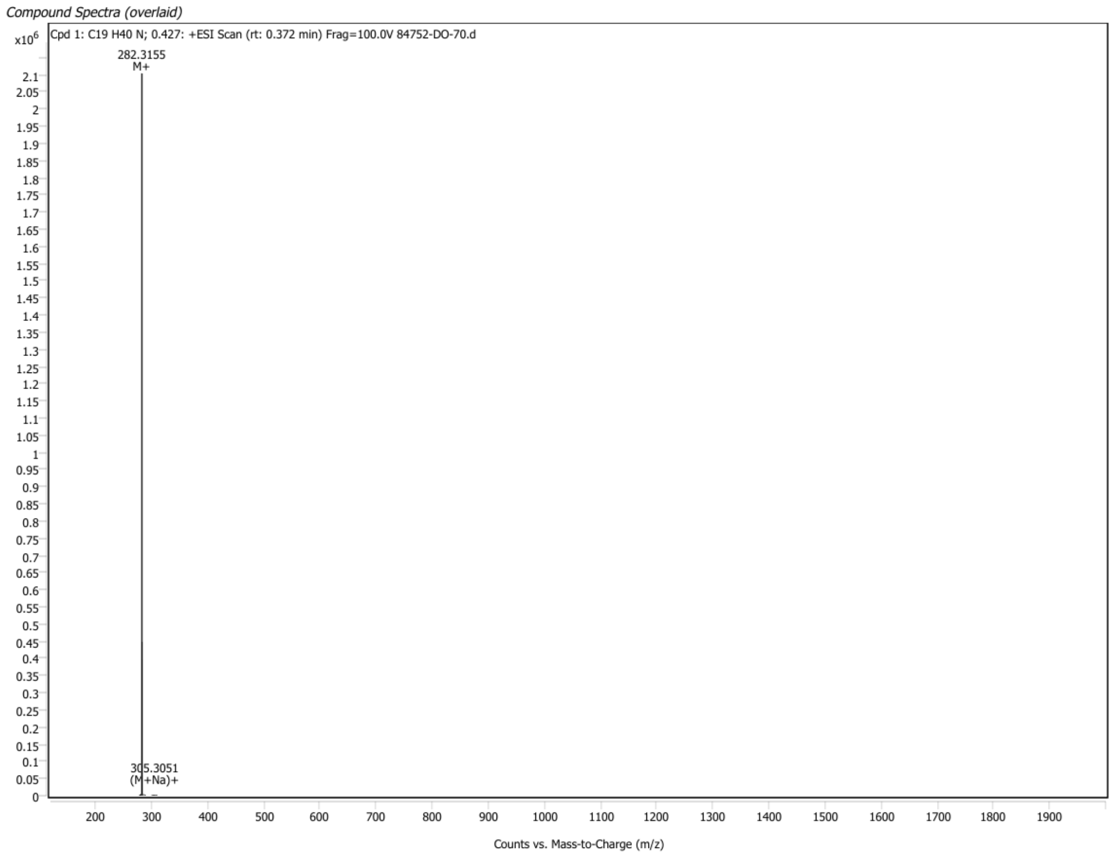
Compound Spectra (overlaid)
Cpd 1: C19 H40 N; 0.427: +ESI Scan (rt: 0.372 min) Frag=100.0V 84752-DO-70.d
x10^6
282.3155
M+
305.3051
(M+Na)+
Counts vs. Mass-to-Charge (m/z)

N-decyl-N,N-dimethylundec-10-en-1-aminium bromide **S3**

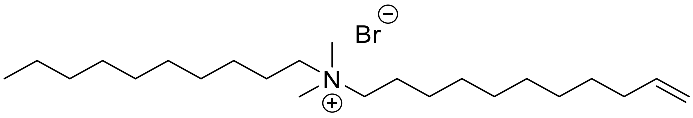


Yield: white solid (0.352 g, 0.84 mmol, 84 %). **$^1$H NMR** (400 MHz, $CDCl_3$) δ 5.86–5.74 (m, 1H), 5.05–4.87 (m, 2H), 3.56–3.47 (m, 4H), 3.41 (s, 6H), 2.07–2.00 (m, 2H), 1.75–1.62 (m, 4H), 1.46–1.22 (m, 26H), 0.88 (t, *J* = 6.4 Hz, 3H). **ESI-MS** *m/z calcd* for $C_{23}H_{48}N^+$ [$M^+$] 338.3782 *found* 338.3781.

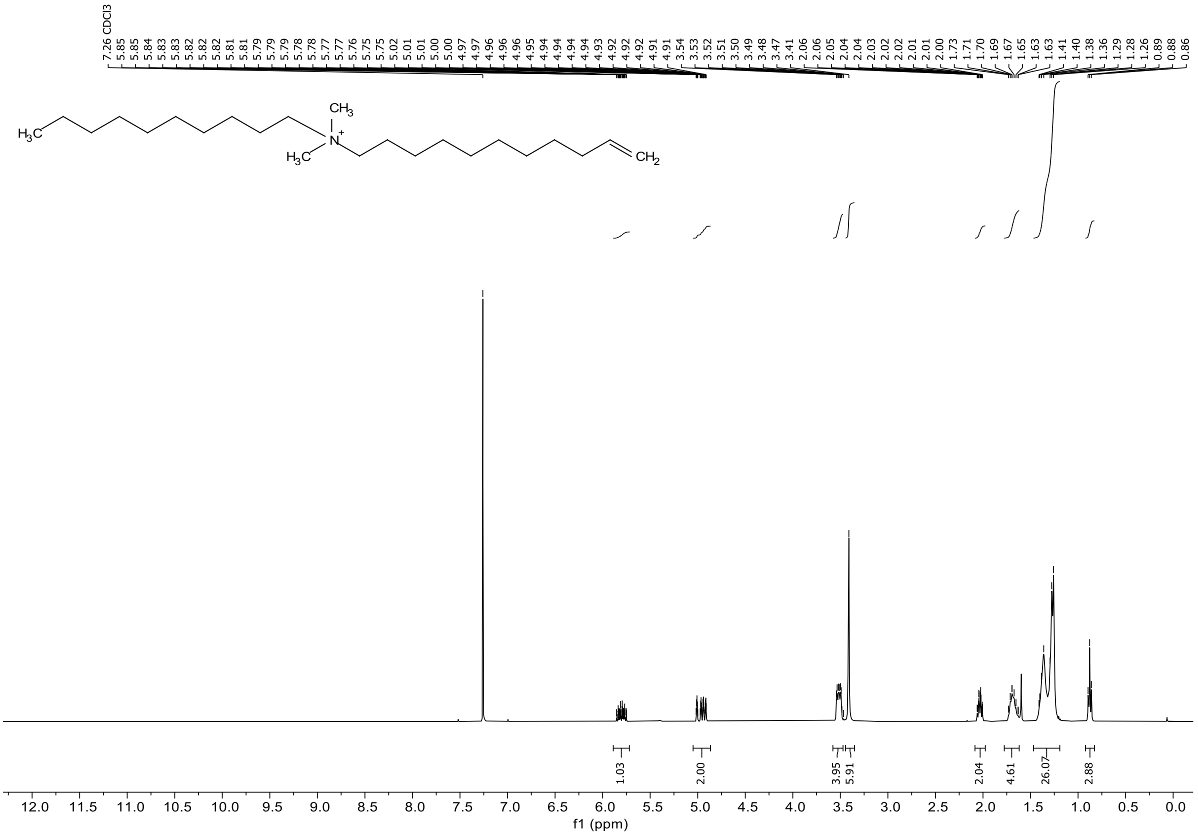

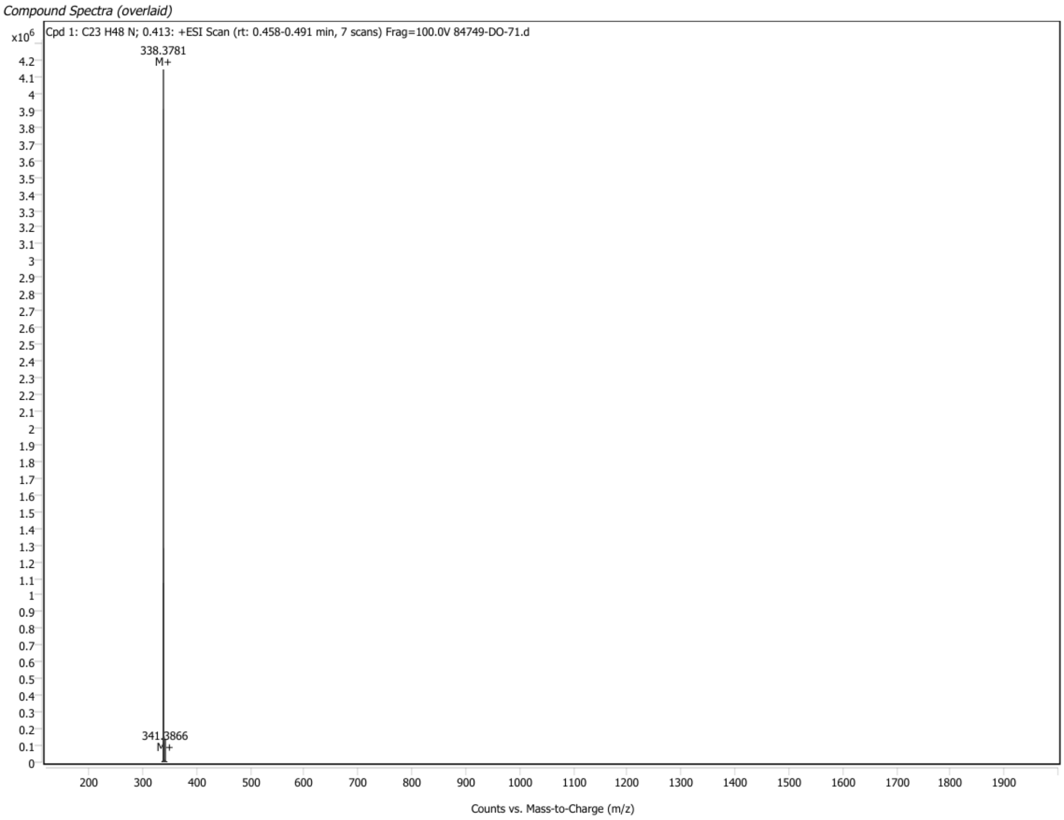

Compound Spectra (overlaid)
Cpd 1: C23 H48 N; 0.413: +ESI Scan (rt: 0.458-0.491 min, 7 scans) Frag=100.0V 84749-DO-71.d
x10^6
338.3781
M+
341.3866
M+
Counts vs. Mass-to-Charge (m/z)

N,N-dimethyl-N-(undec-10-en-1-yl)hexadecan-1-aminium bromide **S4**

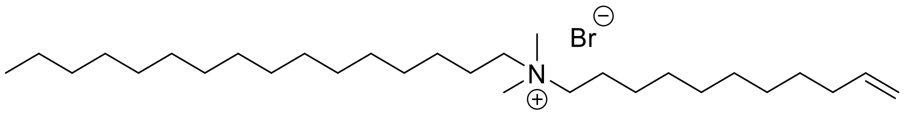


Yield: white solid (0.413 g, 0.82 mmol, 81 %). **$^1$H NMR** (400 MHz, $CDCl_3$) δ 5.90–5.74 (m, 1H), 5.04–4.89 (m, 2H), 3.56–3.45 (m, 4H), 3.41 (s, 6H), 2.09–1.98 (m, 2H), 1.76–1.64 (m, 4H), 1.39–1.23 (m, 38H), 0.88 (t, *J* = 6.6 Hz, 3H). **ESI-MS** *m/z calcd* for $C_{29}H_{60}N^+$ [$M^+$] 422.4721 *found* 422.4714.

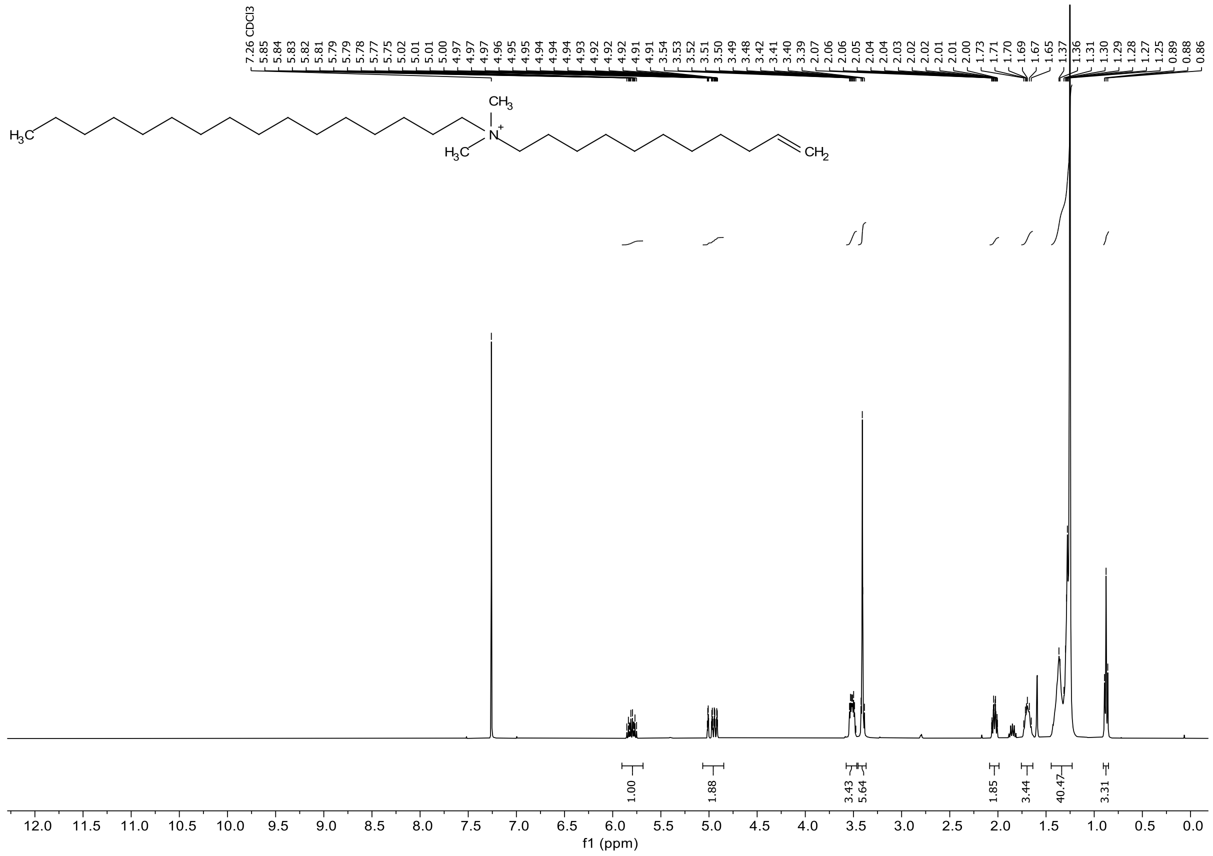

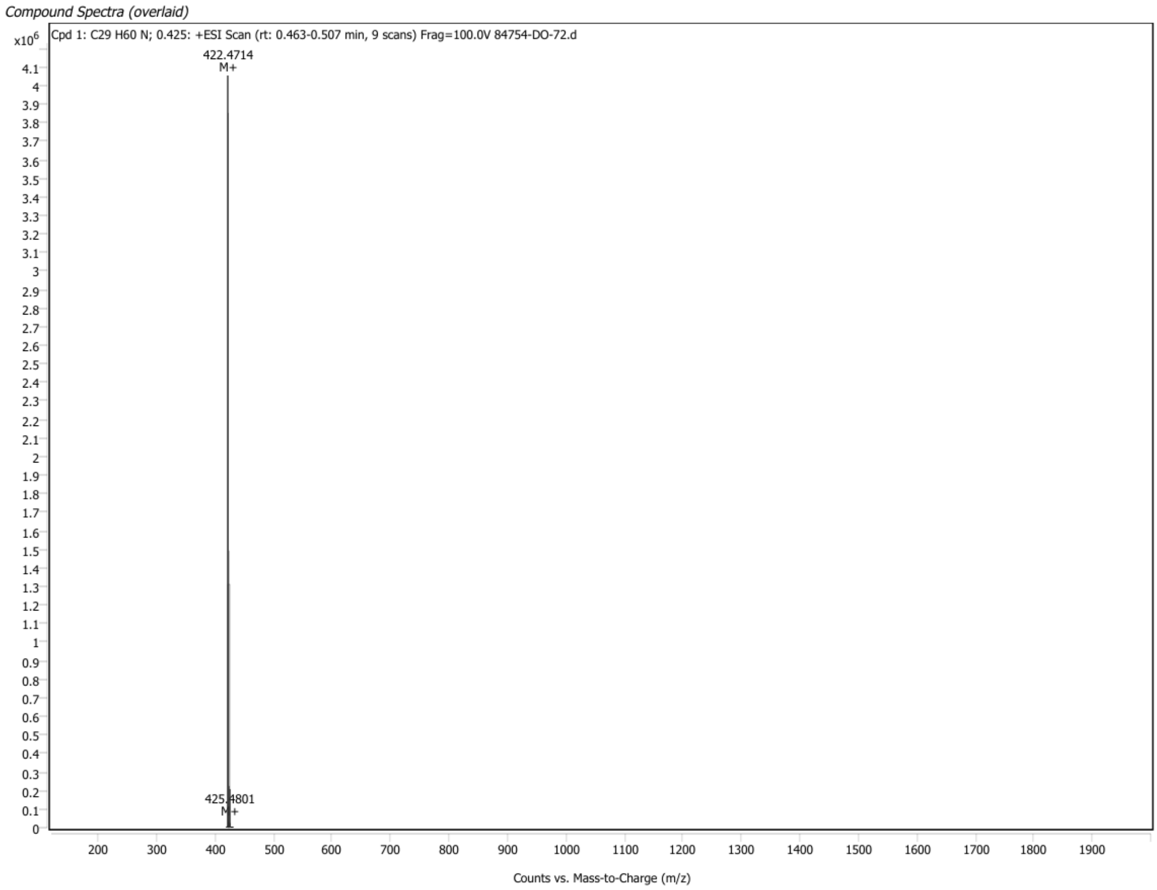


**General procedure for the synthesis of thioesters S5–S7**

To the respective alkene, starting from **S2** (0.409 g, 1.128 mmol), **S3** (0.352 g, 0.841 mmol) or **S4** (0.331 g, 0.658 mmol) in anhydrous THF (0.2 M) was added AIBN (0.4 eq). The mixture was sparged with $N_2$ for 10 mins, then thioacetic acid (4.0 eq.) was added. The solution was heated at 70 °C for 16 hrs under $N_2$. Once cooled, the solution was concentrated *in vacuo*. The products were triturated several times with $Et_2O$ and dried further *in vacuo* to yield the corresponding thioesters.

11-(acetylthio)-N-hexyl-N,N-dimethylundecan-1-aminium bromide **S5**

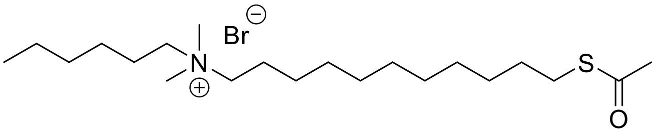

Yield: yellow oil (0.401 g, 0.91 mmol, 81%) **$^1$H NMR** (400 MHz, $CDCl_3$) δ 3.57–3.46 (m, 4H), 3.40 (s, 6H), 2.84 (t, *J* = 7.2 Hz, 2H), 2.32 (s, 3H), 1.76–1.64 (m, 4H), 1.44–1.22 (m, 22H), 0.88 (d, *J* = 7.0 Hz, 3H). **ESI-MS** *m/z calcd* for $C_{21}H_{44}NOS^+$ [$M^+$] 358.3138 *found* 358.3144.

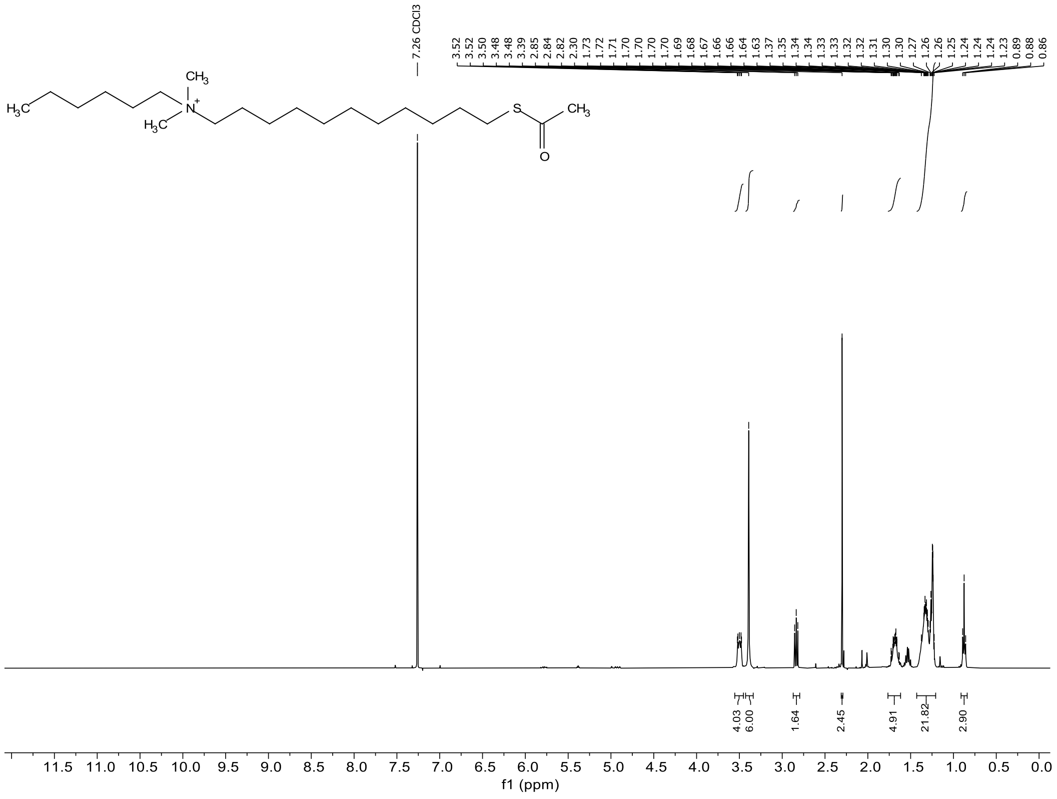

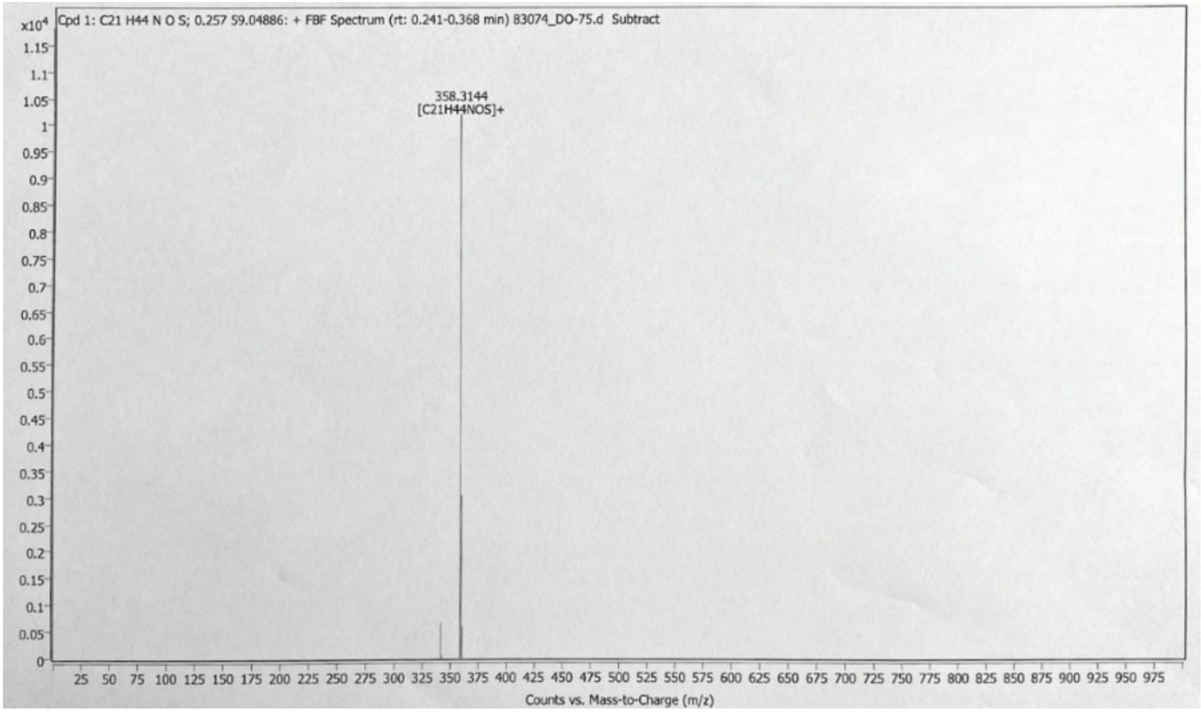
x10^4
Cpd 1: C21 H44 N O S; 0.257 59.04886: + FBF Spectrum (rt: 0.241-0.368 min) 83074_DO-75.d Subtract
358.3144
[C21H44NOS]+
Counts vs. Mass-to-Charge (m/z)

11-(acetylthio)-N-decyl-N,N-dimethylundecan-1-aminium bromide **S6**

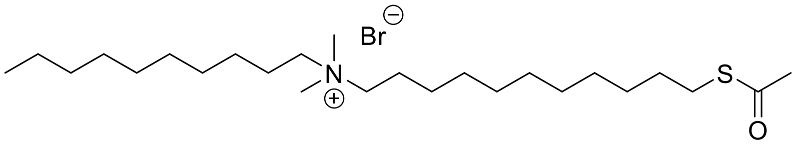


Yield: yellow oil (0.349 g, 0.71 mmol, 84%). $^{1}$**H NMR** (400 MHz, $CDCl_3$) δ 3.52–3.46 (m, 4H), 3.39 (s, 6H), 2.84 (t, *J* = 7.1 Hz, 2H), 2.30 (s, 3H), 1.77–1.62 (m, 4H), 1.58–1.49 (m, 2H), 1.41–1.22 (m, 28H), 0.86 (d, *J* = 6.8 Hz, 3H). **ESI-MS** *m/z calcd* for $C_{25}H_{52}NOS^+$ [M$^+$] 414.3764 *found* 414.3771.

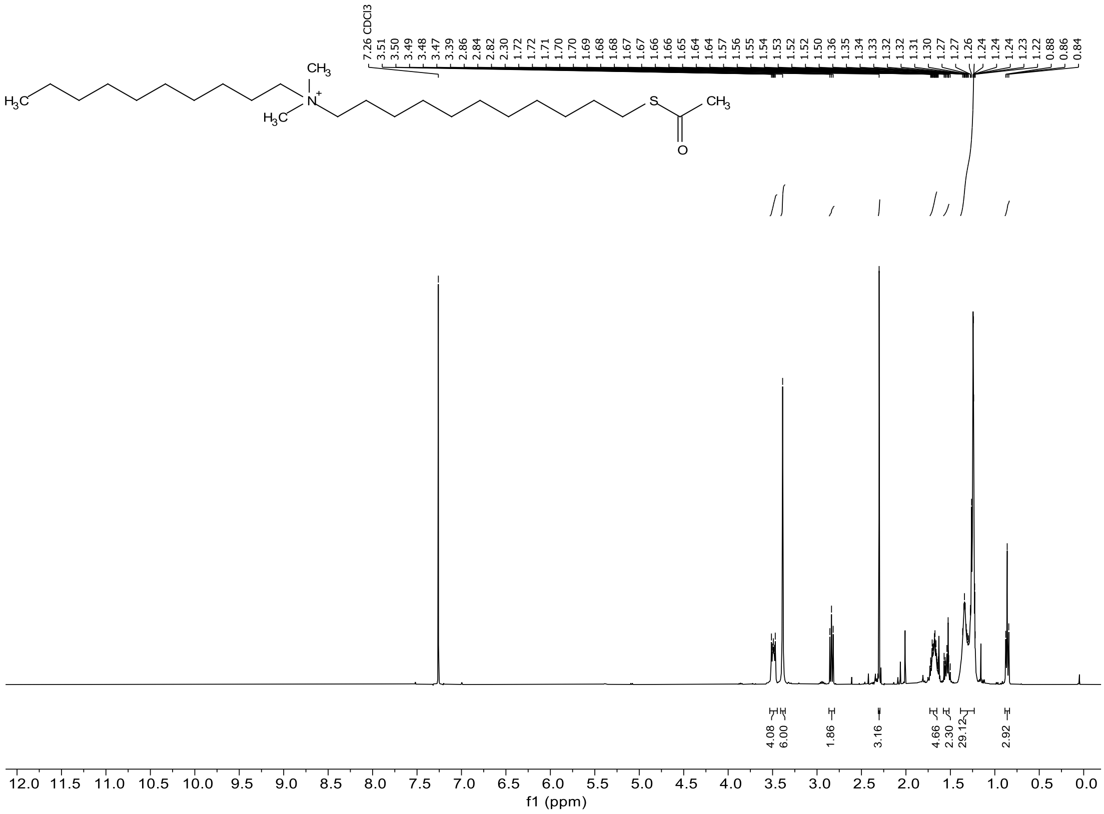

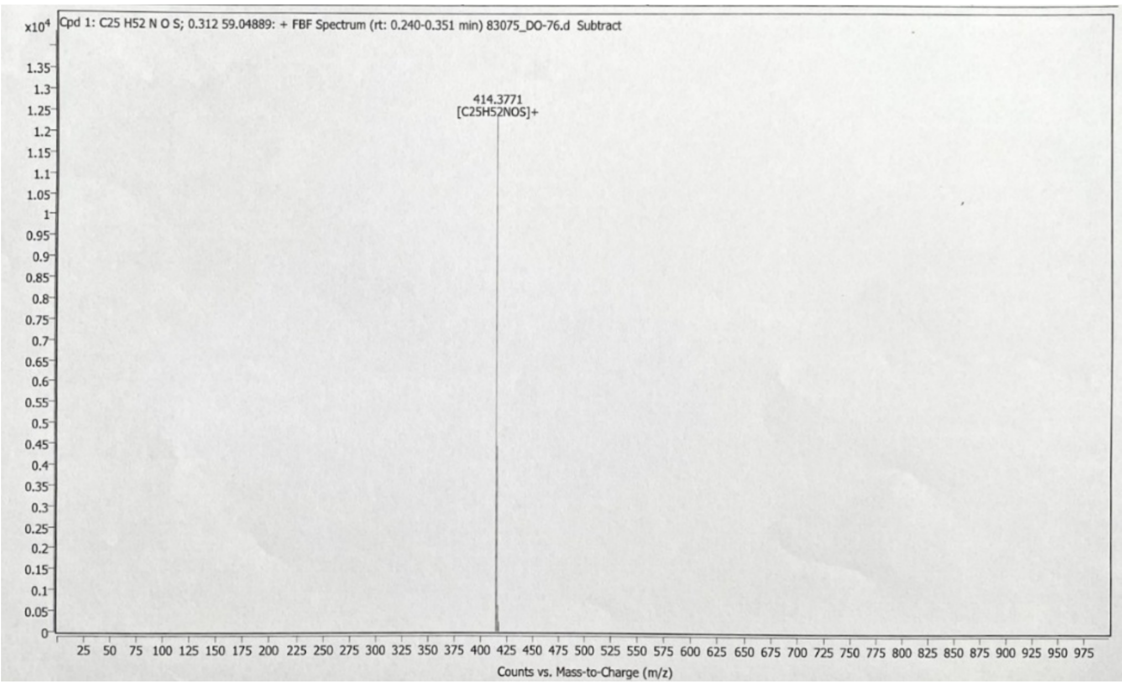

x10^4
Cpd 1: C25 H52 N O S; 0.312 59.04889: + FBF Spectrum (rt: 0.240-0.351 min) 83075_DO-76.d Subtract
414.3771
[C25H52NOS]+
Counts vs. Mass-to-Charge (m/z)

N-(11-(acetylthio)undecyl)-N,N-dimethylhexadecan-1-aminium bromide **S7**

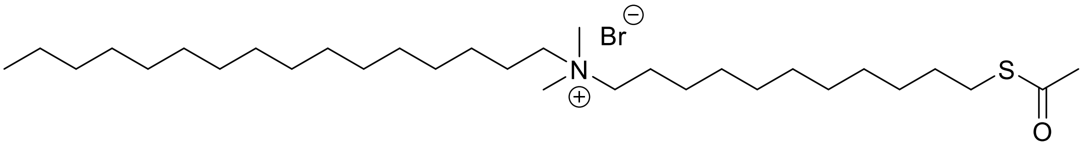


Yield: light brown solid (0.289 g, 0.50 mmol, 76%). **$^1$H NMR** (400 MHz, $CDCl_3$) δ 3.55–3.47 (m, 4H), 3.38 (s, 6H), 2.86 (t, *J* = 6.6 Hz, 2H), 2.32 (s, 3H), 1.77–1.62 (m, 4H), 1.43–1.24 (m, 42H), 0.88 (t, *J* = 6.8 Hz, 3H). **ESI-MS** *m/z calcd* for $C_{31}H_{64}NOS^+$ [$M^+$] 498.4703 *found* 498.4719.

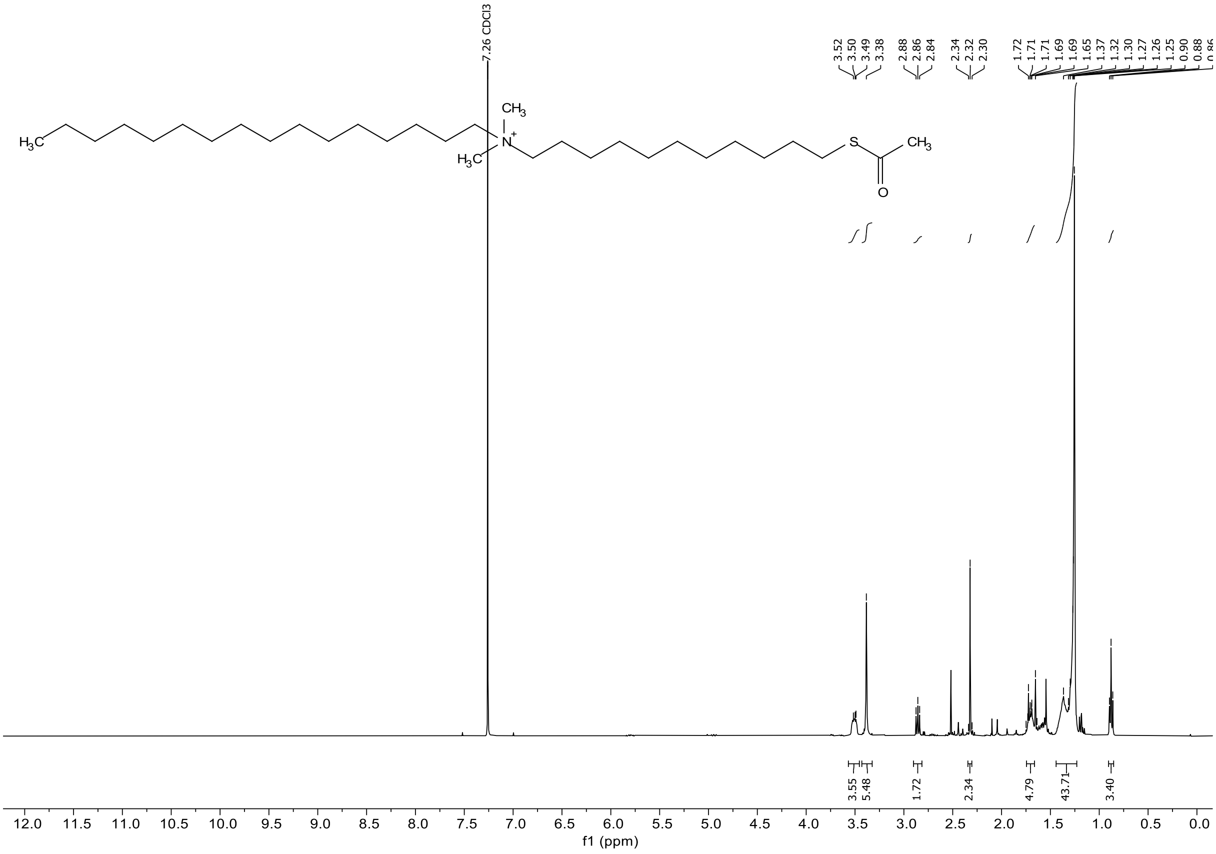


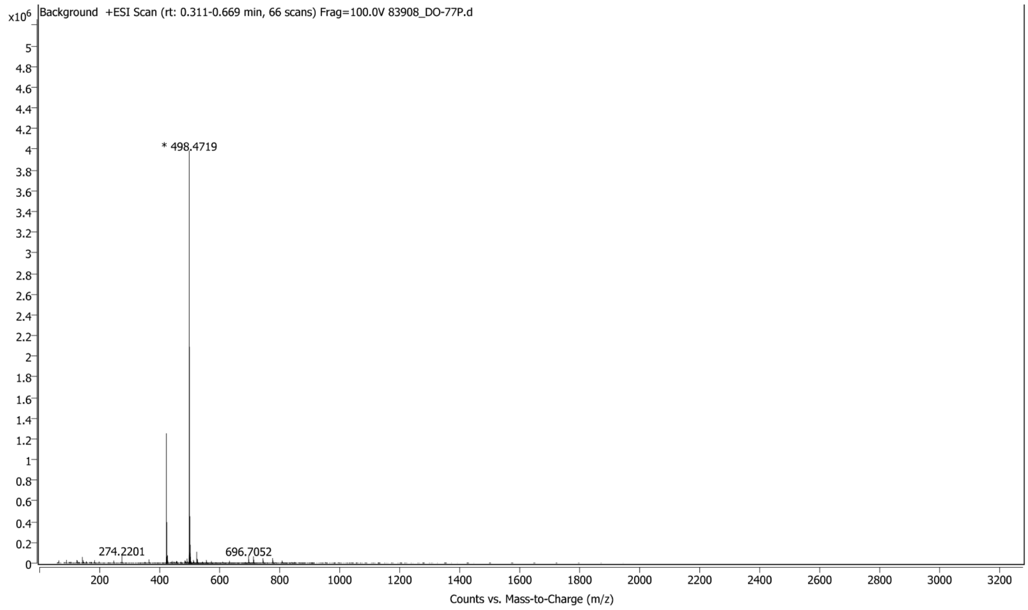

**General procedure for the synthesis of quaternary ammonium thiols 1–3**

To access the free thiols, a few milligrams (small-scale) of the respective thioesters compound was deprotected using methanolic HCl (1.25 M, >10 eq.), 3 hrs at 55 °C to give the respective free thiols. Conversion was assumed to be quantitative and material was carried forward to make the given concentrations of ink.

N-hexyl-11-mercapto-N,N-dimethylundecan-1-aminium chloride **1**

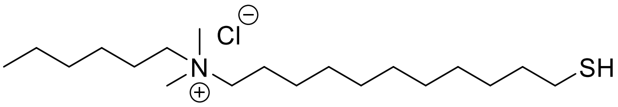


**$^1$H NMR** (400 MHz, $CDCl_3$) δ 3.57–3.47 (m, 4H), 3.40 (s, 6H), 2.52 (q, *J* = 7.4 Hz, 2H), 1.75–1.63 (m, 6H), 1.48–1.23 (m, 20H), 0.90 (t, *J* = 6.8 Hz, 3H). **ESI-MS** *m/z calcd* for $C_{19}H_{42}NS^+$ [$M^+$] 316.3038 *found* 316.3031.

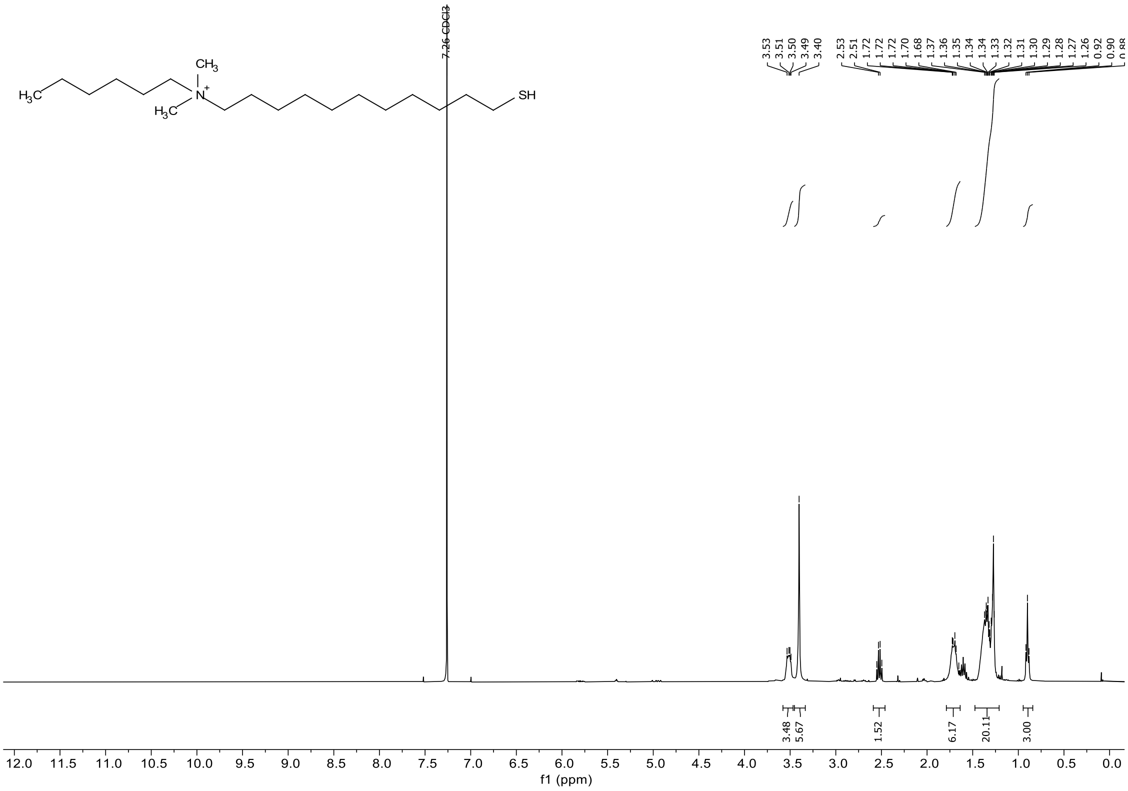

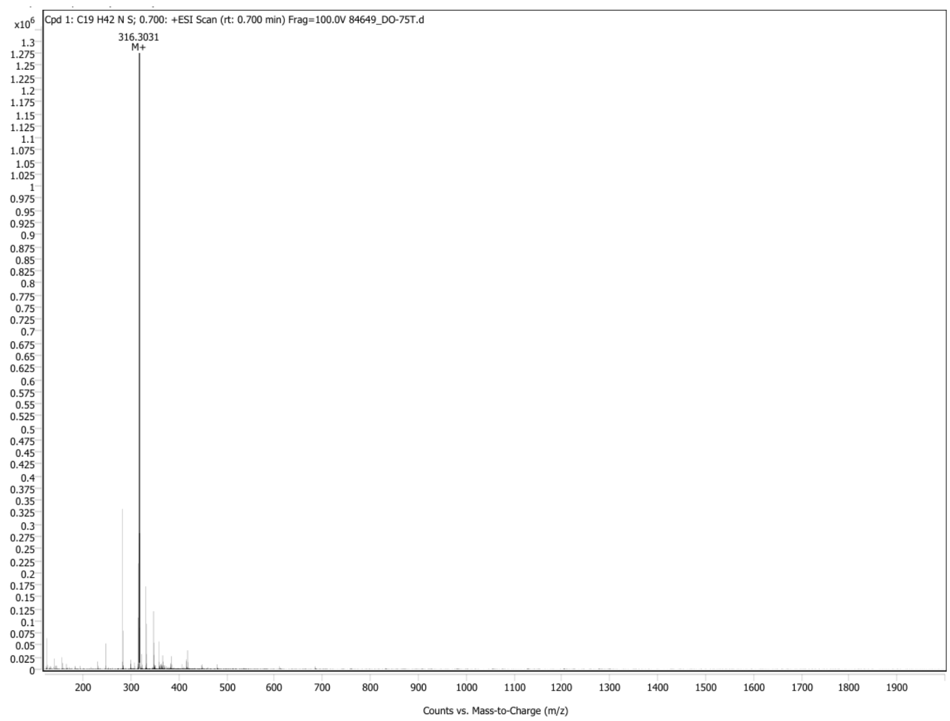
Cpd 1: C19 H42 N S; 0.700: +ESI Scan (rt: 0.700 min) Frag=100.0V 84649_DO-75T.d
x10^6
316.3031
M+
Counts vs. Mass-to-Charge (m/z)

N-decyl-11-mercapto-N,N-dimethylundecan-1-aminium chloride **2**

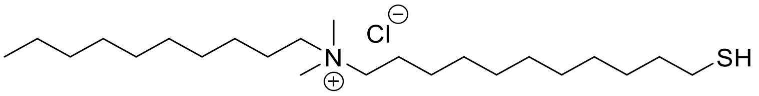


$^1$**H NMR** (400 MHz, MeOD) δ 3.30–3.26 (m, 4H), 3.06 (s, 6H), 2.49 (t, *J* = 7.2 Hz, 2H), 1.80–1.69 (m, 4H), 1.64–1.54 (m, 2H), 1.48–1.27 (m, 28H), 0.90 (t, *J* = 6.7 Hz, 3H). **ESI-MS** *m/z calcd* for $C_{23}H_{50}NS^+$ [$M^+$] 372.3664 *found* 372.3768.

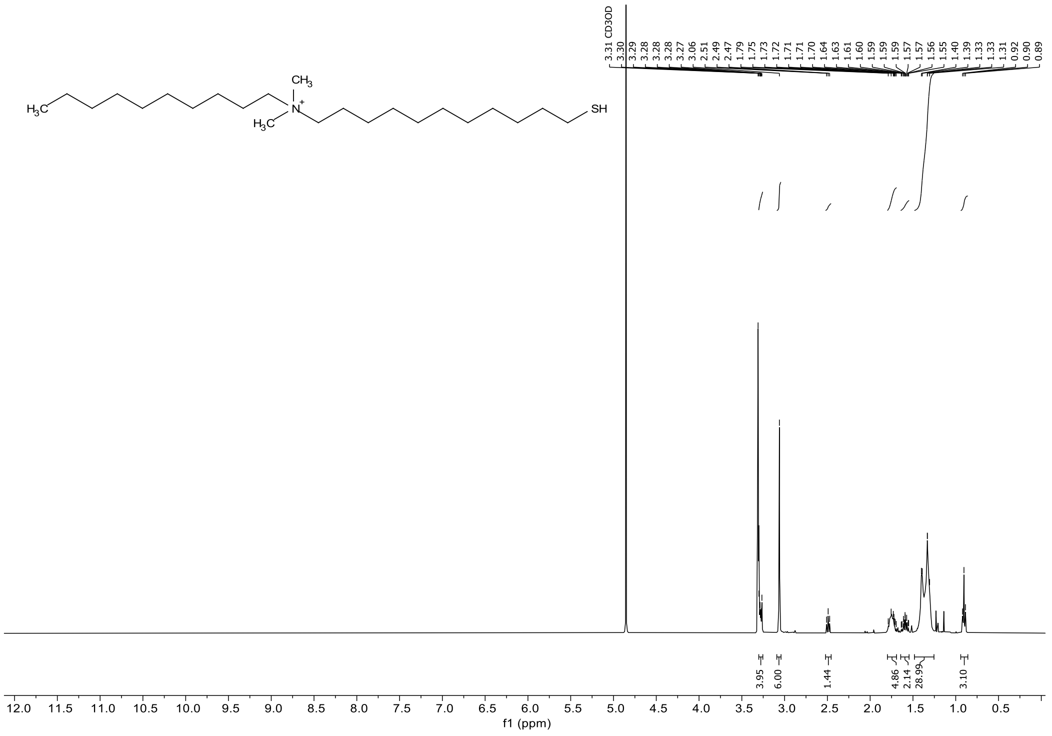

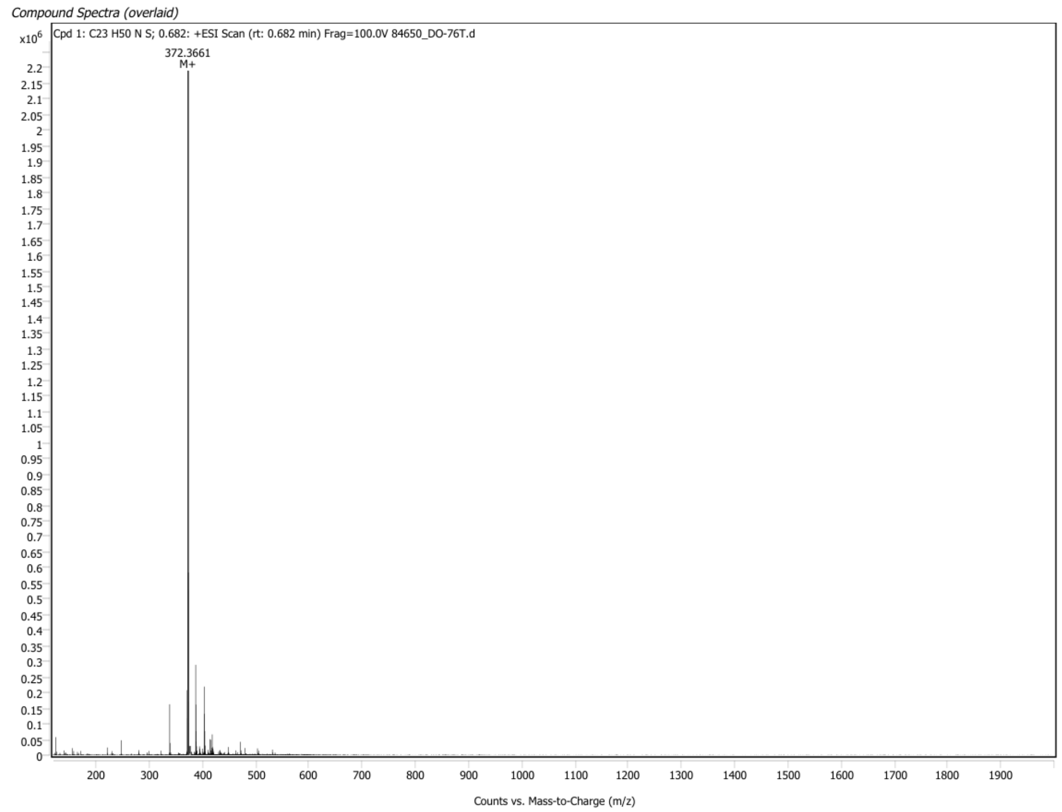


N-(11-mercaptoundecyl)-N,N-dimethylhexadecan-1-aminium chloride **3**

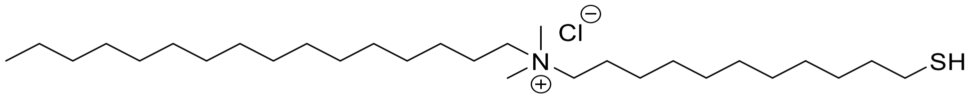

**$^1$H NMR** (400 MHz, $CDCl_3$) δ 3.51–3.42 (m, 4H), 3.37 (s, 6H), 2.52 (t, *J* = 7.4 Hz, 2H), 1.74–1.64 (m, 4H), 1.15–1.30 (m, 40H), 0.91–0.85 (m, 3H). **ESI-MS** *m/z calcd* for $C_{29}H_{62}NS^+$ [$M^+$] 456.4603 *found* 456.4593.

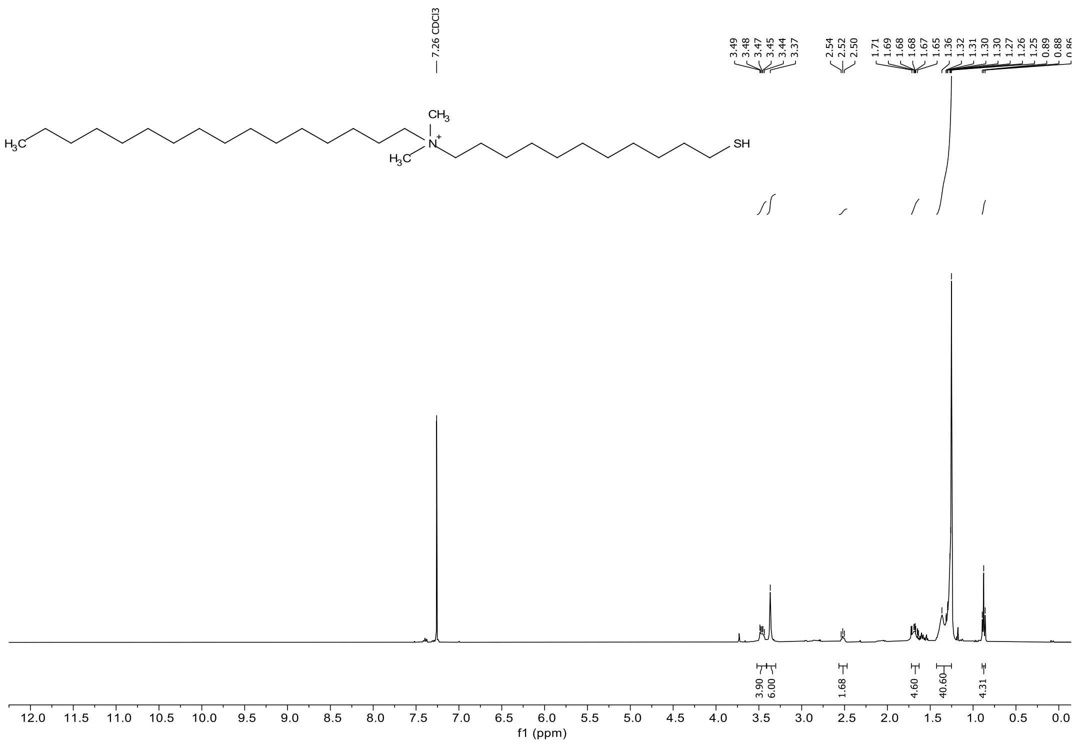

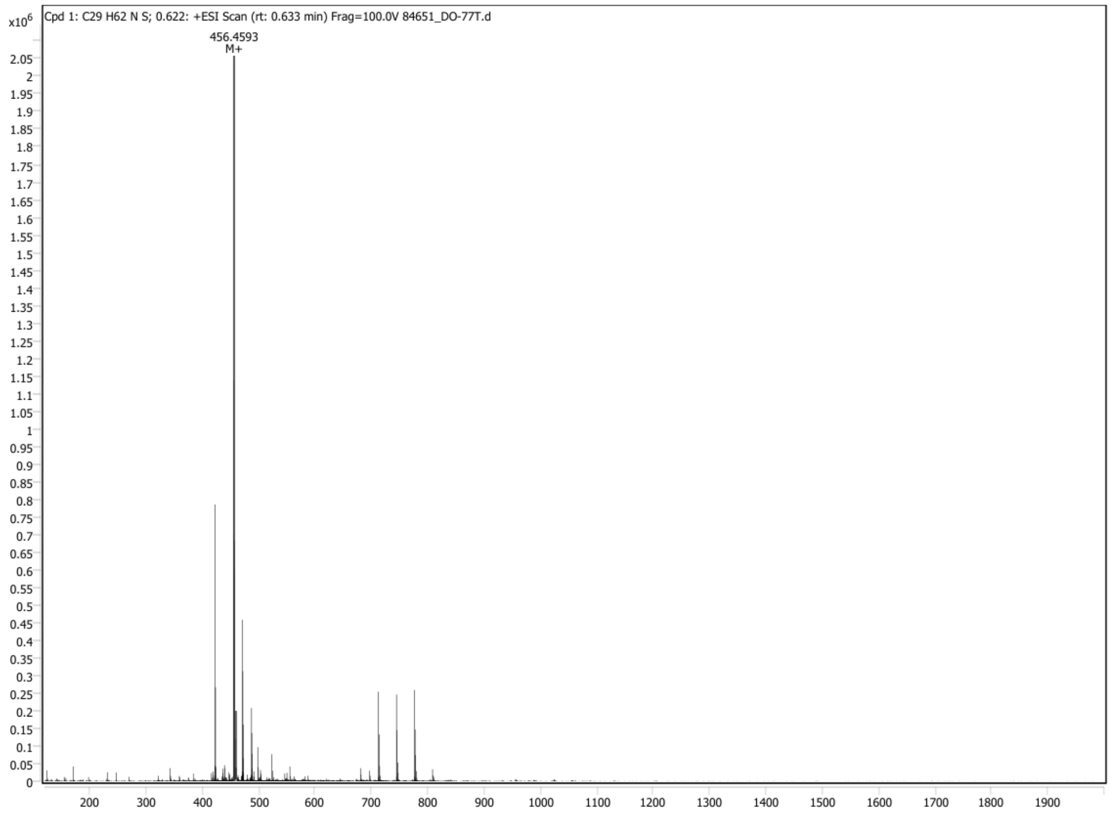


## Synthesis of guanidine-based (alk11) Ligands 4–6

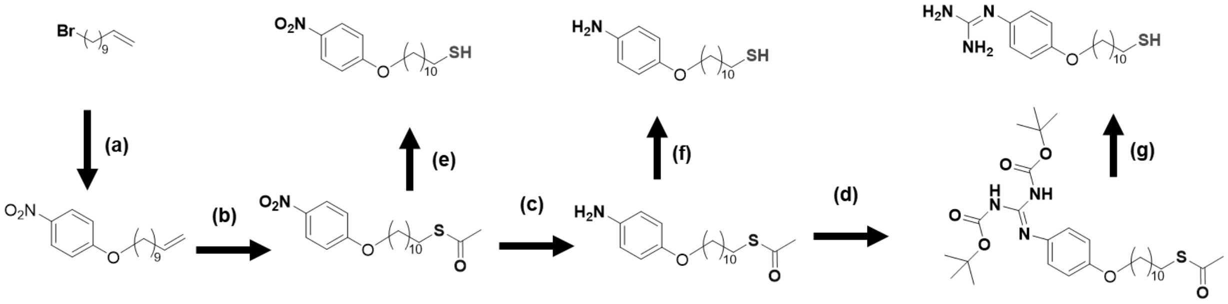


(a) $K_2CO_3$, Acetone, 60°C, 54% (b) AIBN, anhydrous THF, $N_2$, 80°C, 16h, 69% (c) $H_2$, Pd/C, THF, RT, 36h, 31 % (d) N,N'-Bis(tert-butoxycarbonyl)-N''-triflylguanidine, $Et_3N$, DCM, RT, 62% (e) & (f) 1.25 M HCl in MeOH (> 10 eq.), 3h, 55 °C quantitative (g) HCl/MeOH/$H_2O$ (2M, > 10 eq.) 3h, 55°C, quantitative

1-Nitro-4-(undec-10-en-1-yloxy)benzene **S8**

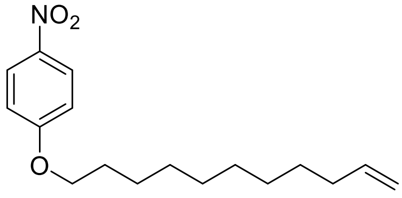

A solution of p-nitrophenol (3.89 g, 28.00 mmol) in acetone (50 mL at 60 °C) was refluxed in the presence of $K_2CO_3$ (10.00 g, 72.35 mmol). After 30 mins, 11-bromoundec-1-ene (3.20 g, 13.80 mmol) was added and the mixture was refluxed for a further 24 hrs. After cooling to RT, the mixture was filtered and the acetone removed *in vacuo*. The residue was extracted into dichloromethane (60 mL) and washed with sat. aq. $Na_2CO_3$ (3 × 60 mL), $H_2O$ (60 mL), and sat. brine (60 mL). The organic phase was dried ($MgSO_4$), and the solvent removed *in vacuo*. Purification by silica column chromatography (5% EtOAc/hexanes) yielded **S8** a white solid

(2.171 g, 7.45 mmol, 54%). Observed analytical data matched that of the original work.[6] $^{1}$**H NMR** (400 MHz, $CDCl_3$) δ 8.17 (d, $J$ = 9.2 Hz, 2H), 6.93 (d, $J$ = 9.2 Hz, 2H), 5.87–5.73 (m, 1H), 5.04–4.89 (m, 2H), 4.08–3.99 (m, 2H), 2.04 (app.q, $J$ = 7.1 Hz, 2H), 1.82 (app.p, $J$ = 6.7 Hz, 2H), 1.49–1.25 (m, 12H).$^{13}$**C NMR** (101 MHz, $CDCl_3$) δ 164.2, 141.2, 139.1, 125.8, 114.3, 114.1, 68.8, 33.7, 29.4, 29.3, 29.2, 29.0, 28.9, 28.8, 25.8.

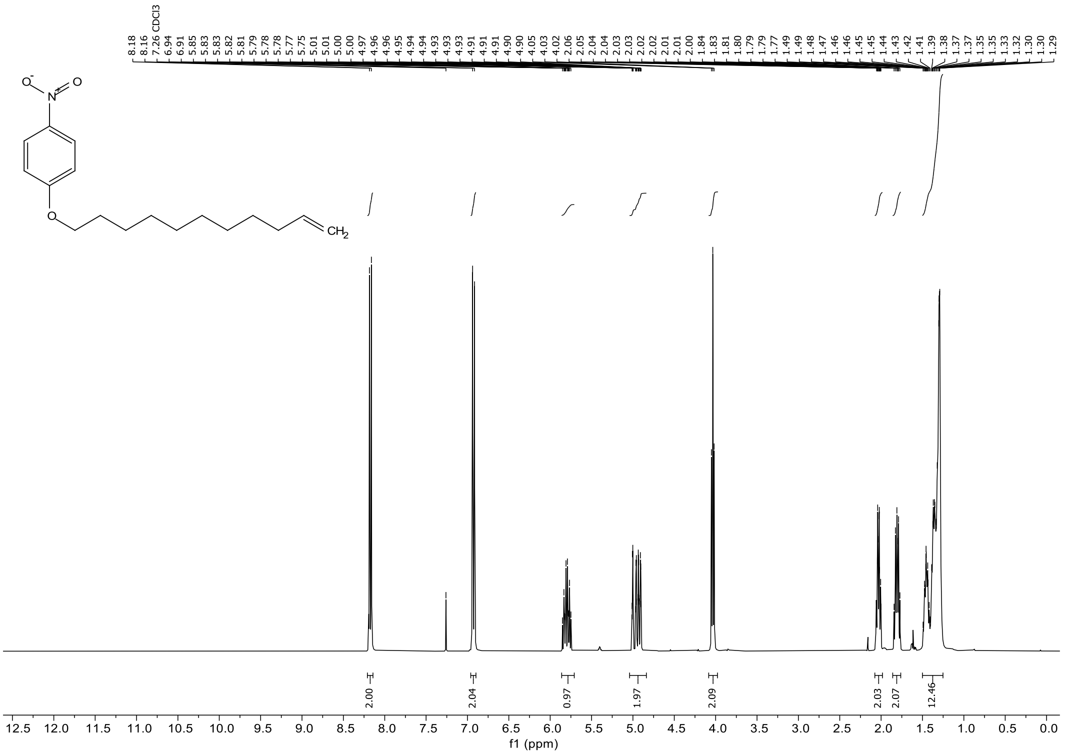


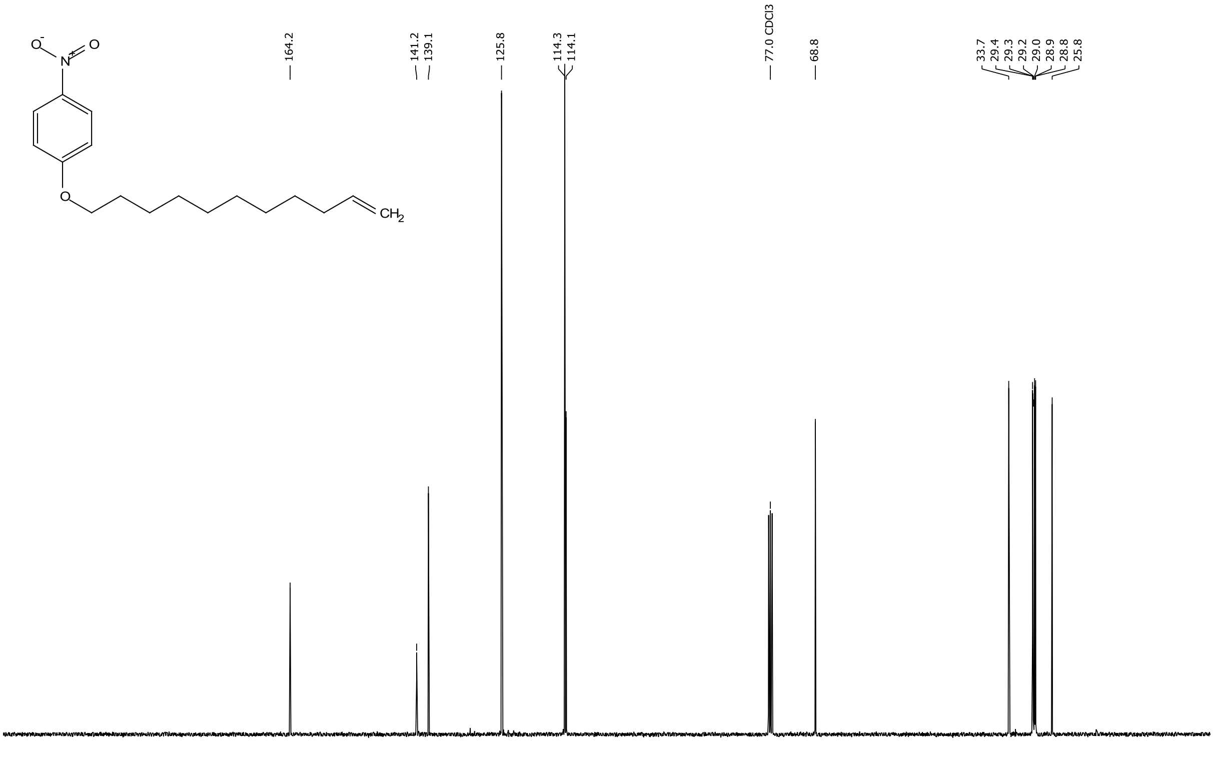

S-(11-(4-nitrophenoxy)undecyl) ethanethioate **S9**

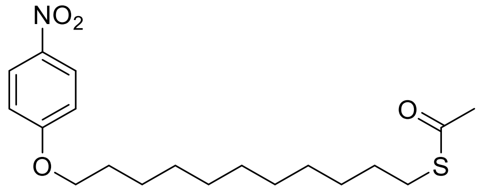

Alkene **S8** (1.40 g, 4.81 mmol) and AIBN (0.47 g, 2.89 mmol) were dissolved in anhydrous toluene (35 mL) under $N_2$ and purged for 15 mins. Thioacetic acid (1.83 g, 24.04 mmol) was added to the reaction and the solution was heated to 75 °C for 16 hrs under $N_2$. The reaction solvent was removed *in vacuo* and the resulting mixture was redissolved in DCM (20 mL) and washed sat. aq. $Na_2CO_3$ (3 × 20 mL), $H_2O$ (25 mL), and sat. brine (25 mL). The organic phase was dried ($MgSO_4$), and the solvent removed *in vacuo*. Triturating the yellow solid with cold hexanes and EtOAc (<1 mL) gave a white solid and yellow liquid, the yellow liquid was removed. The sample was further purified by silica column chromatography (hexanes/EtOAc 40:1 to 30:1) yielding **S9** as a white solid (1.222 g, 3.32 mmol, 69%). Observed analytical data matched that of the original work.[6] **$^1$H NMR** (400 MHz, $CDCl_3$) δ 8.18 (d, $J$ = 9.3 Hz, 2H), 6.93 (d, $J$ = 9.3 Hz, 2H), 4.03 (t, $J$ = 6.5 Hz, 2H), 2.85 (t, $J$ = 7.3 Hz, 2H), 2.31 (s, 3H), 1.86–1.75 (m, 2H), 1.60–1.50 (m, 2H), 1.50–1.18 (m, 14H). **$^{13}$C NMR** (101 MHz, $CDCl_3$) δ 196.0, 164.2, 141.2, 125.8, 114.3, 68.8, 30.6, 29.4, 29.4, 29.4, 29.4, 29.2, 29.1, 29.0, 28.9, 28.7, 25.8. **ESI-MS** *m/z calcd* for $C_{19}H_{29}NO_4S$ $[M + H]^+$ 368.1896 *found* 368.1897.

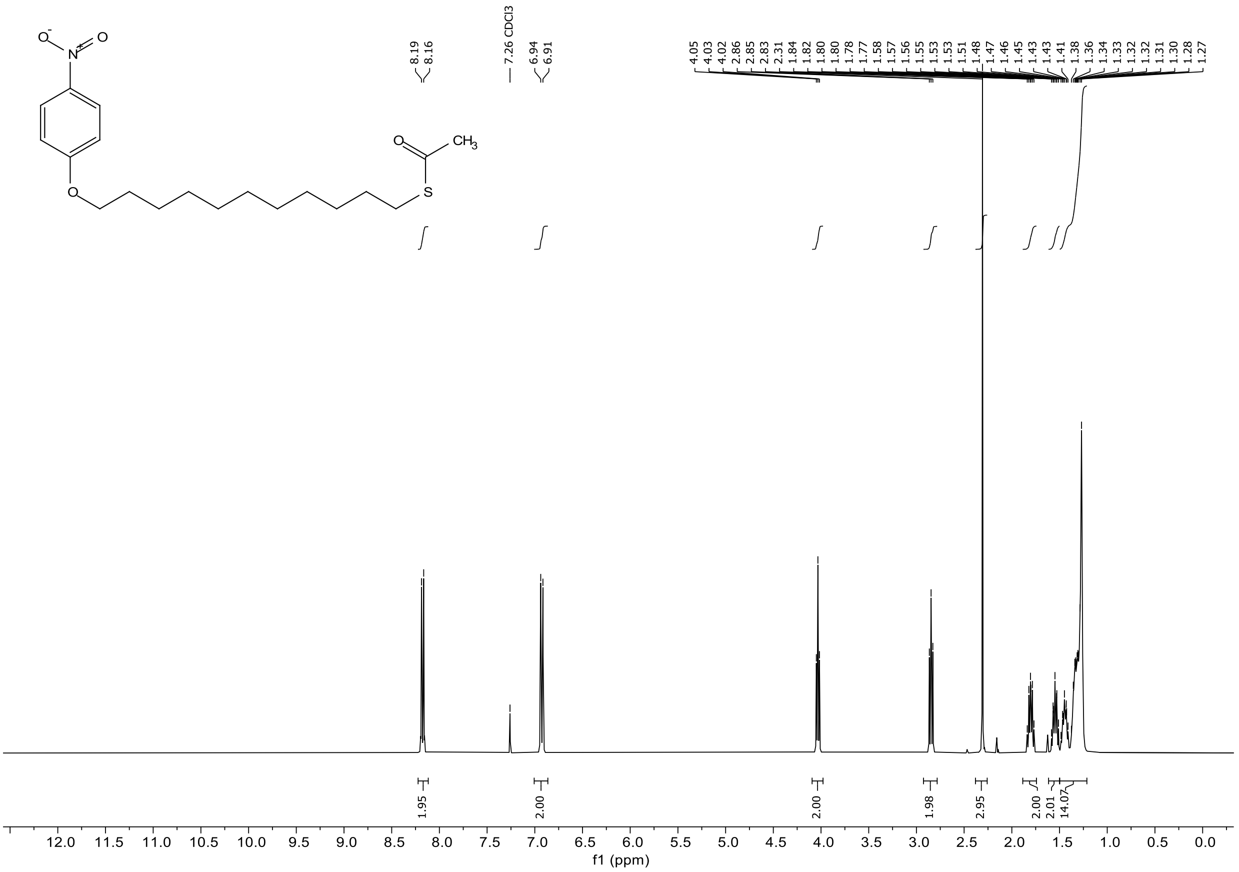

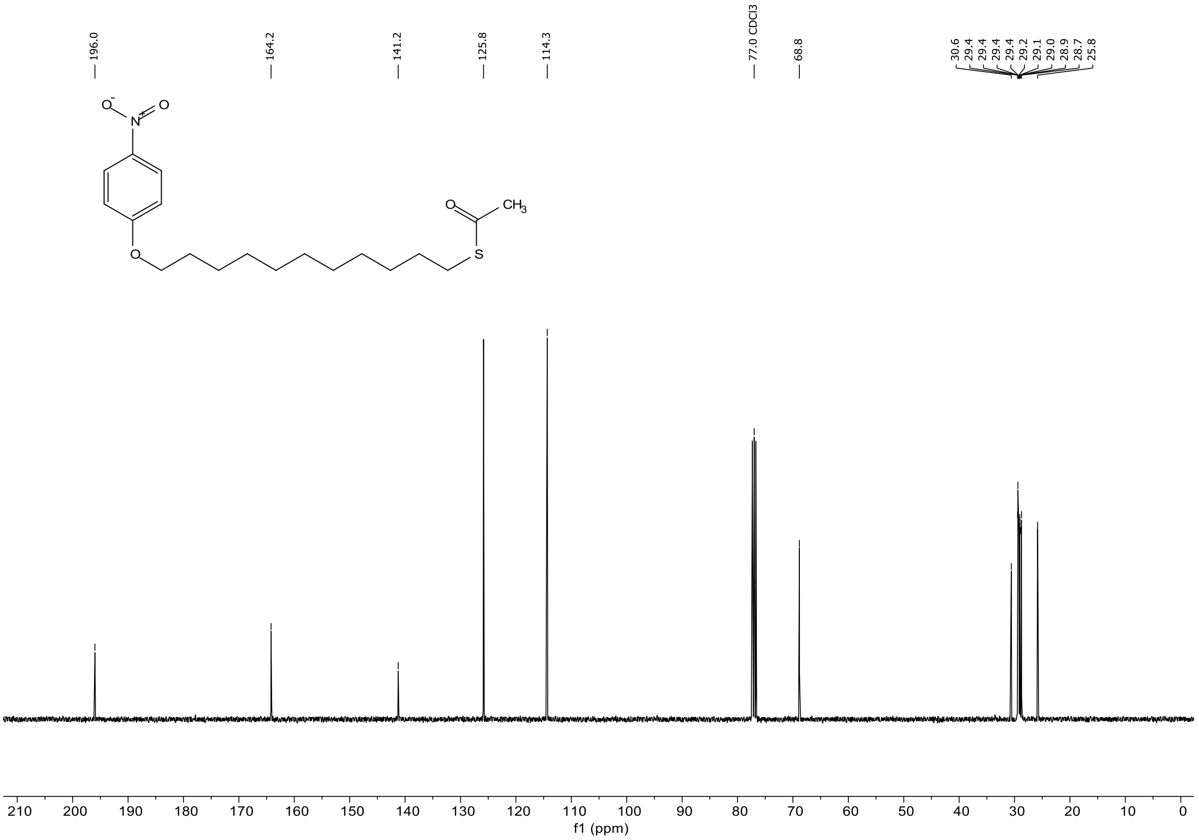
196.0
164.2
141.2
125.8
114.3
77.0 CDCl3
68.8
30.6
29.4
29.4
29.4
29.4
29.2
29.1
29.0
28.9
28.7
25.8
O
N
O
O
CH3
O
S
f1 (ppm)

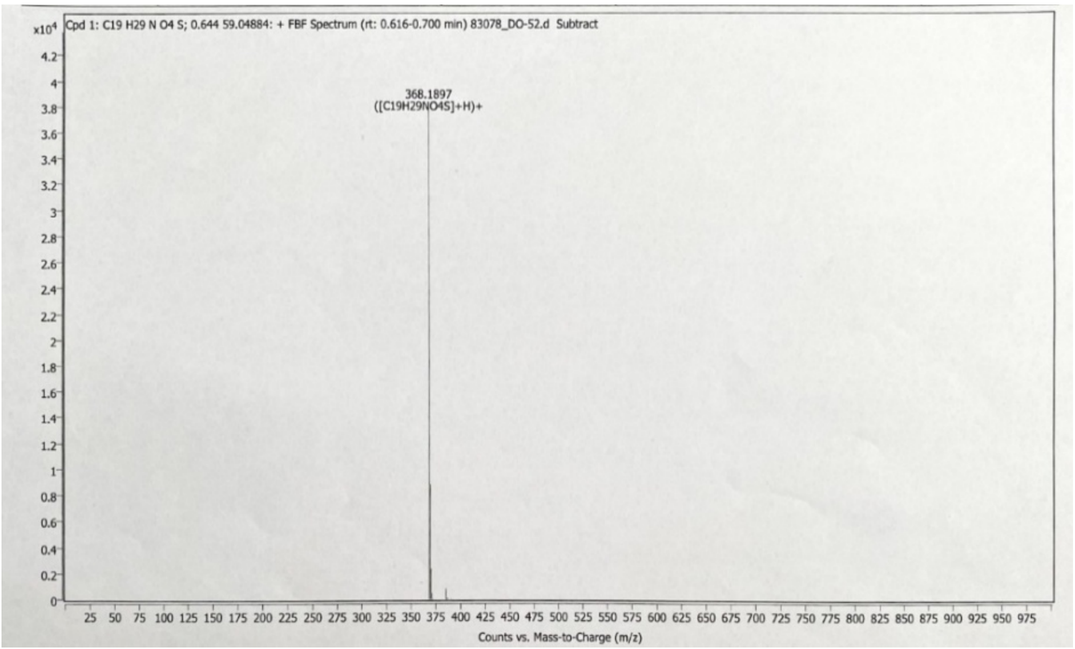
Cpd 1: C19 H29 N O4 S; 0.644 59.04884: + FBF Spectrum (rt: 0.616-0.700 min) 83078_DO-52.d Subtract
x10^4
368.1897
([C19H29NO4S]+H)+
Counts vs. Mass-to-Charge (m/z)

11-(4-Nitrophenoxy)undecane-1-thiol **4**

Thioester **S9** was deprotected using methanolic HCl (1.25M, >10 eq.), for 3 hrs at 55 °C. The solvent was evaporated, obtaining the thiol **4.**

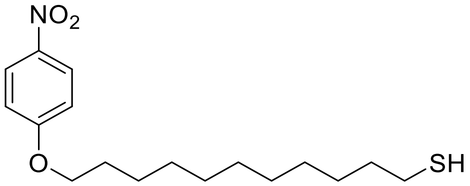


**$^1$H NMR** (400 MHz, $CDCl_3$) δ 8.19 (d, *J* = 9.2 Hz, 2H), 6.94 (d, *J* = 9.2 Hz, 2H), 4.04 (t, *J* = 6.5 Hz, 2H), 2.52 (q, *J* = 7.4 Hz, 2H), 1.87–1.78 (m, 2H), 1.66–1.57 (m, 2H), 1.51–1.42 (m, 2H), 1.41–1.24 (m, 12H). **ESI-MS** *m/z calcd* for $C_{17}H_{27}NO_3S$ $[M + H]^+$ 326.1784 *found* 326.1778

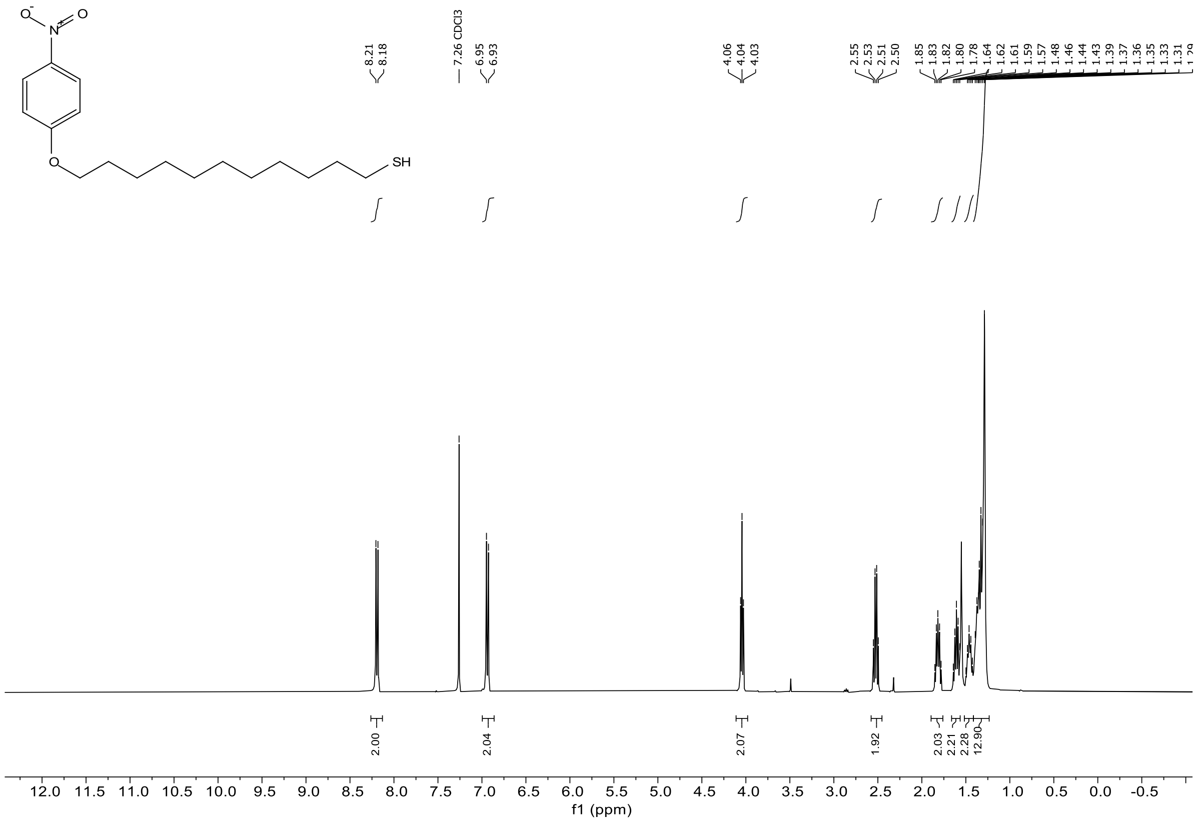


S-(11-(4-aminophenoxy)undecyl) ethanethioate **S10**

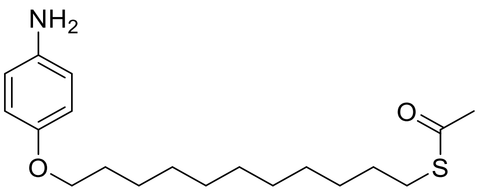


Nitroaromatic **S9** (0.400 g ,1.09 mmol) was dissolved in THF (10 mL) and Pd/C (10% Pd, 50 mg, 0.047 mmol) was added. The mixture was stirred at RT under $H_2$ atmosphere for 36 h. After filtration through a Celite plug and evaporation of the solvent *in vacuo*, the crude product was purified by silica column chromatography (hexanes/EtOAc from 15:1 to 8:1) obtaining the product **S10** as a white solid (0.112 g, 0.33 mmol, 31%). Observed analytical data matched

that of the original work.[6] $^1$**H NMR** (400 MHz, $CDCl_3$) δ 6.74 (d, *J* = 8.8 Hz, 2H), 6.64 (d, *J* = 8.8 Hz, 2H), 3.87 (t, *J* = 6.6 Hz, 3H), 3.40 (br, 2H), 2.86 (t, *J* = 7.4 Hz, 2H), 2.32 (s, 3H), 1.79–1.69 (m, 2H), 1.64–1.54 (m, 2H), 1.49–1.19 (m, 14H). **ESI-MS** *m/z calcd* for $C_{19}H_{31}NO_2S$ [M + H]$^+$ 338.2148 *found* 338.2157.

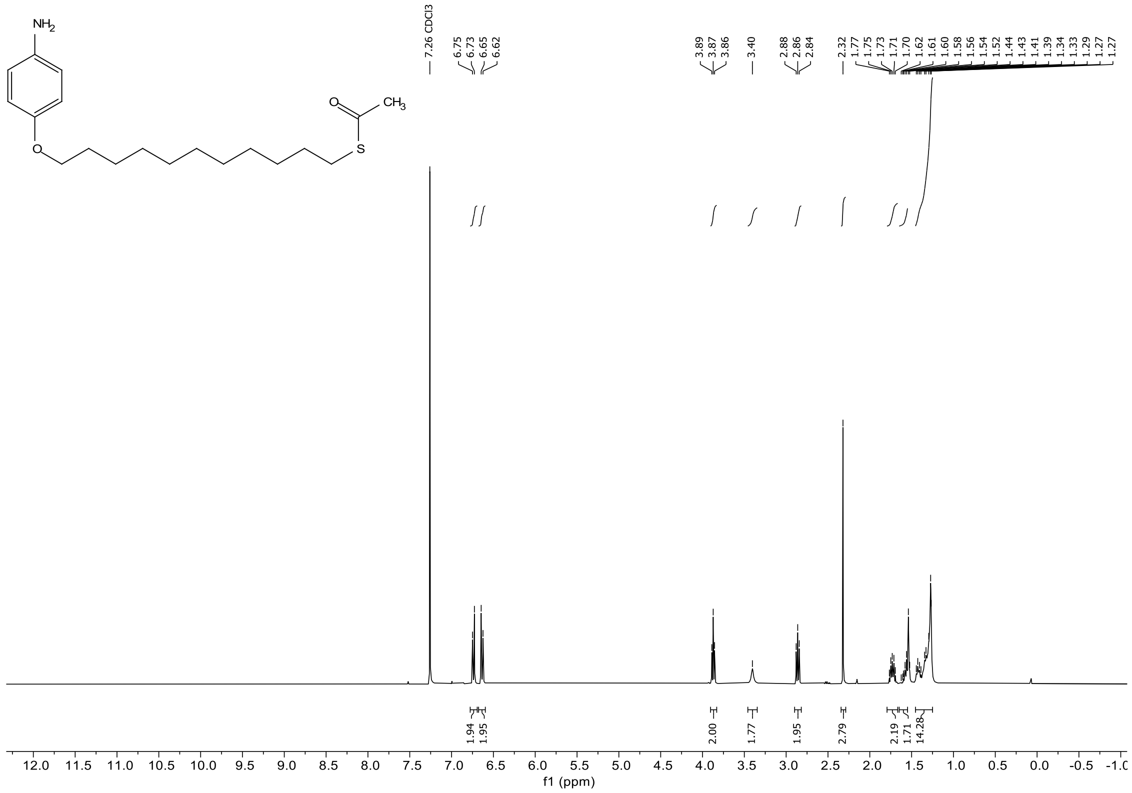


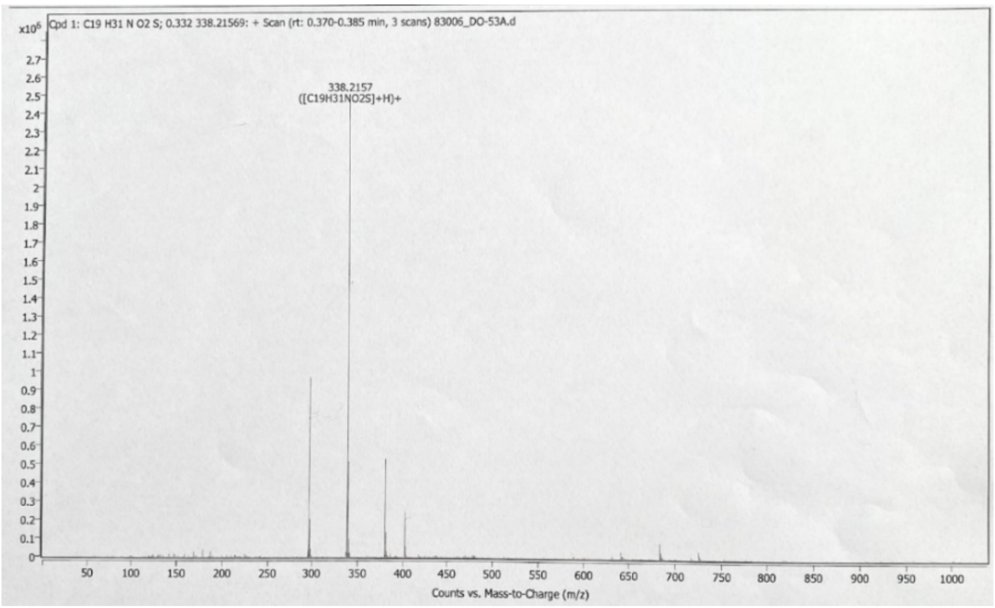

4-((11-Mercaptoundecyl)oxy)benzenaminium chloride **5**

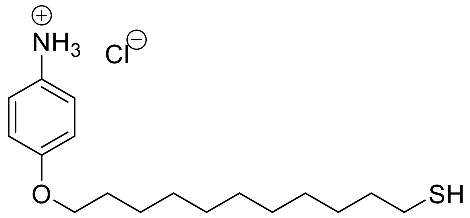


Thioester was deprotected using methanolic HCl (1.25M, >10 eq.), for 3 hrs at 55 °C. The solvent was evaporated, obtaining thiol **5**. **$^1$H NMR** (400 MHz, $CDCl_3$) δ 10.36 (br, 3H), 7.44 (d, *J* = 9.0 Hz, 2H), 6.91 (d, *J* = 9.0 Hz, 2H), 3.93 (t, *J* = 6.6 Hz, 2H), 2.52 (q, *J* = 7.4 Hz, 2H), 1.82–1.71 (m, 2H), 1.66–1.55 (m, 2H), 1.53–1.17 (m, 14H). **ESI-MS** *m/z calcd* for $C_{17}H_{29}NOS$ $[M + H]^+$ 296.2043 *found* 296.2049.

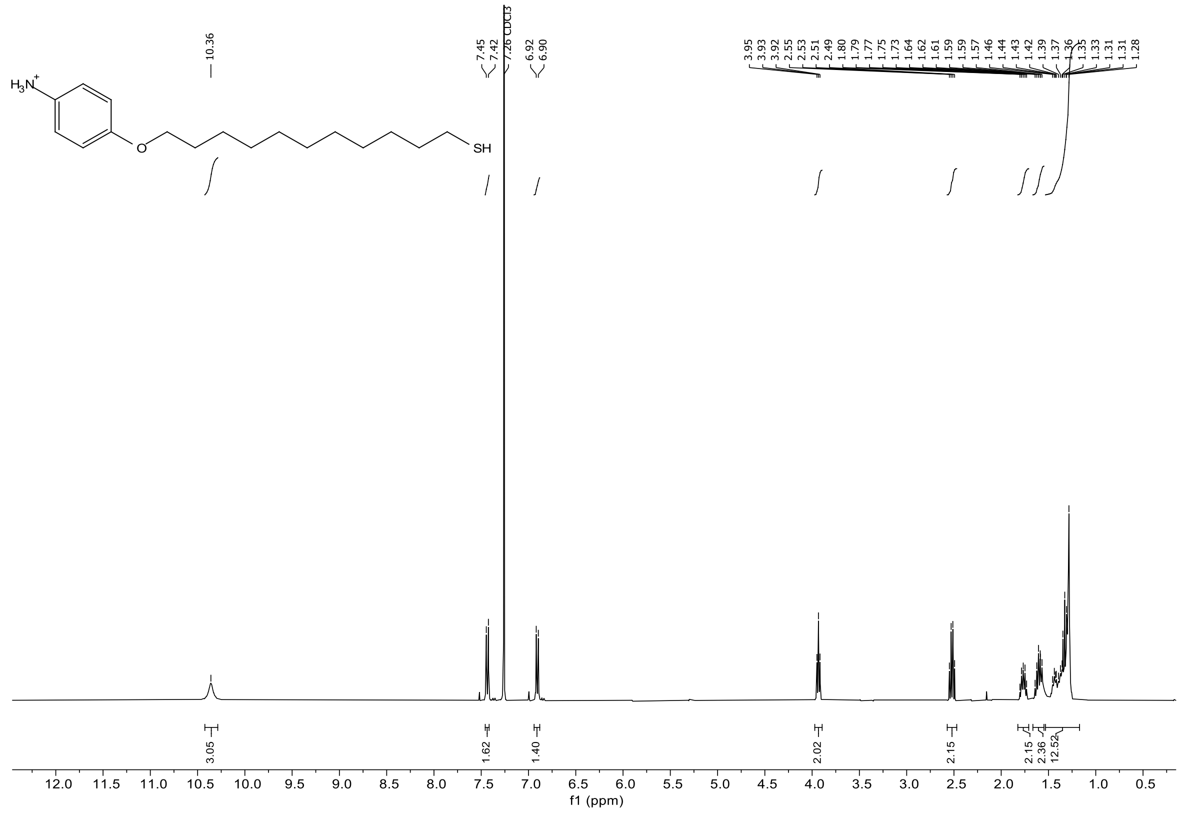

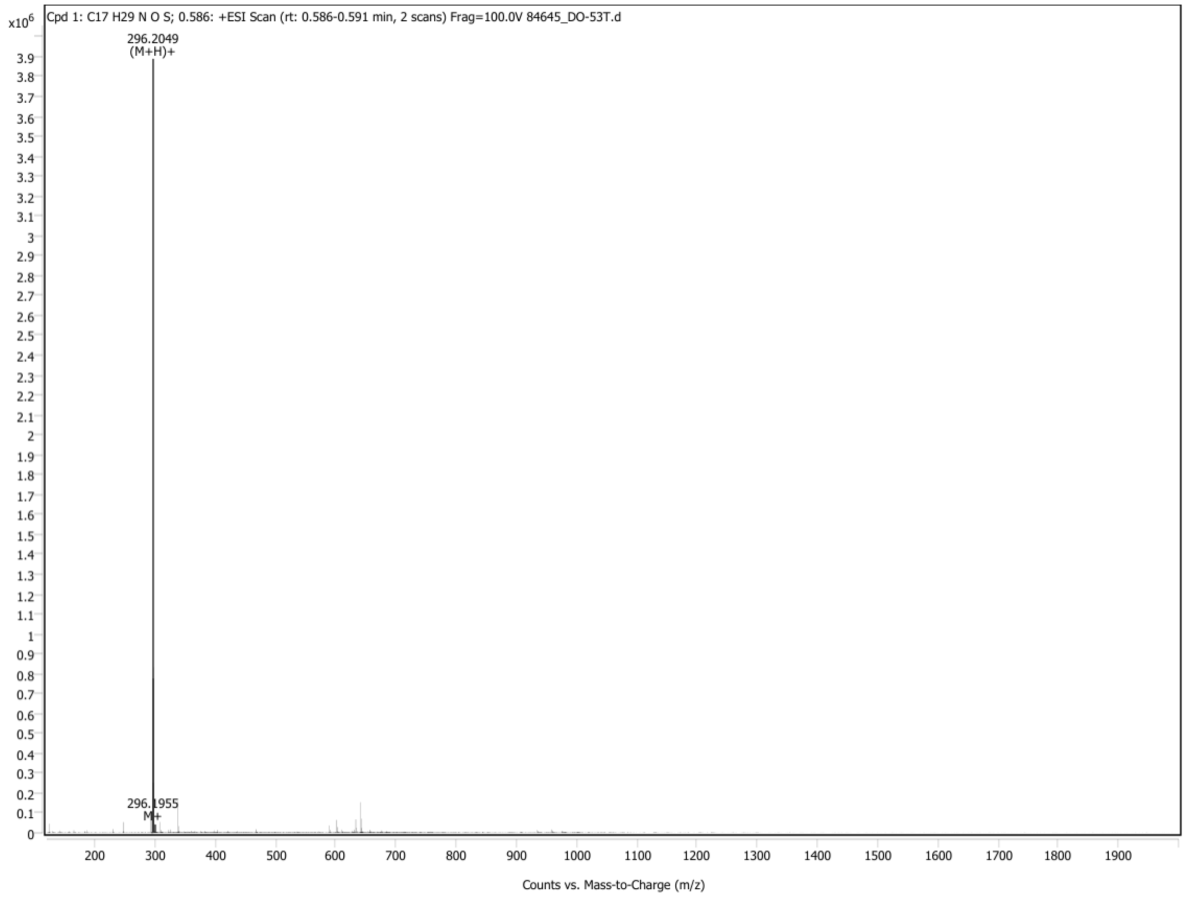


S-(11-(4-((2,2,10,10-Tetramethyl-4,8-dioxo-3,9-dioxa-5,7 diazaundecan-6-ylidene)amino)phenoxy)undecyl) ethanethioate **S11**

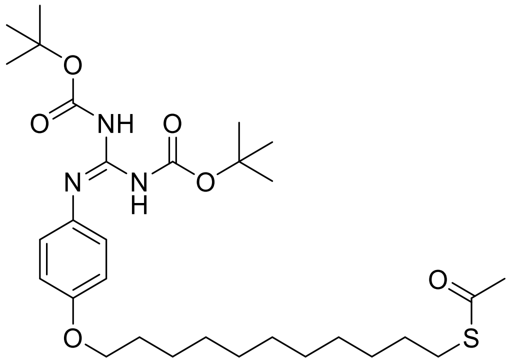

Amine **S10** (0.033 g, 0.098 mmol),) N,N'-bis(tert-butoxycarbonyl)-N''-triflylguanidine (0.044 g, 0.112 mmol) and $Et_3N$ (0.013 g, 0.126 mmol) were dissolved in anhydrous DCM (2.4 mL) under $N_2$ and purged for 15 mins. The solution was stirred at RT for 40 hrs. The resulting solution was diluted with DCM (100 mL) washed with 2 M aq. $NaHSO_4$ (1 × 15 mL), and washed further with sat. aq. $NaHCO_3$ (3 × 15 mL), $H_2O$ (15 mL), and sat. brine (15 mL). The organic phase was dried ($MgSO_4$), and the solvent removed *in vacuo.* The crude material was purified by silica column chromatography (EtOAc/hexanes 2.5% to 12.5%) giving the product **S11** as a white solid (0.035 g, 0.06 mmol, 62%). Observed analytical data matched that of the original work.[6] **$^1$H NMR** (400 MHz, $CDCl_3$) δ 11.64 (s, 1H), 10.17 (s, 1H), 7.49–7.44 (m, 2H), 6.87–6.82 (m, 2H), 3.92 (t, *J* = 6.6 Hz, 2H), 2.86 (t, *J* = 7.3 Hz, 2H), 2.32 (s, 3H), 1.80–1.71 (m, 2H), 1.53 (s, 9H), 1.49 (s, 9H), 1.46–1.25 (m, 16H). **$^{13}$C NMR** (101 MHz, $CDCl_3$) δ 196.1, 163.7, 156.4, 153.6, 153.4, 129.6, 123.8, 114.7, 83.5, 79.4, 68.3, 30.6, 29.5, 29.5, 29.5, 29.4, 29.4, 29.2, 29.2, 29.1, 28.8, 28.2, 28.1, 26.0. **ESI-MS** *m/z calcd* for $C_{30}H_{49}N_3O_6S$ $[M + H]^+$ 580.3415 *found* 580.3450.

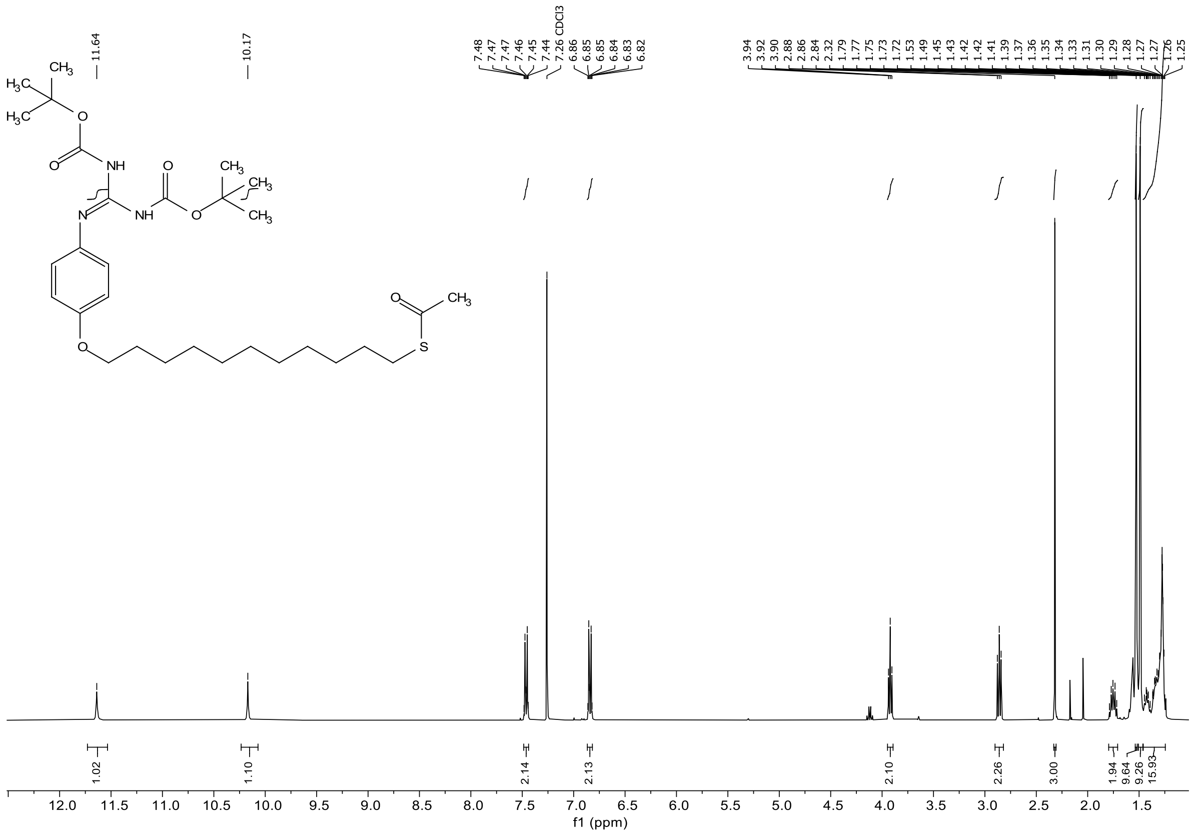

11.64
10.17
7.48 7.47 7.47 7.46 7.45 7.44 7.26 CDCl3 6.86 6.85 6.85 6.84 6.83 6.82
3.94 3.92 3.90 2.88 2.86 2.84 2.32 1.79 1.77 1.75 1.73 1.72 1.53 1.49 1.45 1.43 1.42 1.42 1.41 1.39 1.37 1.36 1.35 1.34 1.33 1.31 1.30 1.29 1.28 1.27 1.27 1.26 1.25
1.02
1.10
2.14
2.13
2.10
2.26
3.00
1.94
9.64
9.26
15.93
f1 (ppm)


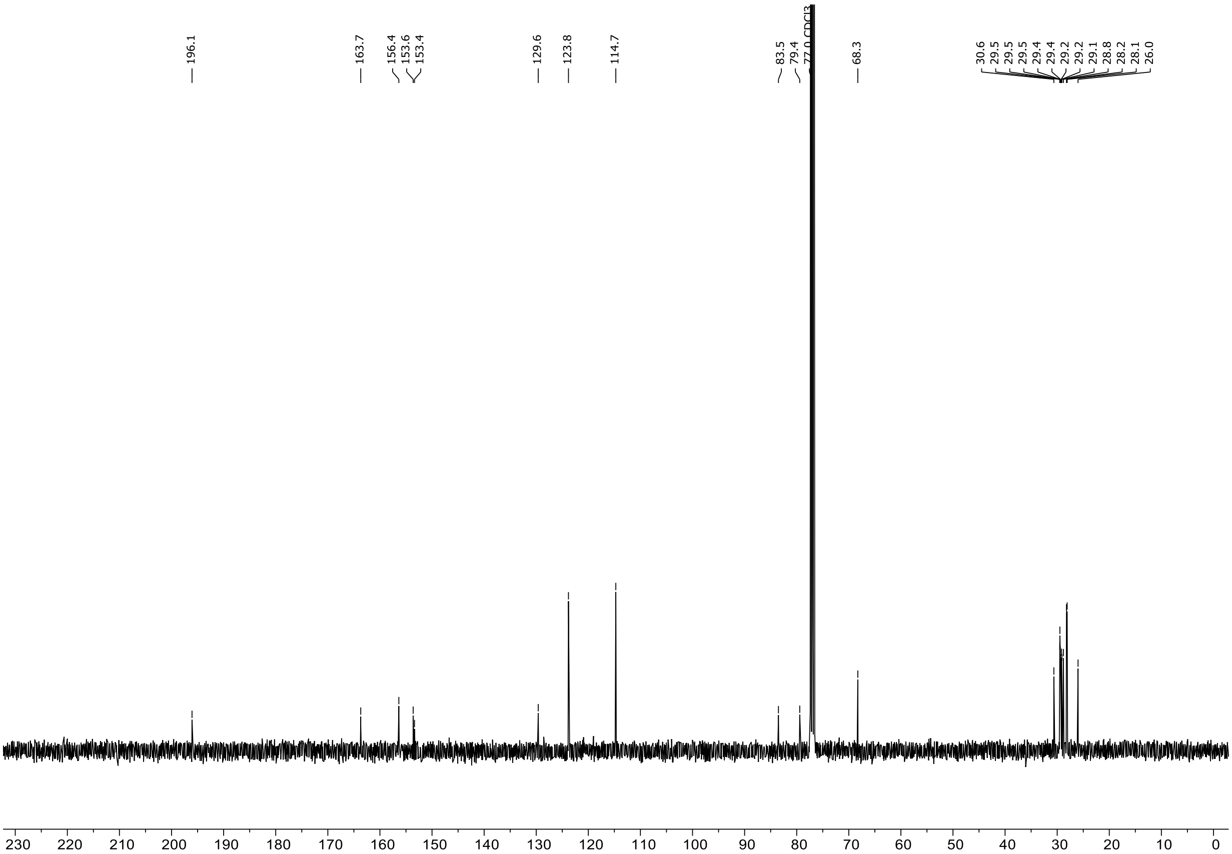

196.1
163.7
156.4 153.6 153.4
129.6
123.8
114.7
83.5 79.4 77.0 CDCl3
68.3
30.6 29.5 29.5 29.5 29.4 29.4 29.2 29.2 29.1 28.8 28.2 28.1 26.0
f1 (ppm)

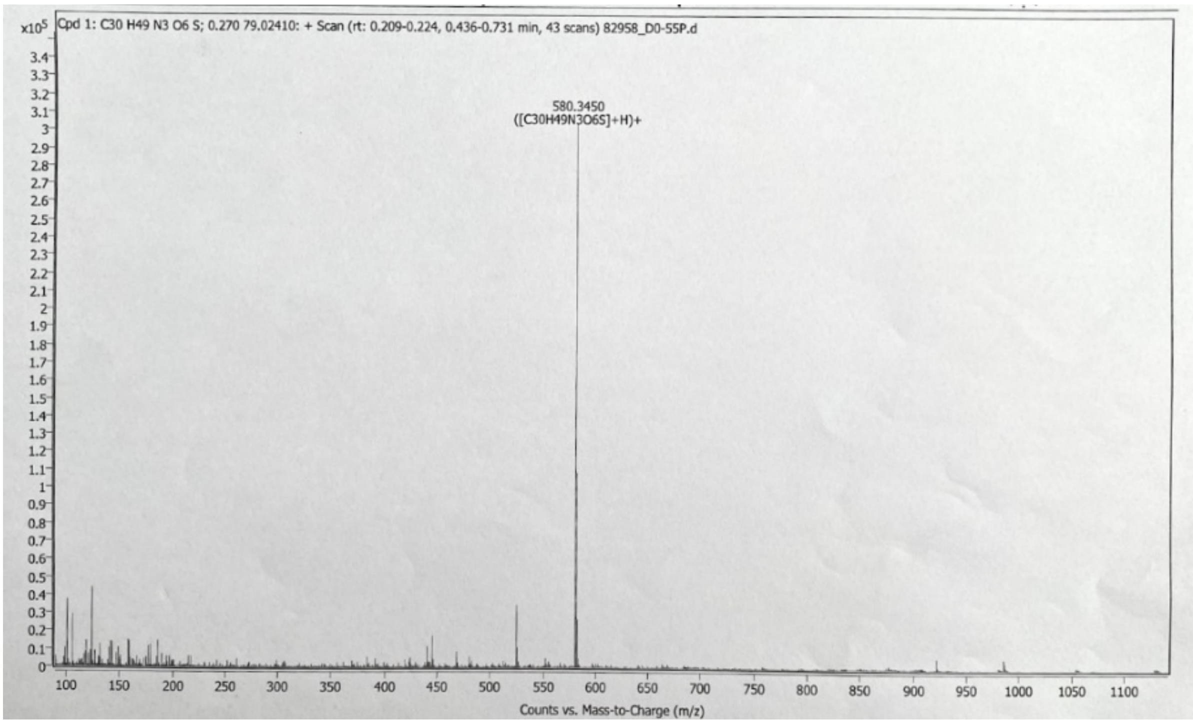


N-(diaminomethylene)-4-((11-mercaptoundecyl)oxy)benzenaminium chloride **6**

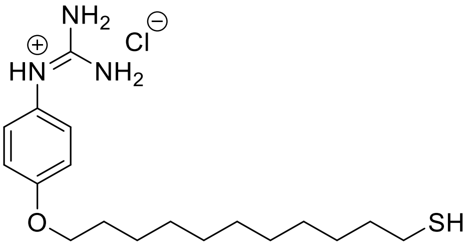

Protected guanidine **S11** was doubly deprotected using methanolic HCl (2M MeOH/$H_2O$ 10:1 v:v, >10 eq.), for 3 hrs at 55 °C. The solvent was evaporated, obtaining deprotected **6**. **$^1$H NMR** (400 MHz, MeOD) δ 7.35–7.13 (m, 2H), 7.09–6.96 (m, 2H), 4.00 (t, *J* = 6.4 Hz, 2H), 2.49 (t, 2H), 1.88–1.73 (m, 2H), 1.66–1.56 (m, 2H), 1.53–1.26 (m, 14H). **ESI-MS** *m/z calcd* for $C_{18}H_{32}N_3OS^+$ $[M]^+$ 338.2261 *found* 338.2258.

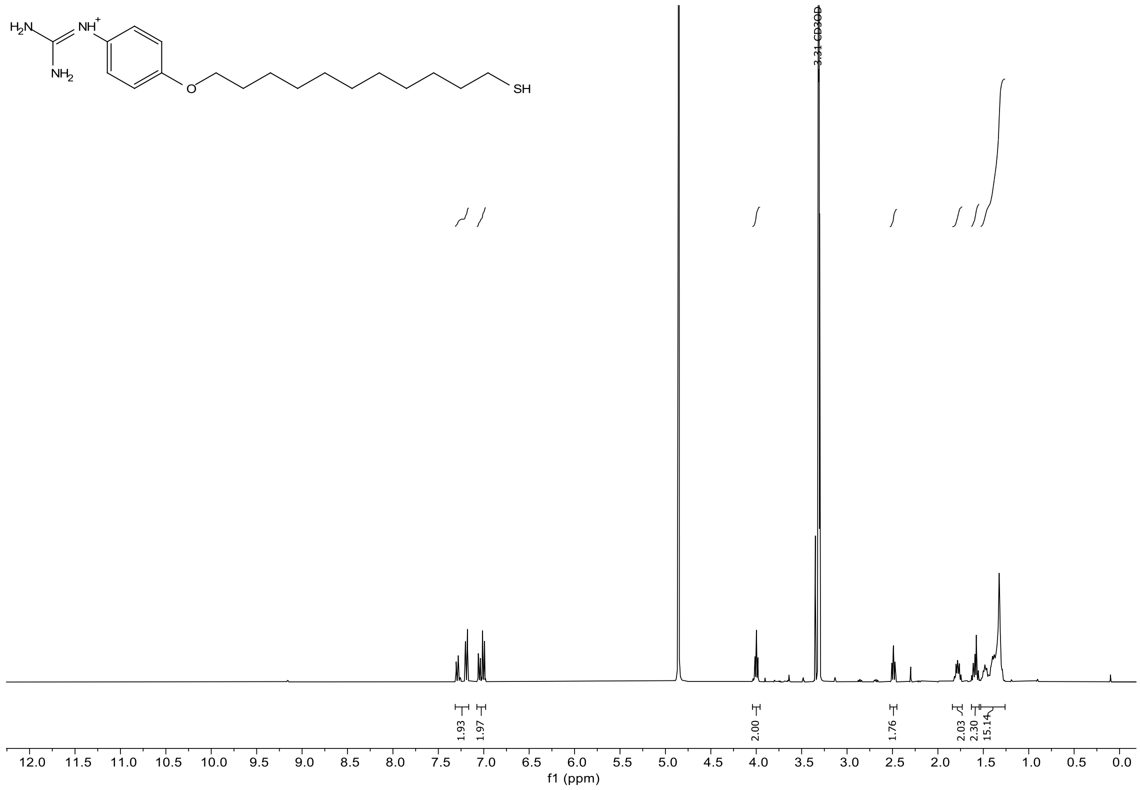

$H_2N$
$NH^+$
$NH_2$
O
SH
3.31 CD3OD
1.93
1.97
2.00
1.76
2.03
2.30
15.14
12.0 11.5 11.0 10.5 10.0 9.5 9.0 8.5 8.0 7.5 7.0 6.5 6.0 5.5 5.0 4.5 4.0 3.5 3.0 2.5 2.0 1.5 1.0 0.5 0.0
f1 (ppm)


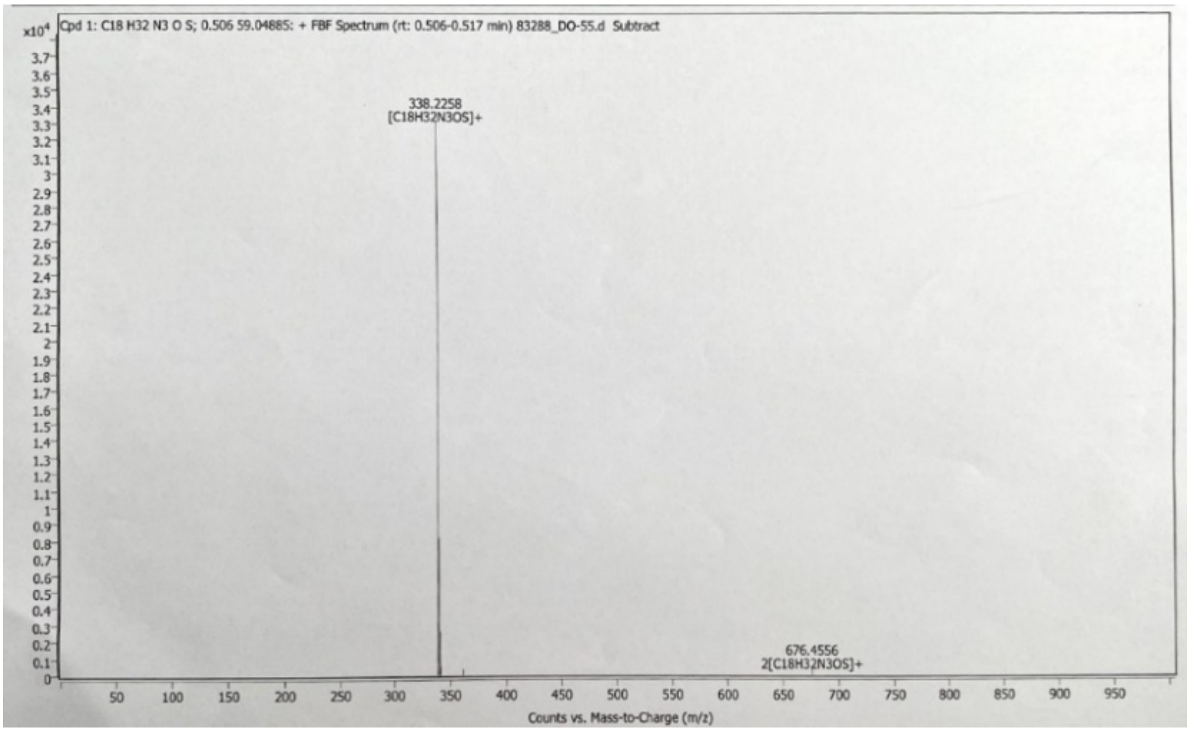

Cpd 1: C18 H32 N3 O S; 0.506 59.04885: + FBF Spectrum (rt: 0.506-0.517 min) 83288_DO-55.d Subtract
x10^4
338.2258
[C18H32N3OS]+
676.4556
2[C18H32N3OS]+
Counts vs. Mass-to-Charge (m/z)

**Synthesis 'Neutral' (alk11- and -PEG4-alk11-) thioester and thiol 8–10**

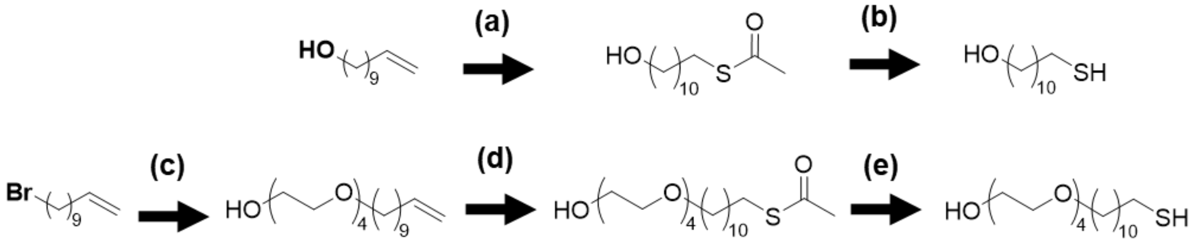

(a)Thioacetic acid, AIBN, anhydrous THF, $N_2$, 24h, 70 °C, 65% (b) 1.25 M HCl in MeOH, 3h, 55 °C, quantitative (c) PEG4, NaOH, anhydrous dioxane, $N_2$, 16h, 100 °C, 70% (d) Thioacetic acid, AIBN, anhydrous THF, $N_2$, 16h, 70 °C, 75% (e) HCl /MeOH (1.25 M, > 10 eq.), 3h, 55 °C, quant.

S-(11-Hydroxyundecyl) ethanethioate **S12**

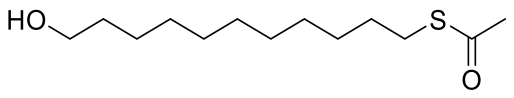

10-Undecen-1-ol (1.00 g, 5.87 mmol) and AIBN (0.386 g, 2.50 mmol) were dissolved in anhydrous THF (25 mL at RT) and sparged with $N_2$ for 15 mins. Thioacetic acid (1.79 g, 23.49 mmol) was then added to the reaction and heated to 75 °C for 16 hrs under $N_2$. The solvent was removed *in vacuo* and the resulting mixture was redissolved in DCM (30 mL) and washed with sat. aq. $NaHCO_3$ (3 × 30 mL), $H_2O$ (30 mL), and sat. brine (30 mL). The organic phase was dried ($MgSO_4$), and the solvent removed *in vacuo*. The compound was purified by silica column chromatography (0–5% MeOH/DCM) yielding the product **S12** as an orange waxy solid (0.951 g, 3.86 mmol, 65%). **$^1$H NMR** (400 MHz, $CDCl_3$) δ 3.63 (t, $J$ = 6.6 Hz, 2H), 2.85 (t, $J$ = 7.4 Hz, 2H), 2.31 (s, 3H), 1.61–1.49 (m, 4H), 1.47–1.22 (m, 14H). **$^{13}$C NMR** (101 MHz, $CDCl_3$) δ 196.1, 63.0, 32.8, 30.6, 29.5, 29.5, 29.4, 29.4, 29.4, 29.1, 29.1, 28.8, 25.7. **ESI-MS** *m/z calcd* for $C_{13}H_{26}O_2S$ $[M + H]^+$ 247.1726 *found* 247.1727.

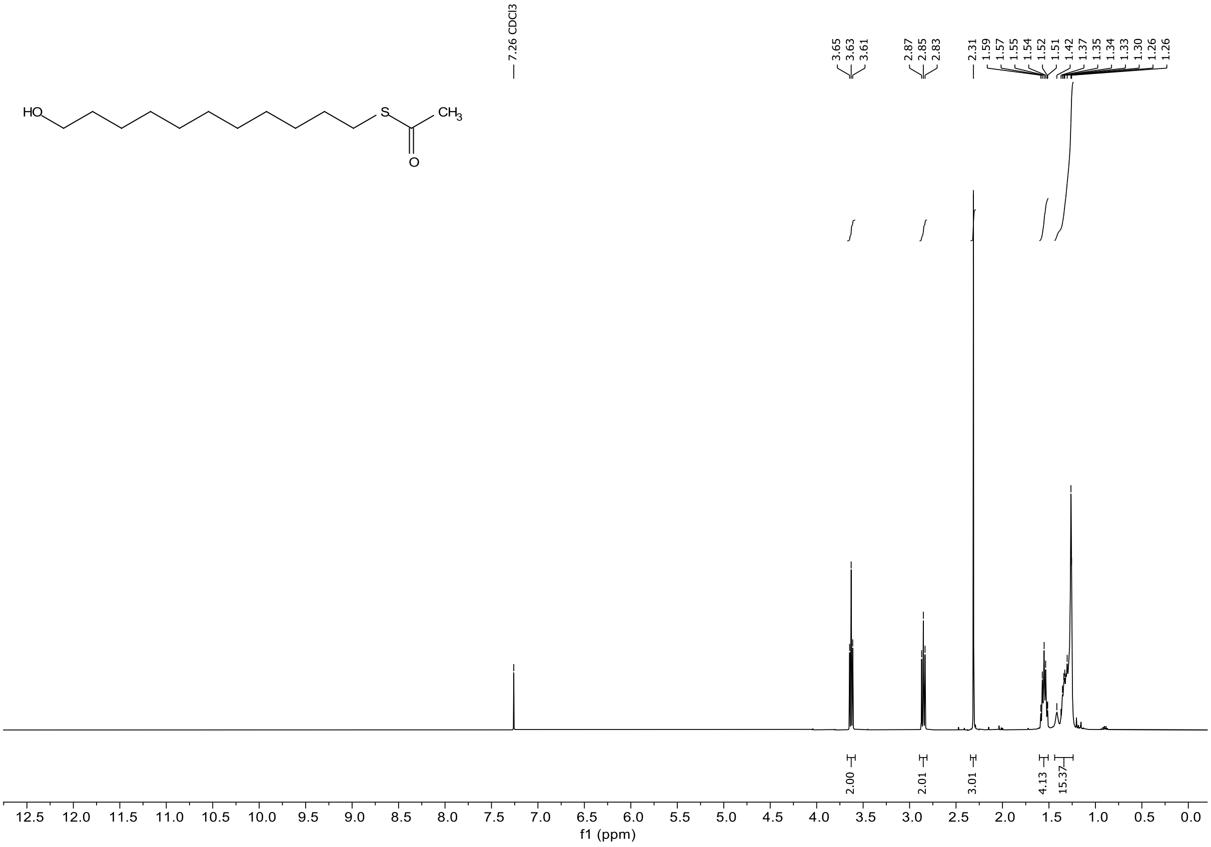

HO
S
CH3
O
7.26 CDCl3
3.65
3.63
3.61
2.87
2.85
2.83
2.31
1.59
1.57
1.55
1.54
1.52
1.51
1.42
1.37
1.35
1.34
1.33
1.30
1.26
1.26
2.00
2.01
3.01
4.13
15.37
12.5 12.0 11.5 11.0 10.5 10.0 9.5 9.0 8.5 8.0 7.5 7.0 6.5 6.0 5.5 5.0 4.5 4.0 3.5 3.0 2.5 2.0 1.5 1.0 0.5 0.0
f1 (ppm)


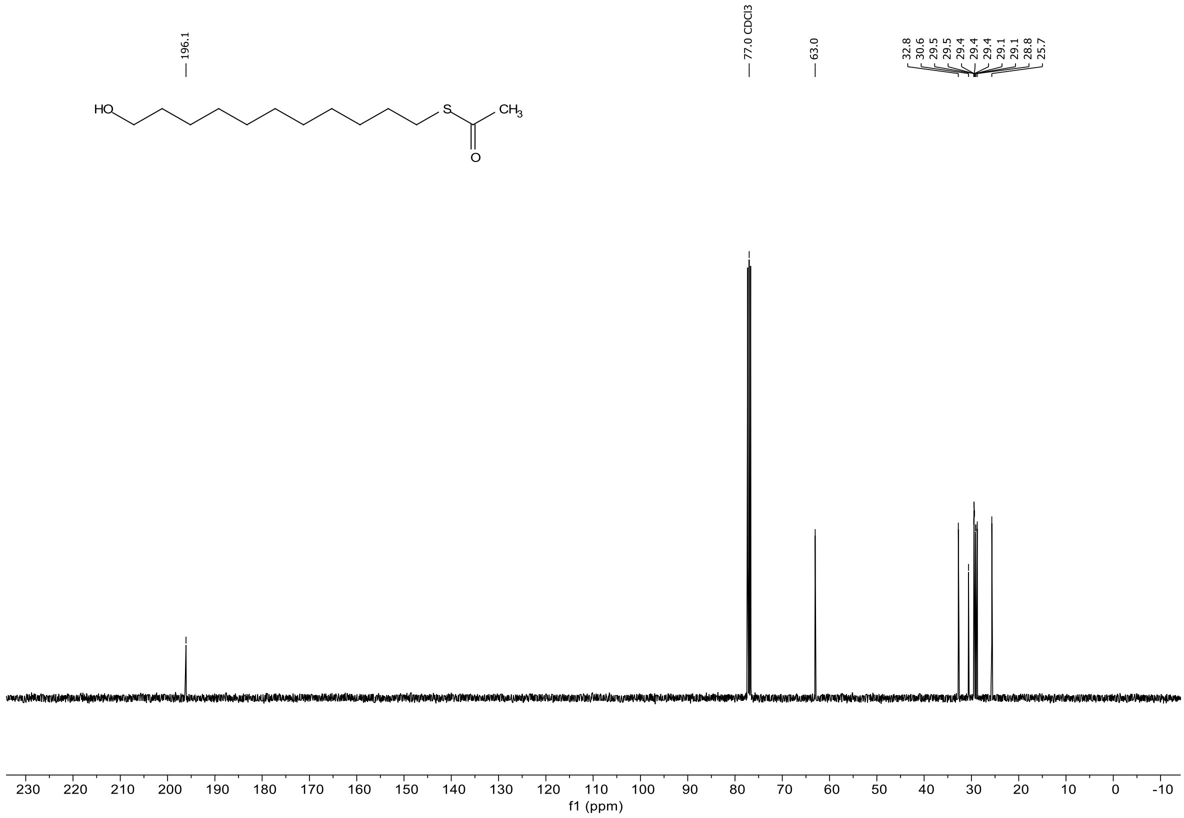

HO
S
CH3
O
196.1
77.0 CDCl3
63.0
32.8
30.6
29.5
29.5
29.4
29.4
29.4
29.1
29.1
28.8
25.7
230 220 210 200 190 180 170 160 150 140 130 120 110 100 90 80 70 60 50 40 30 20 10 0 -10
f1 (ppm)

*Compound Spectra (overlaid)*

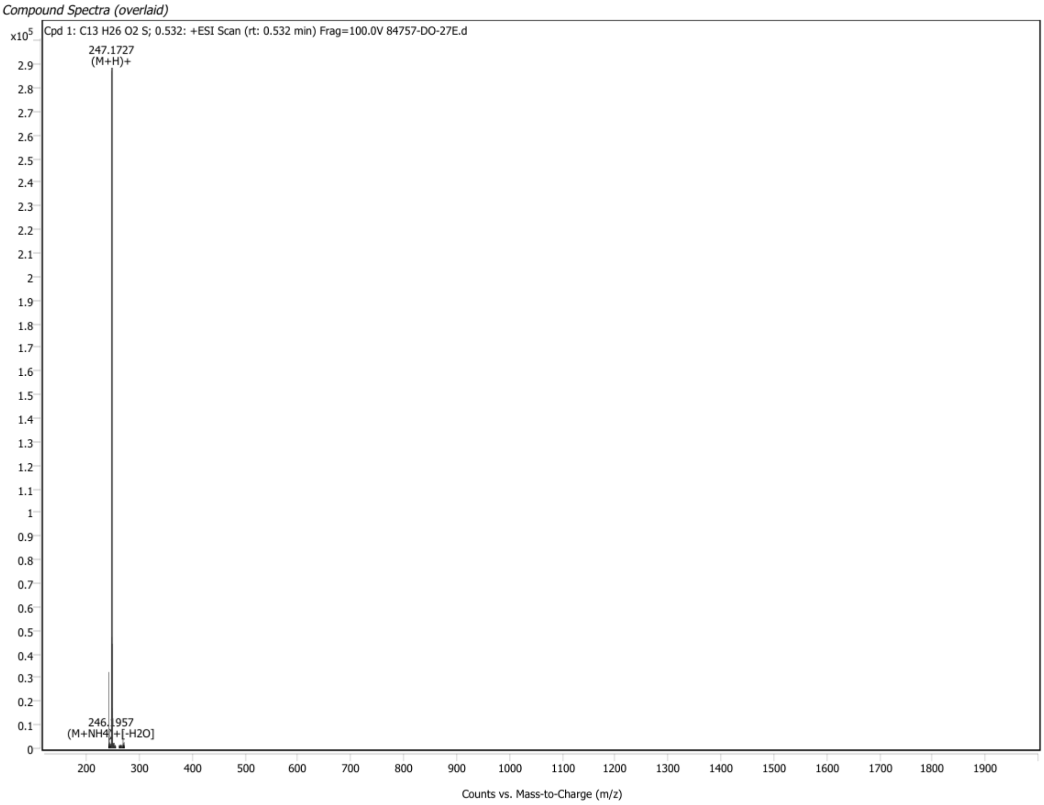

11-Mercaptoundecan-1-ol **8**

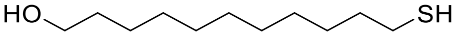

Thioester was deprotected using methanolic HCl (1.25M, >10 eq.), for 3 hrs at 55 °C. The solvent was evaporated, obtaining the thiolated compound **8**. **$^1$H NMR** (400 MHz, $CDCl_3$) δ 3.64 (t, $J$ = 6.6 Hz, 2H), 2.57–2.48 (m, 2H), 1.71–1.49 (m, 4H), 1.41–1.19 (m, 14H). **ESI-MS** *m/z calcd* for $C_{11}H_{24}OS$ [M + H]$^+$ 205.1621 *found* 205.1613.

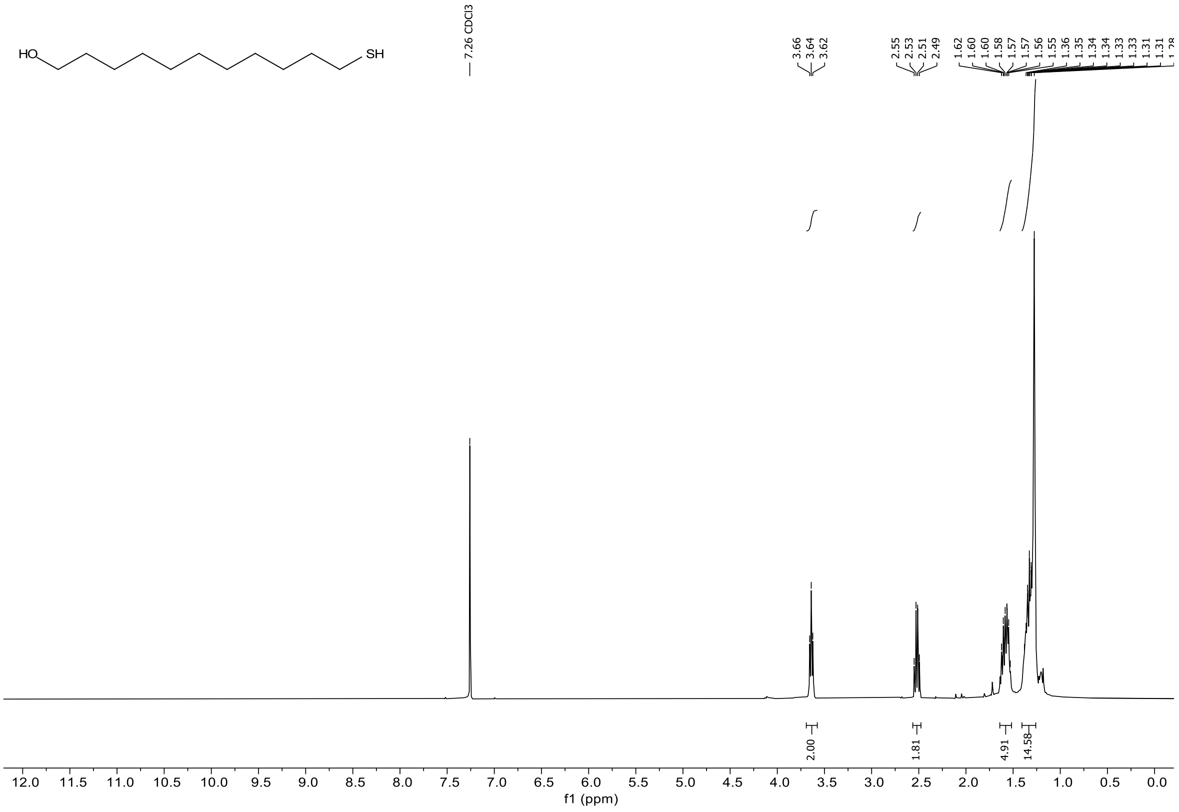

3,6,9,12-Tetraoxatricos-22-en-1-ol **S13**

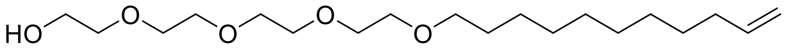


Tetraethylene glycol (13.63 mL, 78.6 mmol) and NaOH (0.63 g, 15.72 mmol) were stirred under an atmosphere of $N_2$, for 30 mins at 100 °C. 11-Bromoundec-1-ene (3.67 g, 15.74 mmol), was added dropwise to the resulting mixture and stirred for a further 24 hrs. The reaction was quenched with sat. aq. $NH_4Cl$ (90 mL) and stirred for 1 hr. The mixture was extracted in EtOAc (100 mL) and washed with sat. aq. $NH_4Cl$ (3 × 30 mL), $H_2O$ (30 mL) and sat. brine wash (30 mL). The organic layer was then dried ($MgSO_4$) and dried *in vacuo*. The compound was further purified by silica column chromatography (20–80% EtOAc/pet. ether), yielding a **S13** as a pale yellow oil (3.828 g, 11.05 mmol, 70%). Observed analytical data matched that of the original work.[7] **$^1$H NMR** (400 MHz, $CDCl_3$) δ 5.88–5.74 (m, 1H), 5.04–4.88 (m, 2H), 3.77–3.55 (m, 16H), 3.45 (t, $J$ = 6.8 Hz, 2H), 2.46 (t, $J$ = 6.3 Hz, 1H), 2.09–1.98 (m, 2H), 1.62–1.49 (m, 2H), 1.43–1.23 (m, 12H). **ESI-MS** *m/z calcd* for $C_{19}H_{38}O_5$ $[M + NH_4]^+$ 364.3063 *found* 364.3062.

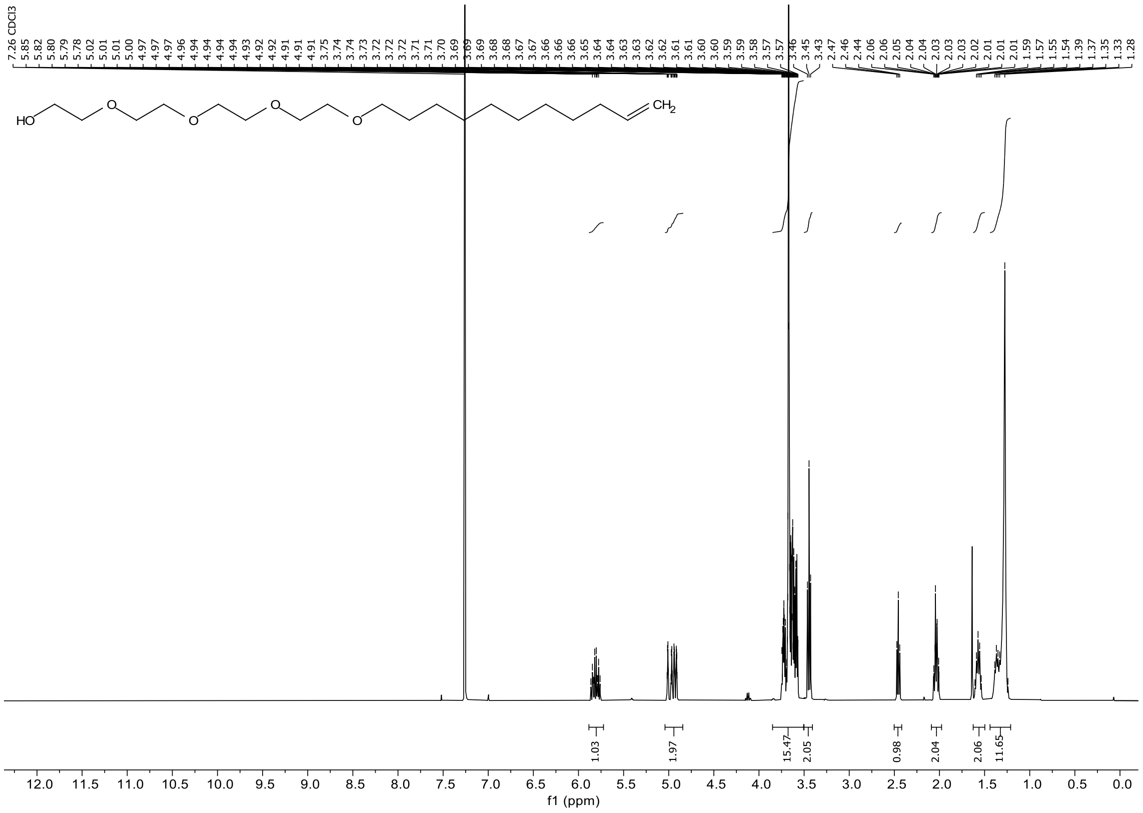

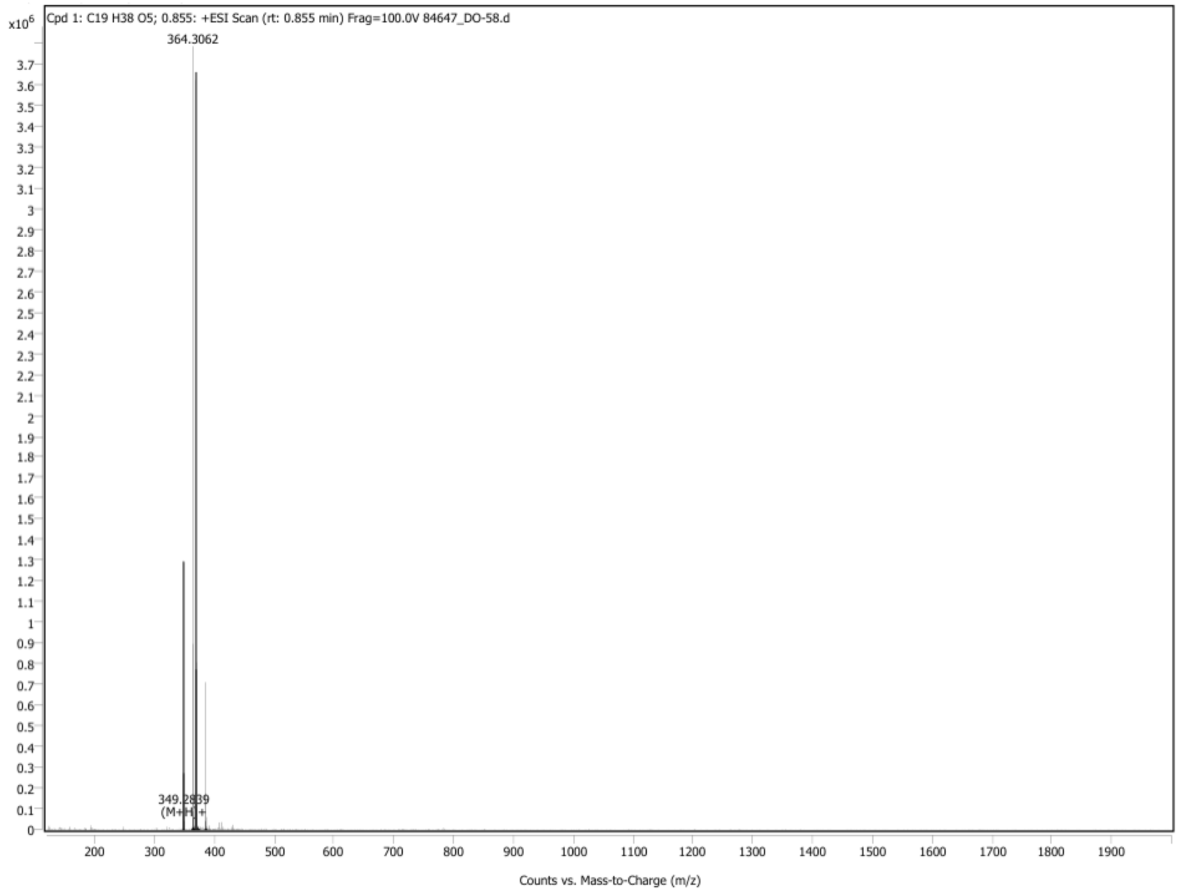


S-(1-hydroxy-3,6,9,12-tetraoxatricosan-23-yl) ethanethioate **S14**

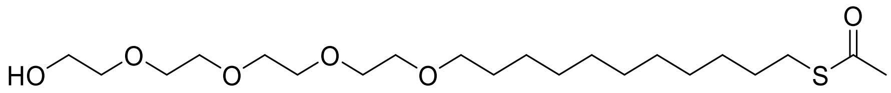


To a solution of alkene **S13** (3.67 g, 10.59 mmol) in anhydrous dioxane (100 mL) was added AIBN (0.869 g, 3.296 mmol). The mixture was sparged with $N_2$ for 10 mins, then thioacetic acid (7.57 mL, 105.91 mmol) was added. The solution was heated at 70 °C overnight under N2 and then concentrated in *vacuo*. The residue was dissolved in DCM (50 mL) and washed with brine (3 x 15 mL), dried with $MgSO_4$, and dried. The product was purified by silica column chromatography (5–25% Acetone/hexanes) to give a yellow oil **S14** (3.429 g, 8.11 mmol, 77%). Observed analytical data matched that of the original work.[7] **$^1$H NMR** (400 MHz, $CDCl_3$) δ 3.78–3.57 (m, 16H), 3.47 (t, *J* = 6.8 Hz, 2H), 2.88 (t, *J* = 7.3 Hz, 2H), 2.35 (s, 3H), 1.61–1.53 (m, 4H), 1.30 (d, *J* = 15.0 Hz, 14H). **$^{13}$C NMR** (101 MHz, $CDCl_3$) δ 195.9, 72.4, 71.4, 70.5, 70.5, 70.5, 70.3, 69.9, 61.6, 30.5, 29.5, 29.4, 29.4, 29.3, 29.3, 29.0, 29.0, 28.7, 26.0 (2 signals missing, presumed overlapping). **ESI-MS** *m/z calcd* for $C_{21}H_{42}O_6S$ [M + Na]$^+$ 445.2594 *found* 445.2629.

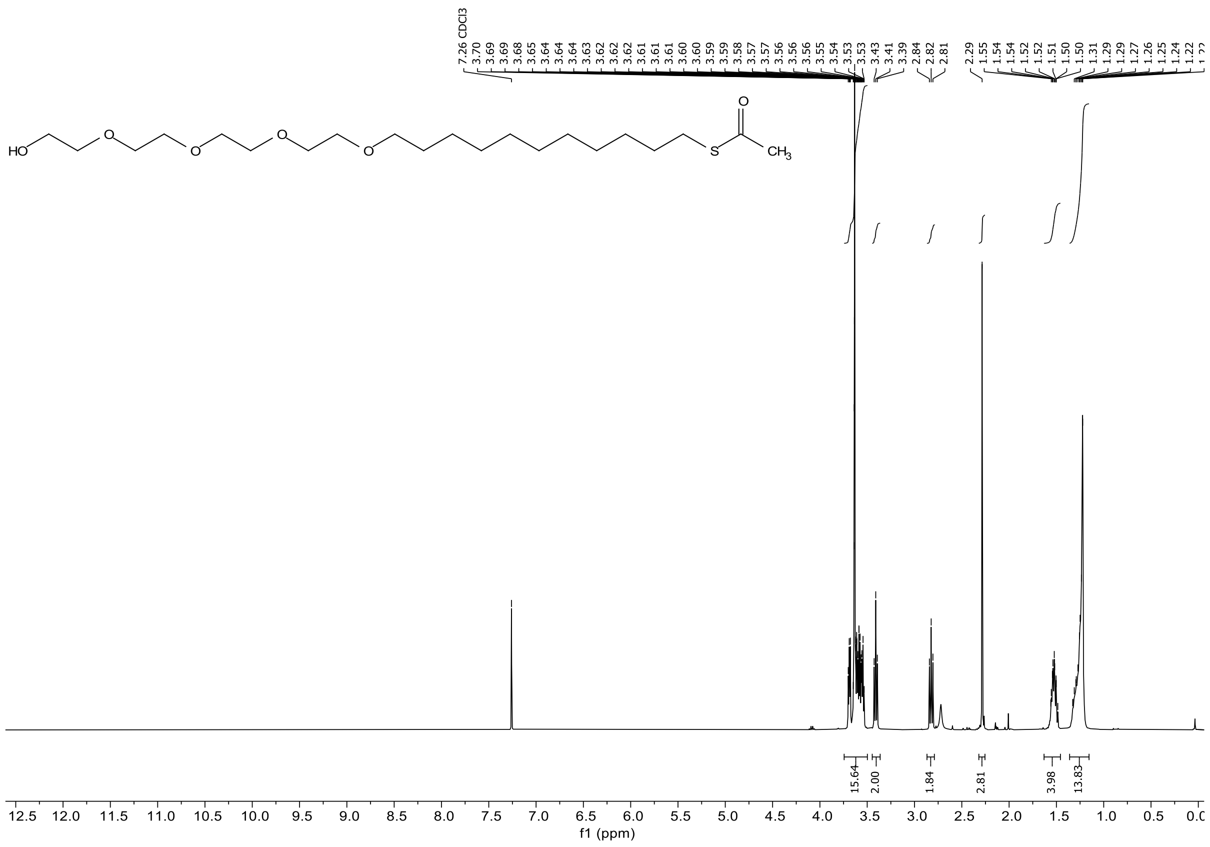
7.26 CDCl3
3.70
3.69
3.69
3.68
3.65
3.64
3.64
3.63
3.62
3.62
3.61
3.61
3.61
3.60
3.60
3.59
3.59
3.58
3.57
3.57
3.56
3.56
3.55
3.54
3.53
3.53
3.43
3.41
3.39
2.84
2.82
2.81
2.29
1.55
1.54
1.54
1.52
1.52
1.51
1.50
1.50
1.31
1.29
1.27
1.26
1.25
1.24
1.22
HO O O O O S O CH3
15.64
2.00
1.84
2.81
3.98
13.83
12.5 12.0 11.5 11.0 10.5 10.0 9.5 9.0 8.5 8.0 7.5 7.0 6.5 6.0 5.5 5.0 4.5 4.0 3.5 3.0 2.5 2.0 1.5 1.0 0.5
f1 (ppm)

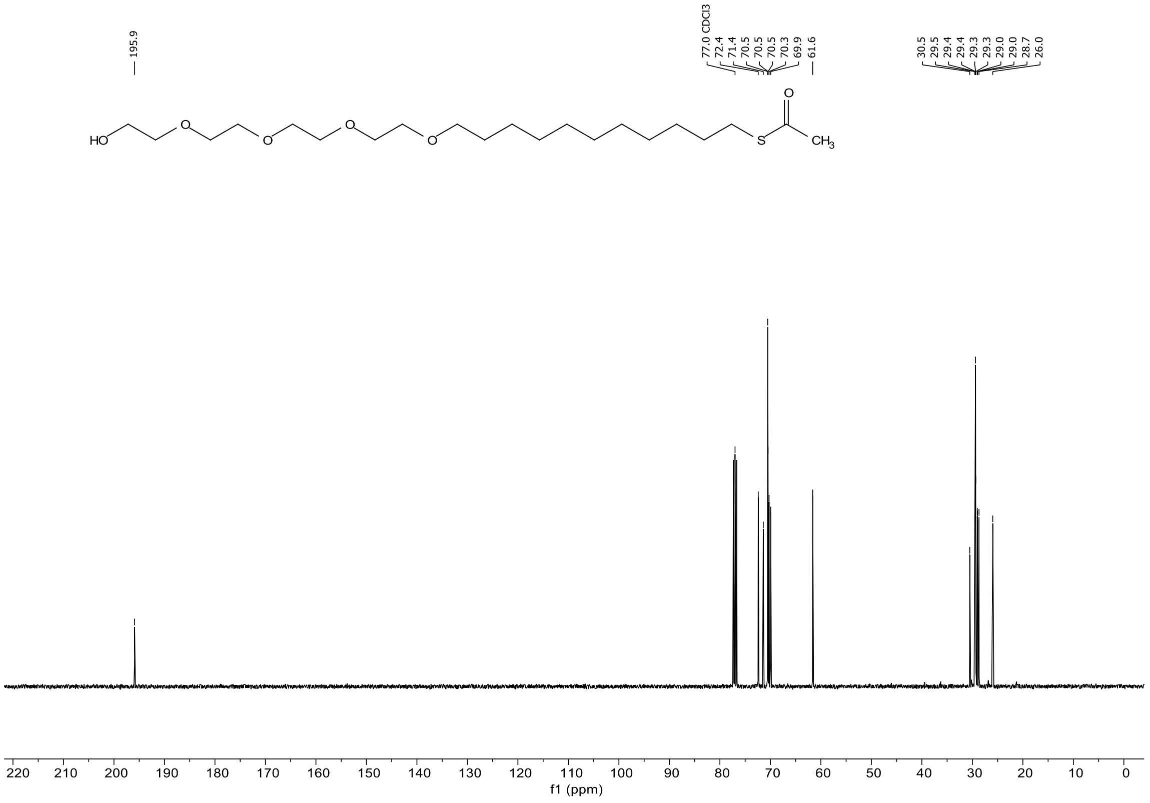
195.9
77.0 CDCl3
72.4
71.4
70.5
70.5
70.5
70.3
69.9
61.6
30.5
29.5
29.4
29.3
29.3
29.0
29.0
28.7
26.0
HO O O O O S O CH3
220 210 200 190 180 170 160 150 140 130 120 110 100 90 80 70 60 50 40 30 20 10 0
f1 (ppm)

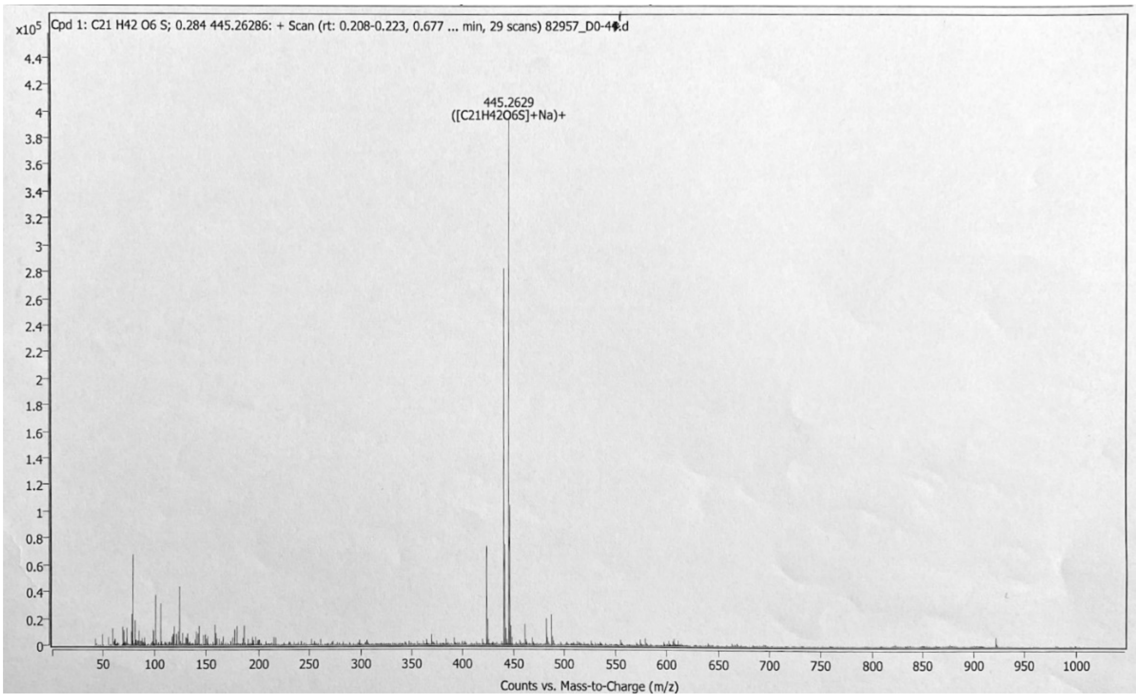


23-Mercapto-3,6,9,12-tetraoxatricosan-1-ol **9**

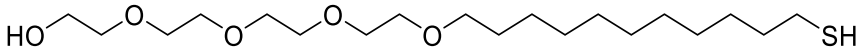

Thioester **S14** was deprotected using methanolic HCl (1.25M, >10 eq.), for 3 hrs at 55 °C. The solvent was evaporated *in vacuo*, obtaining the thiolated compound **9**.**$^1$H NMR** (400 MHz, $CDCl_3$) δ 3.79–3.55 (m, 16H), 3.45 (t, *J* = 6.8 Hz, 2H), 2.52 (app.q, *J* = 7.5 Hz, 2H), 1.63–1.51 (m, 4H), 1.46–1.21 (m, 16H). **ESI-MS** *m/z calcd* for $C_{19}H_{40}O_5S$ $[M + Na]^+$ 403.2489 *found* 403.2491.

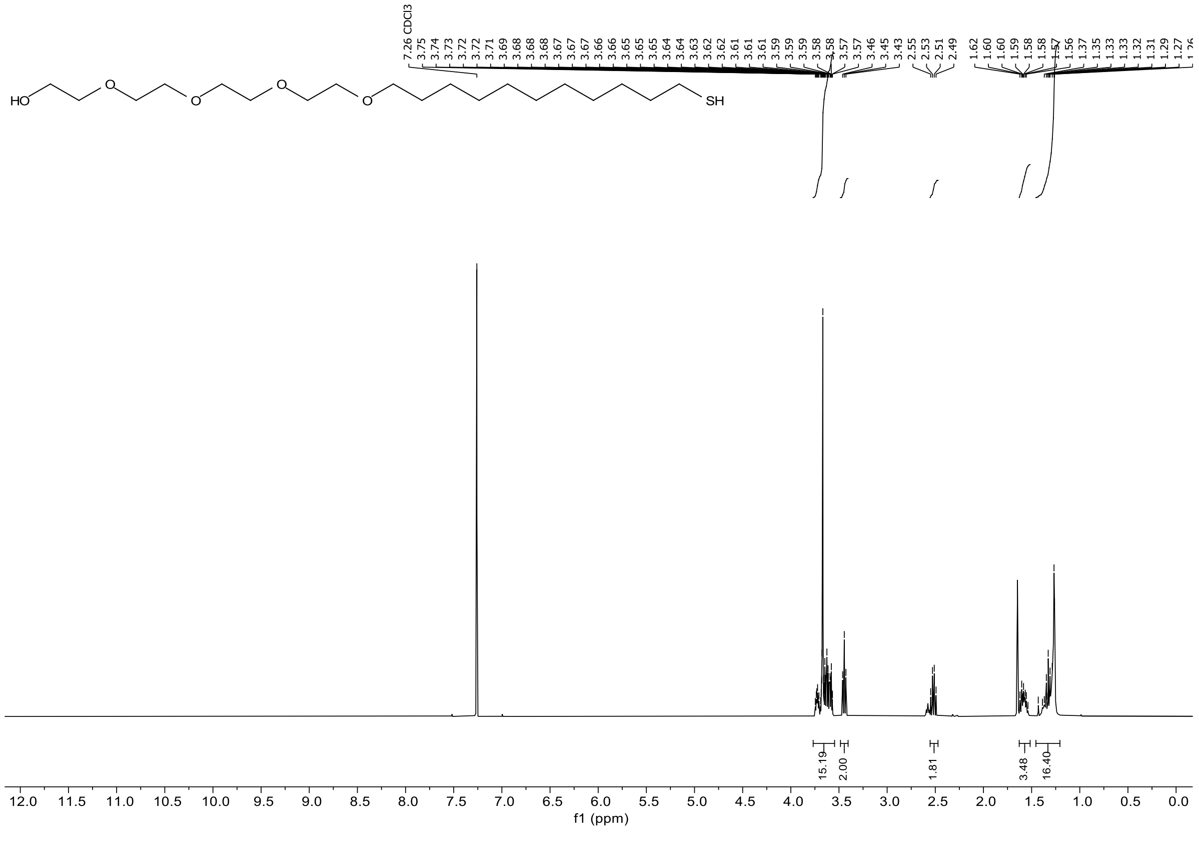

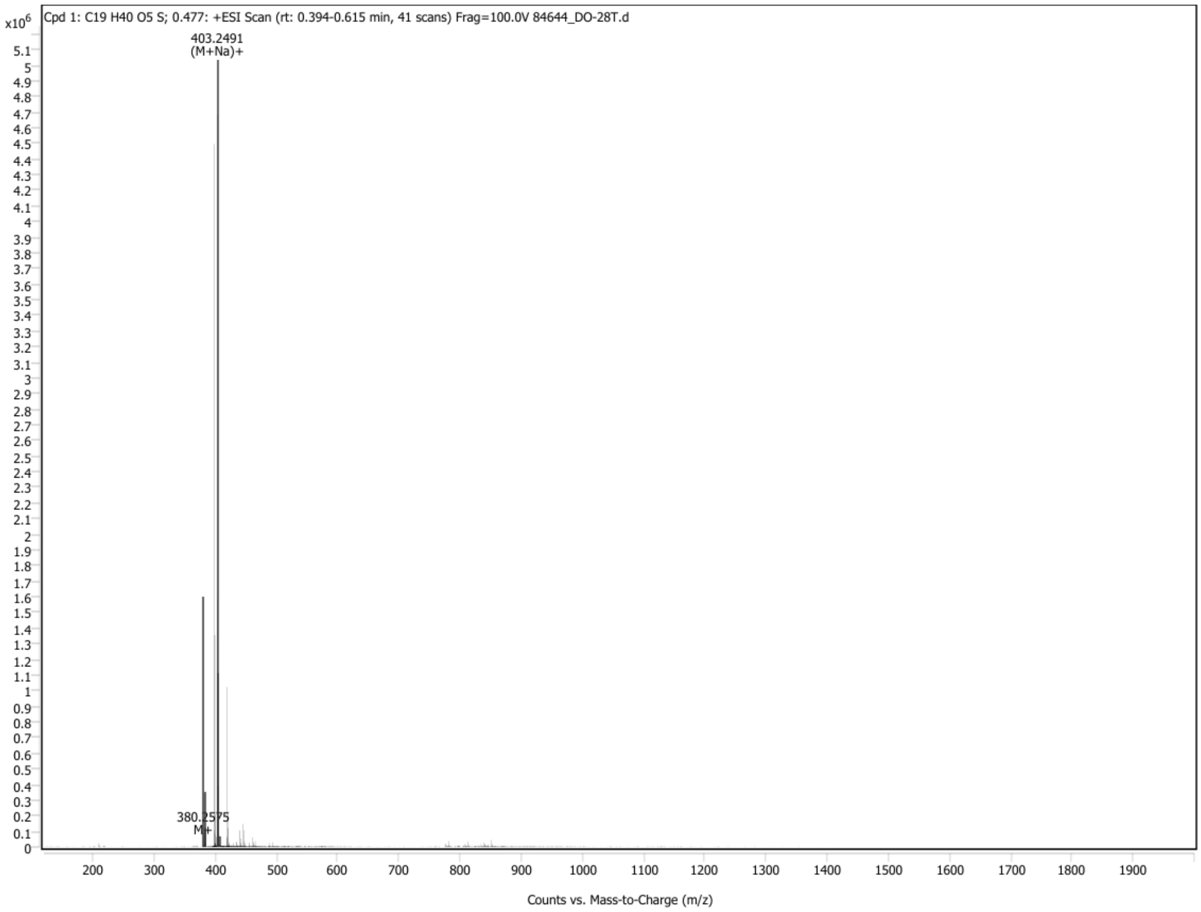


O-(5-(1,2-Dithiolan-3-yl)pentanoate)polyethylene glycol **10**

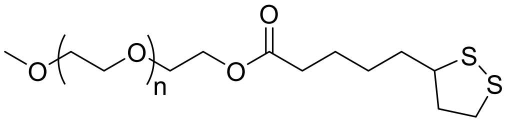

Based on a procedure from Uyeda *et al.*[8] $PEG_{750}$ (18.0 g, 24.1 mmol), lipoic acid (0.50 g, 2.41 mmol) and DMAP (89 mg, 0.7 mmol) were dissolved in DCM (15 mL) and cooled to 0 °C. Dicyclohexylcarbodiimide (DCC, 0.50 g, 2.41 mmol) in DCM (3 mL) was added dropwise and the reaction was stirred for 1 hr before being warmed to RT. The reaction was left stirring for 72 h, then the mixture was filtered through a celite plug and the solvent was removed *in vacuo.* The residue partitioned with sat. $NaHCO_3$ (100 mL) and EtOAc (100 mL). The organic layer was dried over $MgSO_4$ and evaporated before purification *via* silica gel chromatography (15:1 chloroform/MeOH) to yield **10** as a yellow oil (1.047 g, 1.13 mmol, 46%). Purification was performed rapidly, as the desired product was found to form an insoluble gel over time. The product oil was stored in 1 mL of chloroform for stability (to avoid gelation). Prior to use on a gold surface an aliquot was taken and 10 eq. of TCEP was added to cleave the disulfide. Observed analytical data matched that of the original work. **$^1$H NMR** (400 MHz, $CDCl_3$) δ 4.23–4.20 (m, 2H), 3.72–3.60 (m, 72H), 3.57–3.52 (m, 4H), 3.37 (s, 3H), 3.22–3.06 (m, 2H), 2.51–2.41 (m, 1H), 2.34 (t, $J$ = 7.4 Hz, 2H), 1.96–1.85 (m, 2H), 1.75–1.59 (m, 4H), 1.55–1.39 (m, 2H). **ESI-MS** *m/z calcd* for $C_{41}H_{80}O_{18}S_2$ $[M + Na]^+$ 947.4679 *found* 947.4691.

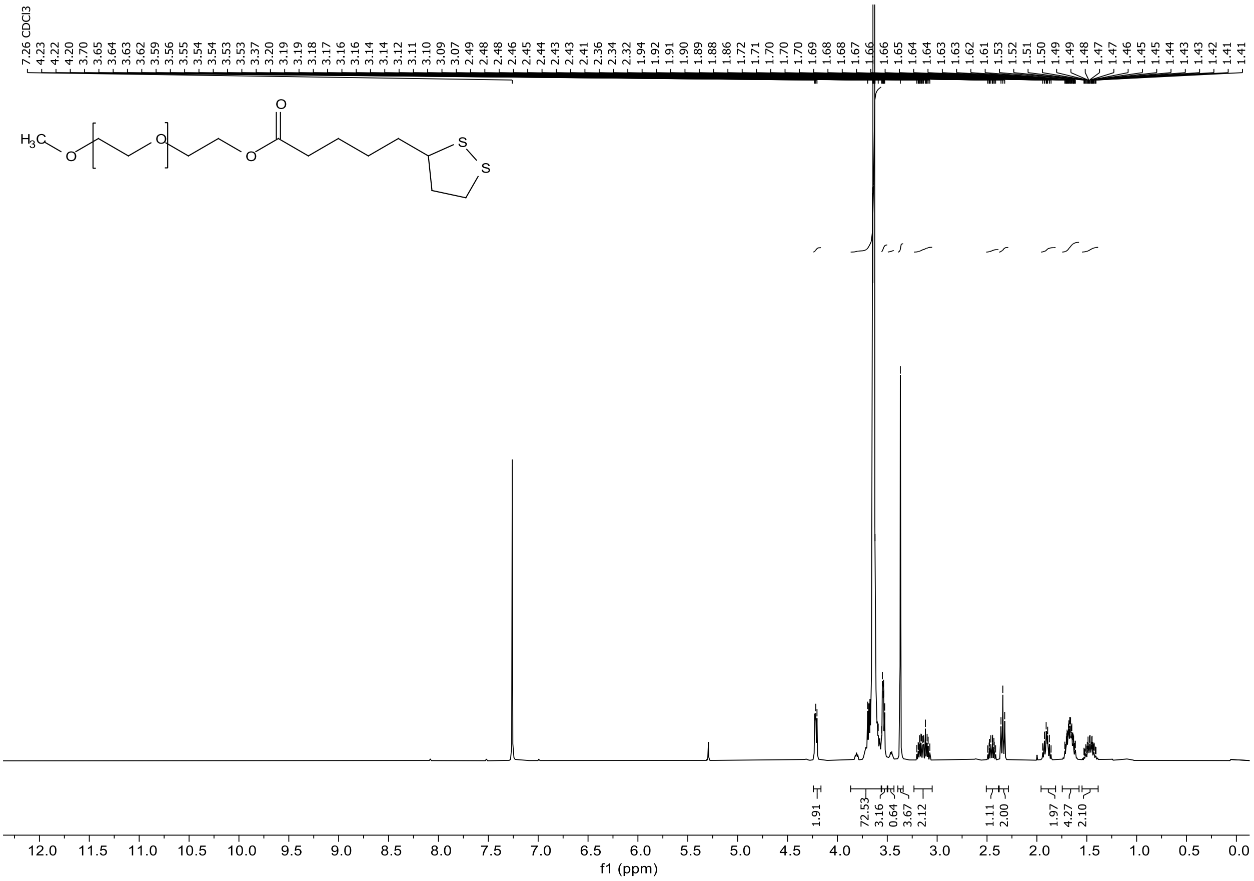
7.26 CDCl3
4.23 4.22 4.20 3.70 3.65 3.64 3.63 3.62 3.59 3.56 3.55 3.54 3.54 3.53 3.53 3.37 3.20 3.19 3.19 3.18 3.17 3.16 3.16 3.14 3.14 3.12 3.11 3.10 3.09 3.07 2.49 2.48 2.48 2.46 2.45 2.44 2.43 2.43 2.41 2.36 2.34 2.32 1.94 1.92 1.91 1.90 1.89 1.88 1.86 1.72 1.71 1.70 1.70 1.69 1.68 1.68 1.67 1.66 1.66 1.65 1.64 1.64 1.63 1.63 1.62 1.61 1.53 1.52 1.51 1.50 1.49 1.48 1.47 1.47 1.46 1.45 1.45 1.44 1.43 1.43 1.42 1.41 1.41
1.91
72.53
3.16
0.64
3.67
2.12
1.11
2.00
1.97
4.27
2.10
f1 (ppm)

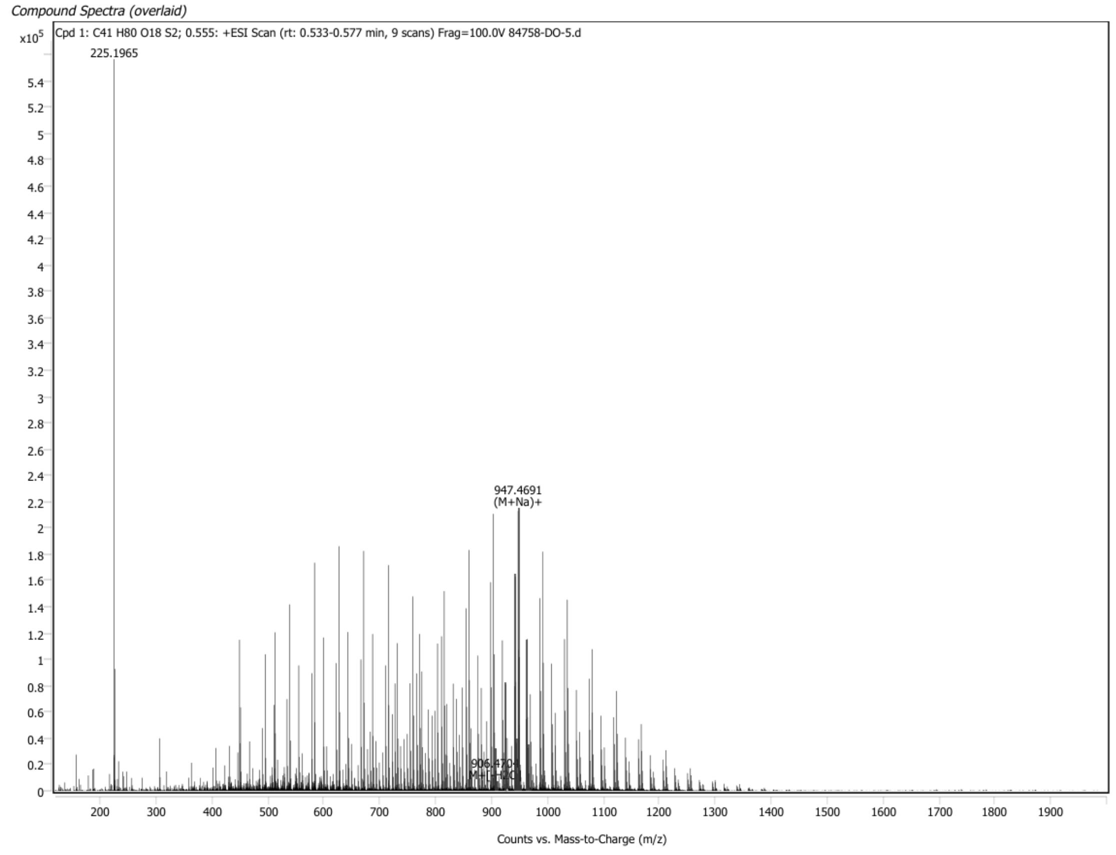
Compound Spectra (overlaid)
Cpd 1: C41 H80 O18 S2; 0.555: +ESI Scan (rt: 0.533-0.577 min, 9 scans) Frag=100.0V 84758-DO-5.d
x10^5
225.1965
947.4691
(M+Na)+
Counts vs. Mass-to-Charge (m/z)

### Synthesis of 'Negative' (-PEG4-alk11-) thiol and thioester 15 and 16

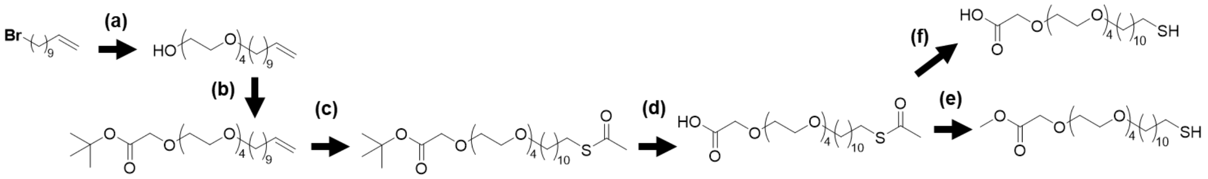

(a) PEG4, NaOH, anhydrous dioxane, $N_2$, 24h, 100 °C, 70% (b) *tert*-butyl bromoacetate, NaH, anhydrous DMF, $N_2$, 16h, RT, 78% (c) Thioacetic acid, AIBN, anhydrous toluene, $N_2$, 16h, 70 °C, 96% (d) 1:1 TFA/anhydrous DCM (0.2M, 30 eq. of TFA), 4h, RT, quant. (e) HCl /MeOH (1.25 M, > 10 eq.), 3h, 55 °C, quant. (f) NaOMe/ MeOH (4.2M, 1.1 eq.), $N_2$, 4h, 55 °C, quant.

Tert-butyl 3,6,9,12,15-pentaoxahexacos-25-enoate **S15**

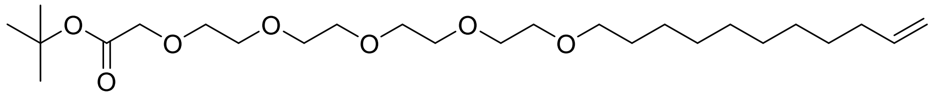

NaH (0.063 g, 1.59 mmol) was added portionwise to a solution of alcohol **S13** (0.5 g, 1.44 mmol) in anhydrous DMF (10 mL) at 0 °C. The mixture was stirred for 10 mins and tert-butyl bromoacetate (0.42 g, 2.16 mmol) was added dropwise. The mixture was stirred at RT for 16 hrs. The mixture was diluted with water (20 mL) and extracted with EtOAc (3 × 40 mL). The combined organic layers was washed with water (40 mL) and sat. brine (40 mL), dried ($MgSO_4$) and concentrated *in vacuo.* Purification by silica column chromatography yielded the product **S15** as a colourless oil (0.525 g, 1.14 mmol, 78 %). Observed analytical data matched that of the original work.[9] **$^1$H NMR** (400 MHz, $CDCl_3$) δ 5.81 (m, 1H), 5.04–4.88 (m, 2H), 4.02 (s, 2H), 3.73–3.56 (m, 16H), 3.44 (t, $J$ = 6.8 Hz, 2H), 2.07–2.00 (m, 2H), 1.47 (s, 9H), 1.43–1.22 (m, 14H).**$^{13}$C NMR** (101 MHz, $CDCl_3$) δ 169.8, 139.4, 114.2, 81.7, 71.7, 70.9, 70.8, 70.7, 70.2, 69.2, 34.0, 29.8, 29.7, 29.6, 29.6, 29.3, 29.1, 28.3, 28.1, 28.0, 26.2 (2 signals missing, presumed overlapping). **ESI-MS** *m/z calcd* for $C_{25}H_{48}O_7$ $[M + Na]^+$ 483.3293 *found* 483.3285.

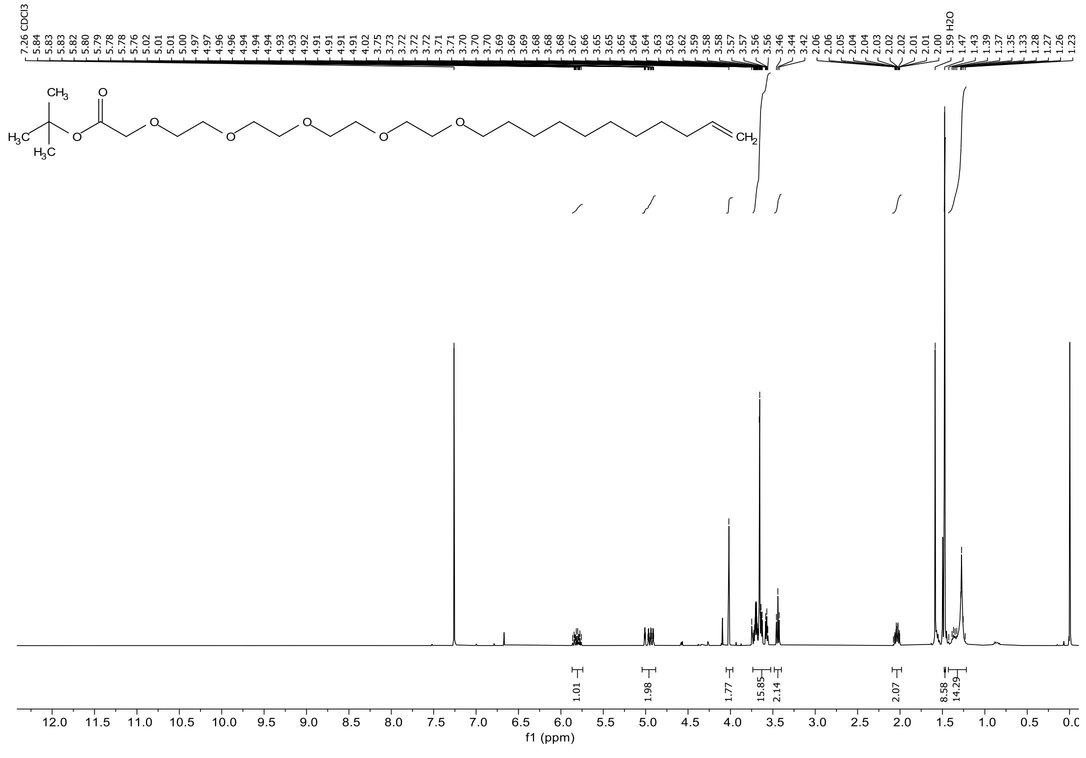

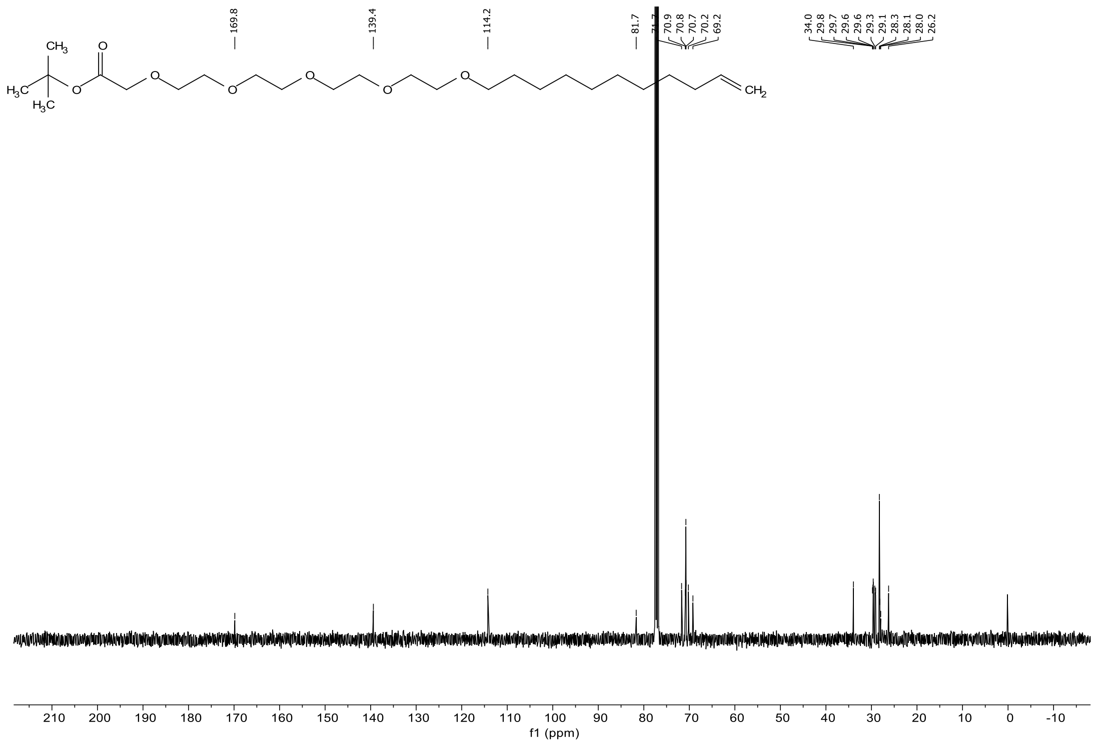


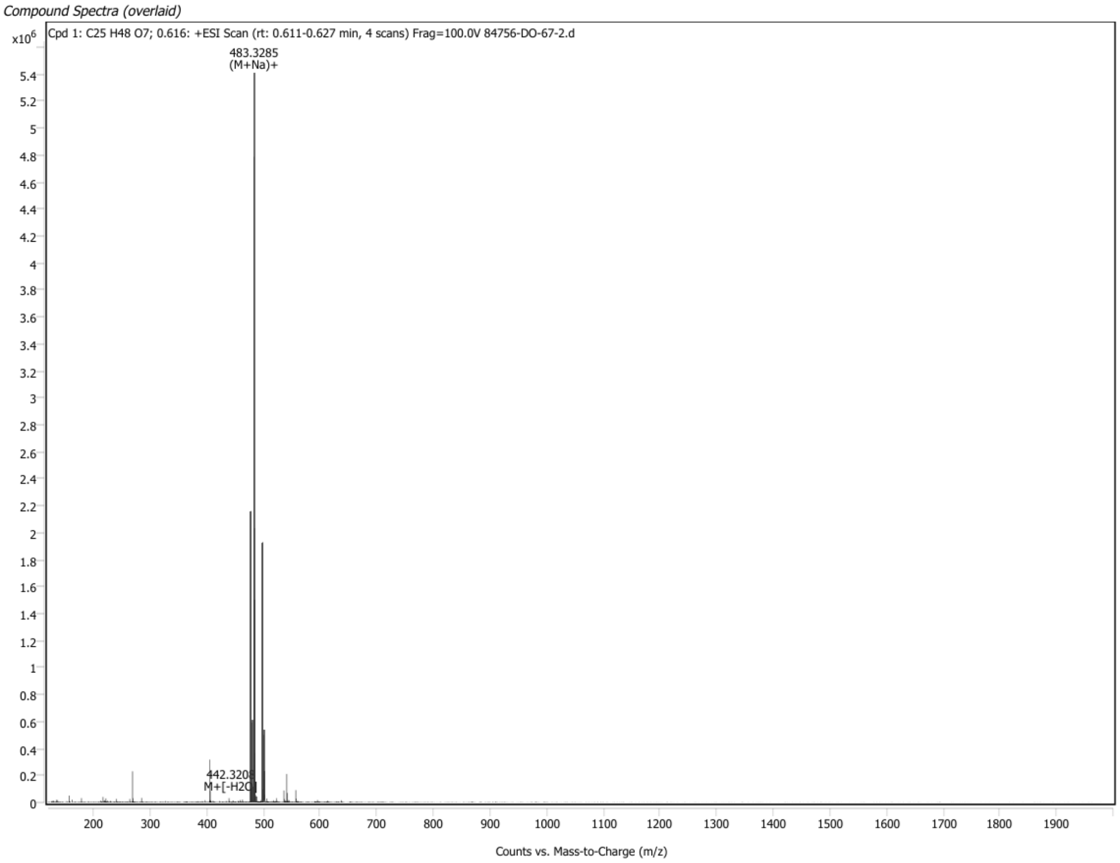


Tert-butyl 28-oxo-3,6,9,12,15-pentaoxa-27-thianonacosanoate **S16**

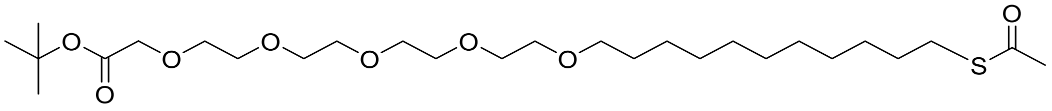

To a solution of alkene **S15** (0.400 g, 0.866 mmol) in anhydrous toluene (2.5 mL), was added AIBN (0.057 g, 0.346 mmol). The mixture was sparged with $N_2$ for 10 mins, then thioacetic acid (0.309 mL, 4.33 mmol) was added. The solution was heated to 70 °C overnight under $N_2$ and then concentrated in *vacuo*. The product **S16** was obtained as a yellow oil (0.445 g, 0.83 mmol, 96%). Observed analytical data matched that of the original work.[9] **$^1$H NMR** (400 MHz, CDCl3) δ 4.02 (s, 2H), 3.75–3.55 (m, 16H), 3.44 (t, J = 6.8 Hz, 2H), 2.90–2.82 (m, 2H), 2.32 (s, 3H), 1.47 (s, 9H), 1.45–1.17 (m, 14H).**$^{13}$C NMR** (101 MHz, $CDCl_3$) δ 196.0, 169.7, 81.8, 81.5, 71.5, 70.6, 70.6, 70.5, 70.0, 69.0, 68.4, 30.6, 29.6, 29.5, 29.4, 29.4, 29.4, 29.2, 29.1, 29.1, 28.8, 28.1, 28.0, 27.9, 26.0. **ESI-MS** *m/z calcd* for $C_{27}H_{52}O_8S$ $[M + Na]^+$ 559.3276 *found* 559.3264.

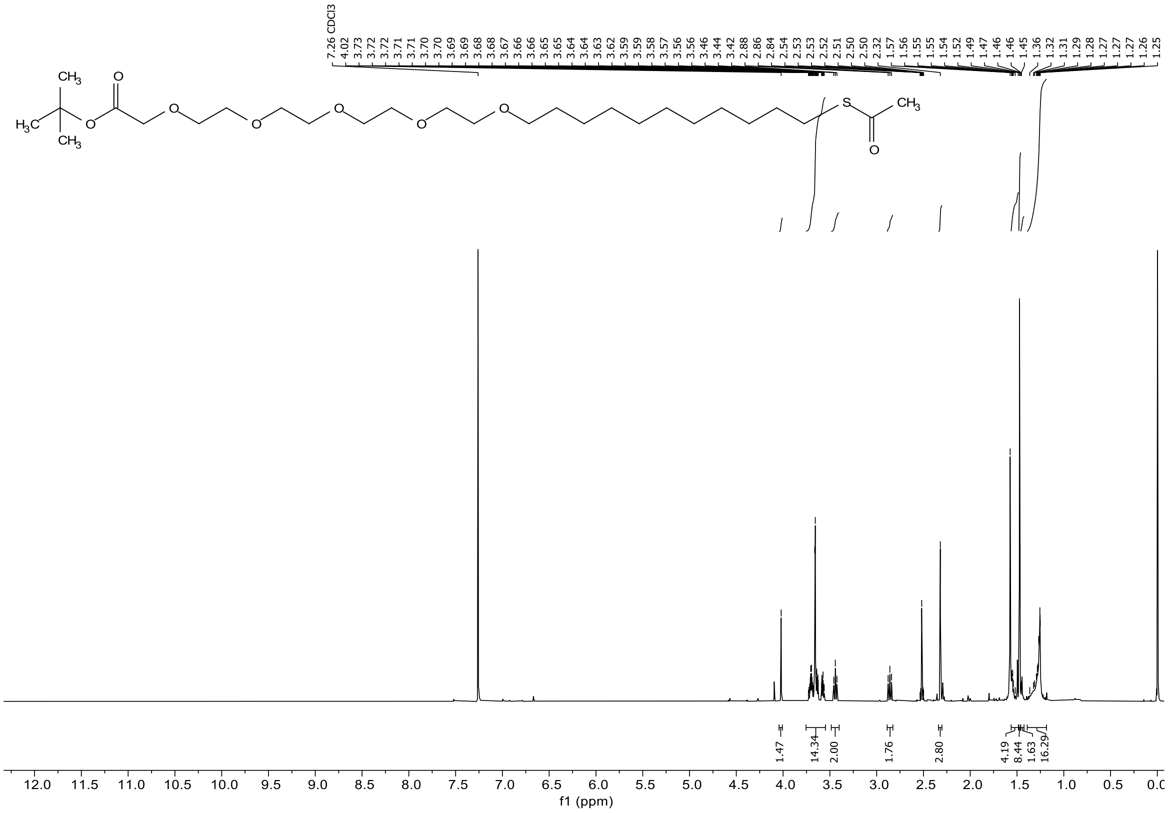

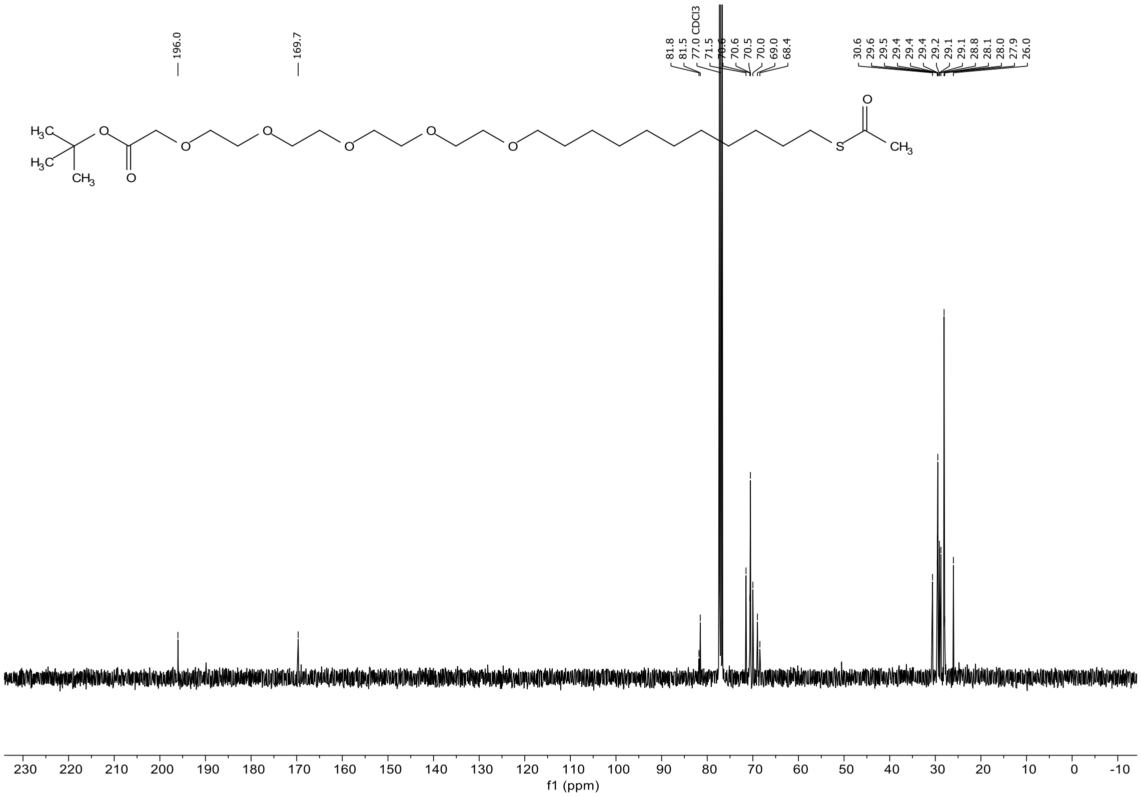
196.0
169.7
81.8
81.5
77.0 CDCl3
71.5
70.6
70.6
70.5
70.0
69.0
68.4
30.6
29.6
29.5
29.4
29.4
29.4
29.2
29.1
29.1
28.8
28.1
28.0
27.9
26.0
f1 (ppm)

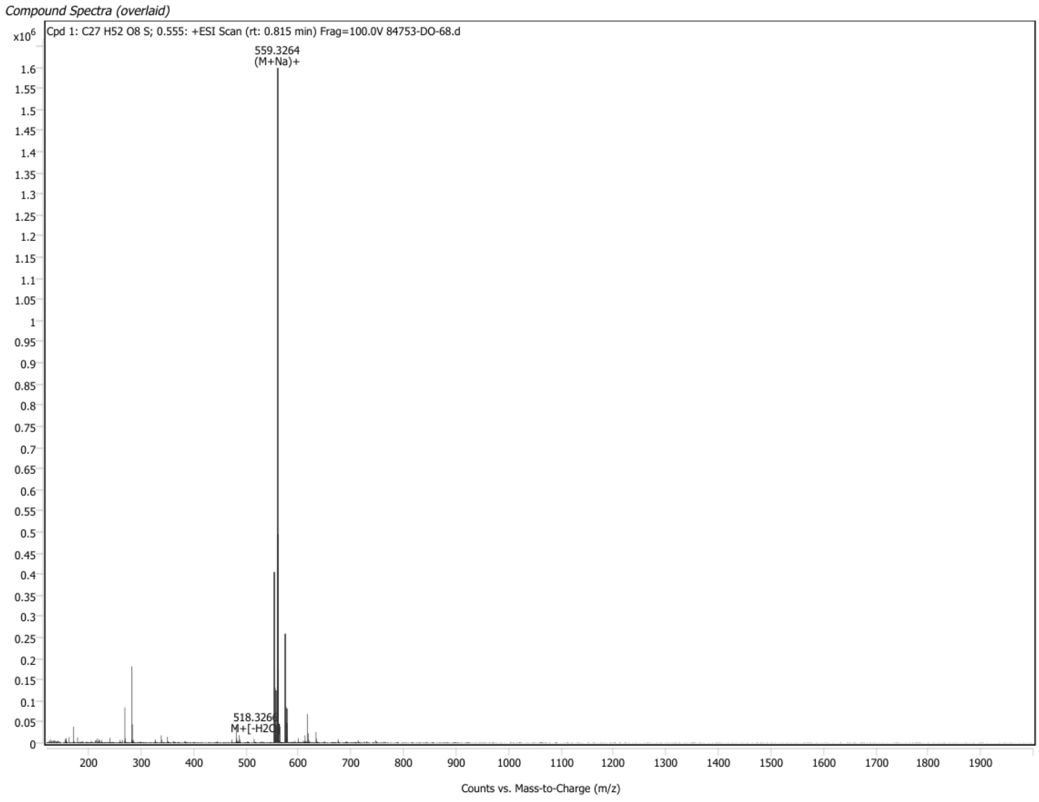
Compound Spectra (overlaid)
Cpd 1: C27 H52 O8 S; 0.555: +ESI Scan (rt: 0.815 min) Frag=100.0V 84753-DO-68.d
559.3264
(M+Na)+
518.326
M+[-H2O
Counts vs. Mass-to-Charge (m/z)

28-Oxo-3,6,9,12,15-pentaoxa-27-thianonacosanoic acid **S17**

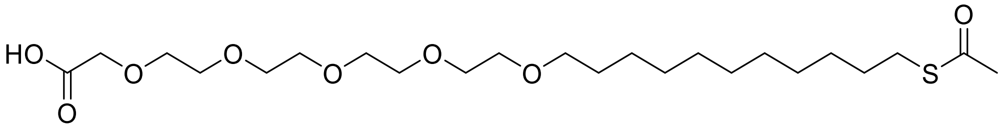


To a dry, sealed vial (purged with $N_2$), containing ester **S16**, was added 1:1 TFA/anhydrous DCM solution (0.2M, 30 eq. of TFA) and left to stir for 4 hrs at RT. The solvent was removed *in vacuo,* yielding **S17** as a yellow, clear oil (quantitative conversion by $^1$H NMR). The compound can be stored as the deprotected carboxylic acid. **$^1$H NMR** (400 MHz, $CDCl_3$) δ 4.15 (s, 2H), 3.78–3.55 (m, 16H), 3.45 (t, *J* = 6.8 Hz, 2H), 2.85 (t, *J* = 7.4 Hz, 2H), 2.32 (s, 3H), 1.61–1.51 (m, 4H), 1.37–1.22 (m, 14H). **ESI-MS** *m/z calcd* for $C_{23}H_{43}O_8S^-$ $[M]^-$ 479.2684 *found* 479.2687.

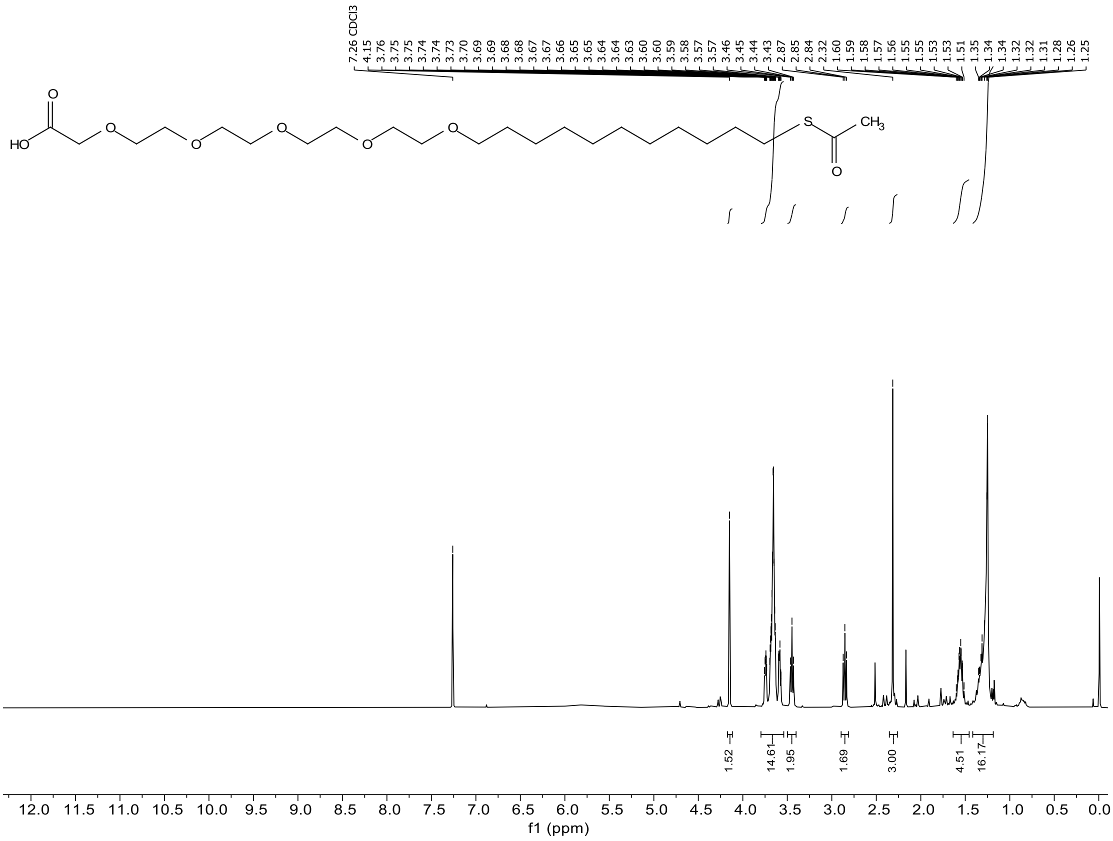

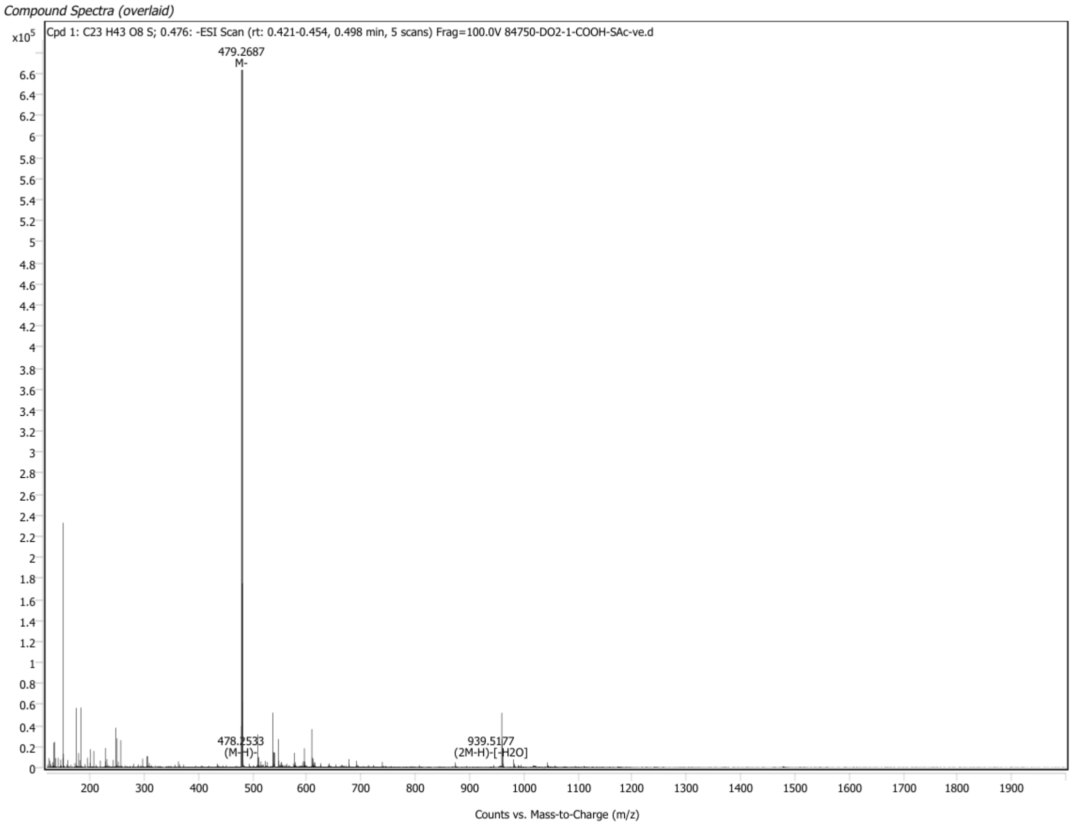


Methyl 26-mercapto-3,6,9,12,15-pentaoxahexacosanoate **16**

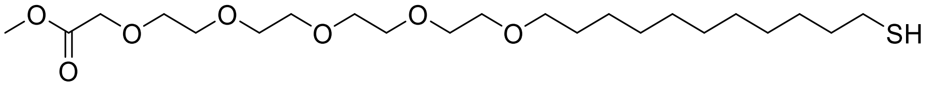


To a vial containing acid **S17** was added 1.25M Methanolic HCl, (1.25M, >10 eq.). The reaction was stirred for 3 hrs at 55 °C to give the product **16**. **$^1$H NMR** (400 MHz, $CDCl_3$) δ 4.17 (s, 2H), 3.75 (s, 3H,), 3.73–3.55 (m, 16H), 3.44 (t, *J* = 6.8 Hz, 2H), 2.52 (q, *J* = 7.5 Hz, 2H), 1.63–1.53 (m, 4H, H10,H18), 1.30–1.23 (m, 14H). **ESI-MS** *m/z calcd* for $C_{22}H_{48}NO_7S$ + $NH_4^+$ [M + $NH_4^+$] 470.3146 *found* 470.3176.

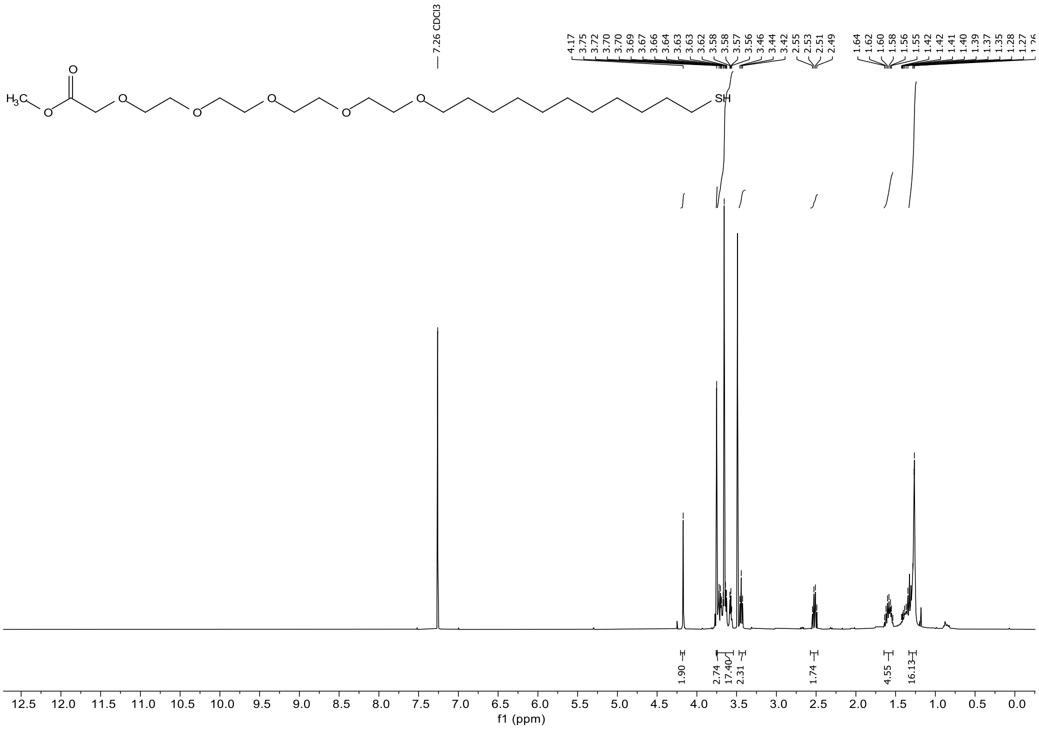
7.26 CDCl3
4.17
3.75
3.72
3.70
3.70
3.69
3.67
3.66
3.64
3.63
3.63
3.62
3.58
3.58
3.57
3.56
3.46
3.44
3.42
2.55
2.53
2.51
2.49
1.64
1.62
1.60
1.58
1.56
1.55
1.42
1.42
1.41
1.40
1.39
1.37
1.35
1.28
1.27
H3C
O
SH
1.90
2.74
17.40
2.31
1.74
4.55
16.13
12.5
12.0
11.5
11.0
10.5
10.0
9.5
9.0
8.5
8.0
7.5
7.0
6.5
6.0
5.5
5.0
4.5
4.0
3.5
3.0
2.5
2.0
1.5
1.0
0.5
0.0
f1 (ppm)

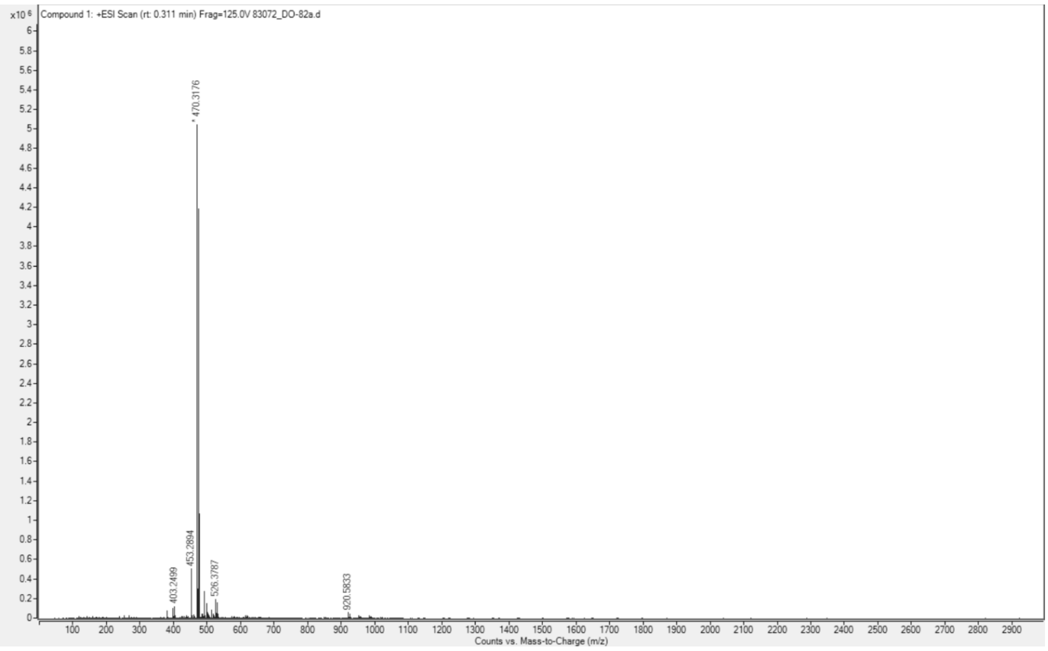
x10 6
Compound 1: +ESI Scan (rt: 0.311 min) Frag=125.0V 83072_DO-82a.d
* 470.3176
453.2894
403.2499
526.3787
920.5833
Counts vs. Mass-to-Charge (m/z)

26-Mercapto-3,6,9,12,15-pentaoxahexacosanoic acid **15**

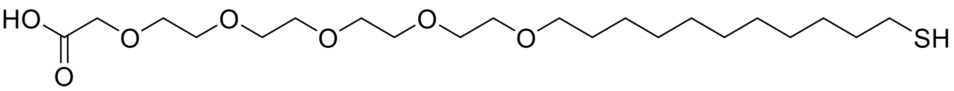


To a vial containing carboxylic acid **S17** was added NaOMe/MeOH (0.1 mL, 4.2M) under inert conditions ($N_2$) for 4 hrs at 55 °C. The reaction was quenched with acetic acid (1.0 eq.) and extracted in $CHCl_3$ and washed with brine. The organic layer is then dried *in vacuo* to give a yellow oil (quantitative) containing product **15**. **$^1$H NMR** (400 MHz, $CDCl_3$) δ 3.92 (s, 2H), 3.76–3.52 (m, 14H), 3.44 (app.dt, *J* = 6.9, 3.4 Hz, 2H), 2.73–2.64 (m, 1H), 2.52 (app.q, *J* = 7.4 Hz, 2H), 1.94 (s, 2H), 1.74–1.49 (m, 4H), 1.42–1.21 (m, 14H). **ESI-MS** *m/z calcd* for $C_{21}H_{42}O_7S$ [M - H]$^-$ 437.2578 *found* submitted 437.2573.

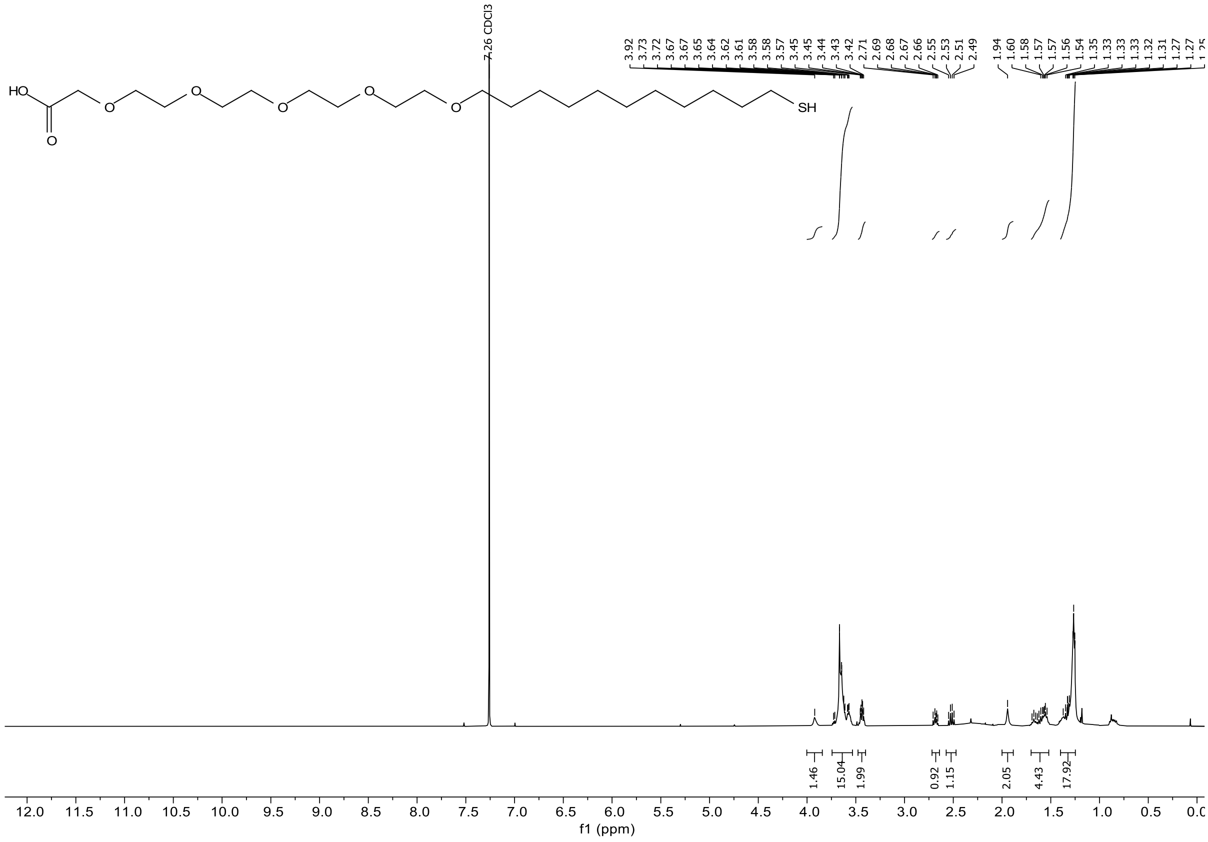

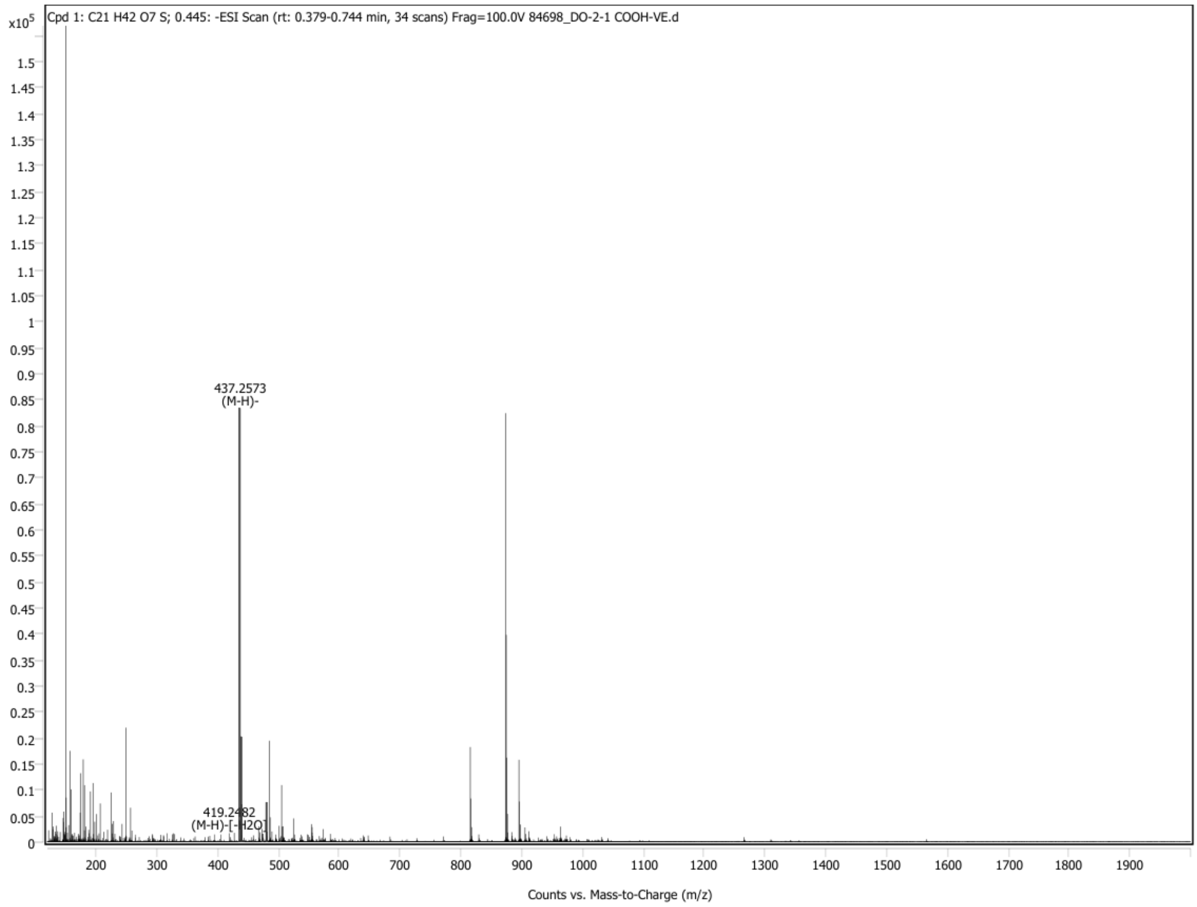


**Synthesis of ‘Positive’ (alk11- and -PEG4-alk11-) thioester and thiol 17 and 18**

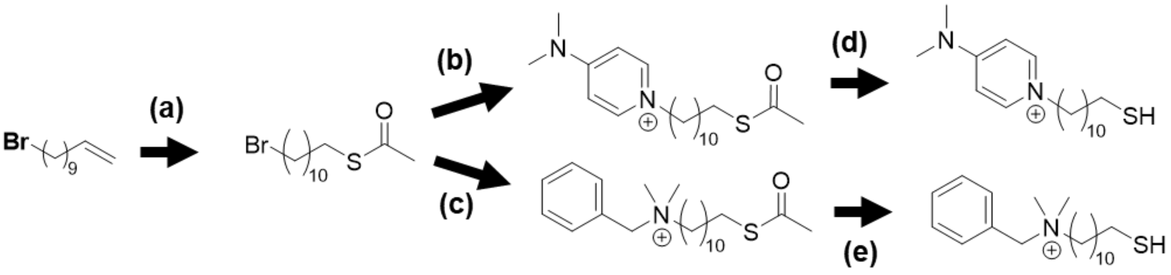


Thioacetic acid, AIBN, anhydrous toluene, $N_2$, 16 hrs, 75 °C, 81% (b) DMAP, anhydrous MeCN, 16 hrs, 75 °C, 77 % (c) *N,N*-dimethyl-benzylamine, EtOH, 16 hrs, 80 °C, 81% (d) & (e) HCl/MeOH (1.25 M, > 10 eq.), 3h, 55 °C, quant.

S-(11-Bromoundecyl) ethanethioate **S18**

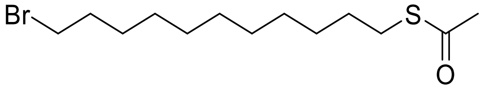

11-Bromo-undec-1-ene (2.33 g, 10.0 mmol) was dissolved in anhydrous toluene (35 mL) and sparged with $N_2$ for 10 mins. AIBN (0.32 g, 1.95 mmol) was added, followed by addition of thioacetic acid (2.98 g, 39.19 mmol). The mixture was heated with stirring to 75 °C, under $N_2$ for 16 hrs. After cooling to ambient temperature, the solvent was removed *in vacuo* and the residue redissolved in DCM (50 mL). The organic layer was washed with sat. aq. $NaHCO_3$ (3 × 30 mL), $H_2O$ (30 mL), and sat. aq. brine (30 mL). The organic layer was then dried ($MgSO_4$), filtered through cotton wool and dried *in vacuo*. The compound was purified by silica column chromatography (5% pet. ether/DCM) yielding **S18** as a clear yellow oil (2.497 g, 8.07 mmol,

81%). Observed analytical data matched that of the original work.[10] $^{1}$**H NMR** (400 MHz, $CDCl_3$) δ 3.40 (s, 2H), 2.86 (t, *J* = 7.4 Hz, 2H), 2.32 (s, 3H), 1.91–1.81 (m, 2H), 1.60–1.50 (m, 2H), 1.46–1.24 (m, 14H).$^{13}$**C NMR** (101 MHz, $CDCl_3$) δ 195.5, 33.7, 32.6, 30.4, 29.3, 29.2, 29.2, 29.2, 28.9, 28.9, 28.6, 28.5, 28.0.

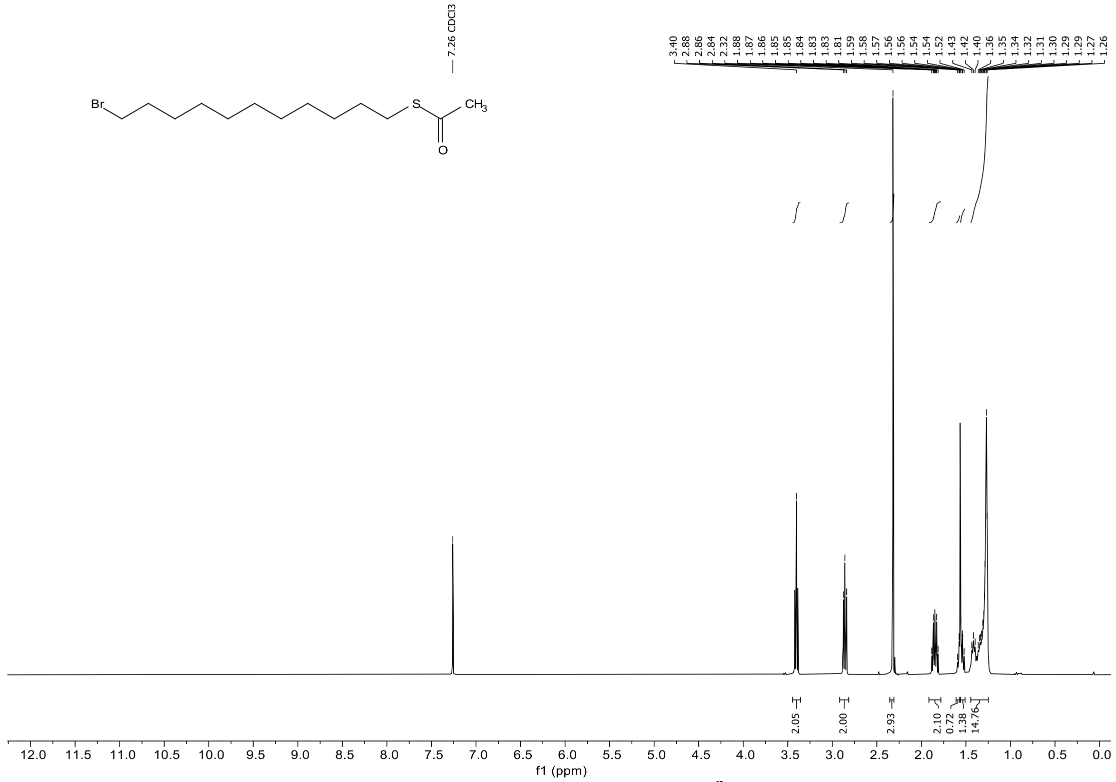


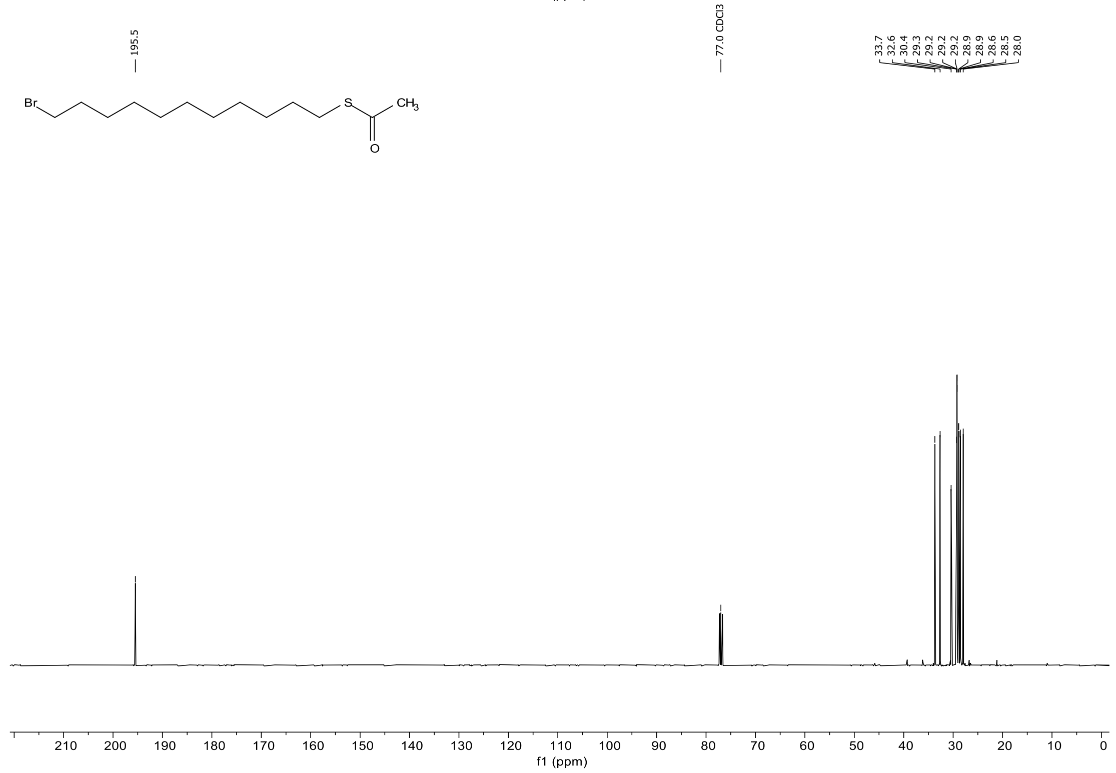

1-(11-(Acetylthio)undecyl)-4-(dimethylamino)pyridin-1-ium bromide **S19**

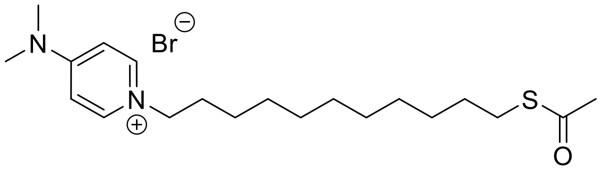


Bromide **S18** (0.10 g, 0.325 mmol) and 4-Dimethylaminopyridine (0.034 g, 0.325 mmol), were dissolved in anhydrous MeCN (1 mL). The reaction was heated to 75 °C and left to stir for 16 hrs. The mixture was concentrated *in vacuo*, the crude product was triturated with cold hexanes:ether (1:1) and further dried *in vacuo*. The product **S19** was obtained without further purification as a brown solid. (0.084 g, 0.195 mmol, 59 %). **$^1$H NMR** (400 MHz, $CDCl_3$) δ 8.45 (d, *J* = 7.8 Hz, 2H), 7.02 (d, *J* = 7.8 Hz, 2H), 4.34 (t, *J* = 7.4 Hz, 2H), 3.27 (s, 6H), 2.85 (t, *J* = 7.4 Hz, 2H), 2.32 (s, 3H), 1.60–1.49 (m, 2H), 1.40–1.18 (m, 14H). **ESI-MS** *m/z calcd* for $C_{20}H_{35}N_2OS$ $[M]^+$ 351.2465 *found* 351.2518.

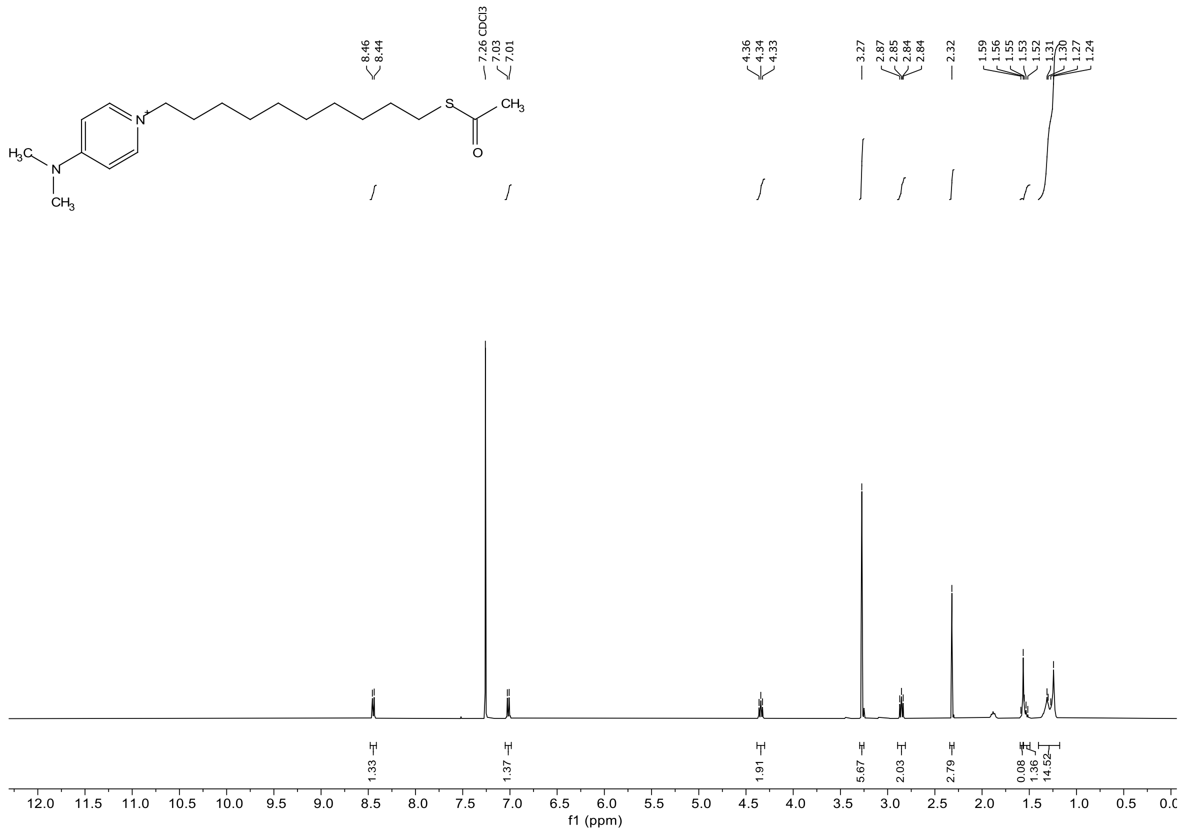

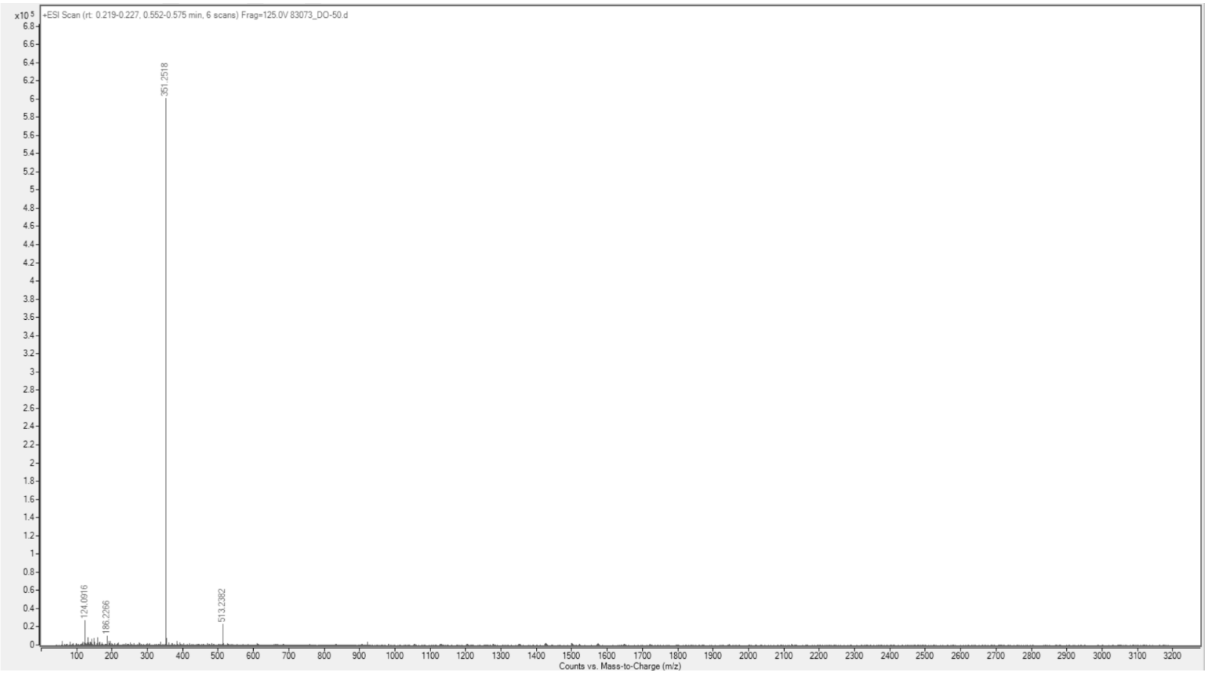


4-(Dimethylamino)-1-(11-mercaptoundecyl)pyridin-1-ium chloride **17**

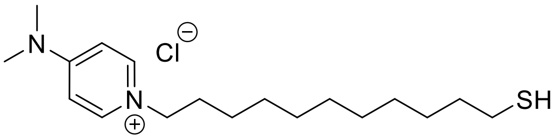

Thioester **S19** was deprotected using methanolic HCl (1.25M, >10 eq.), for 3 hrs at 55 °C. The solvent was evaporated, obtaining the thiolated compound. **$^1$H NMR** (400 MHz, $CDCl_3$) 8.46 (d, *J* = 7.8 Hz, 2H), 7.01 (d, *J* = 7.8 Hz, 2H), 4.35 (t, *J* = 7.4 Hz, 2H), 3.27 (s, 6H), 2.52 (q, *J* = 7.4 Hz, 2H), 1.89 (t, *J* = 7.4 Hz, 2H), 1.60 (m, 2H), 1.43–1.20 (m, 16H). **ESI-MS** *m/z calcd* for $C_{18}H_{33}N_2S+$ $[M]^+$ 309.2359 *found* 309.2360.

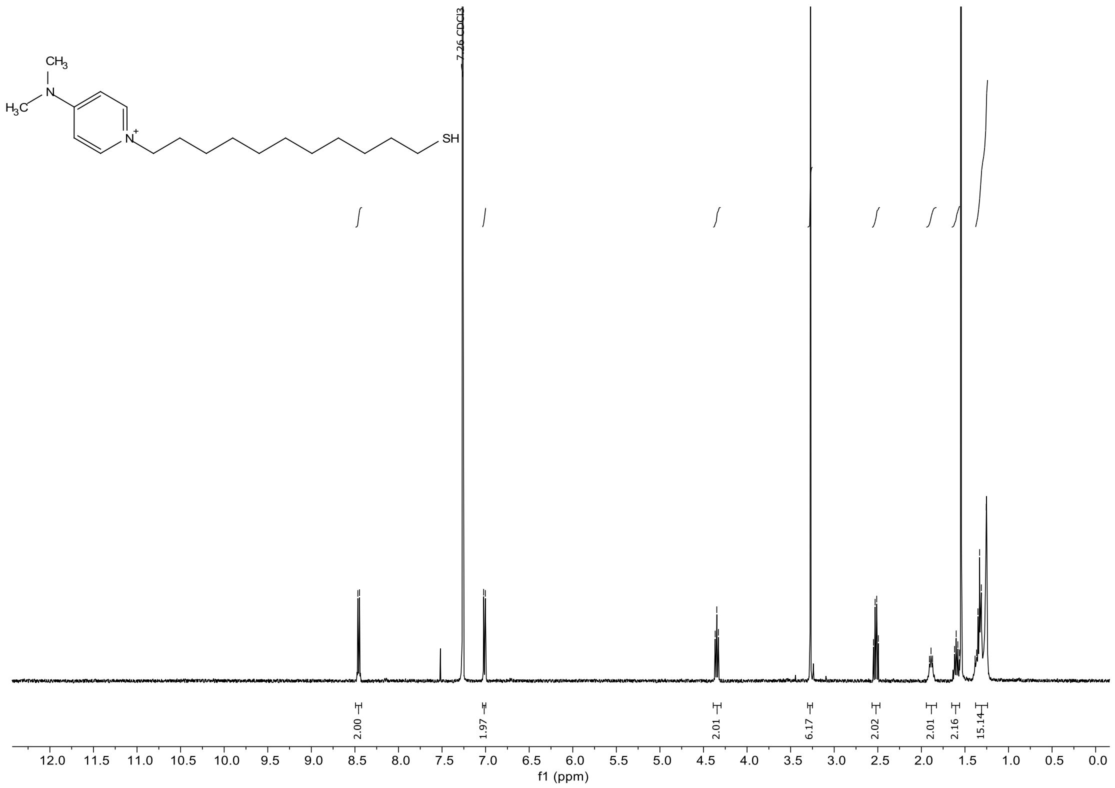

7.26 CDCl3
SH
2.00
1.97
2.01
6.17
2.02
2.01
2.16
15.14
12.0 11.5 11.0 10.5 10.0 9.5 9.0 8.5 8.0 7.5 7.0 6.5 6.0 5.5 5.0 4.5 4.0 3.5 3.0 2.5 2.0 1.5 1.0 0.5 0.0
f1 (ppm)


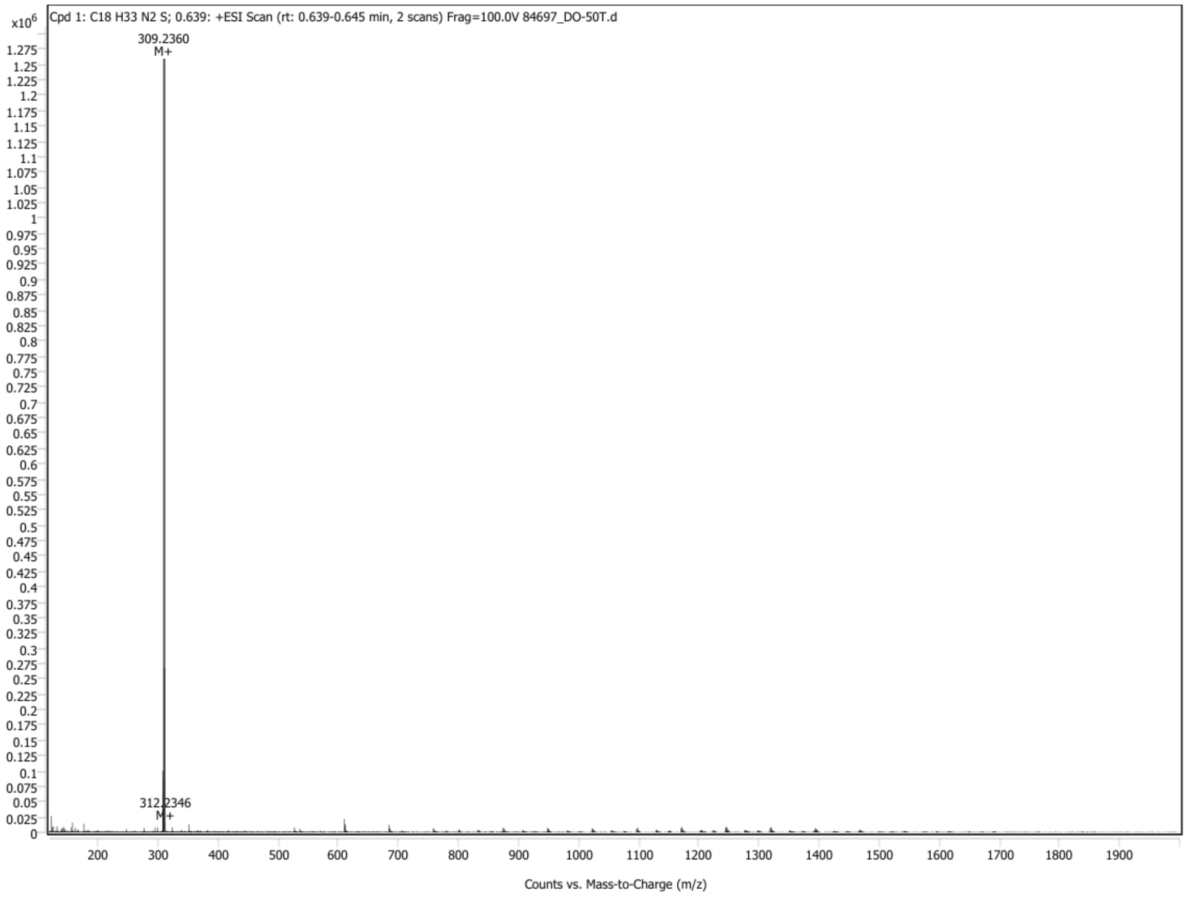

Cpd 1: C18 H33 N2 S; 0.639: +ESI Scan (rt: 0.639-0.645 min, 2 scans) Frag=100.0V 84697_DO-50T.d
x10^6
309.2360
M+
312.2346
M+
Counts vs. Mass-to-Charge (m/z)

11-(Acetylthio)-N-benzyl-N,N-dimethylundecan-1-aminium bromide **S20**

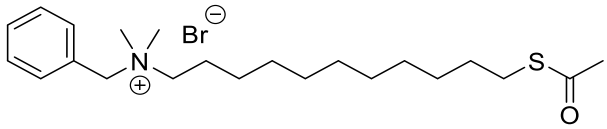

An oven dried vial was charged with bromide **S18** (0.120 g, 0.39 mmol) and EtOH (3.9 mL) and subsequently stirred and sparged with $N_2$ for 5 mins. N,N-dimethyl-benzylamine (0.157 g, 1.17 mmol) was added to the solution. The reaction was heated to 80 °C and left to stir for 16 hrs. The solvent was removed in *vacuo*, and the residue triturated with $Et_2O$ (5 mL) and hexanes (5 mL) and further dried in vacuo. The residue was dissolved in DCM (50 mL), was washed with $NaHCO_3$ (15 mL), $H_2O$ (15 mL), brine (15 mL). The organic phase was dried ($MgSO_4$), and the solvent removed *in vacuo*. The resulting product was triturated with $Et_2O$ (5 mL) to remove any starting material and further dried *in vacuo*, leaving product **S20** as a light brown solid (0.144 g, 0.31 mmol, 81%). **$^1$H NMR** (400 MHz, $CDCl_3$) 7.67–7.39 (m, 5H), 5.03 (s, 2H), 3.54–3.42 (m, 2H), 3.29 (s, 6H), 2.84 (t, $J$ = 7.4 Hz, 2H), 2.31 (s, 3H), 1.95 (br, 2H), 1.54 (app.p, $J$ = 7.1 Hz, 2H), 1.37–1.14 (m, 14H). **$^{13}$C NMR** (101 MHz, $CDCl_3$) δ 196.1, 133.2, 130.7, 129.2, 127.3, 67.5, 63.7, 49.7, 30.6, 29.4, 29.3, 29.2, 29.2, 29.2, 29.1, 29.0, 28.7, 26.2, 22.9. **ESI-MS** *m/z calcd* for $C_{22}H_{38}NOS$ $[M]^+$ 364.2669 *found* 364.2666.

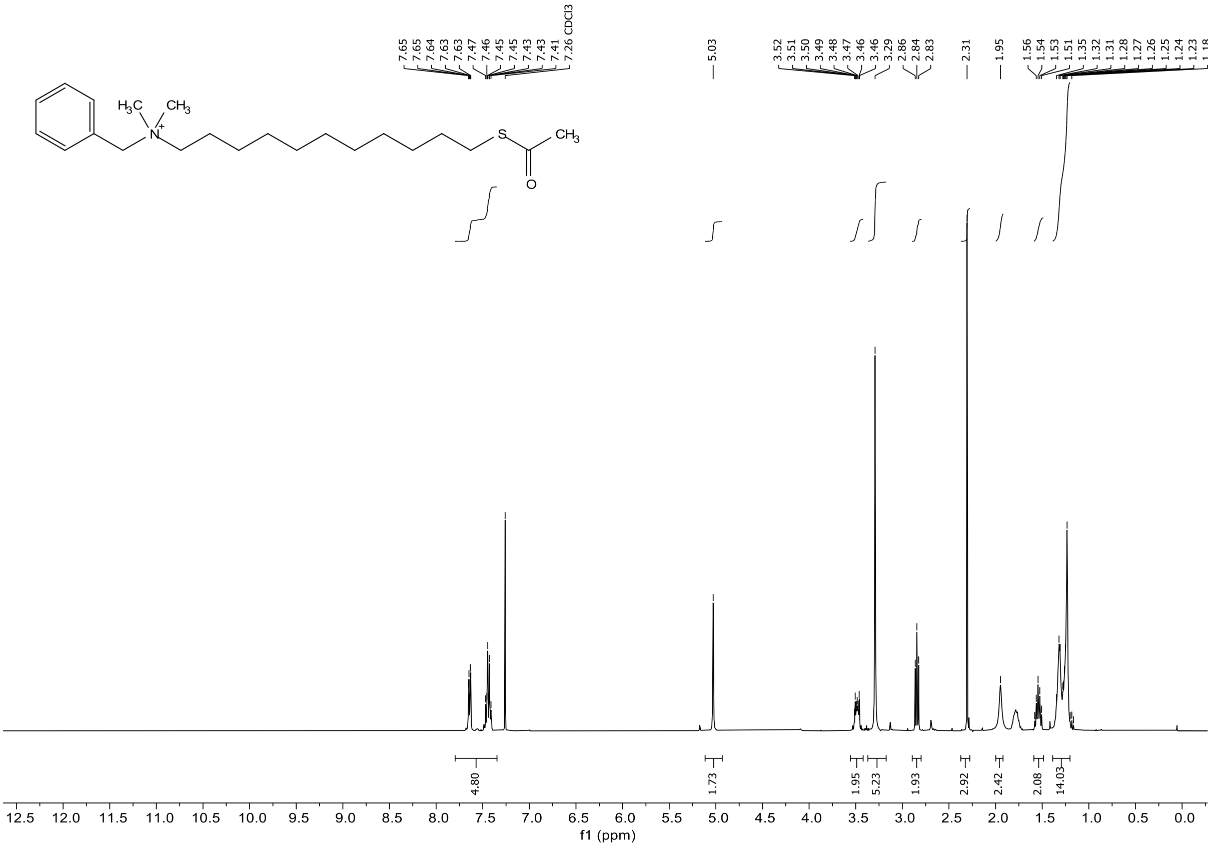

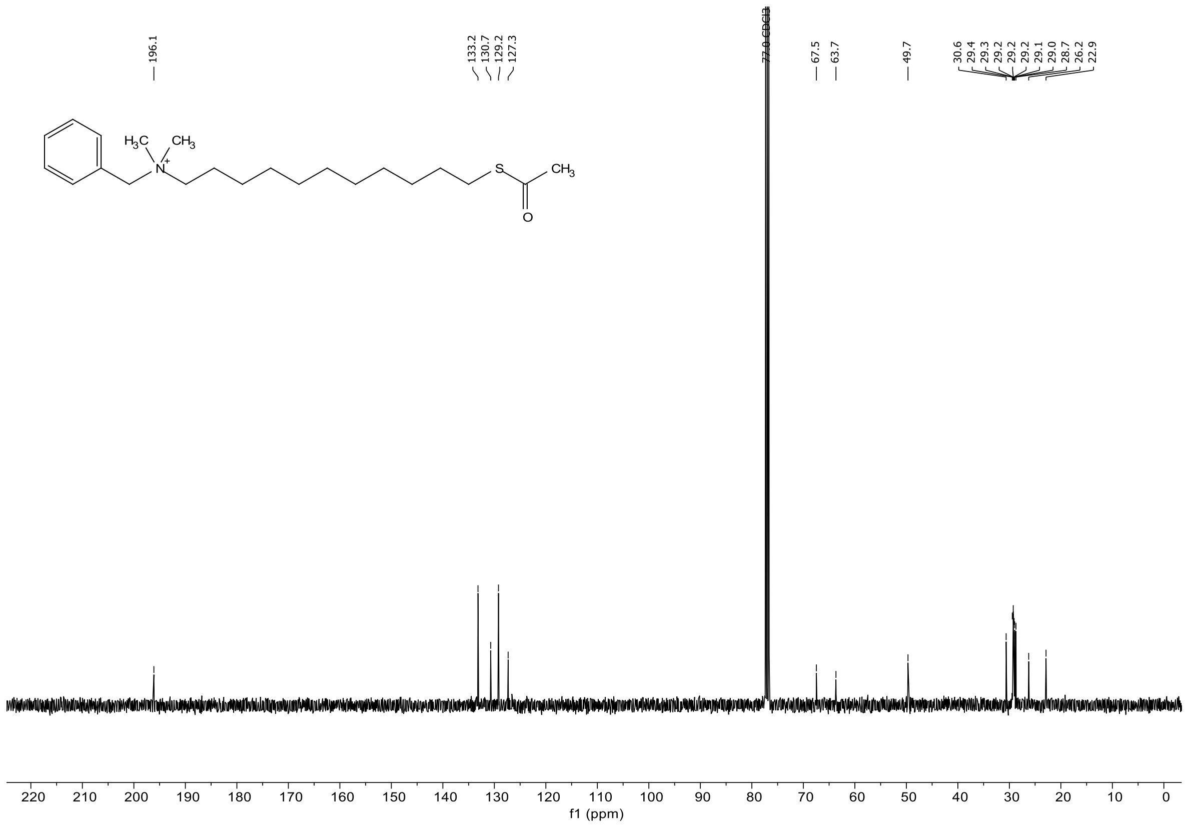


*Compound Spectra (overlaid)*

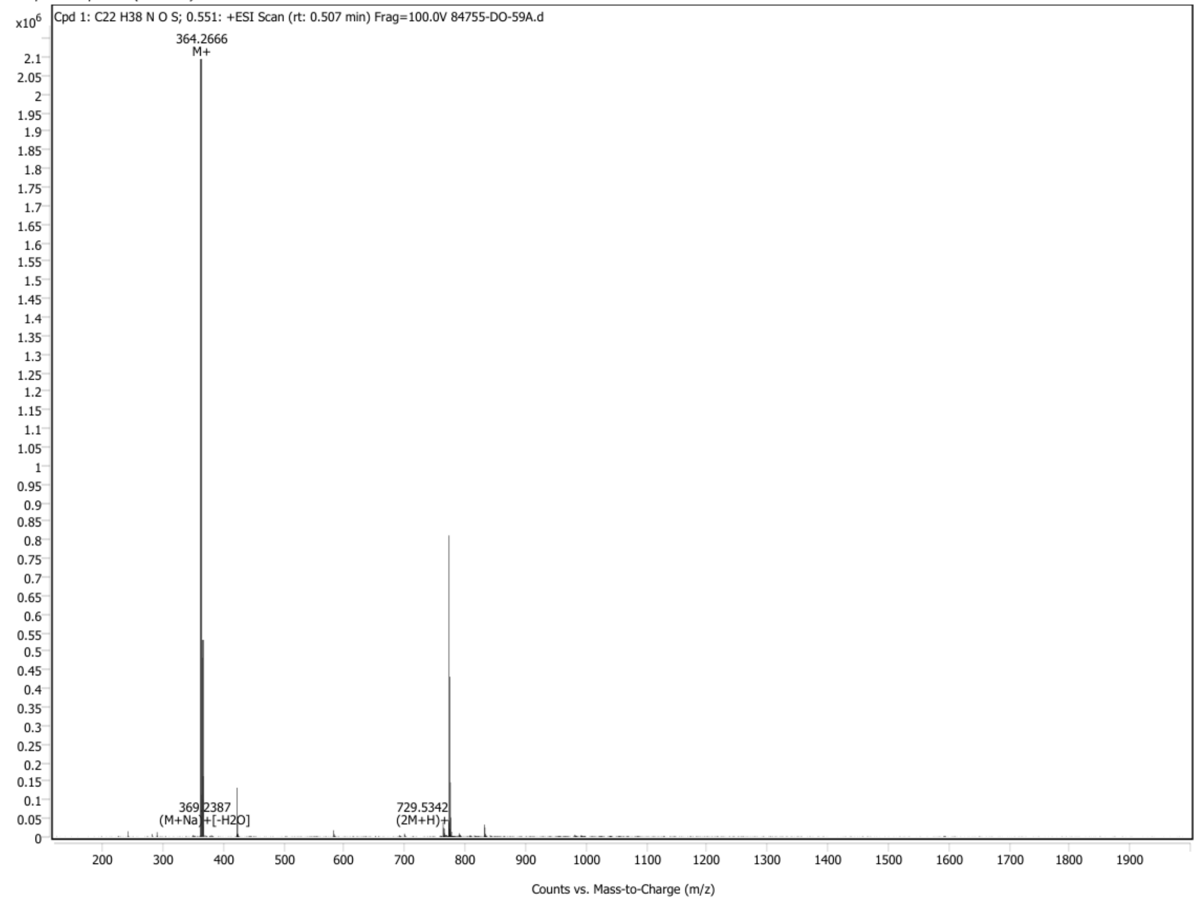

N-benzyl-11-mercapto-N,N-dimethylundecan-1-aminium chloride **18**

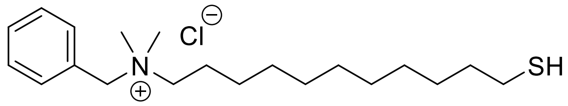


Thioester **S20** was deprotected using methanolic HCl (1.25M, >10 eq.), 3 hrs at 55 °C to yield the corresponding thiol **18**.[1]**H NMR** (400 MHz, $CDCl_3$) δ 7.70–7.41 (m, 5H), 5.04 (s, 2H), 3.56–3.45 (m, 2H), 3.31 (s, 6H), 2.52 (q, *J* = 6.9 Hz, 2H), 1.79 (br, *J* = 10.5 Hz, 2H), 1.67–1.51 (m, 2H), 1.44–1.21 (m, 14H).m **ESI-MS** *m/z calcd* for $C_{20}H_{36}NS^{+}$ $[M]^{+}$ 322.2563 *found* 322.2566

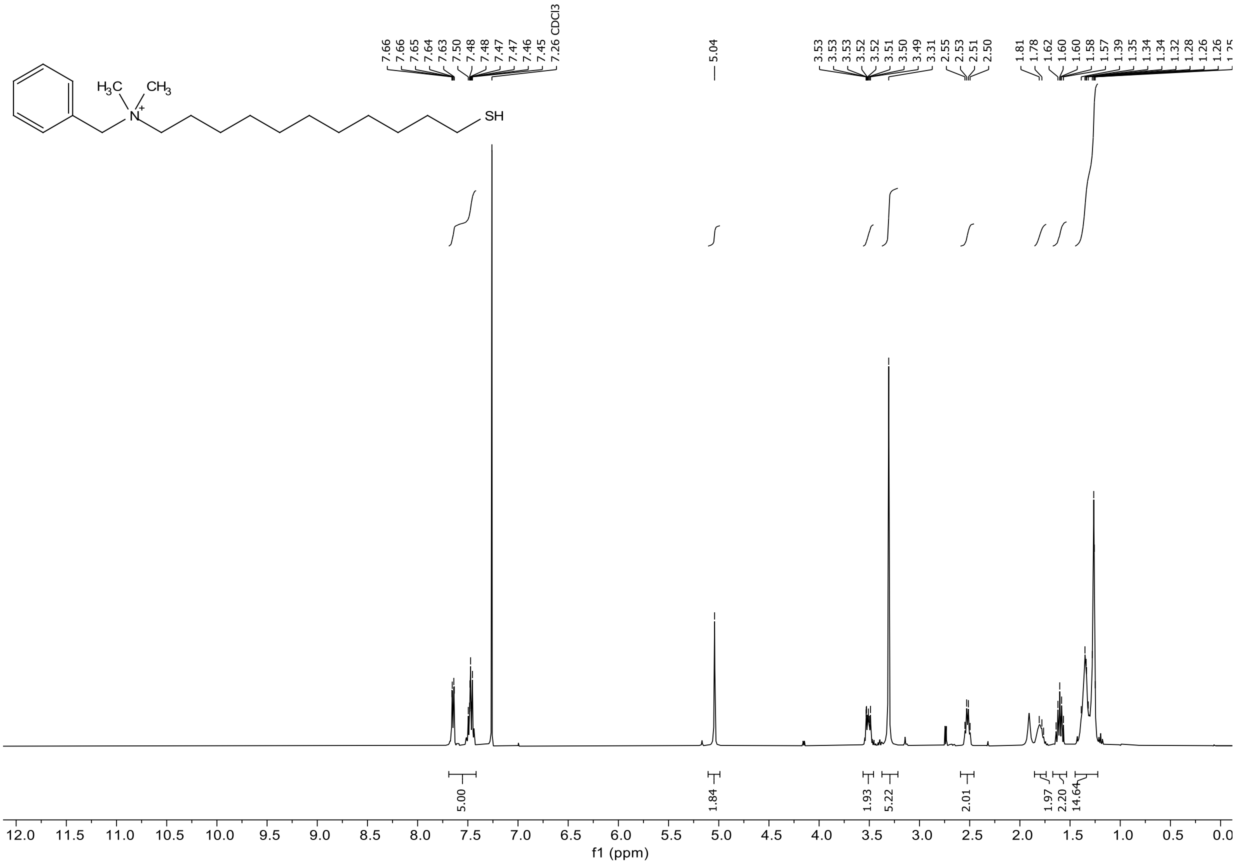

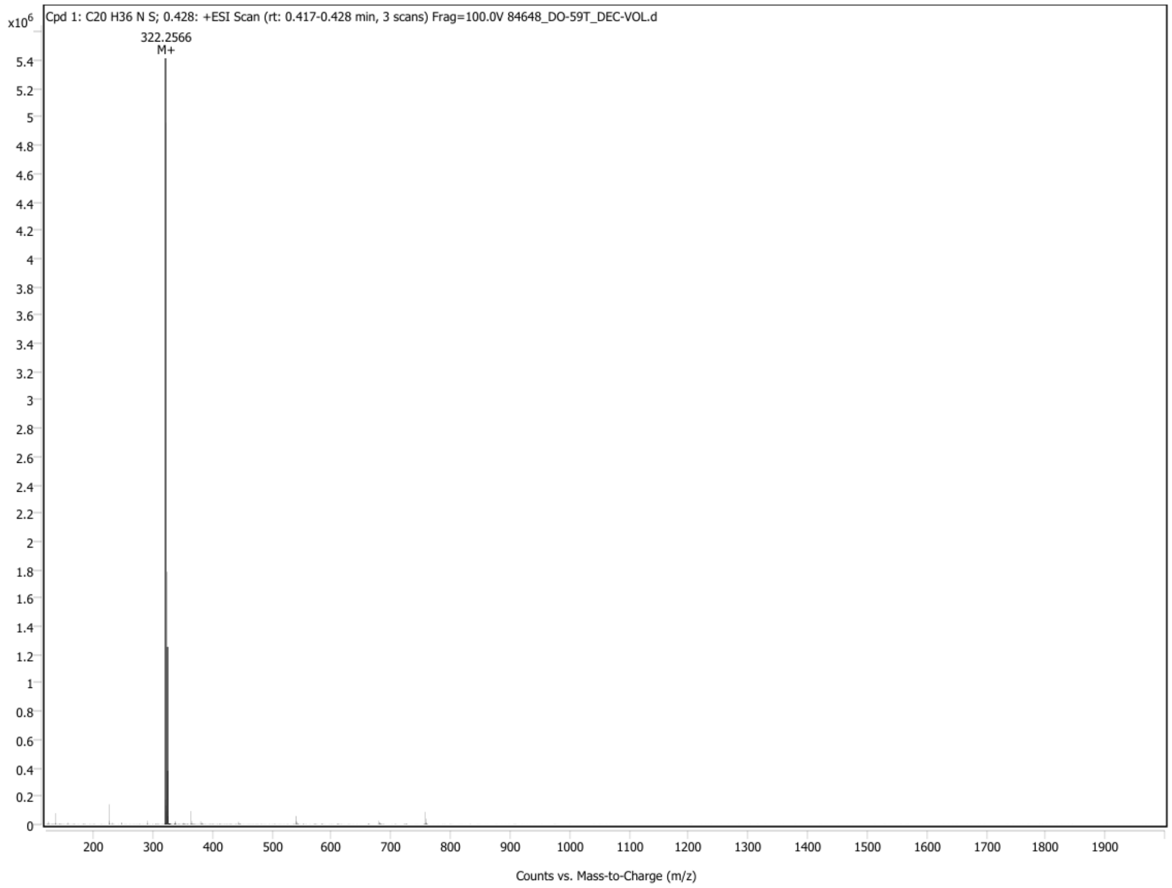


**Synthesis of 'Positive' Amino-(-PEG4-alk11-) Ligands 19–21**

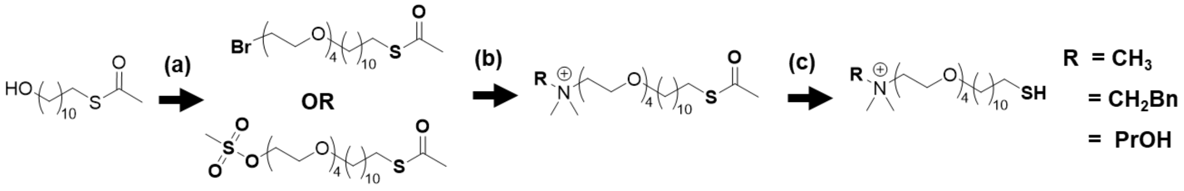


(a1) MsCl, $NEt_3$, anhydrous DCM, $N_2$, 16 hrs, RT, quant. (a2) $CBr_4$,$PPh_3$, anhydrous DCM, $N_2$,16 hrs, RT, 85% (b) react with tertiary amine (c) HCl /MeOH (1.25 M, > 10 eq.), 3h, 55 °C, quant.

S-(1-Bromo-3,6,9,12-tetraoxatricosan-23-yl) ethanethioate **S21**

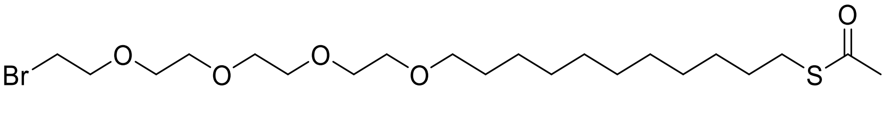

Adapted from P. Dutta *et al.,*[11] alcohol **S14** (0.49 g, 1.15 mmol), was dissolved in anhydrous DCM (4.3 mL) and sparged with $N_2$ for 15 mins. Carbon tetrabromide was added portionwise (0.46 g, 1.36 mmol) under stirring. The mixture was cooled to 0 °C and triphenyl phosphine ($PPh_3$, 0.44 g, 1.67 mmol), dissolved in anhydrous DCM (1 mL), was added dropwise. The reaction was allowed to warm to RT and left to stir for 16 hrs. The solvent was removed *in vacuo*, leaving a clear orange oil. The mixture was extracted with petroleum ether (40–60) (3 × 15 mL), leaving behind triphenyl phosphine oxide. The combined organic layers were concentrated *in vacuo.* Purification by column chromatography (30% EtOAc/petroleum ether 40–60) obtained the product **S21** as a yellow oil (0.565 g, 1.17 mmol , 75%). The analytical data was closely analogous to that of the original work (n.b. original work utilised a PEG3 linker, this work uses a PEG4 linker).[11] **$^1$H NMR** (400 MHz, $CDCl_3$) δ 3.81 (t, $J$ = 6.3, 2H), 3.72–3.58 (m, 12H), 3.51–3.41 (m, 4H), 2.86 (t, $J$ = 7.4 Hz, 2H), 2.32 (s, 3H), 1.56 (m, 4H), 1.26 (m, 14H). **$^{13}$C NMR** (101 MHz, $CDCl_3$) δ 196.0, 71.5, 71.2, 70.7, 70.6, 70.6, 70.6, 70.5,

70.0, 30.6, 30.3, 29.6, 29.5, 29.5, 29.5, 29.4, 29.4, 29.1, 29.1, 28.8, 26.1. **ESI-MS** *m/z calcd* for $C_{21}H_{41}BrO_5S$ [M + $NH_4$]$^+$ 504.2176 *found* 504.2177.

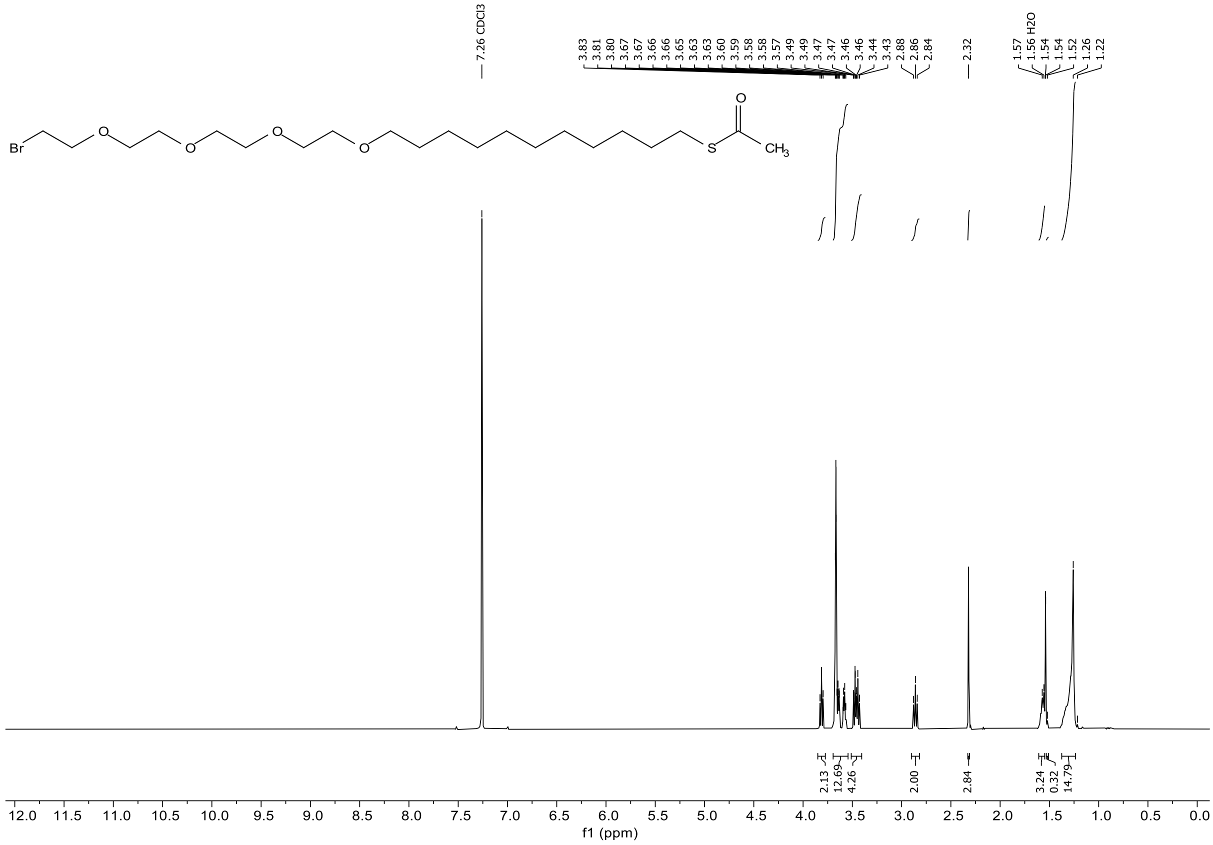


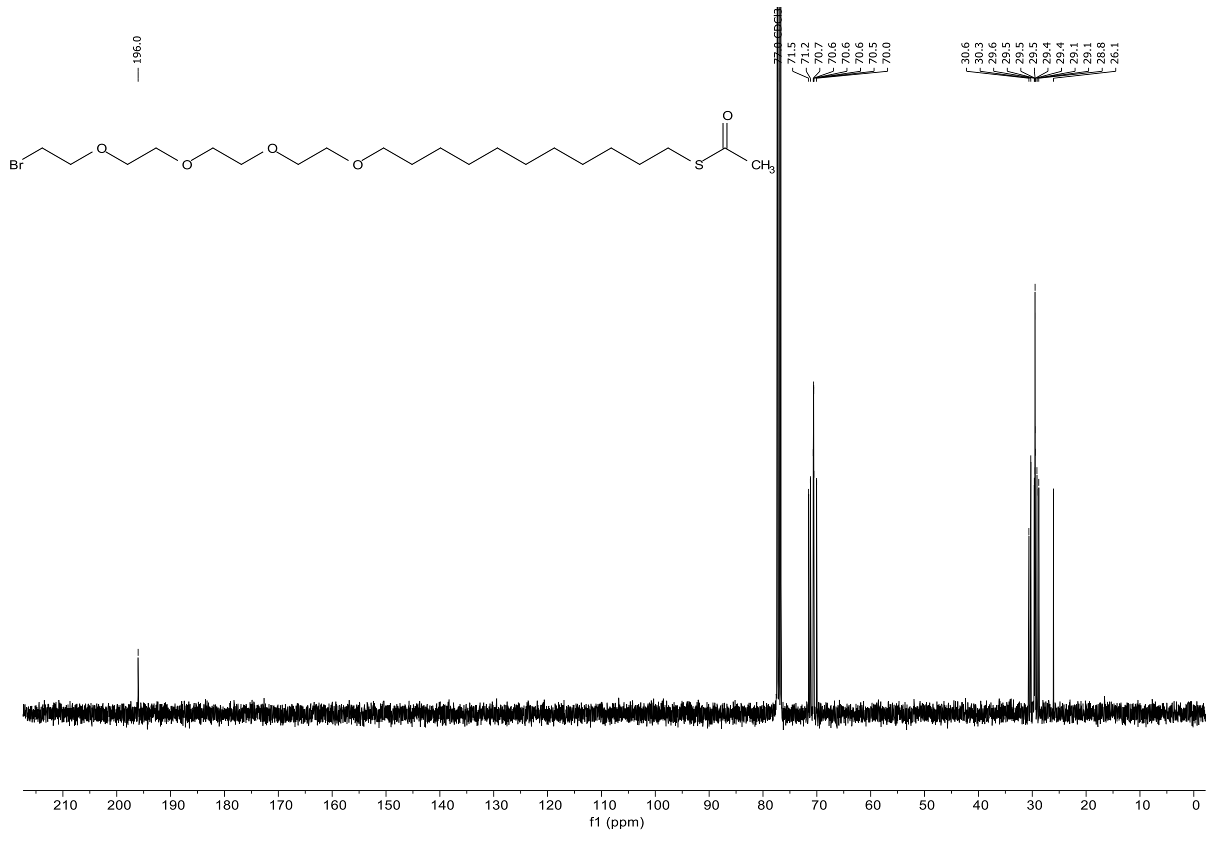

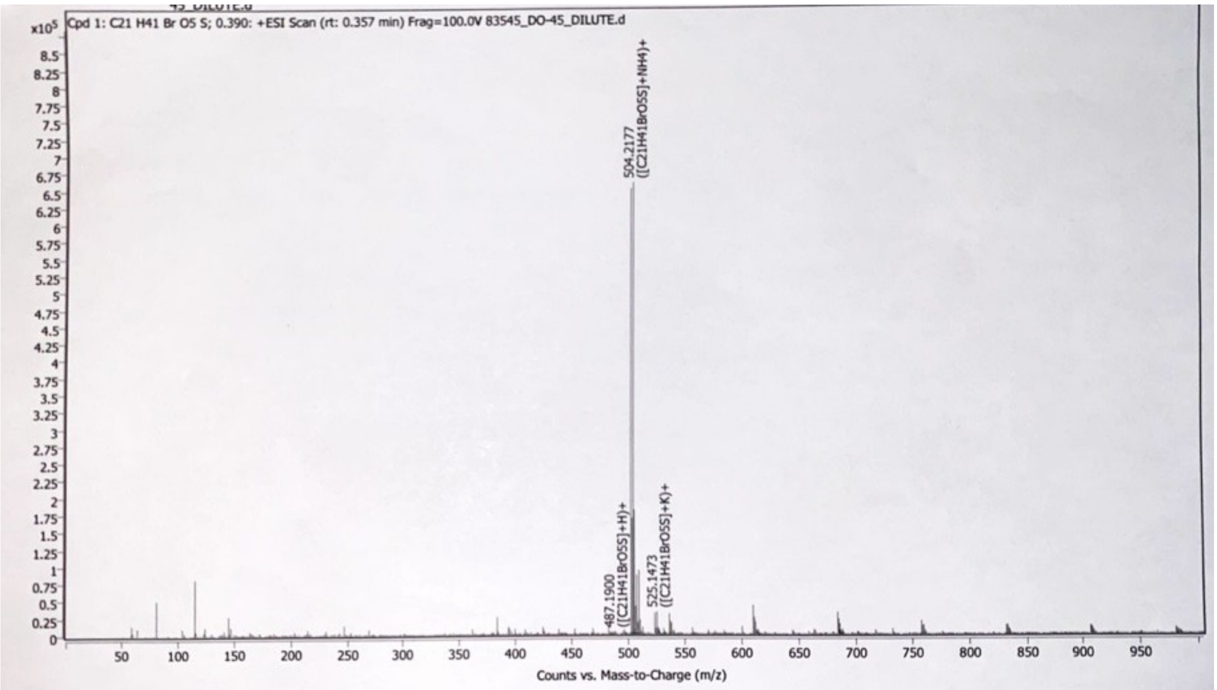

45_DILUTE.d
x10^5
Cpd 1: C21 H41 Br O5 S; 0.390: +ESI Scan (rt: 0.357 min) Frag=100.0V 83545_DO-45_DILUTE.d
504.2177
([C21H41BrO5S]+NH4)+
487.1900
([C21H41BrO5S]+H)+
525.1473
([C21H41BrO5S]+K)+
Counts vs. Mass-to-Charge (m/z)

N,N,N-trimethyl-25-oxo-3,6,9,12-tetraoxa-24-thiahexacosan-1-aminium bromide **S22**

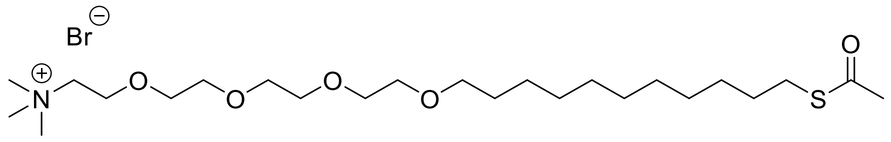


The molecule was synthesised according to literature procedure.[6] Bromide **S21** (0.073 g, 0.15 mmol) was dissolved in EtOH (1.5 mL), and trimethylamine (4.2 M in EtOH, 0.02 mL, 0.30 mmol) was added. The reaction mixture was stirred under $N_2$ at RT for 48 h. The solvent was then removed *in vacuo* and the residue was triturated with cold hexanes:ether (1:1). The residue was further dried to obtain **S22** as a cloudy white oil (0.064 g, 0.14 mmol, 91%). Observed analytical data matched that of the original work.[12]

**$^1$H NMR** (400 MHz, $CDCl_3$) δ 4.05 (d, *J* = 6.7 Hz, 4H), 3.73–3.54 (m, 12H), 3.49 (s, 9H), 3.43 (t, *J* = 6.9 Hz, 2H), 2.88 (t, *J* = 7.4 Hz, 2H), 2.35 (s, 3H), 1.64–1.50 (m, 4H), 1.28 (m, 14H).

**ESI-MS** *m/z calcd* for $C_{24}H_{50}NO_5S^+$ $[M]^+$ 464.3404 *found* 464.3413.

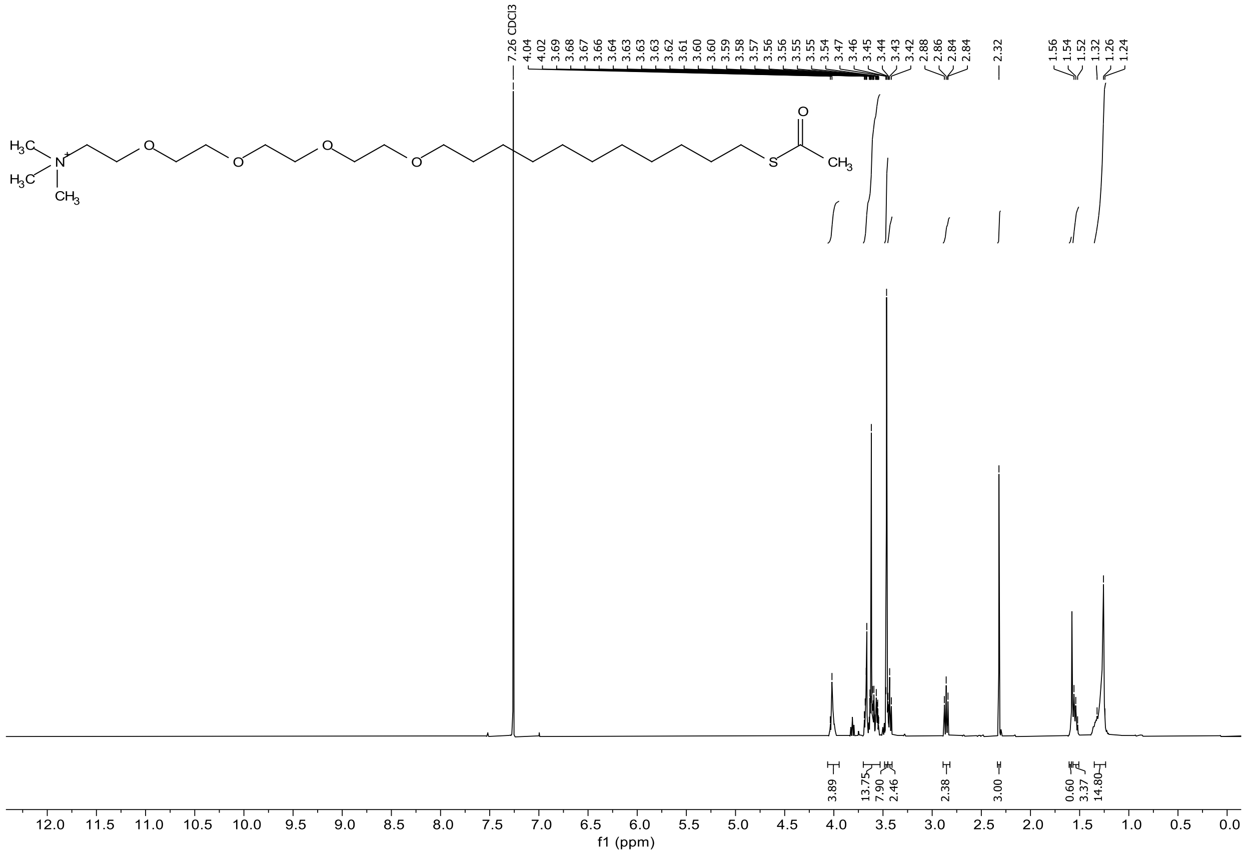

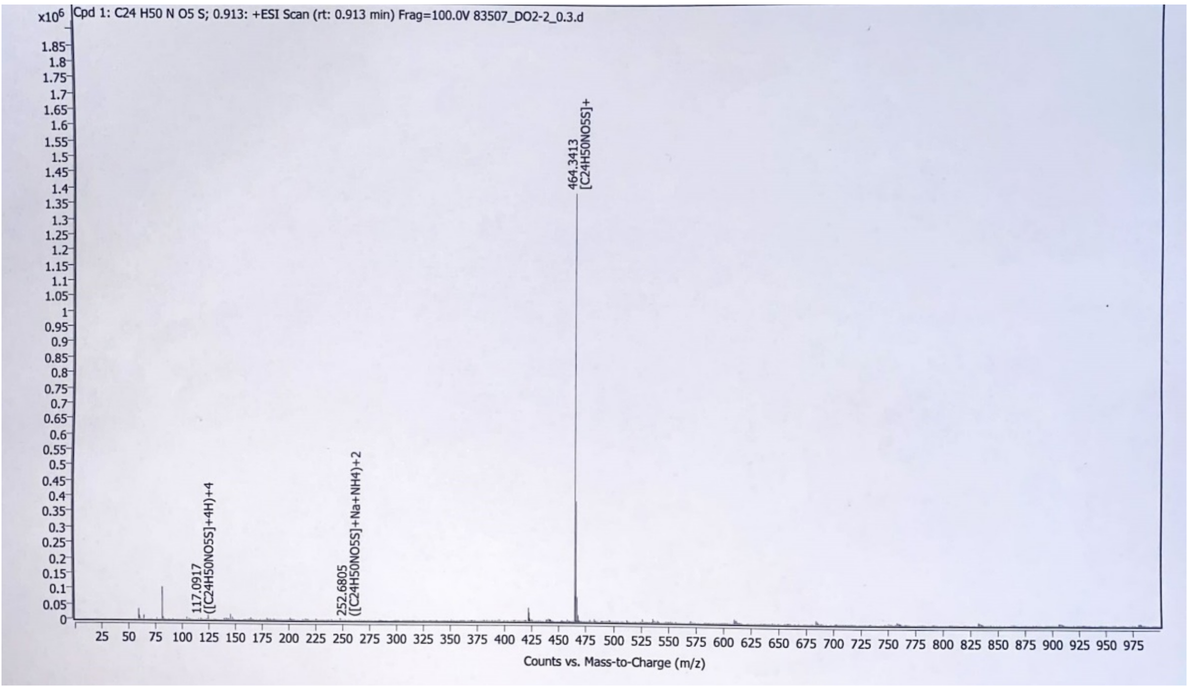


23-Mercapto-N,N,N-trimethyl-3,6,9,12-tetraoxatricosan-1-aminium chloride **19**

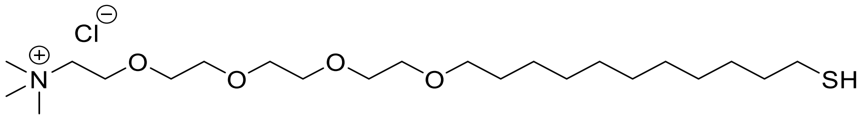


Thioester **S22** was deprotected using methanolic HCl (1.25M, >10 eq.), 3 hrs at 55 °C to give the corresponding thiol **19** (quant. by NMR). **$^1$H NMR** (400 MHz, $CDCl_3$) δ 4.01 (s, 4H), 3.70–3.54 (m, 12H), 3.47 (s, 9H), 3.43 (t, *J* = 6.9 Hz, 2H), 2.52 (q, *J* = 7.5 Hz, 2H), 1.61–1.52 (m, 2H), 1.40–1.21 (m, 14H). **ESI-MS** *m/z calcd* for $C_{22}H_{48}NO_4S^+$ [M]$^+$ 422.3299 *found* 422.3306.

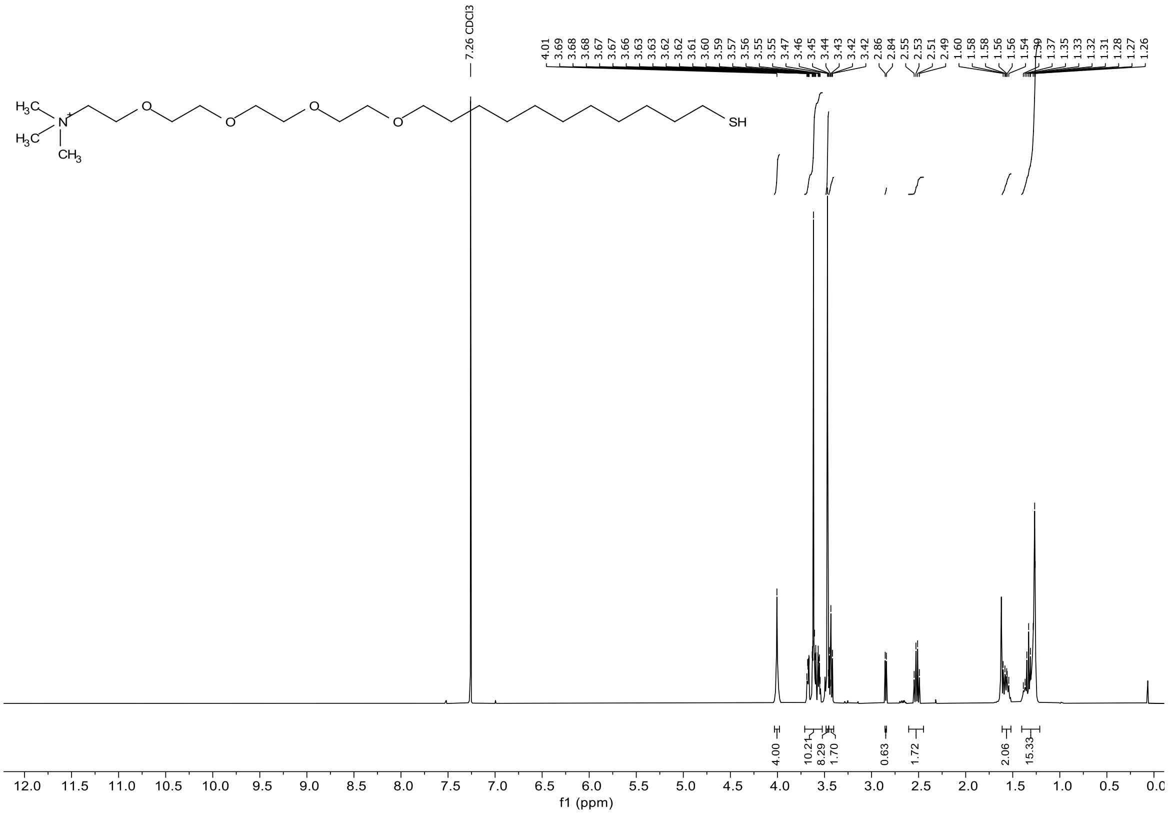

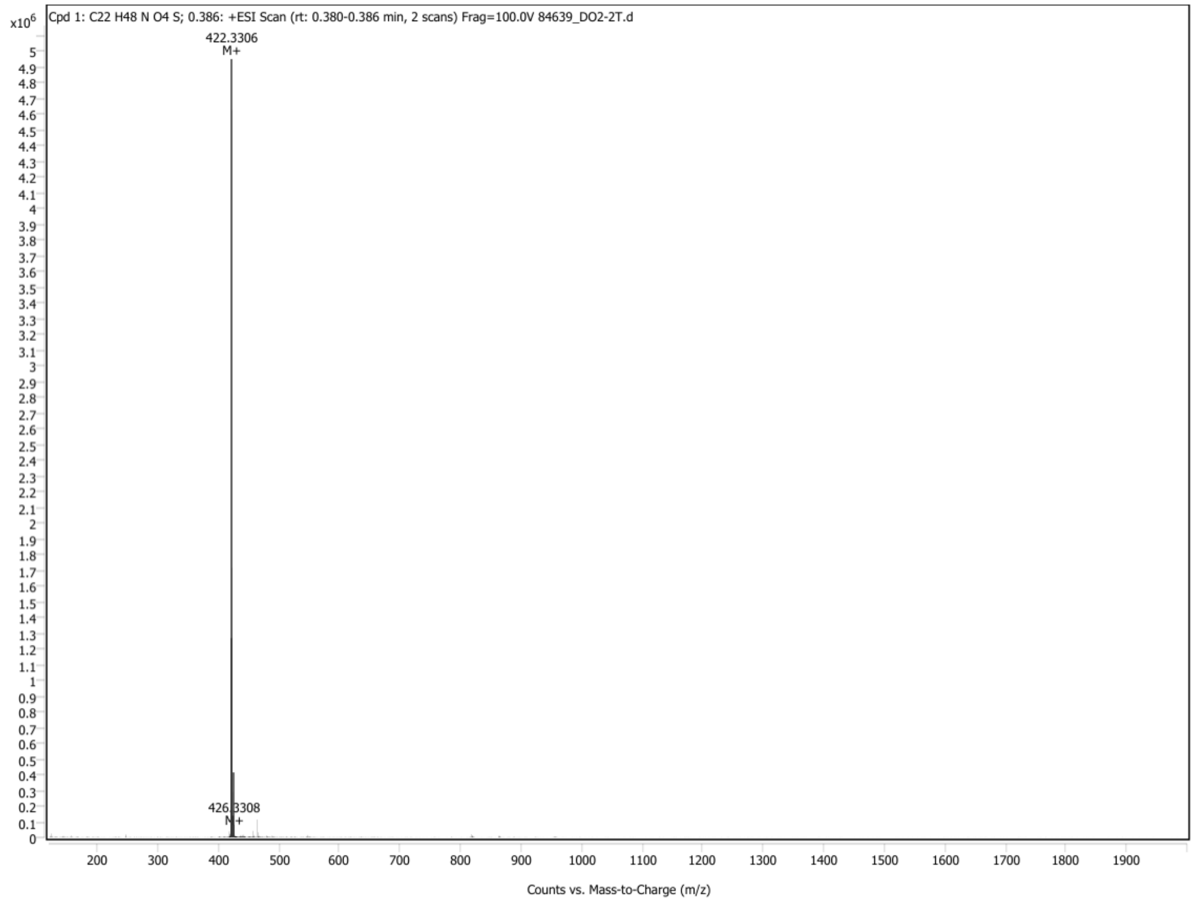


N-benzyl-N,N-dimethyl-25-oxo-3,6,9,12-tetraoxa-24-thiahexacosan-1-aminium methanesulfonate **S23**

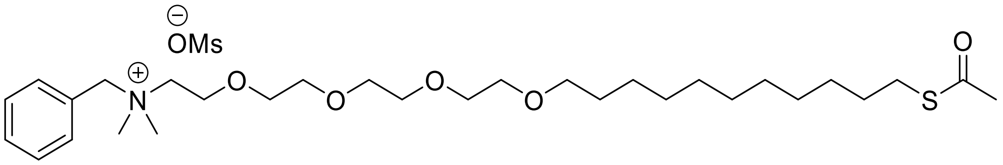


Alcohol **S14** (0.200 g, 0.47 mmol) was dissolved in anhydrous DCM (1.5 mL) under $N_2$. To the mixture, NEt3 (0.1 mL, 0.71 mmol) was added at 0 °C and the reaction was stirred for 15 mins. Methane sulfonyl chloride (0.04 mL, 0.52 mmol) was added dropwise to the flask. The reaction mixture was stirred at room temperature for 16 hrs. The solvent was removed *in vacuo* and the crude mixture re-dissolved in EtOAc. The organic layer was washed with dilute HCl (0.1 M, 2 × 10 mL) and sat. aq. $NH_4Cl$ (3 × 15 mL). The organic layer was then dried ($MgSO_4$) and evaporated *in vacuo*. The crude mesylate was taken forward without further purification.

To a stirring solution of the mesylate (0.228 g, 0.46 mmol) in a MW vial, dissolved in EtOH (4.6 mL), was added dimethyl-benzylamine (0.205 mL, 1.365 mmol). The reaction was heated to 80 °C and left to stir for 16 hrs. The mixture was cooled to RT and the solvent removed *in vacuo*. The mixture was redissolved in DCM (30 mL) was washed with sat. aq. $NaHCO_3$ (2 × 15 mL), $H_2O$ (15 mL), and sat. brine (15 mL). The organic phase was dried ($MgSO_4$), and the solvent removed *in vacuo*. Purification by silica column chromatography (0–5% MeOH/DCM) gave the product **S23** as a pale oil (0.153 g, 0.24 mmol, 63 % yield). **$^1$H NMR** (400 MHz, $CDCl_3$) δ 7.65–7.41 (m, 5H), 4.81 (s, 2H), 4.04 (d, *J* = 5.4 Hz, 2H), 3.90–3.83 (m, 2H), 3.73–3.47 (m, 12H), 3.40 (t, *J* = 6.8 Hz, 2H), 3.25 (s, 6H), 2.85 (t, *J* = 7.4 Hz, 2H), 2.81 (s, 3H, $MsO^-$), 2.32 (s, 3H), 1.61–1.48 (m, 4H), 1.39–1.18 (m, 12H). **$^{13}$C NMR** (101 MHz, $CDCl_3$) δ 196.08, 133.41, 130.66, 129.18, 127.43, 71.49, 70.54, 70.45, 70.34, 70.07, 69.98, 69.42, 65.27, 50.54,

39.58, 30.63, 29.59, 29.53, 29.47, 29.44, 29.42, 29.12, 29.07, 28.78, 26.04. The analytical data was closely analogous to that of the original work (n.b. original work utilised a Trityl (-Trt) protecting group for the thiol, this work uses a thioester (-SAc) here).[13] **ESI-MS** *m/z calcd* for $C_{30}H_{54}NO_5S^+$ $[M]^+$ 540.3717 *found* 540.3725

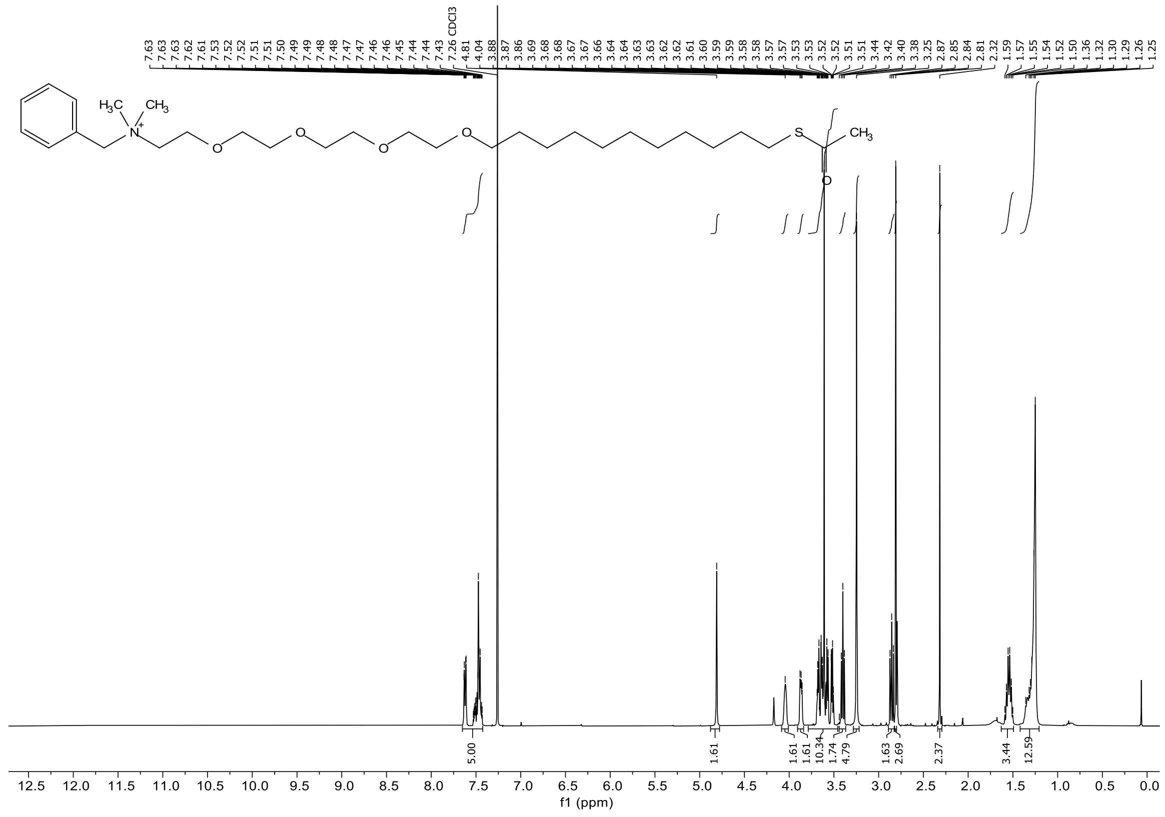

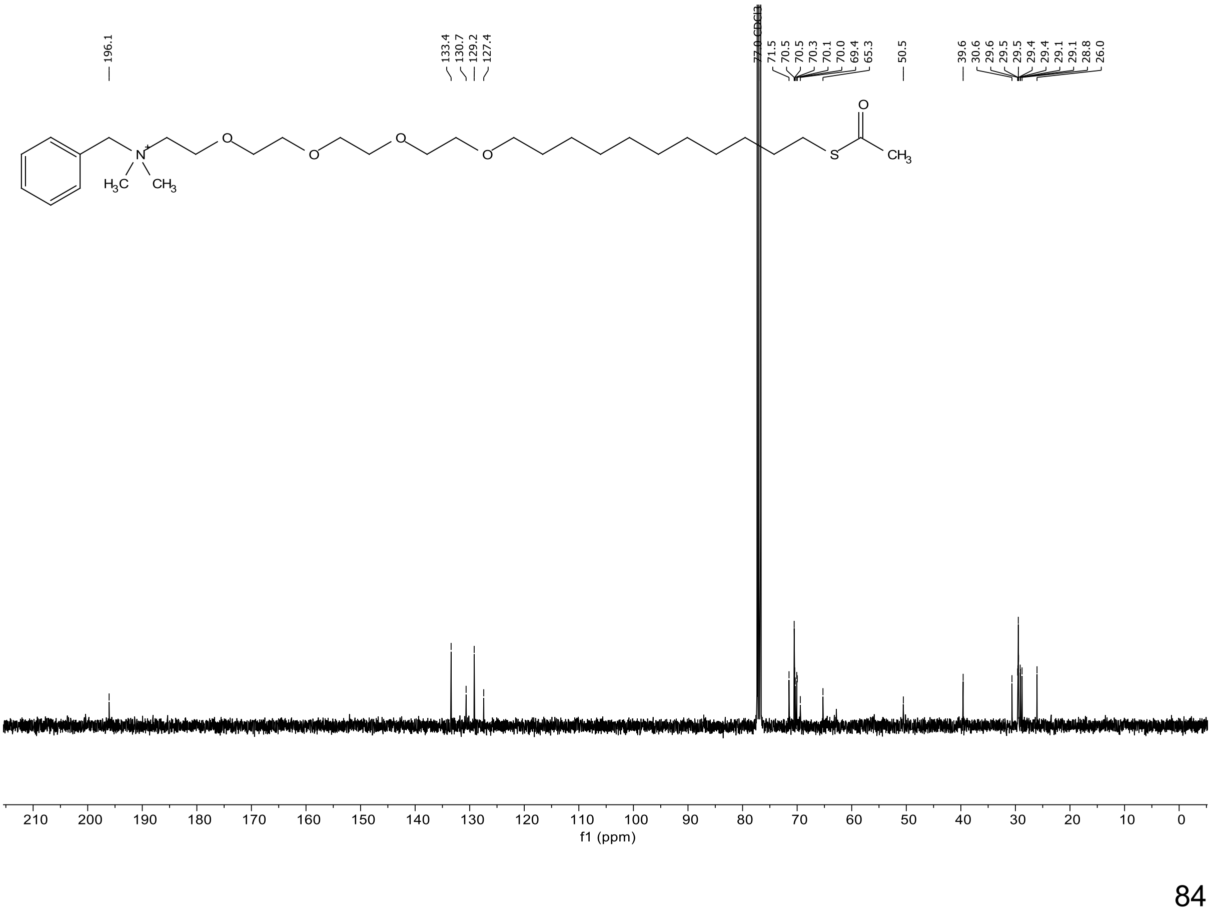

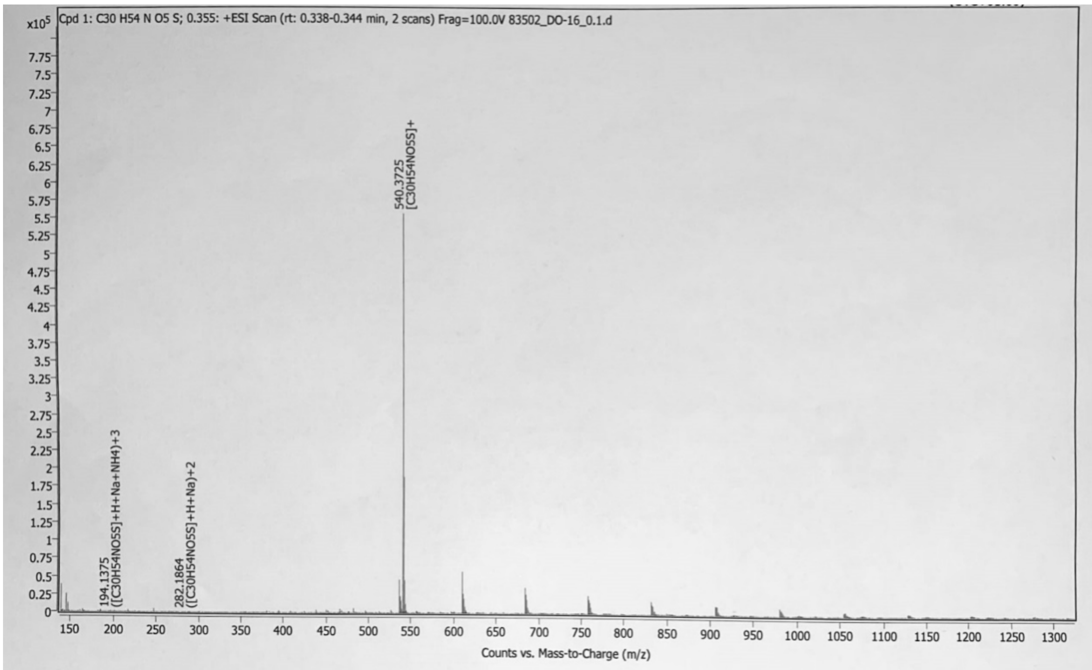


N-benzyl-23-mercapto-N,N-dimethyl-3,6,9,12-tetraoxatricosan-1-aminium chloride **20**

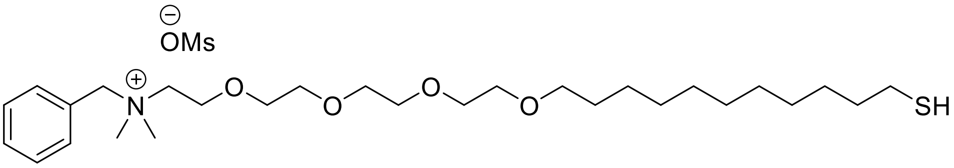


Thioester **S23** was deprotected using methanolic HCl (1.25M, >10 eq.), 3 hrs at 55 °C to give the thiol. $^1$H NMR (400 MHz, $CDCl_3$) δ 7.67–7.42 (m, 5H), 4.80 (s, 2H), 4.05 (br, 2H), 3.85 (br, 2H), 3.73–3.50 (m, 12H), 3.40 (t, *J* = 6.9z, 2H), 3.24 (s, 6H), 2.87 (s, 3H, $MsO^-$), 2.52 (q, *J* = 7.4 Hz, 2H), 1.66–1.49 (m, 4H), 1.42–1.23 (m, 14H). **ESI-MS** *m/z calcd* for $C_{28}H_{52}NO_4S^+$ $[M]^+$ 498.3612 *found* 498.3620.

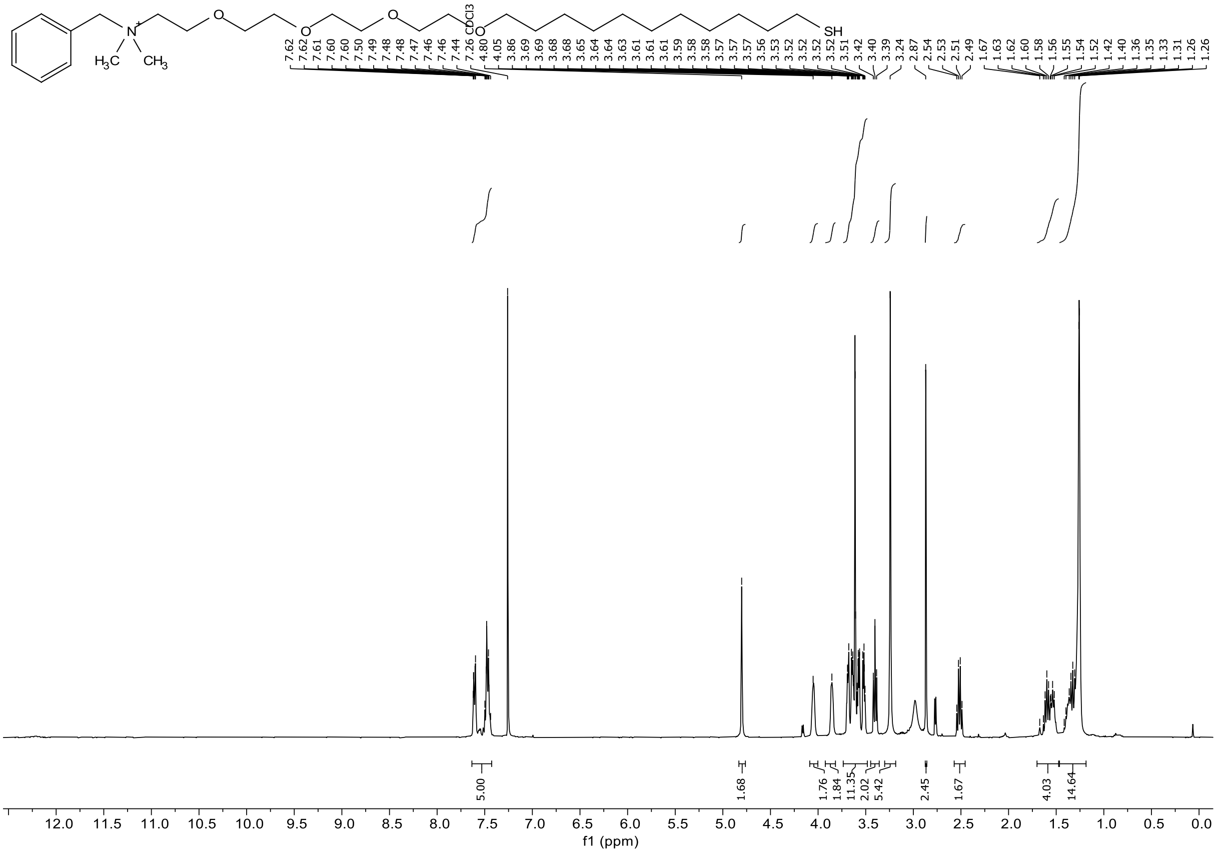

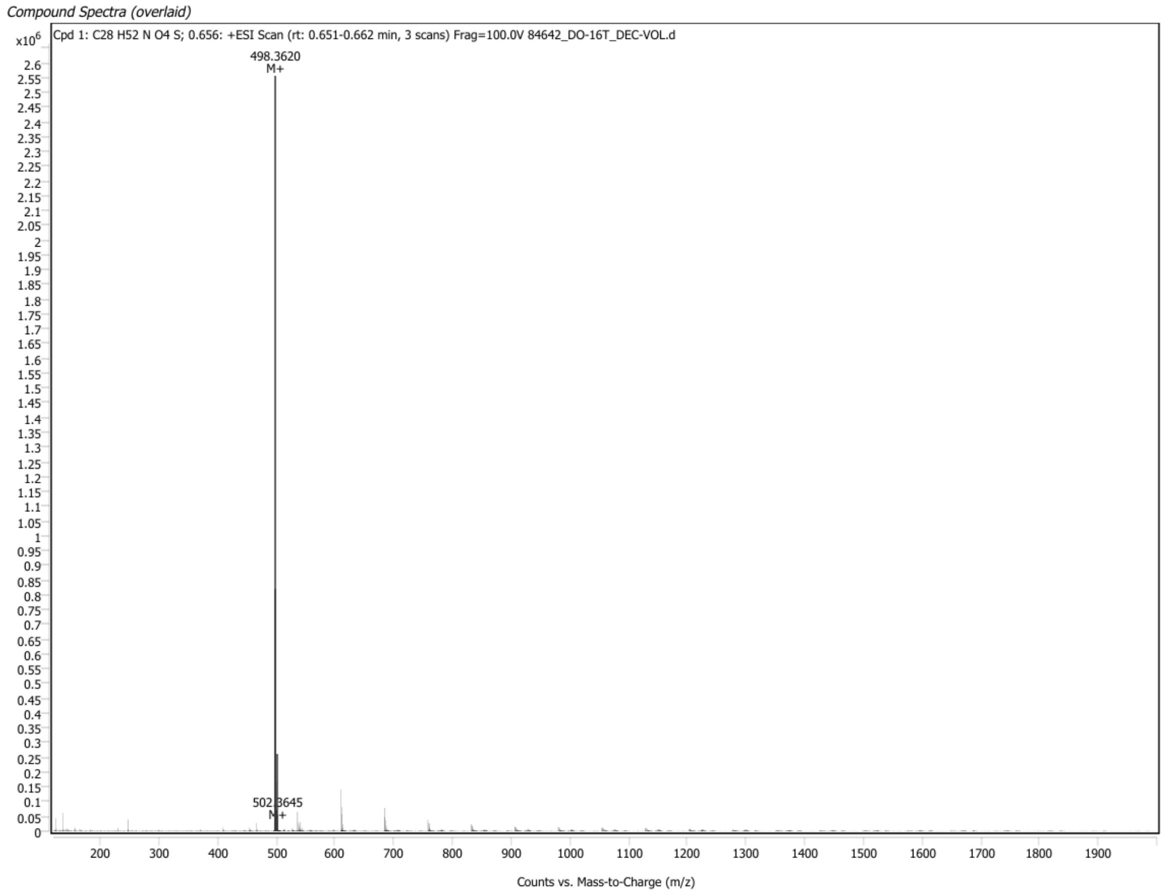
Compound Spectra (overlaid)
Cpd 1: C28 H52 N O4 S; 0.656: +ESI Scan (rt: 0.651-0.662 min, 3 scans) Frag=100.0V 84642_DO-16T_DEC-VOL.d
498.3620
M+
502.3645
M+
Counts vs. Mass-to-Charge (m/z)

N-(3-hydroxypropyl)-N,N-dimethyl-25-oxo-3,6,9,12-tetraoxa-24-thiahexacosan-1-aminium bromide **S24**

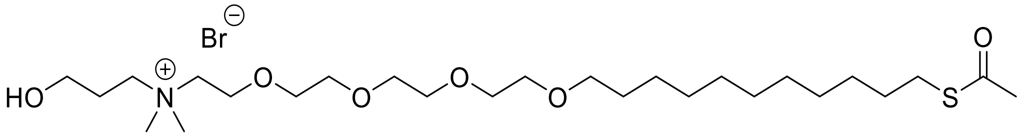


To a stirring solution of bromide **S21** (0.063 g, 0.13 mmol) in a 1:4 DCM/EtOH solution (0.70 mL), was added 3-Dimethylamino-1-propanol dropwise (0.05 mL, 0.39 mmol). The reaction was heated to 50 °C and left to stir for 48 h. The solvent was then removed *in vacuo* and purified by trituration with cold hexane:ether (1:1, 2 mL). The residue was further dried and the product **S24** was obtained as colourless oil (0.071 g, 0.121 mmol, 92%). Analytical data was closely analogous to that of the original work (n.b. original work utilised a Trityl (-Trt) protecting group for the thiol, this work uses a thioester (-SAc) here).[14] **$^1$H NMR** (400 MHz, $CDCl_3$) δ 4.00 (br.s, 2H) 3.90–3.79 (m, 5H), 3.79–3.55 (m, 16H), 3.44 (t, *J* = 6.9 Hz, 2H), 3.36 (s, 6H), 2.86 (t, *J* = 7.4 Hz, 2H), 2.32 (s, 3H), 2.15–2.02 (m, 2H), 1.55 (d, *J* = 7.2 Hz, 2H), 1.20–1.30 (m, 16H). **$^{13}$C NMR** (101 MHz, $CDCl_3$) δ 196.14, 71.54, 70.57, 70.48, 70.42, 70.21, 70.20, 70.01, 64.98, 64.28, 58.37, 52.21, 30.67, 29.70, 29.62, 29.57, 29.50, 29.47, 29.45, 29.16, 29.11, 28.81, 26.07. **ESI-MS** *m/z calcd* for $C_{26}H_{54}NO_6S^+$ $[M]^+$ 508.3666 *found* 508.3678.

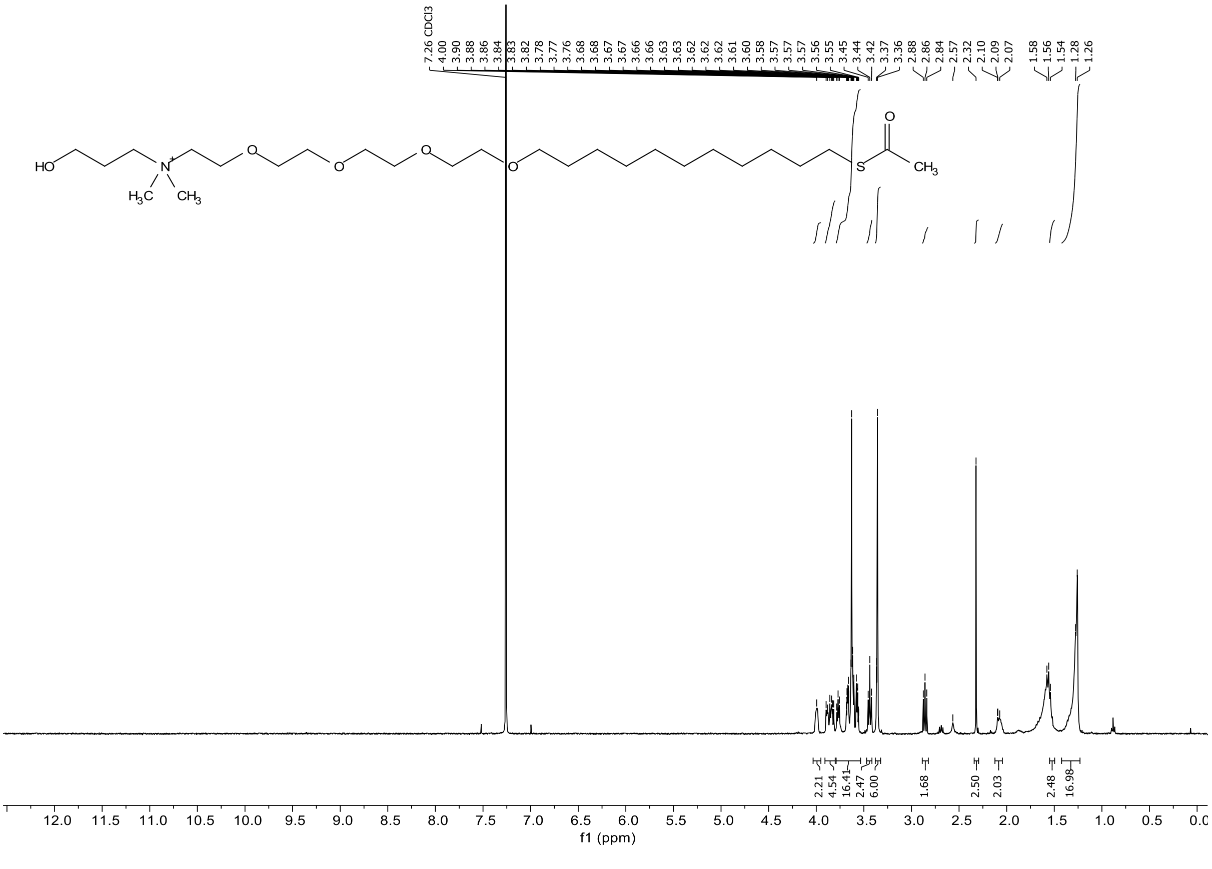

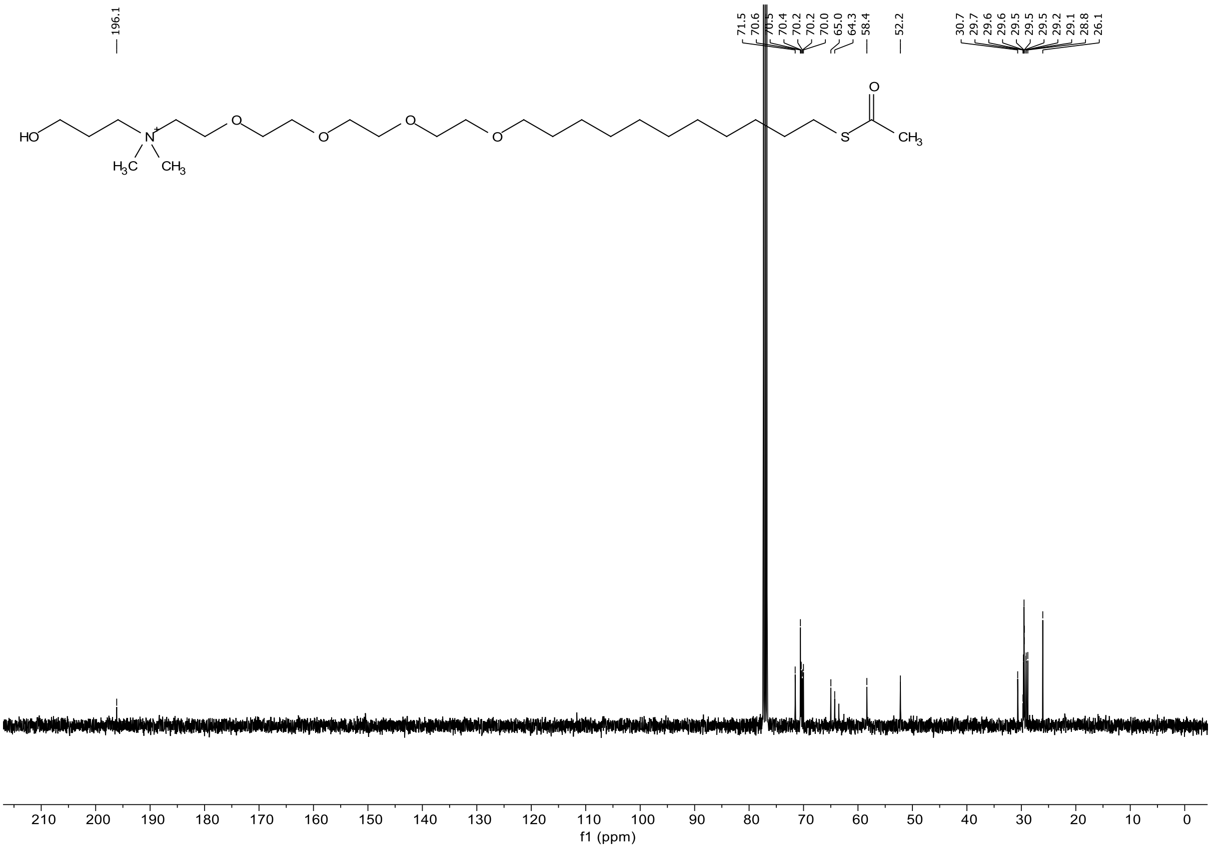


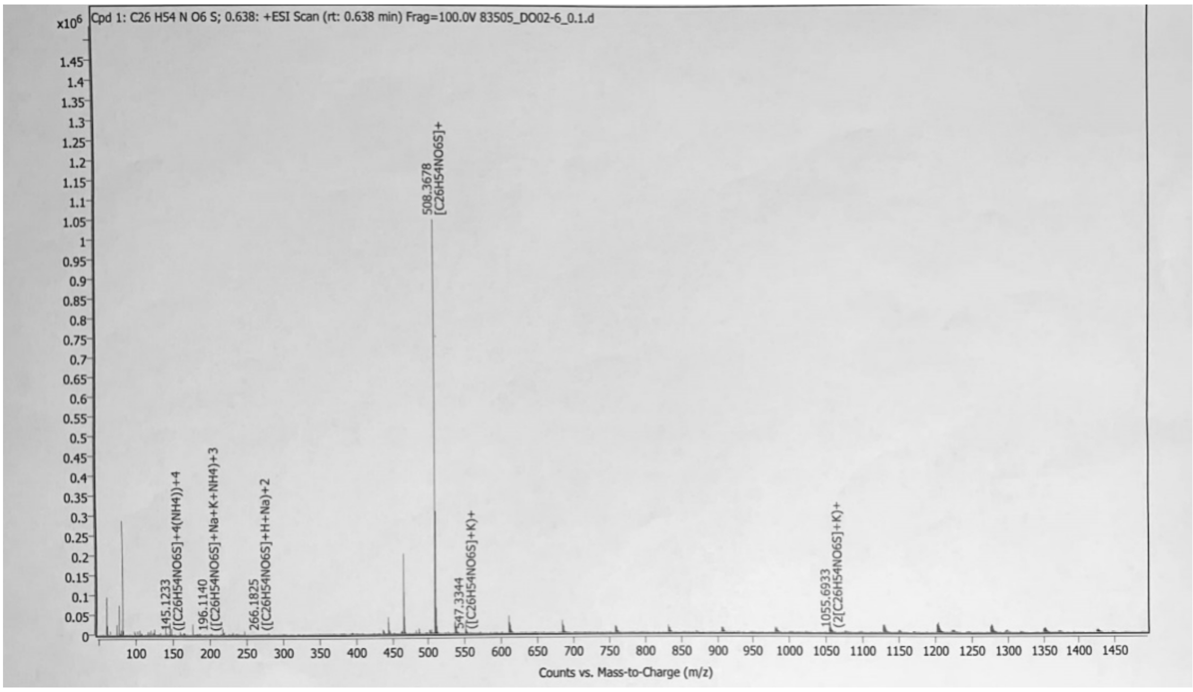


N-(3-hydroxypropyl)-23-mercapto-N,N-dimethyl-3,6,9,12-tetraoxatricosan-1-aminium chloride **21**

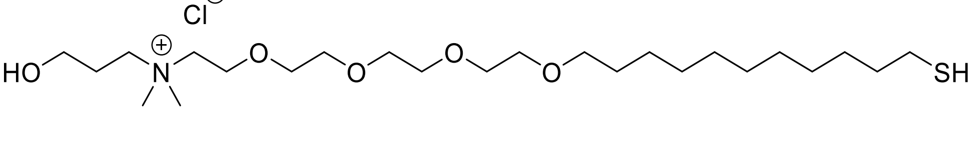

Thioester **S24** was deprotected using methanolic HCl (1.25M, >10 eq.), for 3 hrs at 55 °C to give the thiol **21**. $^{1}H$ NMR (400 MHz, $CDCl_3$) δ 3.99 (br, 2H), 3.89–3.79 (m, 4H), 3.77–3.73 (m, 2H), 3.70–3.55 (m, 14H), 3.43 (t, *J* = 6.9 Hz, 2H), 3.36 (s, 6H), 2.52 (q, *J* = 7.5 Hz, 2H), 1.73–1.50 (m, 4H), 1.41–1.24 (m, 16H). **ESI-MS** *m/z calcd* for $C_{24}H_{52}NO_5S^+$ [M]$^+$ 466.3561 *found* 466.3561.

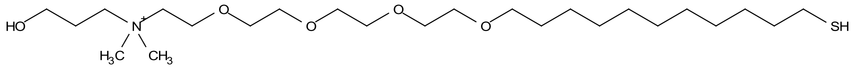


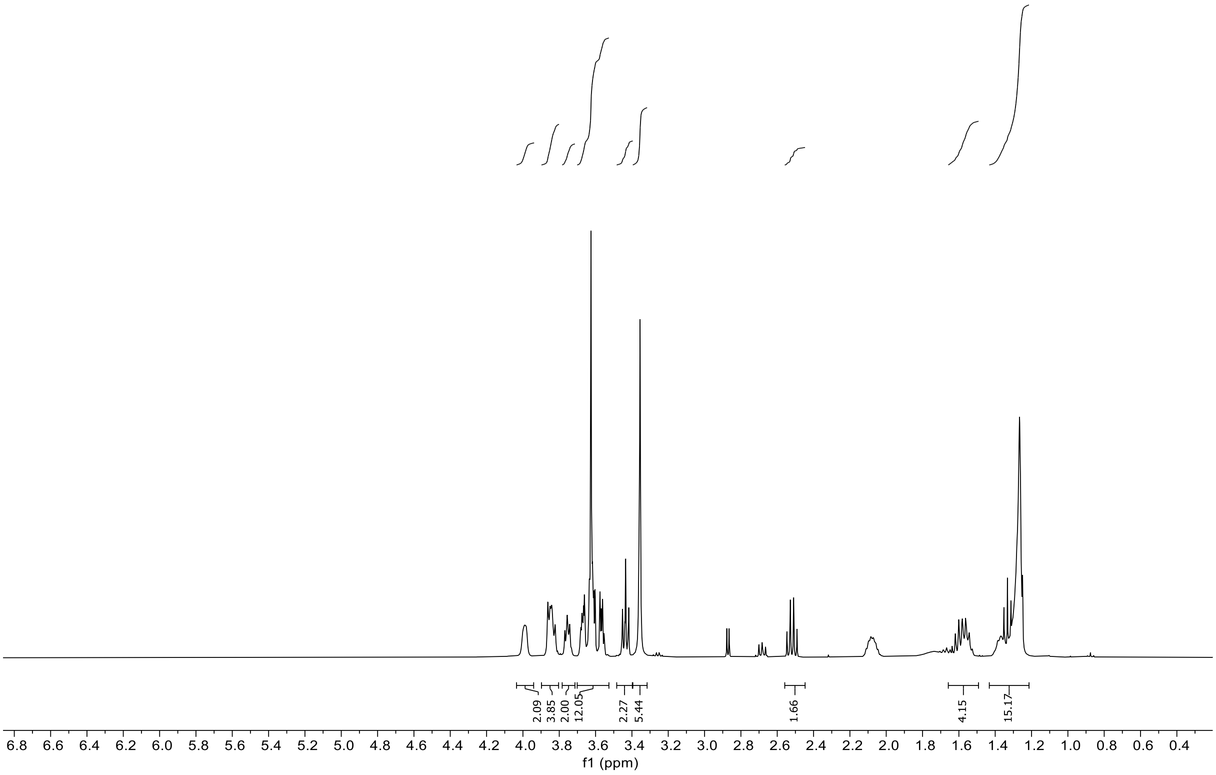

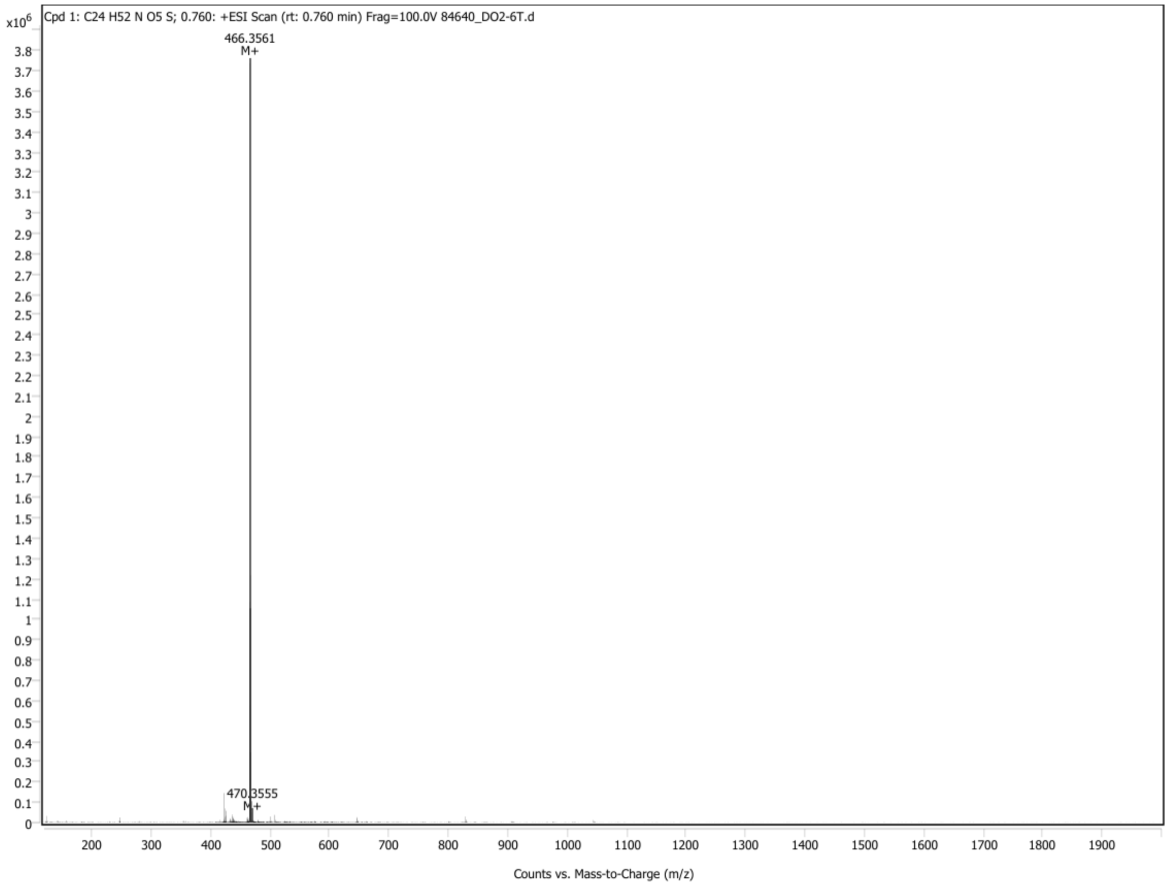


**Synthesis of Chelator 22**

2,2'-((1-Carboxy-5-(4-mercaptobutanamido)pentyl)azanediyl)diacetic acid **22**

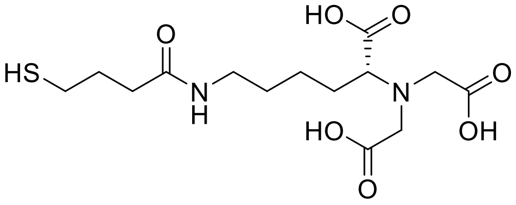

Procedure was adapted from Du Roure *et al.*[15] *Nα,Nα*-Bis(carboxymethyl)-L-lysine hydrate (NTA-Lysine, 0.100 g, 0.357 mmol) was reacted with γ-thiobutyrolactone (0.060 g, 0.587 mmol) and $NaHCO_3$ (0.100 g, 1.19 mmol) in 1 mL of $H_2O$ for 15 hrs at 72 °C. The solution was cooled to 0 °C and acidified to pH 3 with AcOH (*ca.* 0.1 mL). The solvent was removed in vacuo leaving behind a pale orange paste. The crude product was recrystallised using EtOH (10 mL) and hexanes (10 mL) yielding **22** as a white hygroscopic solid on the walls of the flask (0.103 g, 0.282 mmol, 79 %). Observed analytical data were in accordance with literature values.[15] **$^1$H NMR** (400 MHz, $D_2O$) δ 3.77 (br, 5H), 3.21 (br, 2H), 2.75 (t, J = 7.1 Hz, 2H), 2.37 (t, J = 7.3 Hz, 2H), 2.00 (app. p, J = 7.0 Hz, 2H), 1.95 – 1.77 (m, 2H), 1.64 – 1.40 (m, 4H). **ESI-MS** *m/z calcd* for $C_{14}H_{24}N_2O_7S$ [M]+ 364.1304 *found* 364.1297.

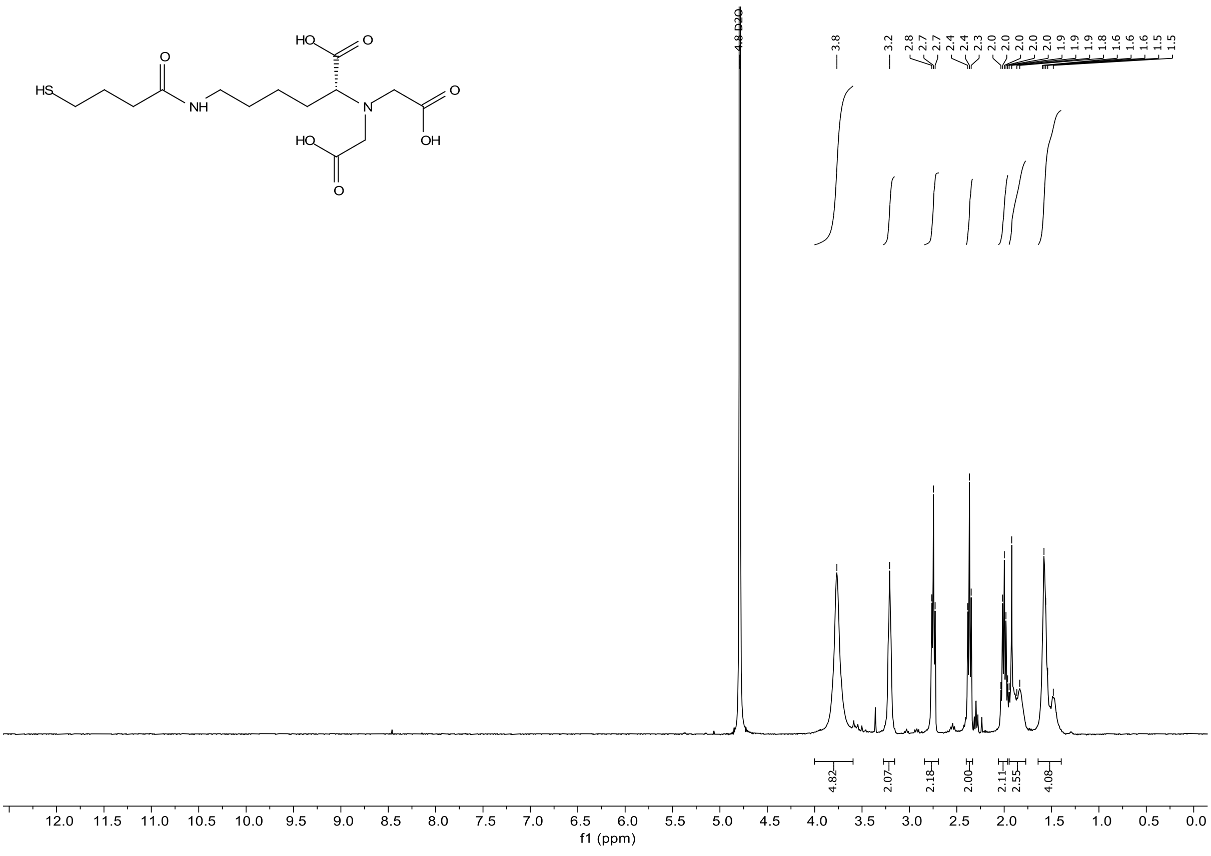
HS
NH
HO
O
N
OH
4.8 D2O
3.8
3.2
2.8
2.7
2.7
2.4
2.4
2.3
2.0
2.0
2.0
2.0
2.0
1.9
1.9
1.8
1.6
1.6
1.5
1.5
4.82
2.07
2.18
2.00
2.11
2.55
4.08
12.0 11.5 11.0 10.5 10.0 9.5 9.0 8.5 8.0 7.5 7.0 6.5 6.0 5.5 5.0 4.5 4.0 3.5 3.0 2.5 2.0 1.5 1.0 0.5 0.0
f1 (ppm)

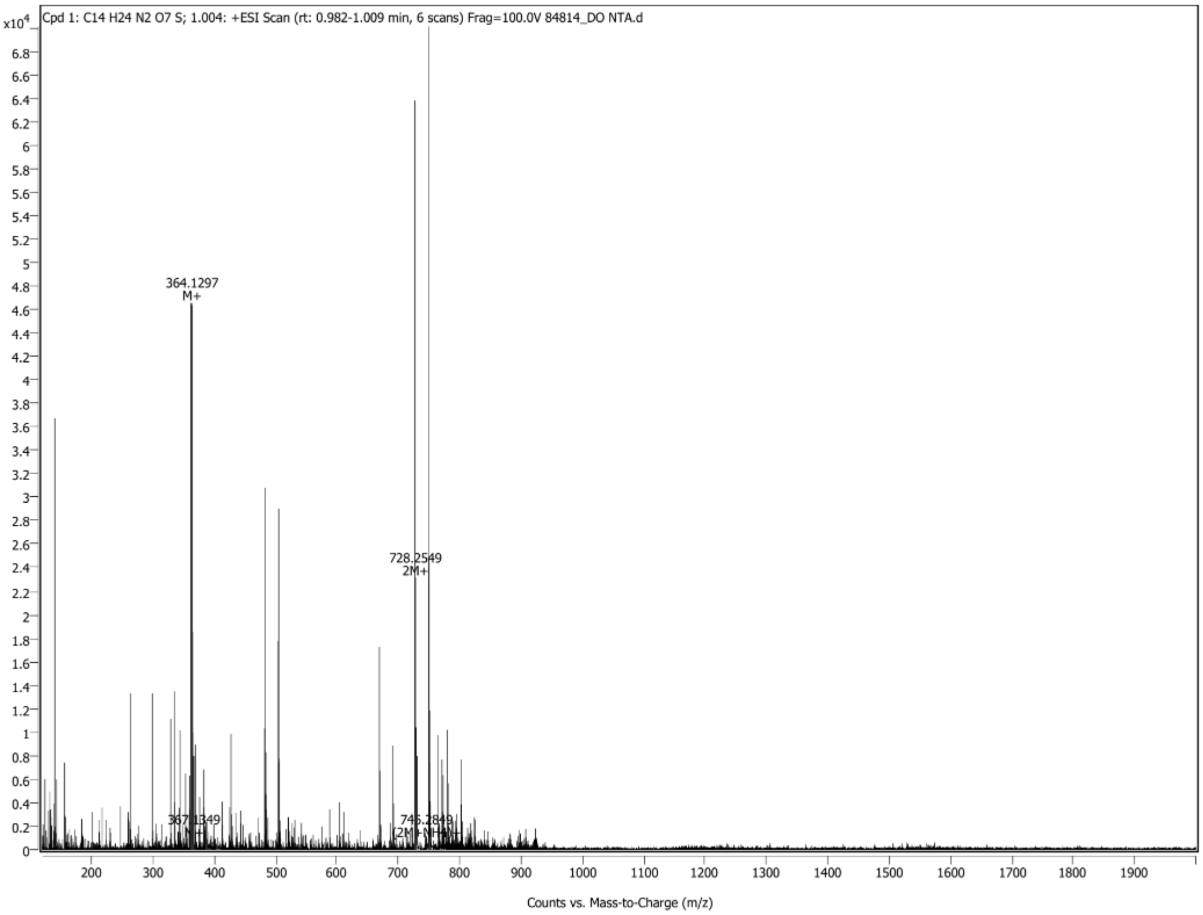
Cpd 1: C14 H24 N2 O7 S; 1.004: +ESI Scan (rt: 0.982-1.009 min, 6 scans) Frag=100.0V 84814_DO NTA.d
x10^4
364.1297
M+
728.2549
2M+
367.1349
745.2849
(2M+NH4)+
Counts vs. Mass-to-Charge (m/z)

## Synthesis of Macrocycles 23 and 24

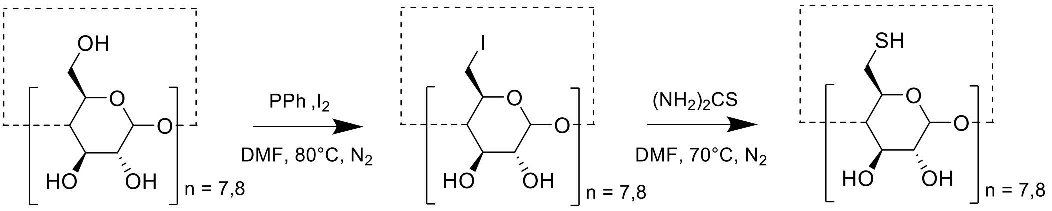


### Per-6-Functionalised Beta-CD

Per-6-iodo-β-cyclodextrin **S25**

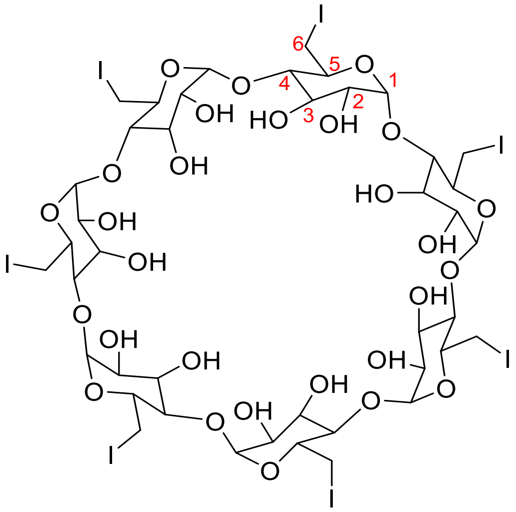

Compound prepared from β-cyclodextrin as described by Defaye and Gadelle.[16] A solution of $PPh_3$ (36.5 g, 139 mmol) and Iodine (35.1 g, 139 mmol) was stirred in anhydrous DMF (139 mL) for 15 mins. β-cyclodextrin (7.50 g, 6.61 mmol) and the solution was stirred at 80 °C for 15 hrs. The solution was concentrated *in vacuo* to half the volume and the pH was adjusted to 9-10 by addition of NaOMe in MeOH (28.9 mL of 5.4 M stock solution) with simultaneous cooling in an ice bath. The solution was warmed to RT for 30 mins to destroy the formate esters in the reaction. Ice water (400 mL) was poured into the reaction mixture to precipitate the product and the solid was collected by filtration. The product was then Soxhlet extracted with (*ca.* 300 mL) MeOH for 20 h. The product was then rigorously dried under high vacuum (rotary evaporator) to yield the product **S25** as pale yellow solid (3.451 g, 1.81 mmol, 27%). Observed analytical data matched that of the original work. **$^1$H NMR** (400 MHz, DMSO-d$_6$) $\delta_H$ 6.04 (d, J = 6.7 Hz, 7H, 2-OH), 5.93 (s, 7H, 3-OH), 4.99 (d, J = 2.65 Hz, 7H, 1-H), 3.81 (d, J = 9.79 Hz, 7H, 6b-H), 3.70–3.56 (m, 22H, 3-H, 5-H), 3.27–3.49 (m, 14H, 2-H, 4-H, 6a-H) **ESI-MS** *m/z calcd* for $C_{42}H_{63}I_7O_{28}$ $[M+Na]^+$ 1926.6711 *found* 1926.6692.

Per-6-thio-β-cyclodextrin **23**

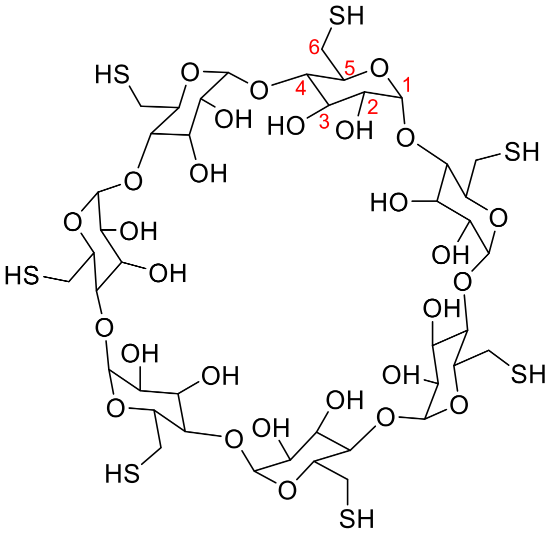


Adapted from the prep by Rojas *et al.*,[17] Iodinated cyclodextrin **S25** (0.965g, 0.507 mmol) was dissolved in anhydrous DMF (10 mL), thiourea (0.301 g, 3.95 mmol) was then added and the reaction was heated to 70 °C under a nitrogen atmosphere. After 19 h, the DMF was removed *in vacuo* to give an orange oil, which was dissolved in water (50 mL). Sodium hydroxide (0.26 g, 6.50 mmol) was added and the reaction mixture heated to a gentle reflux under a nitrogen atmosphere. After 1 h, the resulting suspension was acidified with sat. aqueous $KHSO_4$ (*ca.* 50 mL) and the precipitate filtered off, washed thoroughly with distilled water, and dried (superficially by filtration). To remove the last traces of DMF, the product was suspended in water (50 mL) and the minimum amount of potassium hydroxide added to give a clear, light yellow solution; the product was then reprecipitated by acidifying with aqueous $KHSO_4$ to *ca.* pH 3. The resulting fine precipitate was carefully filtered, resuspended in EtOH (5 mL) and evaporated *in vacuo* to yield **23** as an off-white (orange tone) powder (0.190 g, 0.15 mmol, 30%). Observed analytical data matched that of the original work. **$^1$H NMR** (400 MHz, DMSO-d$_6$) $\delta_H$ 5.92 (d, $J$ = 6.9 Hz, 7H, 2-OH), 5.81 (s, 7H, 3-OH), 4.92 (s, 7H, 1-H), 3.61 (t, $J$ = 8.6 Hz, 14H, 3-H, 5-H), 3.19 (br.d, $J$ = 1.8 Hz, 5H, 6b-H), 2.72–2.79 (m, 8H, 6a-H), 2.13 (t, $J$ = 7.6 Hz, 7H, SH). **ESI-MS** *m/z calcd* for $C_{42}H_{70}O_{28}S_7$ [M + Na]$^+$ 1269.1991 *found* 1269.1974.

**Per-6-Functionalised Gamma-CD**

Per-6-iodo-γ-cyclodextrin **S26**

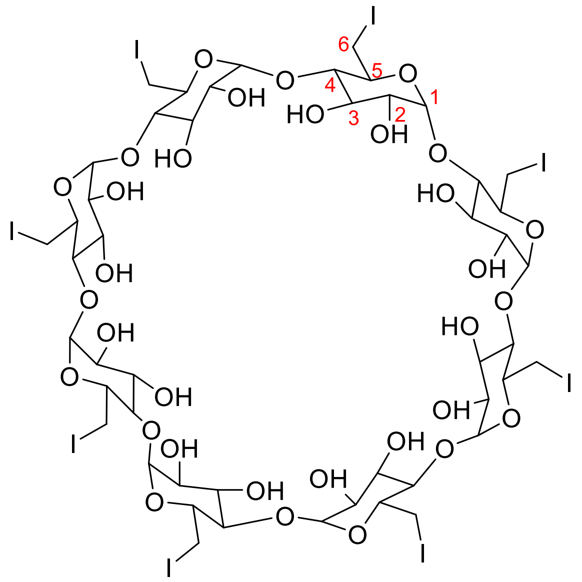


Adapted from Rojas *et al.,*[17] under $N_2$, $PPh_3$ (21.0 g, 80 mmol) and $I_2$ (20.5 g, 80 mmol) were dissolved in anhydrous DMF (80 mL). The reaction mixture was stirred for 10 mins, then γ-cyclodextrin (4.32 g, 3.33 mmol) was added and stirred for 16 hrs at RT. After concentrating *in vacuo* to around half volume, the pH was adjusted to 9–10 by the addition of 5.4 M sodium methoxide in methanol (*ca.* 5 mL), and the reaction was stirred at RT for 30 min. The mixture was poured into ice water (*ca.* 900 mL), and the resulting light brown precipitate was collected by filtration. The precipitate was washed extensively with DCM (3 × 20 mL) (to remove triphenyl phosphine oxide). Once the solid was dried, the product **S26** was obtained as a light brown solid (4.882 g, 2.57 mmol, 70%). Some TPPO remained (ca. 0.4 eq), likely trapped in the cavity of the larger gamma-CD. **$^1$H NMR** (400 MHz, DMSO-d$_6$) $\delta_H$ 6.2–5.9 (m, 16H) 5.03 (d, J = 3.52 Hz, 8H, 1-H), 3.82 (d, J = 9.79 Hz, 8H, 6b-H), 3.70–3.50 (m, 24H, 3-H, 5-H), 3.45–3.20 (m, 16H, 2-H, 4-H, 6a-H).

Per-thio-γ-cyclodextrin **24**

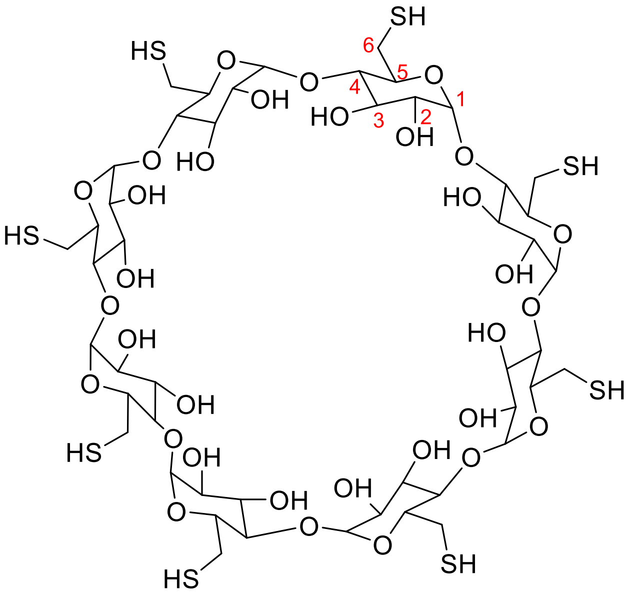

Adapted from procedure to make Compound **23**: Compound **S26** (2.499 g, 1.15 mmol) was dissolved in DMF (25 mL); thiourea (0.768 g, 10.1 mmol) was then added and the reaction mixture heated to 70 °C under an $N_2$ atmosphere. After 19 h, the DMF evaporated *in vacuo* to give an yellow oil, which was dissolved in water. Aq. NaOH solution (0.12 M, 125 mL) was added and the reaction mixture heated to a gentle reflux under a nitrogen atmosphere. After 1 hr, the resulting suspension was acidified with sat. aqueous $KHSO_4$ (*ca.* 100 mL) to pH 3 and the precipitate filtered off, washed thoroughly with distilled water, and dried (superficially by filtration). To remove the last traces of DMF, the product was suspended in water (*ca.* 100 mL) and the minimum amount of potassium hydroxide added to give a clear solution; the product was then reprecipitated by acidifying with sat. aqueous $KHSO_4$ to around pH 3. The resulting fine precipitate was carefully filtered off and dried under vacuum to yield **24** (0.523g, 0.367 mmol, 32 % yield) as an off-white powder.**$^1$H NMR** (400 MHz, DMSO-$d_6$) $\delta_H$ 5.92 (d, $J$ = 6.9 Hz, 7H, 2-OH), 5.81 (s, 7H, 3-OH), 4.92 (s, 7H, 1-H), 3.61 (t, $J$ = 8.6 Hz, 14H, 3-H, 5-H), 3.19 (br.d, $J$ = 1.8 Hz, 5H, 6b-H), 2.72–2.79 (m, 8H, 6a-H), 2.13 (t, $J$ = 7.6 Hz, 7H, SH). **ESI-MS** *m/z calcd* for $C_{48}H_{80}O_{32}S_8$ $[M + Na]^+$ 1447.2291 *found* 1447.2259.

## Supplementary References


1. Sperling, J. R. *et al.* A cross-reactive plasmonic sensing array for drinking water assessment. *Environmental Science: Nano* **10**, 3500–3508 (2023).

2. Yuan, M. *et al.* A Method for Removing Self-Assembled Monolayers on Gold. *Langmuir* **24**, 8707–8710 (2008).

3. Ihde, M. H., Pridmore, C. F. & Bonizzoni, M. Pattern-Based Recognition Systems: Overcoming the Problem of Mixtures. *Analytical Chemistry* **92**, 16213–16220 (2020).

4. Holmlin, R. E., Chen, X., Chapman, R. G., Takayama, S. & Whitesides, G. M. Zwitterionic SAMs that Resist Nonspecific Adsorption of Protein from Aqueous Buffer. *Langmuir* **17**, 2841–2850 (2001).

5. Thebault, P. *et al.* Preparation and antimicrobial behaviour of quaternary ammonium thiol derivatives able to be grafted on metal surfaces. *European Journal of Medicinal Chemistry* **44**, 717–724 (2009).

6. Salvio, R. & Cincotti, A. Guanidine based self-assembled monolayers on Au nanoparticles as artificial phosphodiesterases. *RSC Advances* **4**, 28678–28682 (2014).

7. Pale-Grosdemange, C., Simon, E. S., Prime, K. L. & Whitesides, G. M. Formation of Self-Assembled Monolayers by Chemisorption of Derivatives of Oligo(ethylene glycol) of Structure HS(CH2)11(OCH2CH2)mOH on Gold. *Journal of the American Chemical Society* **113**, 12–20 (1991).

8. Medintz, I. L. *et al.* Proteolytic activity monitored by fluorescence resonance energy transfer through quantum-dot–peptide conjugates. *Nature Materials* **5**, 581–589 (2006).

9. Hong, R. *et al.* Control of Protein Structure and Function through Surface Recognition by Tailored Nanoparticle Scaffolds. *Journal of the American Chemical Society* **126**, 739–743 (2004).

10. Chu, Z., Han, Y., Král, P. & Klajn, R. “Precipitation on Nanoparticles”: Attractive Intermolecular Interactions Stabilize Specific Ligand Ratios on the Surfaces of Nanoparticles. *Angewandte Chemie International Edition* **57**, 7023–7027 (2018).

11. Dutta, P., Sawoo, S., Ray, N., Bouloussa, O. & Sarkar, A. Engineering Bioactive Surfaces with Fischer Carbene Complex: Protein A on Self-Assembled Monolayer for Antibody Sensing. *Bioconjugate Chemistry* **22**, 1202–1209 (2011).

12. Pandit, S., Karunakaran, S., Boda, S. K., Basu, B. & De, M. High Antibacterial Activity of Functionalized Chemically Exfoliated MoS2. *ACS Applied Materials & Interfaces* **8**, 31567–31573 (2016).

13. Tonga, G. Y. *et al.* Supramolecular regulation of bioorthogonal catalysis in cells using nanoparticle-embedded transition metal catalysts. *Nature Chemistry* **7**, 597–603 (2015).

14. Miranda, O. R. *et al.* Enzyme-Amplified Array Sensing of Proteins in Solution and in Biofluids. *Journal of the American Chemical Society* **132**, 5285–5289 (2010).

15. Du Roure, O., Debiemme-Chouvy, C., Malthête, J. & Silberzan, P. Functionalizing Surfaces with Nickel Ions for the Grafting of Proteins. *Langmuir* **19**, 4138–4143 (2003).

16. Gadelle, A. & Defaye, J. Selective Halogenation at Primary Positions of Cyclomaltooligosaccharides and a Synthesis of Per-3,6-anhydro Cyclomaltooligosaccharides. *Angewandte Chemie International Edition in English* **30**, 78–80 (1991).

17. Rojas, M. T., Koeniger, R., Stoddart, J. F. & Kaifer, A. E. Supported Monolayers Containing Preformed Binding Sites. Synthesis and Interfacial Binding Properties of a Thiolated .beta.-Cyclodextrin Derivative. *Journal of the American Chemical Society* **117**, 336–343 (1995).
